# Diruthenium Tetracarboxylate-Catalyzed Enantioselective Cyclopropanation with Aryldiazoacetates

**DOI:** 10.1021/acs.organomet.3c00268

**Published:** 2023-06-30

**Authors:** Joshua K. Sailer, Jack C. Sharland, John Bacsa, Caleb F. Harris, John F. Berry, Djamaladdin G. Musaev, Huw M. L. Davies

**Affiliations:** † Department of Chemistry, Emory University, 1515 Dickey Drive, Atlanta, Georgia 30322, United States; ‡ Department of Chemistry, University of Wisconsin, 1101 University Avenue, Madison, Wisconsin 53706, United States; § Cherry L. Emerson Center for Scientific Computation, Emory University, 1521 Dickey Drive, Atlanta, Georgia 30322, United States

## Abstract

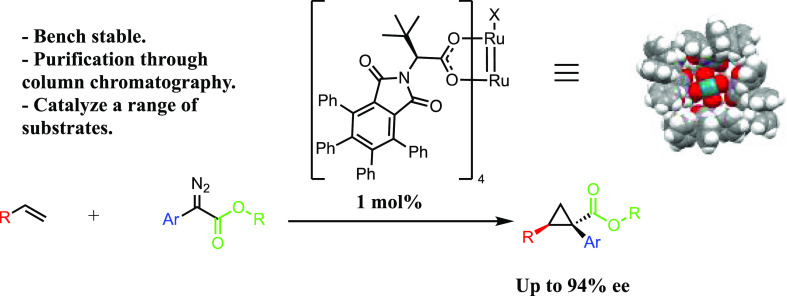

A series of chiral bowl-shaped diruthenium(II,III) tetracarboxylate catalysts were prepared and evaluated in asymmetric cyclopropanations with donor/acceptor carbenes derived from aryldiazoacetates. The diruthenium catalysts self-assembled to generate *C*_4_-symmetric bowl-shaped structures in an analogous manner to their dirhodium counterparts. The optimum catalyst was found to be Ru_2_(*S*-TPPTTL)_4_·BAr^F^ [*S*-TPPTTL = (*S*)-2-(1,3-dioxo-4,5,6,7-tetraphenylisoindolin-2-yl)-3,3-dimethylbutanoate, BAr^F^ = tetrakis(3,5-bis(trifluoromethyl)phenyl)borate], which resulted in the cyclopropanation of a range of substrates in up to 94% ee. Synthesis and evaluation of first-row transition-metal congeners [Cu(II/II) and Co(II/II)] invariably resulted in catalysts that afforded little to no asymmetric induction. Computational studies indicate that the carbene complexes of these dicopper and dicobalt complexes, unlike the dirhodium and diruthenium systems, are prone to the loss of carboxylate ligands, which would destroy the bowl-shaped structure critical for asymmetric induction.

## Introduction

Dirhodium tetracarboxylates are exceptional catalysts for carbene group transfer reactions.^1^ During the ligand exchange, chiral ligands can self-assemble to generate high symmetry catalysts capable of high levels of asymmetric induction^2^ and turnover numbers.^3^ The recent advances in generating diazo compounds in flow have greatly increased the practical relevance of this chemistry,^4^ and the dirhodium-catalyzed cyclopropanation chemistry of donor/acceptor carbenes has been used for scale-up synthesis of pharmaceutically relevant targets.^5^ Due to the success of the dirhodium catalysts, there has been considerable interest in developing cheaper dimetallic lantern complexes as replacement catalysts.

**Scheme 1:**
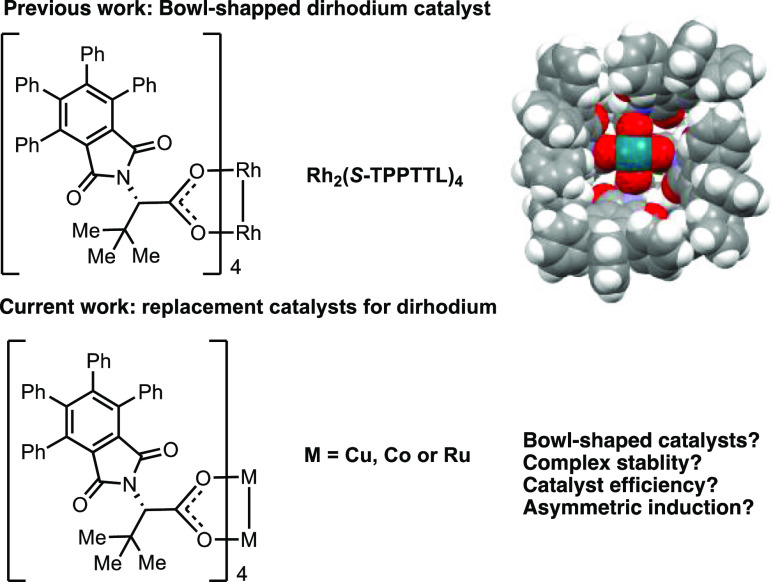
Previous and Current Work

This study describes the synthesis and evaluation of chiral dimetallic complexes for asymmetric cyclopropanation with donor/acceptor carbenes (Scheme 1). We primarily focused on the use of the tetraphenylphthalimido *tert*-leucinato (TPPTTL) chiral ligand because it generates a well-defined bowl-shaped dirhodium complex capable of a range of synthetically useful reactions.^3a,4c,6^ A suitable replacement catalyst would need to display high catalytic activity, similar self-assembly of the ligands to form the bowl-shaped catalyst pocket, and sufficient stability of the complexes during the desired transformations to achieve high asymmetric induction.

The dicopper, dicobalt, and diruthenium tetracarboxylates were selected as the most promising test systems on the basis of the established literature on dimetallic lantern compounds.^7^ Although lantern structures of low-cost transition-metal complexes would be ideal, their application to carbene chemistry has been limited. For example, achiral molybdenum and chromium complexes were shown to be inactive.^7a^ An achiral cobalt complex was found to be catalytically competent in the cyclopropanation of styrene using donor–acceptor carbenes, albeit with low TON (5) and moderate yield.^7a^ Chiral complexes of dicopper,^8^ rhodium/bismuth,^9^ and diruthenium^7c,7d,10^ have been characterized by X-ray crystallography, and in each case, the ligands do appear to self-assemble in a similar way to that of the corresponding dirhodium complexes. No catalytic carbene reactions of the dicopper complex has been reported. The chiral rhodium–bismuth lantern complexes are capable of cyclopropanation reactions with donor/acceptor carbenes and achieve similar levels of asymmetric induction as the corresponding dirhodium complexes, but they react about 1000 times slower.^9a^ Recently, rhodium–bismuth complexes have been shown to be effective catalysts for site-selective and enantioselective C–H functionalization at activated sites.^11^ A chiral diruthenium paddlewheel complex is able to perform the cyclopropanation of styrene derivatives using acceptor/acceptor carbenes derived from iodonium ylides with high yield and enantioselectivity.^7c^ The single example using diazomalonate as the acceptor/acceptor carbene precursor, however, resulted in low levels of asymmetric induction (46% ee).^7c^ Cyclopropanation reactions with donor/acceptor carbenes catalyzed by the diruthenium complex have not been reported.

## Results and Discussion

**Figure 1:**
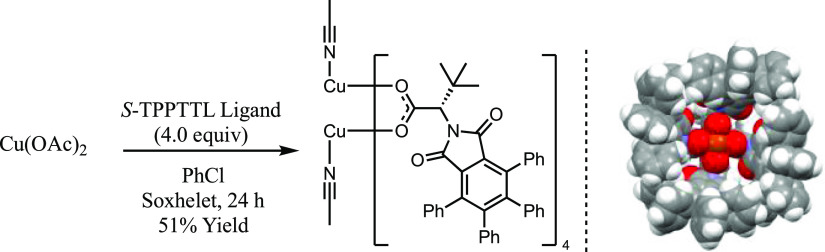
Synthesis and crystal structure of Cu_2_(*S*-TPPTTL)_4_ with axially coordinated MeCN removed for clarity. For full structural details, see the Supporting Information.

This study began by exploring the synthesis and evaluation of the dicopper tetracarboxylates. A wide variety of copper catalysts have been used to decompose diazo compounds,^12^ and the Fox group has previously reported bowl-shaped C_4_-symmetric dicopper complexes.^13^ However, no reactions were described for these complexes. Here, we synthesized Cu_2_(*S*-TPPTTL)_4_ according to the standard ligand exchange reaction^2b^ and found it to be sufficiently stable for purification by chromatography, although the isolated complex coeluted with an extra equivalent of the ligand, *S*-TPPTTL, which presumably is bound to the accessible axial site of the copper complex. Recrystallization of the complex from a dilute acetonitrile (MeCN) solution afforded suitable crystals of Cu_2_(*S*-TPPTTL)_4_ for X-ray crystallographic analysis (Figure 1) and resolved the desired complex from any remaining ligand. The *S*-TPPTTL ligands self-assembled around the dicopper core in a similar way to that of the corresponding dirhodium catalyst, adopting the desired bowl-shaped C_4_-symmetric structure.

Cu_2_(*S*-TPPTTL)_4_ was then tested in the asymmetric cyclopropanation of styrene in the presence of 2,2,2-trichloroethyl 2-(4-bromophenyl)-2-diazoacetate (**2**, Table 1). Cu_2_(*S*-TPPTTL)_4_ (5 mol %) with axially coordinated MeCN was unable to decompose the diazo compound (entry 1) unless it was heated to 40 °C. At this temperature, the cyclopropanation was performed successfully, although the resultant product was isolated essentially as a racemate (entry 2). We hypothesized that the presence of MeCN was inhibiting the ability of Cu_2_(*S*-TPPTTL)_4_ to decompose the diazo compound, and on heating, the bowl-shaped structure was being destroyed. To address this issue, the complex was subjected to high vacuum over several days at room temperature to remove the axial ligating MeCN molecules. During the evacuation, the color of the complex changed from a light blue to dark blue, suggesting that the ligand environment around the copper had changed. This complex was then tested in the cyclopropanation reaction, and this time, it was able to effectively decompose the diazo compound at room temperature and afford the desired product. Unfortunately, the product was still essentially racemic (entry 3), suggesting that Cu_2_(*S*-TPPTTL)_4_ is too unstable to remain intact during the reaction.

**Table 1:**
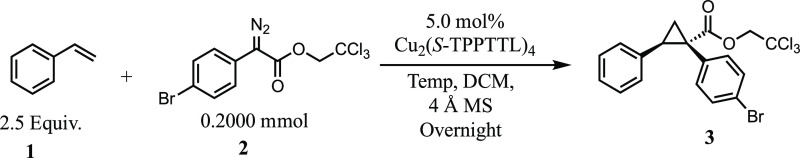
Cu_2_(*S*-TPPTTL)_4_-Catalyzed Cyclopropanation

entry	catalyst	temp (°C)	yield (%)	ee (%)
1	Cu_2_(*S*-TPPTTL)_4_·2MeCN	25	0	N/a
2	Cu_2_(*S*-TPPTTL)_4_·2MeCN	40	73	<5
3	Cu_2_(*S*-TPPTTL)_4_	25	95	<5

**Figure 2:**
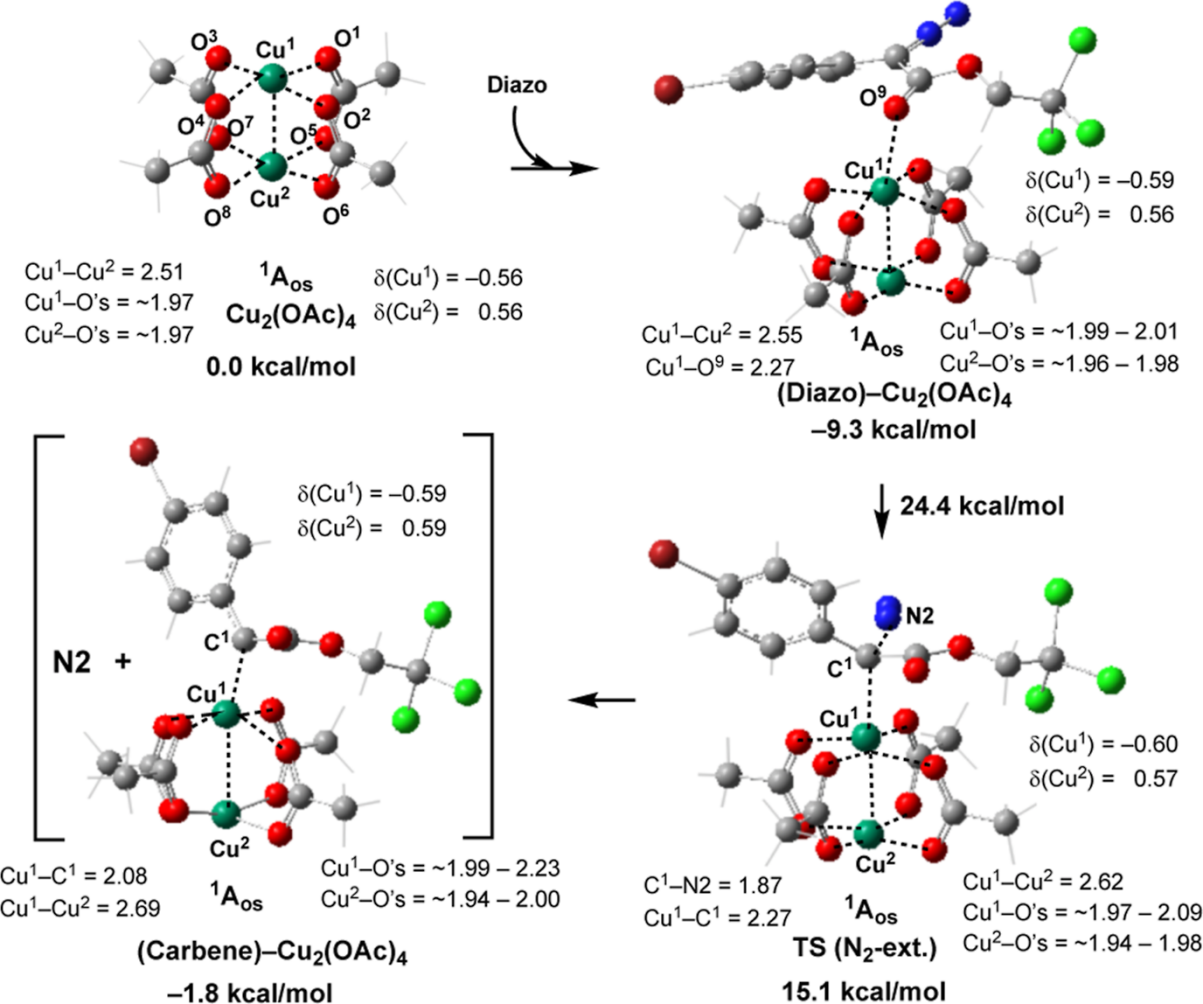
Important geometry (in Å) and free energy (Δ*G*’s) parameters as well as un-paired spin populations, δ (in |e|), of the selected atoms of Cu_2_(OAc)_4_, the **2**–Cu_2_(OAc)_4_ complex, nitrogen extrusion transition state, and final carbene complex.

The experimental findings and hypothesis presented above were further validated by computational analysis of the structure and stability of the (MeCN)–[Cu_2_(OAc)_4_] and 2(MeCN)–[Cu_2_(OAc)_4_] complexes (Figure 2). We also calculated the energetics and potential energy surface (PES) of the diazo decomposition, i.e., reaction L–[Cu_2_(OAc)_4_] + **2** → (Carbene)–[Cu_2_(OAc)_4_] + N_2_ (see Figure 2). These calculations were performed at the [B3LYP-D3(BJ)] + PCM(in DCM)/[6-31G(d,p) + lanl2dz(Cu and Br)] level of theory (see the Supporting Information), and it was shown that (a) ground electronic states of Cu_2_(OAc)_4_ and L–[Cu_2_(OAc)_4_] complexes, where L = MeCN and diazo **2**, are the antiferromagnetically coupled singlet states, while that of 2(MeCN)–[Cu_2_(OAc)_4_] is a triplet state, (b) molecule MeCN and diazo compound **2** (via its carbonyl oxygen) coordinate to the Cu(II)-center of Cu_2_(OAc)_4_ with 4.9 and 9.3 kcal/mol free energies, respectively. In other words, in the presence of MeCN, additional energy is required to displace MeCN and generate the **2**-Cu_2_(OAc)_4_ complex [or (Diazo)- Cu2(OAc)4], and (c) the following nitrogen extrusion requires a 24.4 kcal/mol free energy barrier and is 7.5 kcal/mol endergonic (with respect to the carbonyl oxygen coordinated diazo intermediate). The presented calculations show an insignificant (only 1.77 kcal/mol) coordination energy of the MeCN molecule to (MeCN)–[Cu_2_(OAc)_4_] at the axial position. Therefore, for simplicity, in our calculation of PES of the diazo decomposition by [Cu_2_(OAc)_4_], we assumed no axial ligand: we are confident that this assumption is not going impact to our general conclusion. Importantly, in the resultant (Carbene)–Cu_2_(OAc)_4_ complex, one of the Cu^II^-centers (which is coordinated to the carbene ligand) is partially removed from the dicopper tetracarboxylate framework, destabilizing the lantern structural motif of dicopper tetracarboxylate. As seen in Figure 2, the calculated Cu–carbene distance is 2.08 Å in (Carbene)–Cu_2_(OAc)_4_, and the Cu^1^–Cu^2^ and Cu^1^–O(carboxylate) distances are elongated from 2.51 and ∼1.97 Å in Cu_2_(OAc)_4_ to 2.69 and 2.23 Å in the Cu–carbene intermediate, respectively. Therefore, carboxylate ligands are likely to be labile once the copper carbene complex is formed, and this would destroy the C_4_ symmetric bowl-shape critical for asymmetric induction.

Another first-row metal with a history of being used in bimetallic carboxylate complexes is cobalt.^7a,7b,14^ Cobalt is a natural choice as an alternative to rhodium because it is directly above rhodium in the periodic table. The synthetic efforts to prepare Co_2_(*S*-TPPTTL)_4_ resulted in the formation of a magenta-colored material. This material was characterized through HR-MS, showing a Co_2_(*S*-TPPTTL)_4_^+^ ion, and IR spectroscopy, but it was not possible to confirm this assignment either through X-ray crystallography or other spectroscopic means. Regardless, the potential dicobalt complex was tested in the cyclopropanation of styrene using a donor–acceptor carbene. While this uncharacterized material was effective in decomposing the diazo compound at both room temperature and 40 °C to form cyclopropane **3** in good yield, the enantioselectivity was found to be very low (8 and 12%, respectively) (Table 2).

**Table 2:**
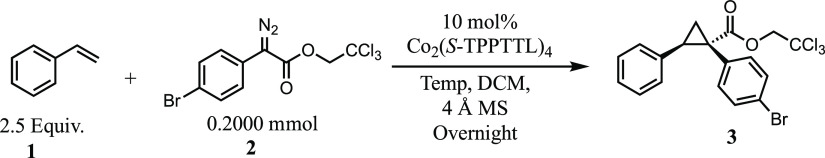
“Co_2_(*S*-TPPTTL)_4_”-Catalyzed Cyclopropanation

entry	catalyst	temp (°C)	yield (%)	ee (%)	d.r.
1	“Co_2_(*S*-TPPTTL)_4_”	25	73	8	>20:1
2	“Co_2_(*S*-TPPTTL)_4_”	40	66	12	>20:1

**Figure 3:**
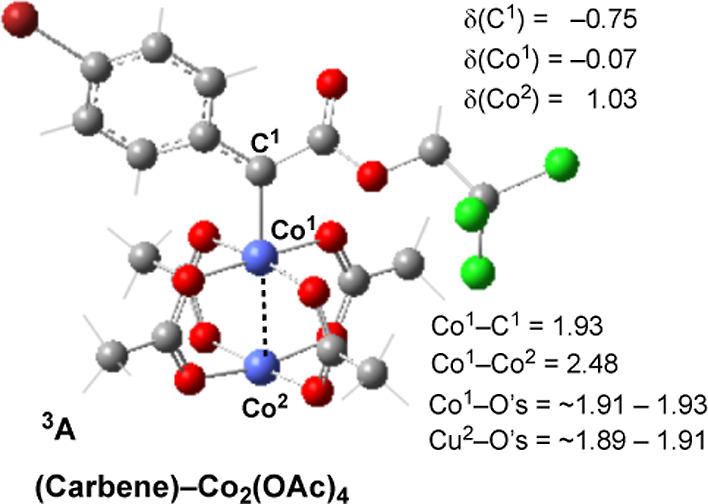
Important geometry (in Å) as well as unpaired spin populations, δ (in |e|), of the selected atoms of the Co_2_(OAc)_4_–carbene complex.

To gain additional insights into the above-presented experimental findings, we also computationally studied the nature of a proposed Co_2_–carbene intermediate. Close analyses of electronic properties of the (Carbene)–Co_2_(OAc)_4_ intermediate revealed that (a) the carbene carbon of this complex has a radical character with *ca* 0.75 |e| unpaired spin and (b) the Co-center that is bonded to the carbene has almost no unpaired spin (Figure 3). Noteworthily, the formation of the radical carbene is also expected for the 2(EtOH)–Co_2_(*esp*)_2_ with two high-spin Co^II^(d^7^) ions. When acting as a triplet carbene, the cobalt carbene has a very different reactivity profile from that of a singlet carbene, behaving more like a radical species.^15^ The cyclopropanation reaction would be a stepwise process forming diradical intermediates and unless the diradical combines immediately, limited asymmetric induction would be expected.

**Scheme 2:**
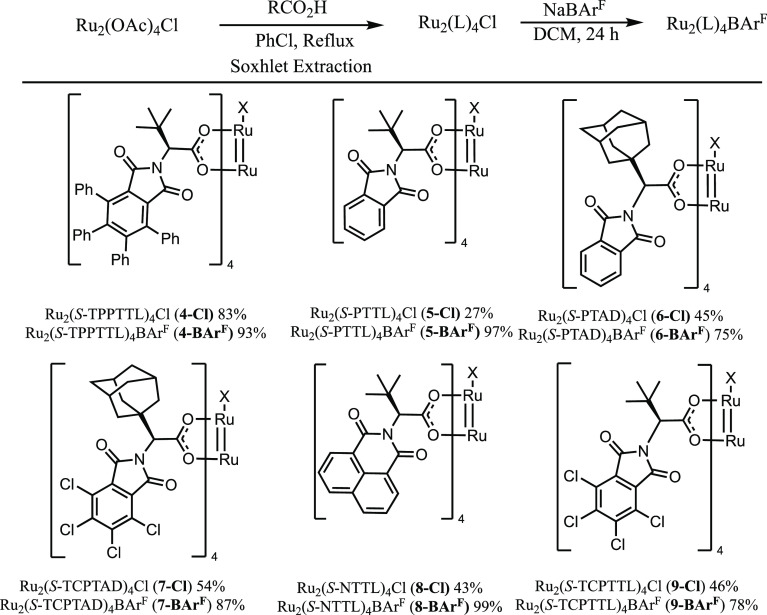
Synthesis of Diruthenium Complexes

Even though it would be desirable if a first-row transition-metal lantern complex could be developed as a replacement for dirhodium, the studies to date have shown that these complexes are either catalytically inactive or incapable of high asymmetric induction, presumably because the lantern structure is not formed or does not remain intact during the carbene reactions.^16^ We next tuned to diruthenium catalysts, which have been used for various carbene reactions.^7c,7d,10,17^ Inspired by the previous work of Miyazawa,^7c^ we decided to synthesize the diruthenium complex Ru_2_(*S*-TPPTTL)_4_Cl using the ligand exchange with Ru_2_(OAc)_4_Cl because the chiral diruthenium complex (Scheme 2), Ru_2_(*S*-TCPTTL)_4_•BAr^F^, had been shown to be capable of high asymmetric induction with acceptor/acceptor carbenes derived from iodonium ylides.^7c^

The ligand exchange was very effective, generating the desired complex, Ru_2_(*S*-TPPTTL)_4_Cl, in 83% yield. Due to the effectiveness of the ligand exchange, a series of related catalysts were prepared, Ru_2_(*S*-PTTL)_4_Cl, Ru_2_(*S*-PTAD)_4_Cl and Ru_2_(*S*-TCPTAD)_4_Cl, Ru_2_(*S*-NTTL)_4_Cl, and Ru_2_(*S*-TCPTTL)_4_Cl (the compound previously reported by Miyazawa). Extension of the ligand exchange to the more sterically crowded triarylcyclopropane carboxylate ligands^18^ was unsuccessful, and future work is needed to design conditions that can efficiently access these complexes. Definitive characterization of these complexes by NMR spectroscopy is not possible because of the paramagnetic nature of the diruthenium core. However, HRMS confirmed that the diruthenium structure was intact, and complete ligand exchange had occurred. Furthermore, suitable crystals were obtained to confirm by X-ray crystallography the bowl-shaped structure of the new catalysts. The cationic complexes of these catalysts were also generated by the reaction of the chloro-complexes with sodium tetrakis[3,5-bis(trifluoromethyl)phenyl]borate (NaBAr^F^).

**Figure 4:**
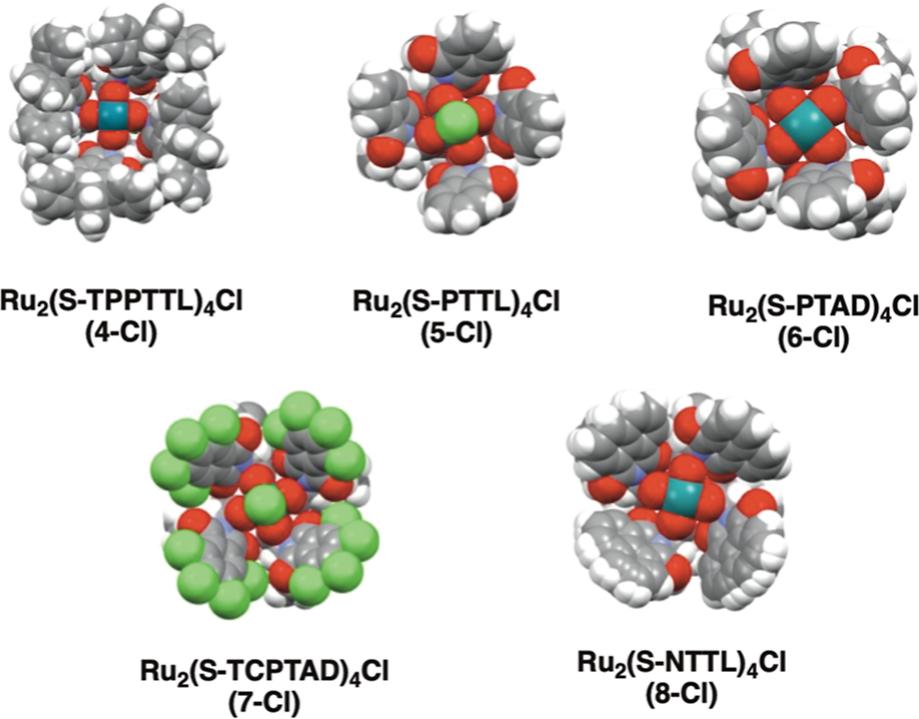
Crystal structures of the diruthenium catalyst (from top left: **4-Cl**, **5-Cl**, **6-Cl**, **7-Cl**, **8-Cl**). Axially coordinated solvent molecules are removed for clarity. Two of the naphthyl rings in **8-Cl** have conformational mobility and are disordered in the X-ray structure.

The crystal structures of the five new chloro-Ru(II,III) complexes are shown in Figure 4. All five complexes self-assemble to form the desired C_4_ symmetric bowl-shaped structures (other axial coordinating ligands and solvent molecules have been removed for clarity, see the Supporting Information for the complete structures). One interesting variation between the five structures is the location of the axially bound chlorine. In two of the catalysts, Ru_2_(*S*-PTTL)_4_Cl and Ru_2_(*S*-TCPTAD)_4_Cl, chlorine is located within the bowl and in Ru_2_(*S*-TPPTTL)_4_Cl, Ru_2_(*S*-PTAD)_4_Cl, and Ru_2_(*S*-NTTL)_4_Cl, it is located on the face outside of the bowl. If the chloride remained intact at the axial position during the catalyzed reaction, this would likely cause a very different catalytic behavior and asymmetric induction, dependent on the position of the chlorine because the axial site outside the bowl, adjacent to the *tert*-butyl or adamantyl groups, is sterically compromised and has a different chiral environment to the axial site within the bowl.^10b^ The crystal structure of Ru_2_(*S*-NTTL)_4_Cl has disorder in the location of two of the naphthylimido rings, indicating that the complex has some conformational mobility in the solid state.

**Figure 5:**
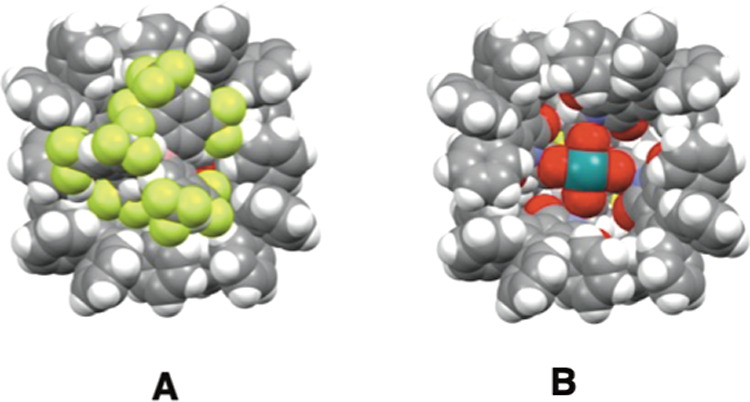
Crystal structures of diruthenium catalyst **4-BAr**^**F**^ (**A** with the BAr^F^ ligand and **B** with the BAr^F^ removed for clarity).

The crystal structure of the diruthenium catalyst **4-BAr**^**F**^ was also obtained as illustrated in Figure 5. An interesting feature of the solid-state structure is the location of the BAr^F^ ligand. Even though BAr^F^ is generally considered as a large, delocalized anion that is separated from the cationic counterion, in this case, it fits nicely on top of the catalyst bowl. However, as shown in Figure 5B, this does not alter the bowl-shape because the structure with the BAr^F^ removed for clarity (Figure 5B) looks very similar to **4-Cl** (Figure 4).

The diruthenium catalysts were examined in a standard cyclopropanation reaction between styrene (**1**) and diazo compound **2** (Table 3). All 12 catalysts effectively decomposed the diazo compound at room temperature with a catalyst loading of 1 mol %. The reactions are highly diastereoselective, and all the catalysts give reasonable levels of enantioselectivity. Ru_2_(*S*-TPPTTL)_4_·BAr^F^ (**4-BAr**^**F**^) is the best catalyst, forming cyclopropane **3** in 70% yield and 82% ee (entry 12). Interestingly, for all catalysts except for **4-BAr**^**F**^ and **9-BAr**^**F**^, the enantioselectivity decreased slightly when the BAr^F^ analogue was used as compared to the Cl derivatives. The two catalysts with the Cl ion coordinated to the front-face in the crystal structure, Ru_2_(*S*-PTTL)_4_Cl (**5-Cl**) and Ru_2_(*S*-TCPTAD)_4_Cl (**7-Cl**), gave similar levels of enantioselectivity to the BAr^F^ analogues, suggesting that the chloride dissociates prior to the cyclopropanation. The unsubstituted phthalimido derivatives, Ru_2_(*S*-PTTL)_4_Cl (**5-Cl**) and Ru_2_(*S*-PTAD)_4_Cl (**6-Cl**), give the opposite asymmetric induction to the other four catalysts. This type of behavior has been previously seen in the carbene chemistry of Rh_2_(*S*-PTAD)_4_.^3b^

**Table 3:**
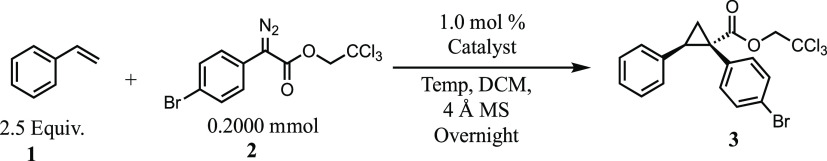
Diruthenium Catalyst Evaluation

entry	catalyst	yield (%)	ee (%)	d.r.
1	Ru_2_(*S*-TCPTTL)_4_Cl	71	60	>20:1
2	Ru_2_(*S*-TCPTTL)_4_BAr^F^	79	68	>20:1
3	Ru_2_(*S*-PTTL)_4_Cl	67	–60	>20:1
4	Ru_2_(*S*-PTTL)_4_ BAr^F^	67	–46	>20:1
5	Ru_2_(*S*-PTAD)_4_Cl	67	–61	>20:1
6	Ru_2_(*S*-PTAD)_4_ BAr^F^	57	–55	>20:1
7	Ru_2_(*S*-TCPTAD)_4_Cl	77	65	>20:1
8	Ru_2_(*S*-TCPTAD)_4_ BAr^F^	60	39	>20:1
9	Ru_2_(*S*-NTTL)_4_Cl	85	50	>20:1
10	Ru_2_(*S*-NTTL)_4_ BAr^F^	71	20	>20:1
11	Ru_2_(*S*-TPPTTL)_4_Cl	66	77	>20:1
12	Ru_2_(*S*-TPPTTL)_4_BAr^F^	70	82	>20:1

The level of asymmetric induction with the optimum catalyst, Ru_2_(S-TPPTTL)_4_·BAr^F^ (**4-BAr**^**F**^), was evaluated across a broad scope of substrates (Table 4). The reaction of **2** with a range of derivatives formed cyclopropanes **10–16** with moderate to high levels of enantioselectivity (76–92% ee). A range of functional groups on the para-position are well tolerated, with 4-methylstyrene as the best carbene trap giving **13** in 74% yield and 92% ee. Under these conditions, small terminal alkenes were particularly good substrates, generating cyclopropanes **17**–**19** in 80–88% yield and 92–94% ee. In virtually all of these reactions, the diastereoselectivity is very high (>20:1 d.r.), but in the case of the allyl-trimethylsilane, cyclopropane **19** was formed with only 11:1 d.r. The reactions with styrene and hexene were then examined with a range of aryldiazoacetate derivatives to form the cyclopropanes **22–33** and **34–45,** respectively.

**Table 4:**
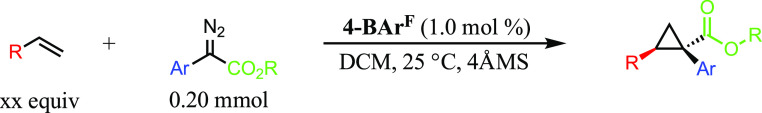

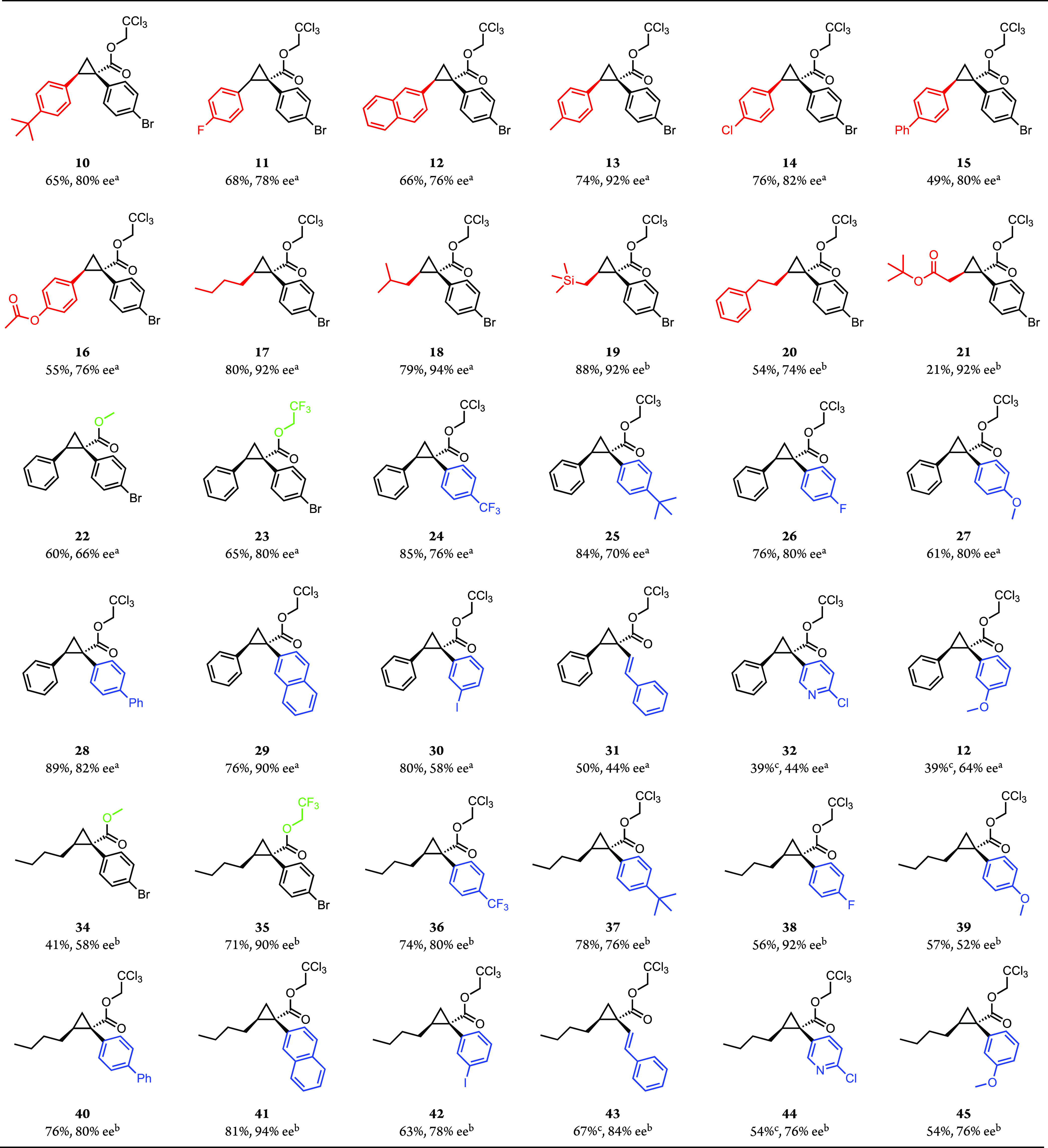
Ru_2_(*S*-TPPTTL)_4_BAr^F^-Catalyzed Cyclopropanation with Aryldiazoacetates^a^

a The scope of cyclopropanation reactions was studied at a 0.200 mmol scale using **4-BAr**^**F**^ at 1.0 mol % catalyst loading.

b Reactions with styrenes used 2.5 equiv of trap.

c Reactions with 1-hexene used 10 equiv of trap.

d The reaction was conducted at 40 °C. Compounds **10–21** illustrate the scope of alkene derivatives. Compounds **22–33** and **34–45** illustrate the scope of aryldiazoacetate with styrene and 1-hexene as the trap, respectively.

In the case of the ester functionality, a methyl ester results in lower levels of asymmetric inductions as seen with **22** (66% ee) and **34** (54% ee), whereas the trifluoroethyl derivatives **23** (80% ee) and **35** (90% ee) were formed with comparable levels of enantioselectivity to the trichloroethyl derivatives. A variety of aryldiazoacetates were examined and they all gave reasonable levels of asymmetric induction. Although there is some variability, in general, the cyclopropanation of 1-hexene gave higher levels of asymmetric induction than the cyclopropanation of styrene, with para-substituted aryldiazoacetates giving higher enantioselectivity than meta-substituted aryldiazoacetates. The most notable substrate is 2,2,2-trichloroethyl 2-diazo-2-(naphthalene-2-yl)acetate, which generated the cyclopropanes **29** and **41** in 90% ee and 94% ee, respectively. A 2-chloropyridyl heterocycle can also be incorporated in the diazo compound, resulting in the formation of **32** and **44**.

**Figure 6:**
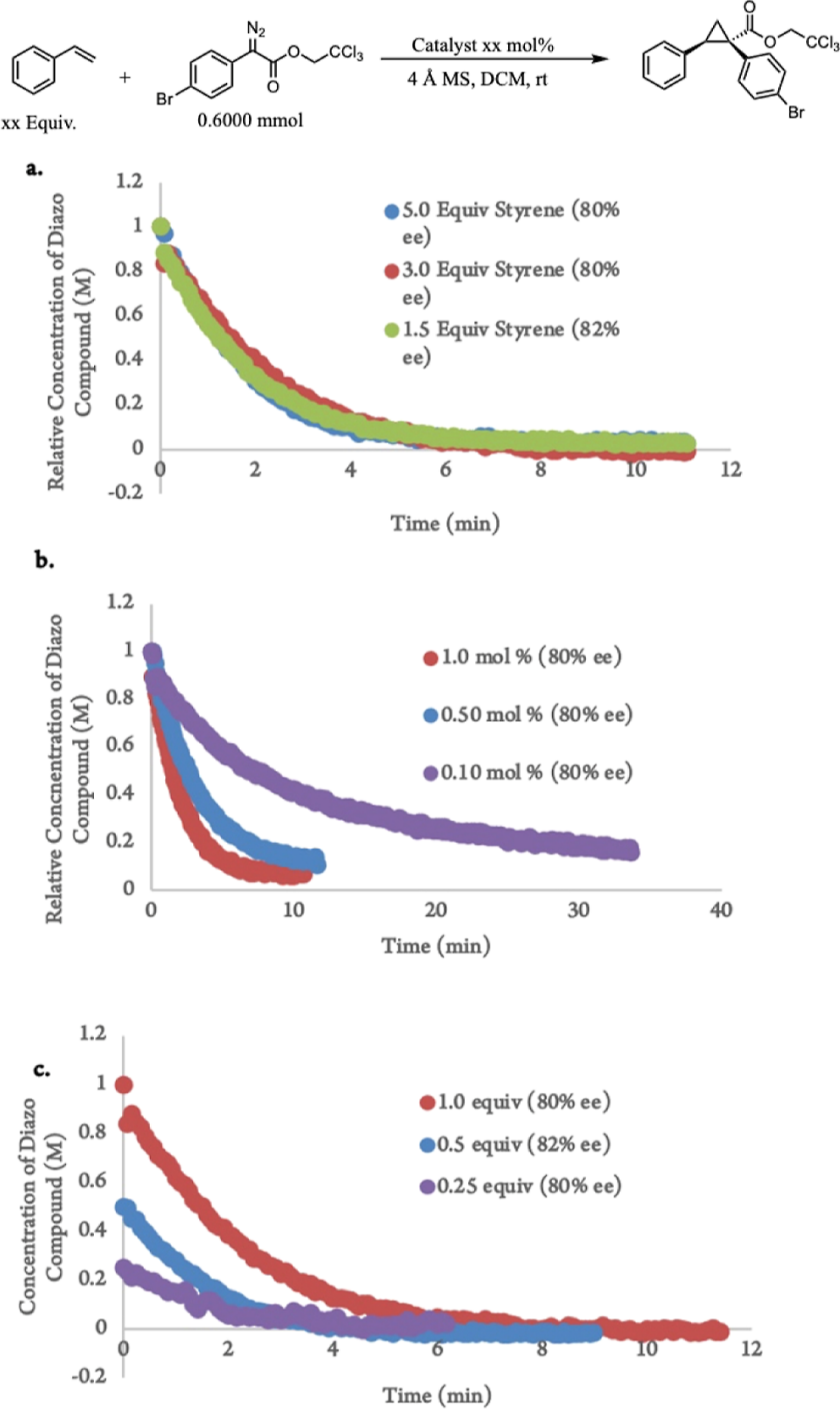
Kinetic profiles of reaction progress kinetic analysis studies for **4-BAr**^**F**^-catalyzed cyclopropanation of styrene. (a) Styrene concentration dependence. Experiments determined zero-order of the styrene trap ([diazo]_0_ = 0.05 M, at a 1.0 mol % catalyst loading. (b) Catalyst concentration dependence experiments. (c) Diazo concentration dependence. Experiments determined a positive order of diazo compound **2** ([styrene]_0_ = 0.15 M, at a 1.0 mol % catalyst loading).

With the scope of both substrate and diazo explored for Ru_2_(*S*-TPPTTL)4·BAr^F^, we turned next to studying the reaction kinetics through ReactIR. This is a convenient way to monitor the progress of reactions involving aryldiazoacetates through the distinct diazo stretch (∼2100 cm^–1^). The disappearance of this stretch is indicative of diazo consumption by the catalyst as has previously been demonstrated to positively correlate with the production of the carbene insertion product.^3b^ Previous studies have shown that styrene has an inhibitory effect on dirhodium catalysis due to π-coordination to the open face of the rhodium. We were concerned that this may be even more pronounced with the diruthenium catalysts because they are cationic species. Varying the amount of styrene in these reactions, however, showed that the diruthenium catalysts are not inhibited by styrene (Figure 6a). The reactions took approximately 10 min to fully consume the diazo compound at a 1 mol % catalyst loading, highlighting one of the differences between the ruthenium and rhodium catalysts, the latter of which is able to complete the reaction within 15 s. Next, the catalyst loading was varied to explore the competency of the diruthenium catalyst at low loadings (Figure 6b). A rate dependency on the catalyst was observed, with the reaction taking roughly 40 min to complete when the loading was lowered to 0.1 mol % compared with 10 min at 1.0 mol %. Finally, the rate dependency of diazo was explored (Figure 6c). The reaction showed a positive order with respect to the diazo concentration, in agreement with the computational results (see the Supporting Information for details).

Even though the diruthenium-catalyzed reactions are capable of similar levels of enantioselectivity to the dirhodium catalysts, two distinctive features of the catalysts are apparent. First, the asymmetric induction is not influenced by the location of the chloride in the solid-state structures. Second, the diruthenium catalysts are about 100 times slower than the dirhodium catalysts. Extended computational studies, at the [B3LYP-D3(BJ)] + PCM(in DCM) level of theory (for details, see above and the Supporting Information), were carried out on model systems [Ru_2_(OAc)_4_]Cl, [Ru_2_(OAc)_4_]^+^, and Rh_2_(OAc)_4_ as well as on the mechanisms of their reactions with diazo compound **2** to rationalize the observed experimental findings and elucidate the difference in reactivity and electronic properties of the diruthenium and dirhodium (tetracarboxylate) complexes.

**Figure 7:**
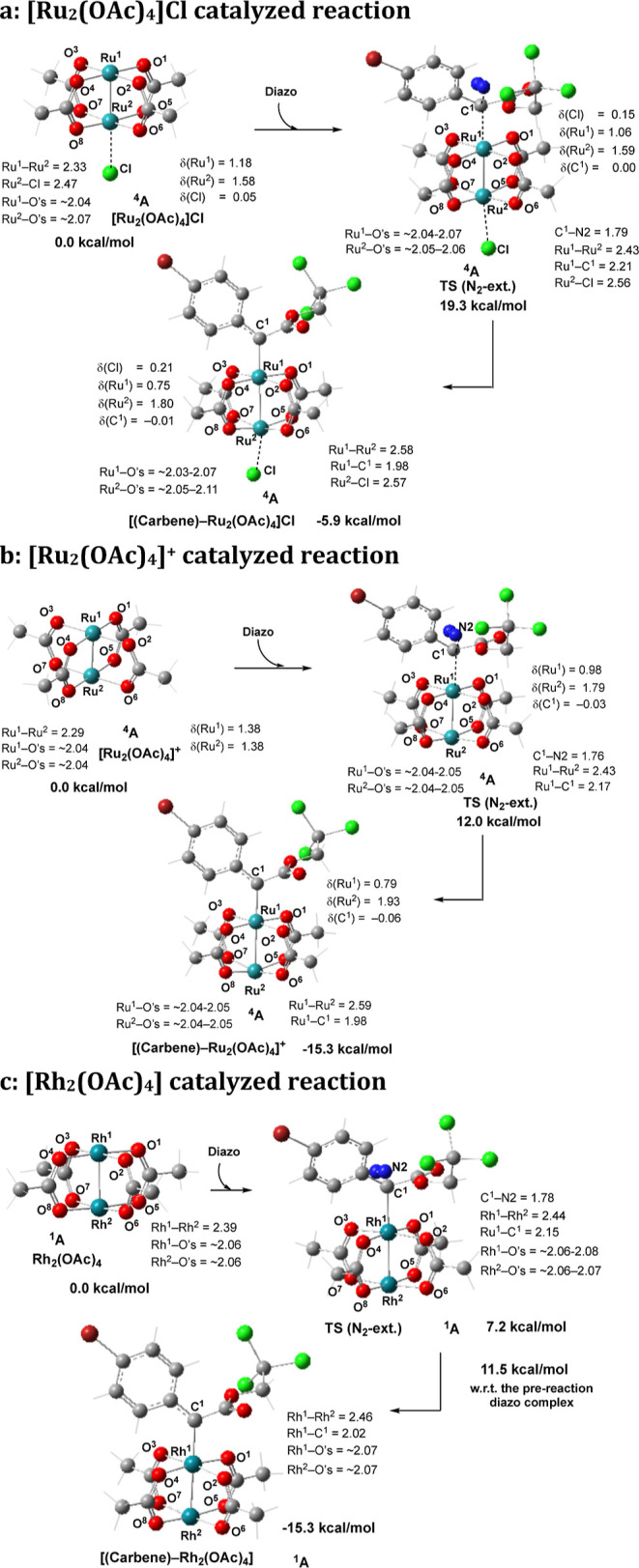
Calculated transition states and intermediates of the diazo decomposition by [Ru_2_(OAc)_4_]Cl, [Ru_2_(OAc)_4_]^+^, and [Rh_2_(OAc)_4_] complexes.

**Figure 8:**
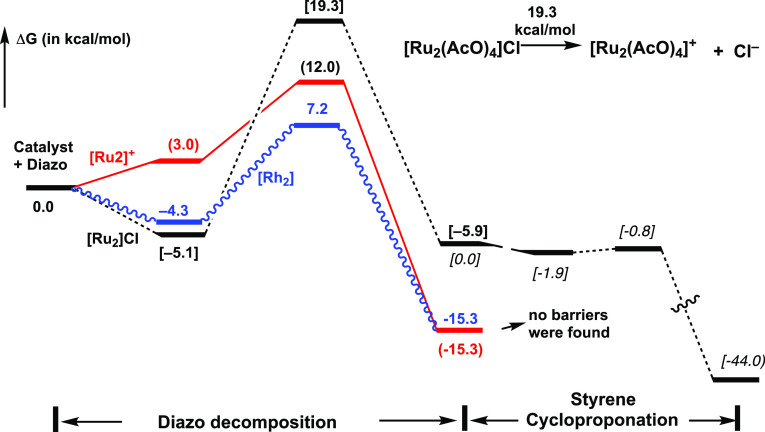
Schematic presentation of free energy surfaces of the diazo decomposition and styrene cyclopropanation reactions by [Ru_2_(OAc)_4_]Cl, [Ru_2_(OAc)_4_]^+^, and [Rh_2_(OAc)_4_] complexes.

In Figure 7, we show the nitrogen extrusion transition states and the resulting metal–carbene complexes of the reaction of these three systems with **2**. In Figure 8, we illustrate the free energy surfaces of these reactions. These calculations show that the ground electronic state of [Ru_2_(OAc)_4_]Cl is the quartet state with the low-spin Ru^II^/Ru^III^ core (see the Supporting Information for more details), where Ru^II^ and the Ru^III^-centers have unpaired spins of 1.18 and 1.58 |e|, respectively, with extensive delocalization of the spins due to Ru–Ru multiple bonding [electron configuration of (π*,δ*)^3^]. The antiferromagnetically coupled doublet electronic state of [Ru_2_(OAc)_4_]Cl is 10.4 kcal/mol higher in energy. The calculated Ru–Ru bond distances in the quartet and doublet state complexes are 2.325 and 2.407 Å, respectively. It has been previously shown that the lower-lying quartet and doublet states of (paddlewheel)–diruthenium complexes react similarly with the given substrate.^7d^ Therefore, for the sake of simplicity, here, we will discuss energy parameters only for the reaction of the ground quartet electronic state of [Ru_2_(OAc)_4_]Cl with **2**.

As seen in Figure 8, this reaction proceeds with 24.4 kcal/mol (or 19.3 kcal/mol) free energy barrier and is exergonic by only 0.8 kcal/mol (or 5.9 kcal/mol), calculated relative to the diazo-[Ru_2_(OAc)_4_]Cl intermediate (or to the reactants, i.e., [Ru_2_(OAc)_4_]Cl and **2**). To elucidate the impact of the axial chloride ligand on the reactivity of the chloro–diruthenium–tetracarboxylate catalysts, we studied the formation of the [Ru_2_(OAc)_4_]^+^ cationic complex from the [Ru_2_(OAc)_4_]Cl precursor as well as the electronic properties and reactivity of the resulting quartet state [Ru_2_(OAc)_4_]^+^ complex with **2**. Briefly, a comparison of the calculated data (see Figures 7a,b, and 8) for [Ru_2_(OAc)_4_]Cl and [Ru_2_(OAc)_4_]^+^ catalysts shows that (a) the dissociation of chloride from [Ru_2_(OAc)_4_]Cl to form an isolated Cl-anion and [Ru_2_(OAc)_4_]^+^ cation requires 19.3 kcal/mol free energy. However, it is conceivable to expect that in the above presented experiments with an explicit solvent, the generated Cl-anion and [Ru_2_(OAc)_4_]^+^ cation form an ion-pair complex [Ru_2_(OAc)_4_]^+^·(Cl^–^), similar to the reported diruthenium(tetracarboxylate)·BAr^F^ complexes. Such ion-pairing, i.e. the formation of [Ru_2_(OAc)_4_]^+^·(Cl^–^), is expected to reduce the energy required for conversion of [Ru_2_(OAc)_4_]Cl to its cationic analogue. Thus, the formation of [Ru_2_(OAc)_4_]^+^·(Cl^–^) from [Ru_2_(OAc)_4_]Cl will require less than 19.3 kcal/mol energy and (b) the Cl-ligand does not critically impact electronic and geometry properties of the Ru^II^/Ru^III^ cores. On the contrary, the lack of the Cl-ligand reduces the nitrogen extrusion barrier to 12.0 kcal/mol and makes the overall diazo decomposition reaction more exergonic (see Figure 8). Thus, comparison of the presented energy parameters in (a) and (b) and utilization of the turnover frequency-determining intermediate and transition state concept^19^ enable us to conclude that the rate-limiting step of the diazo decomposition by the chloro–diruthenium–tetracarboxylate precursor occurring via the “chloride dissociation then nitrogen extrusion” pathway is the chloride dissociation step, which requires <19 kcal/mol energy. Furthermore, the calculated chloride dissociation (<19 kcal/mol) and nitrogen extrusion (12.0 kcal/mol) barriers of the “chloride dissociation then nitrogen extrusion” pathway of the reaction of [Ru_2_(OAc)_4_]Cl with **2** are smaller than the 24.4 kcal/mol barrier required for the nitrogen extrusion directly by the [Ru_2_(OAc)_4_]Cl complex (i.e., without the Cl-dissociation). In other words, the decomposition of the diazo compound **2** on the chloro–diruthenium–tetracarboxylate catalysts, most likely, occurs via the “chloride dissociation then nitrogen extrusion” pathway with <19 kcal/mol free energy barrier.

The reported <19 kcal/mol free energy barrier for the diazo decomposition on [Ru_2_(OAc)_4_]Cl via the “chloride dissociation then nitrogen extrusion” pathway is larger than 11.5 kcal/mol required for the diazo decomposition on the dirhodium–(tetracarboxylate) complex Rh_2_(OAc)_4_. This finding indicates that the dirhodium complex would be more efficient for the diazo decomposition than its diruthenium analogue. When the kinetic studies are performed with the [Ru_2_(*S*-TPPTTL)_4_]·BAr^F^, a similar effect would be expected. Though the BAr^F^ counterion is large and diffuse, it does not fully dissociate from the diruthenium tetracarboxylate cation as is apparent in the crystal structure of the complex (Figure 5), instead preferring to occupy the catalyst bowl. As such, though dissociation of the BAr^F^ anion to generate the active diruthenium cationic catalyst is expected to be lower than that of chloride, it contributes to the slower reactivity of these complexes relative to the dirhodium analogues.

To summarize, the above-presented computational findings are consistent with the experiments indicating that (a) the Cl ion coordinated to the front-face in the catalyst crystal structure will dissociate during the reaction, leading to similar asymmetric induction regardless of the location of initial chloride coordination and (b) the diazo decomposition by the chloro–diruthenium–carboxylate catalysts is a slower process than that with the dirhodium catalysts.^6,19a,21^

The next step of the reaction, i.e., styrene cyclopropanation by metal–carbene complexes generated via diazo decomposition, has been previously established to be an energetically facile process^19a,20^ and is not the rate-limiting step of the reaction. Regardless, here, we also studied styrene cyclopropanation by the carbene intermediates Carbene–[Ru_2_(OAc)_4_]^+^, Carbene–[Ru_2_(OAc)_4_]Cl, and Carbene–[Rh_2_(OAc)_4_]. Consistent with the previously established findings, we found that these reactions are highly exergonic (31.9, 44.0, and 33.3 kcal/mol, respectively) and occur with no (or very small) energy barriers at the current level of calculations (see Figure 8).^22^

## Conclusions

These studies demonstrate that diruthenium tetracarboxylate catalysts are promising replacements for their dirhodium congeners in the reactions of donor/acceptor carbenes. After initial attempts to utilize first-row transition-metal lantern complexes (dicopper and dicobalt) failed to result in significant levels of asymmetric induction, this study converged on the use of diruthenium (II/III) tetracarboxylates as the most promising alternative to dirhodium. Five new chiral diruthenium complexes were synthesized, and the counterion effect was explored by replacing the axial Cl with the bulky counterion BAr^F^ for each compound. These complexes are air-stable and can easily be purified by column chromatography. After the initial evaluation of cyclopropanation involving donor/acceptor carbenes derived from aryl diazoacetates with this series of diruthenium complexes, Ru_2_(*S*-TPPTTL)_4_·BAr^F^ emerged as the most selective complex for this system. The scope of the reaction was then explored and high enantioselectivity was achieved with a diverse range of substrates, with comparable levels of asymmetric induction to the dirhodium system. The kinetics of the reaction were investigated, highlighting the differences between the diruthenium catalysts and the dirhodium congeners including slower reaction rates and no inhibition by π-coordination of alkene substrates. Finally, the presented computations showed that (a) the reasons behind the failure of the dicopper and dicobalt catalysts are (a1) the lability of the carboxylate ligands in the Cu–carbene intermediate, which destroys the C_4_ symmetric bowl-shape critical for asymmetric induction and (a2) the radical character of carbene in the cobalt–carbene intermediate, which limits asymmetric induction, and (b) the decomposition of the diazo compound on the chloro–diruthenium–tetracarboxylate catalysts occurs via the “chloride dissociation then nitrogen extrusion” pathway, with the chloride dissociation being a rate-limiting step, and is slower process than that with the dirhodium catalysts.

Further refinement of the diruthenium catalysts will be needed before they outperform the dirhodium catalysts in terms of overall efficiency. The reactions are currently limited to a catalyst loading of 0.1 mol %, whereas the dirhodium catalysts can operate effectively at a catalyst loading of 0.001 mol %.^3^ Future studies will be directed toward further enhancing the diruthenium catalysts and continuing the search for first-row transition-metal lantern complexes that have the correct properties to be effective chiral catalysts.

## Supporting Information Available

Complete experimental and computational procedures and compound characterization by experimental methods (PDF)
Cartesian coordinates of all calculated structures (PDF)
