# Supplementary material for: Diruthenium Tetracarboxylate-Catalyzed Enantioselective Cyclopropanation with Aryldiazoacetates
Source: Organometallics. 2023 Jun 30;42(15):2122–33. doi: 10.1021/acs.organomet.3c00268 (PMC10428512; doi:10.1021/acs.organomet.3c00268)
Supplement: Supplementary file 1 — om3c00268_si_001.pdf [file om3c00268_si_001.pdf]

Supporting Information

Diruthenium Tetracarboxylate-Catalyzed Enantioselective Cyclopropanation with Aryldiazoacetates.

Joshua K. Sailer,[1] Jack C. Sharland,[1] John Bacsa,[1] Caleb F. Harris,[2] John F. Berry,[2] Djamaladdin G. Musaev*,[1,3] and Huw M. L. Davies.*,[1]

[1]Department of Chemistry, Emory University, 1515 Dickey Drive, Atlanta, Georgia 30322, United States

[2]Department of Chemistry, University of Wisconsin, 1101 University Avenue, Madison, Wisconsin 53706, United States

[3]Cherry L. Emerson Center for Scientific Computation, Emory University, 1521 Dickey Drive, Atlanta, Georgia 30322, United States

Table of Contents

General Considerations **2**

Preparation of Known Compounds **2**

Preparation of Diazo Compounds **3**

Cyclopropanation Reactions **4**

Catalyst Synthesis **21**

HPLC Chromatographs **35**

NMR Spectra of Novel Compounds **80**

References **102**

Computational Details **102**

Cartesian Coordinates of all calculated structures **103**

CAUTION: Diazo compounds are high energy compounds and need to be treated with respect. Even though we experienced no energetic decomposition in this work, care should be taken in handling large quantities of diazo compounds. Large scale reactions should be conducted behind a blast shield. For a more complete analysis of the risks associated with diazo compounds see the recent review by Bull et. al.[1]

General Considerations

All experiments were carried out in flame-dried glassware under argon atmosphere unless otherwise stated. Flash column chromatography was performed on silica gel. Unless otherwise noted, all other reagents were obtained from commercial sources (Sigma Aldrich, Fisher, TCI Chemicals, AK Scientific, Combi Blocks, Oakwood Chemicals, Ambeed) and used as received without purification. 1H, 13C, and 19F NMR spectra were recorded at either 400 MHz (13C at 100 MHz) on Bruker 400 spectrometer or 600 MHz (13C at 151 MHz) on INOVA 600 or Bruker 600 spectrometer. NMR spectra were run in solutions of deuterated chloroform (CDCl3) with residual chloroform taken as an internal standard (7.26 ppm for 1H, and 77.16 ppm for 13C), and were reported in parts per million (ppm). The abbreviations for multiplicity are as follows: s = singlet, d = doublet, t = triplet, q = quartet, p = pentet, m = multiplet, dd = doublet of doublet, etc. Coupling constants (J values) are obtained from the spectra. Thin layer chromatography was performed on aluminum-back silica gel plates with UV light and cerium aluminum molybdate (CAM) stain to visualize. Mass spectra were taken on a Thermo Finnigan LTQ-FTMS spectrometer with APCI or ESI. Melting points (mp) were measured in open capillary tubes with a Mel-Temp Electrothermal melting points apparatus and are uncorrected. IR spectra were collected on a Nicolet iS10 FT-IR spectrometer from Thermo Scientific and reported in unit of cm-1. Enantiomeric excess (% ee) data were obtained on an Agilent 1100 HPLC or an Agilent 1290 Infinity UHPLC, eluting the purified products using a mixed solution of HPLC-grade 2-propanol (i-PrOH) and n-hexane.

Preparation of Known Compounds

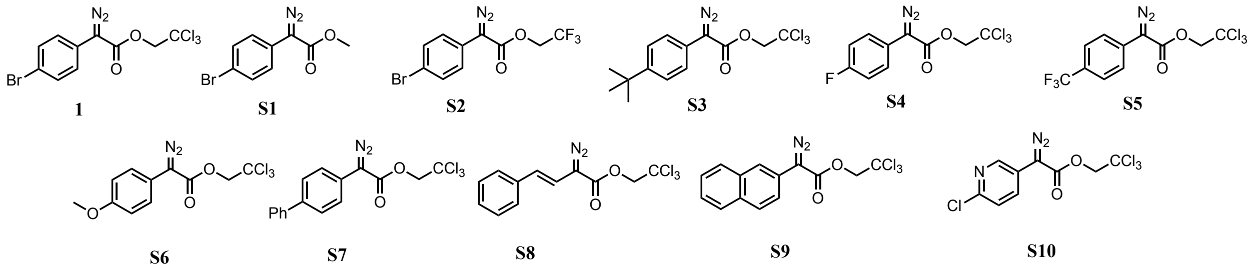

Figure S1: Known aryl-diazoacetate compounds.

Diazo compound **1**, **S3**, **S4**, **S5**, **S10** were prepared according to the established literature and matched the reported spectra.[2]

Diazo compound **S2**, **S6**, **S8**, and **S9** were prepared according to the established literature and matched the reported spectra.[3]

Diazo compound **S1** was prepared according to the established literature and matched the reported spectra.[4]
Diazo compound **S7** was prepared according to the established literature and matched the reported spectra.[5]

Preparation of carboxylic acid ligands:

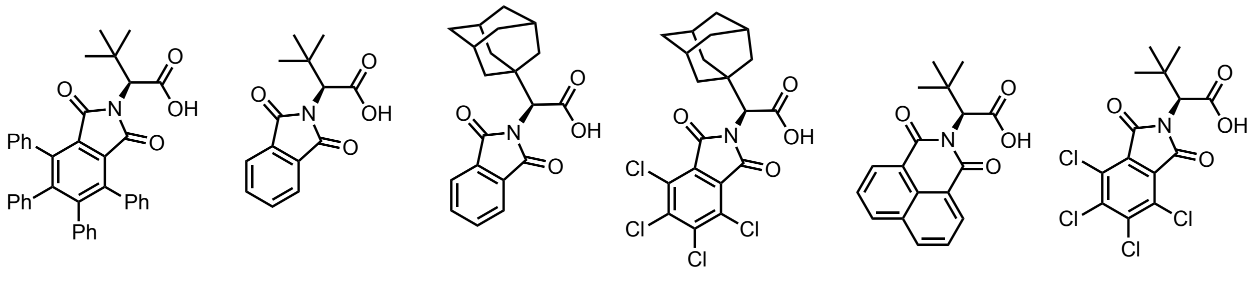

S12 **S13** **S14** **S15** **S16** **S17**

Figure S2: Known carboxylic acid ligands.

Ligand **S12, S13, S14, S15, S16,** and **S17** were prepared according to the established literature and matched the reported spectra.[6-11]

Preparation of known catalysts:

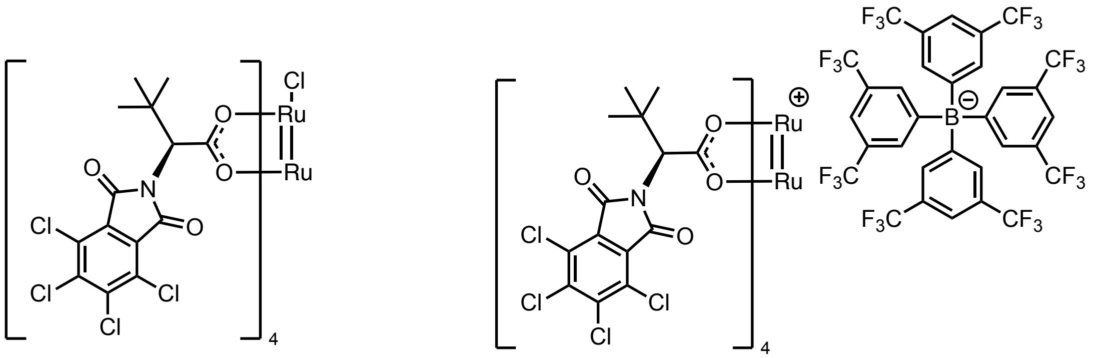

S18 **S19**

Figure S3: Known catalysts diruthenium catalyst.

Catalyst **S18** and **S19** were prepared according to the established literature and matched the reported spectra.[11]

Preparation of Diazo Compounds

2,2,2-Trichloroethyl 2-diazo-2-(3-iodophenyl)acetate (S11)

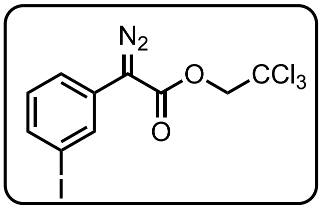

To a solution of 2-nitrobenzensulfonyl azide (2.6 g, 11 mmol, 1.5 equiv), and 2,2,2-trichloroethyl 2-(3-iodophenyl)acetate (3.0 g, 7.5 mmol, *1.0* equiv) dissolved in 50 mL of acetonitrile at 0 °C was added 2,3,4,5,6,7,8,9,10-octahydropyrido[1,2-a][1,3]diazepine (2.5 mL, 17 mmol, 2.2 equiv) was added slowly. Once addition was complete, the solution was allowed to stir for 1 h. Then, saturated NH_4Cl solution (50 mL) was added to the flask. The resulting solution was poured into a 250 mL separatory funnel and diluted with ether, washed with water (3x 20 mL) and brine (3x 15 mL) then dried over $MgSO_4$ and concentrated to afford a crude yellow solid. The crude material was purified through column chromatography (0% hexanes/diethyl ether, 0-2% hexanes/diethyl ether) to afford a yellow solid (2.26 g, 72%).
^{1}H NMR (600 MHz, $CDCl_3$): δ 7.91 (t, J=1.7 Hz, 1H), 7.57 (ddd, J = 7.9, 1.7, 1.0 Hz, 1H), 7.46 (m, H), 7.15 (t, J = 7.9 Hz, 1H), 4.94 (s, 2H);
^{13}C NMR (151 MHz, $CDCl_3$): δ 162.8, 135.2, 132.5, 130.5, 127.0, 123.0, 94.9, 73.9.
HMRS (+p APCI): calcd for $C_{10}H_7O_2{}^{35}Cl_3{}^{127}I$ [-N_2] 390.8551, found 390.8548.
IR (neat): 2094, 1710, 1585, 1554, 1475, 1373, 1343, 1273, 1238, 1140, 1087, 1043, 990, 934, 826, 777, 717, 702, 678, 577 (cm^{-1}).

Cyclopropanation Reactions

General Procedure 1

To a flame dried vial equipped with a stir bar and 4Å MS (100 weight%) under inert atmosphere was added catalyst (1 mol %) and substrate (0.5 mmol, 2.5 equiv) which was subsequently dissolved in 2 mL of DCM. Then, the diazo compound (0.2 mmol, 1.0 equiv) was dissolved in 2 mL of DCM and added to the reaction vial over a period of 2 h using a syringe pump. The reaction was run at room temperature for 18 h. Once completed the reaction solution was passed through a small silica plug to remove ruthenium catalyst, concentrated in vacuo, and purified through flash chromatography (0-18% hexanes/diethyl ether) to afford the desired product.

General Procedure 2

To a flame dried vial equipped with a stir bar and 4Å MS (100 weight%) under inert atmosphere was added catalyst (1 mol %) and substrate (2.0 mmol, 10 equiv) which was subsequently dissolved in 2 mL of DCM. Then, the diazo compound (0.2 mmol, 1.0 equiv) was dissolved in 2 mL of DCM and added to the reaction vial over a period of 2 h using a syringe pump. The reaction was run at room temperature for 18 h. Once completed the reaction solution was passed through a small silica plug to remove ruthenium catalyst, concentrated in vacuo, and purified through flash chromatography (0-18% hexanes/diethyl ether to afford the desired product.

2,2,2-Trichloroethyl (1*S*,2*R*)-1-(4-bromophenyl)-2-phenylcyclopropane-1-carboxylate (8)

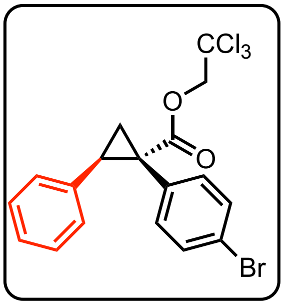

General procedure 1 was employed for the cyclopropanation of styrene (57.5 µL, 0.5 mmol, 2.5 equiv) with 2,2,2-trichloroethyl 2-(4-bromophenyl)-2-diazoacetate (0.2 mmol, 74.5 mg, 1.0 equiv) using Ru_2(*S*-TPPTTL)$_4$BAr^F (6.6 mg, 1 mol %) as catalyst. Purification by column chromatography afforded a crystalline solid (63 mg, 70%). Spectra matched literature precedent.[3]

^{1}H NMR (400 MHz, $CDCl_3$) δ 7.31 (d, J = 8.5 Hz, 2H), δ 7.15 (dd, J = 5.0, 1.9 Hz, 3H), δ 6.98 (d, J = 8.5 Hz, 2H), δ 6.85 (m, 2H), 4.88 (d, J = 11.9 Hz, 1H), δ 4.69 (d, J = 11.9 Hz, 1H), δ 3.27 (dd, J = 9.4, 7.5 Hz, 1H), δ 2.33 (dd, J = 9.4, 5.2 Hz, 1H), δ 2.02 (dd, J = 7.5, 5.2 Hz, 1H).

Chiral HPLC: The enantiopurity was determined to be 92:8 er by chiral HPLC analysis (Chiracel AD-H, 1.0% IPA/Hexanes, 1.0 mL/min, λ=230 nm, RT: Major: 6.7 min, Minor: 8.5 min).

2,2,2-Trichloroethyl (1*S*,2*R*)-1-(4-bromophenyl)-2-(4-(*tert*-butyl)phenyl)cyclopropane-1-carboxylate (9)

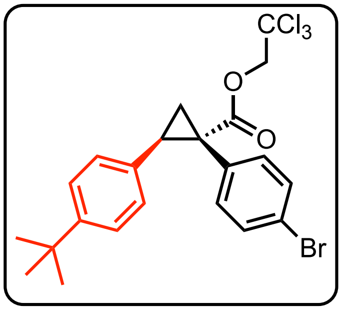

General procedure 1 was employed for the cyclopropanation of 1-(*tert*-butyl)-4-vinylbenzene (91.6 µL, 0.5 mmol, 91.6 µL, 2.5 equiv) with 2,2,2-trichloroethyl 2-(4-bromophenyl)-2-diazoacetate (0.2 mmol, 74.5 mg, 1.0 equiv) using Ru_2(*S*-TPPTTL)$_4$BAr^F (6.6 mg, 1 mol %) as catalyst. Purification by column chromatography afforded an oil (66 mg, 65%)

^{1}H NMR (600 MHz, $CDCl_3$) δ 7.27 (d, J = 8.5 Hz, 2H), δ 7.13 (d, J = 8.5 Hz, 2H), δ 6.96 (d, J = 8.5 Hz, 2H), δ 6.73 (d, J = 8.5 Hz, 2H), δ 4.84 (d, J = 11.9 Hz, 1H), δ 4.64 (d, J = 11.9 Hz, 1H), δ 3.18 (dd, J = 9.4, 7.5, 1H), δ 2.28 (dd, J = 9.4, 5.1 Hz, 1H), δ 1.93 (dd, J = 7.5, 5.1 Hz, 1H), δ 1.24 (s, 1H);

^{13}C NMR (151 MHz, $CDCl_3$) δ 171.7, 149.9, 133.8, 133.1, 132.2, 130.9, 127.8, 124.9, 121.5, 95.0, 74.4, 65.9, 36.5, 34.4, 33.8, 31.3, 20.5, 15.3.

HRMS (+p APCI) calcd for $C_{22}H_{23}BrCl_3O_2$ $(M+H)^+$ 502.9942 found 502.9945.

IR (neat): 2960, 2866, 1734, 1511, 1489, 1461, 1394, 1362, 1268, 1238, 1213, 1126, 1112, 1090, 1070, 1054, 1011, 972, 835, 805 (cm^{-1}).

Chiral HPLC: The enantiopurity was determined to be 90:10 er by chiral HPLC analysis (Chiracel OD-H, 1.0% IPA/Hexanes, 1.0 mL/min, λ=230 nm, RT: Major: 7.2 min, Minor: 4.7 min).

2,2,2-Trichloroethyl (1*S*,2*R*)-1-(4-bromophenyl)-2-(4-fluorophenyl)cyclopropane-1-carboxylate (10)

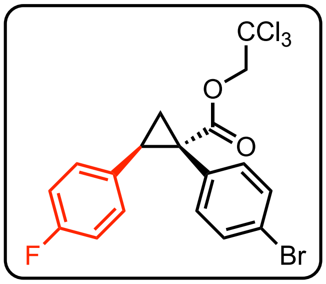

General procedure 1 was employed for the cyclopropanation of 1-fluoro-4-vinylbenzene (91.6 µL, 0.5 mmol, 2.5 equiv) with 2,2,2-trichloroethyl 2-(4-bromophenyl)-2-diazoacetate (0.2 mmol, 74.48 mg, 1.0 equiv) using Ru_2(*S*-TPPTTL)$_4$BAr^F (6.6 mg, 1 mol %) as catalyst. Purification by column chromatography afforded a crystalline solid (64 mg, 68%). Spectrum matched literature precedent.[3]

^{1}H NMR(400 MHz, $CDCl_3$) δ 7.28 (d, J = 8.4 Hz, 2H), δ 6.93 (d, J = 8.4 Hz, 2H), δ 6.78 (m, 4H), δ 4.83 (d, J = 11.9 Hz, 1H), δ 4.64 (d, J = 11.9 Hz, 1H), δ 3.20 (dd, J = 9.4, 7.4 Hz, 1H), δ 2.28 (dd, J = 9.4, 5.3 Hz, 1H), δ 1.92 (dd, J = 7.4, 5.3 Hz, 1H).
Chiral HPLC: The enantiopurity was determined to be 89:11 er by chiral HPLC analysis (Chiracel AD-H, 1.0% IPA/Hexanes, 1.0 mL/min, λ=230 nm, RT: Major: 6.9 min, Minor: 9.2 min).

2,2,2-Trichloroethyl (1*S*,2*R*)-1-(4-bromophenyl)-2-(naphthalen-2-yl)cyclopropane-1-carboxylate (11)

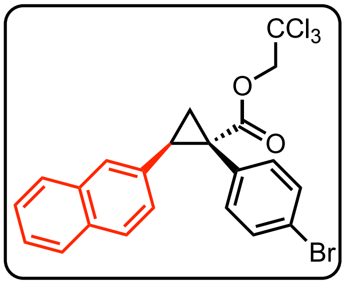

This structure was synthesized through general procedure 1. 2-Vinylnaphthalene (77.11 mg, 0.5 mmol, 2.5 equiv), 2,2,2-trichloroethyl 2-(4-bromophenyl)-2-diazoacetate (0.2 mmol, 74.5 mg, 1.0 equiv), and Ru_2(*S*-TPPTTL)$_4$BAr^F (6.6 mg, 1 mol %) were added to the reaction. The reaction was run overnight in 4 mL of DCM. Purification by column chromatography afforded a crystalline solid (66 mg, 66%). Spectra matched literature precedent.[12]
^{1}H NMR (400 MHz, $CDCl_3$) δ 7.75 (m, 1H), δ 7.68 (m, 1H), δ 7.60 (d, J = 8.5 Hz, 1H), δ 7.44 (m, 2H), δ 7.40 (m, 1H), δ 7.25 (d, J = 8.5 Hz, 2H), δ 7.02 (d, J = 8.5 Hz, 2H), δ 6.88 (dd, J = 8.5, 1.8 Hz, 1H), δ 4.89 (d, J = 11.9 Hz, 1H), δ 4.71 (d, J = 11.9 Hz, 1H), δ 3.43 (dd, J = 9.4, 7.4 Hz, 1H), δ 2.40 (dd, J = 9.4, 5.2, 1H), δ 2.14 (dd, J = 7.5, 5.2 Hz, 1H).
Chiral HPLC: The enantiopurity was determined to be 88:12 er by chiral HPLC analysis (Chiracel OD-H, 1.0% IPA/Hexanes, 1.0 mL/min, λ=230 nm, RT: Major: 13.0 min, Minor: 11.9 min).

2,2,2-Trichloroethyl (1*S*,2*R*)-1-(4-bromophenyl)-2-(*p*-tolyl)cyclopropane-1-carboxylate (12)

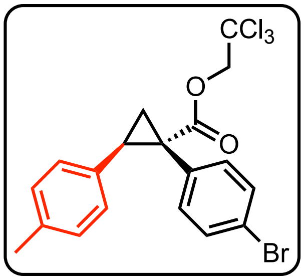

This structure was synthesized through general procedure 1. 1-Methyl-4-vinylbenzene (65.9 μL, 0.5 mmol, 2.5 equiv), 2,2,2-trichloroethyl 2-(4-bromophenyl)-2-diazoacetate (0.2 mmol, 74.5 mg, 1.0 equiv), and Ru_2(*S*-TPPTTL)$_4$BAr^F (6.6 mg, 1 mol %) were added to the reaction. The reaction was run overnight in 4 mL of DCM. Purification by column chromatography afforded a crystalline solid (69 mg, 75%):
MP: 95-98 °C
^{1}H NMR (600 MHz, $CDCl_3$) δ 7.30 (d, J = 8.5 Hz, 2H), δ 6.97 (d, J = 8.5 Hz, 2H), δ 6.94 (d, J = 8.2 Hz, 2H), δ 6.71 (d, J = 8.2 Hz, 2H), δ 4.85 (d, J = 11.9 Hz, 1H), δ 4.66 (d, J = 11.9 Hz, 1H), δ 3.21 (dd, J = 9.4, 7.45Hz, 1H), δ 2.29 (dd, J = 9.4, 5.1 Hz, 1H), δ 2.26 (s, 3H), δ 1.96 (dd, J = 7.5, 5.1 Hz, 1H).
^{13}C NMR (151 MHz, $CDCl_3$) δ 171.7, 136.5, 133.7. 133.1. 132.2, 130.9, 128.8, 121.5, 95.0, 74.4, 36.5, 33.9, 21.0, 20.3.
HRMS (+p APCI) calcd for $C_{19}H_{17}BrCl_3O_2$ (M+H) 460.9472 found 460.9471.
IR (neat): 3017, 2921, 1734, 1518, 1489, 1446, 1395, 1446, 1395, 1375, 1240, 1206, 1152, 1120, 1090, 1071, 1055, 1011, 969, 828 (cm^{-1}).
Chiral HPLC: Enantiopurity was determined to be 96:4 er by chiral HPLC analysis (Chiracel AD-H, 1.0% IPA/Hexanes, 1.0 mL/min, λ=230 nm, RT: Major: 6.9 min, Minor: 5.9 min)

2,2,2-Trichloroethyl (1*S*,2*R*)-1-(4-bromophenyl)-2-(4-chlorophenyl)cyclopropane-1-carboxylate (13)

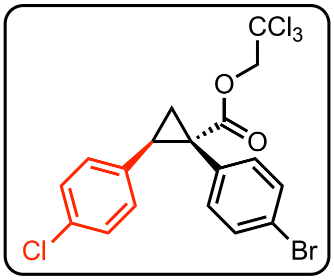

General procedure 1 was employed for the cyclopropanation of 1-methyl-4-vinylbenzene (60.0 μL, 0.5 mmol, 2.5 equiv) with 2,2,2-trichloroethyl 2-(4-bromophenyl)-2-diazoacetate (0.2 mmol, 74.5 mg, 1.0 equiv) using Ru_2(*S*-TPPTTL)$_4$BAr^F (6.6 mg, 1 mol %) as catalyst. Purification by column chromatography afforded a crystalline solid (73.6 mg, 76%). Spectra matched literature precedent.[3]

^{1}H NMR (400 MHz, $CDCl_3$) δ 7.29 (d, J = 8.5 Hz, 2H), δ 7.08 (d, J = 8.5 Hz, 2H), δ 6.93 (d, J = 8.5 Hz, 2H), δ 6.73 (d, J = 8.5 Hz, 2H), 4.82 (d, J = 11.9 Hz, 1H), δ 4.64 (d, J = 11.9 Hz, 1H), δ 3.18 (dd, J = 9.4, 7.4 Hz, 1H), δ 2.29 (dd, J = 9.4, 5.3 Hz, 1H), δ 1.92 (dd, J = 7.4, 5.3 Hz, 1H).

Chiral HPLC: Enantiopurity was determined to be 91:9 by chiral HPLC analysis (Chiracel AD-H, 1.0% IPA/Hexanes, 1.0 mL/min, λ=230 nm, RT: Major: 10.1 min, Minor: 7.5 min)

2,2,2-Trichloroethyl (1*S*,2*R*)-2-([1,1'-biphenyl]-4-yl)-1-(4-bromophenyl)cyclopropane-1-carboxylate (14)

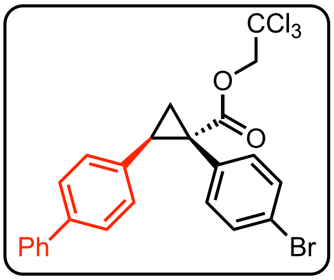

General procedure 1 was employed for the cyclopropanation of 4-vinyl-1,1'-biphenyl (0.5 mmol, 90.1 mg, 2.5 equiv) with 2,2,2-trichloroethyl 2-(4-bromophenyl)-2-diazoacetate (0.2 mmol, 74.5 mg, 1.0 equiv) using Ru_2(*S*-TPPTTL)$_4$BAr^F (6.6 mg, 1 mol %) as catalyst. Purification by column chromatography afforded a crystalline solid (49.3 mg, 47%):

MP: 145-147 °C

^{1}H NMR (600 MHz, $CDCl_3$) δ 7.53 (d, J = 8.3 Hz, 2H), δ 7.41 (dd, J = 8.5, 7.0 Hz, 2H), δ 7.37 (d, J = 8.3 Hz, 2H), δ 7.33 (m, 1H), δ 7.29 (d, J = 8.5, 2H), δ 6.99 (d, J = 8.5 Hz, 2H), δ 6.87 (d, J = 8.3 Hz, 2H) δ 4.85 (d, J = 11.9, 1H), δ 4.67 (d, J = 11.9 Hz, 1H), δ 3.26 (dd, J = 9.4, 7.5 Hz, 1H), δ 2.33 (dd, J = 9.4, 5.2 Hz, 1H), δ 2.00 (dd, 7.5, 5.2 Hz, 1H);

^{13}C NMR (151 MHz, $CDCl_3$) δ 171.6, 140.4, 139.6, 134.4, 133.7, 132.9, 131.1, 128.8, 127.3, 126.9, 126.7, 121.7, 94.9, 74.4, 36.8, 33.7, 20.6.

HRMS (+p APCI) calcd for $C_{24}H_{19}BrCl_3O_2$ (M+H) 522.9628 found 522.9631.

IR (neat): 3029, 1735, 1488, 1439, 1395, 1375, 1239, 1210, 1153, 1121, 1089, 1071, 1055, 1011, 972, 841, 826, 806 (cm^{-1}).

Chiral HPLC: Enantiopurity was determined to be 90:10 er by chiral HPLC analysis (Chiracel AD-H, 1.0% IPA/Hexanes, 1.0 mL/min, λ=254 nm, RT: Major: 8.5 min, Minor: 12.7 min).

2,2,2-Trichloroethyl (1*S*,2*R*)-2-(4-acetoxyphenyl)-1-(4-bromophenyl)cyclopropane-1-carboxylate (15)

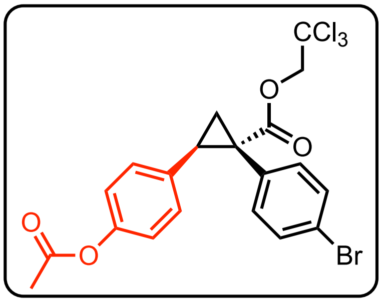

This structure was synthesized through general procedure 1. 4-vinylphenyl acetate (77.5 μL, 0.5 mmol, 2.5 equiv), 2,2,2-trichloroethyl 2-(4-bromophenyl)-2-diazoacetate (0.2 mmol, 74.5 mg, 1.0 equiv), and Ru_2(*S*-TPPTTL)$_4$BAr^F (6.6 mg, 1 mol %) were added to the reaction. The reaction was run overnight in 4 mL of DCM. Purification by column chromatography afforded a crystalline solid (55.7 mg, 55%). Spectra matched literature precedent.[3]

^{1}H NMR (400 MHz, $CDCl_3$) δ 7.07 (m, 3H), δ 6.98 (d, J = 8.1 Hz, 2H), δ 6.89 (d, J = 8.3 Hz, 2H), δ 6.83 (d, J = 8.3 Hz, 2H), δ 4.86 (d, J = 11.9 Hz, 1H), δ 4.67 (d, J = 11.9 Hz, 1H), δ 3.24 (dd, J = 9.4, 7.4 Hz, 1H), δ 1.95 (s, 3H), δ 1.95 (dd, J = 7.43, 5.29 Hz, 1H). Other cyclopropane proton signal falls under the methyl singlet at δ 1.95.

Chiral HPLC: Enantiopurity was determined to be 91:9 er by chiral HPLC analysis (Chiracel AD-H, 1.0% IPA/Hexanes, 1.0 mL/min, λ=230 nm, RT: Major: 15.4 min, Minor: 29.9 min).

2,2,2-Trichloroethyl (1*S*,2*S*)-1-(4-bromophenyl)-2-butylcyclopropane-1-carboxylate (16)

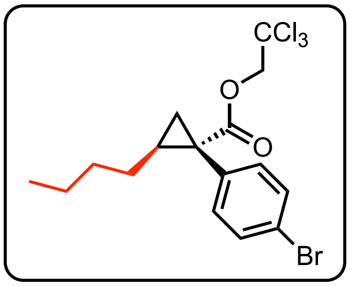

General procedure 2 was employed for the cyclopropanation of Hex-1-ene (250 μL, 2.0 mmol, 10 equiv) with 2,2,2-trichloroethyl 2-(4-bromophenyl)-2-diazoacetate (0.2 mmol, 74.5 mg, 1.0 equiv) using Ru_2(*S*-TPPTTL)$_4$BAr^F (6.6 mg, 1 mol %) as catalyst. Purification by column chromatography afforded an oil (68.4 mg, 80%). Spectra matched literature precedent.[13]

^{1}H NMR (400 MHz, $CDCl_3$): δ 7.48 (d, 8.4 Hz, 2H), δ 7.21 (d, 8.4 Hz, 2H), δ 4.81 (d, J = 12.0 Hz, 1H), δ 4.56 (d, J = 12.0 Hz, 1H), δ 1.95 (m, 1H), δ 1.88 (dd, J = 9.2, 4.2 Hz, 1H), δ 1.39 (m, 3H), δ 1.28 (m, 2H), δ 1.20 (dd, J = 6.9, 4.3 Hz, 1H), δ 0.85 (t, J = 7.3 Hz, 3H), δ 0.60 (ddd, J = 11.9, 9.7, 7.1 Hz, 1H).

Chiral HPLC: Enantiopurity was determined to be 96:3 er by chiral HPLC analysis (R,R-Whelk, 0.0% IPA/Hexanes, 1.0 mL/min, λ=230 nm, RT: Major: xx min, Minor: xx min).

2,2,2-Trichloroethyl (1*S*,2*S*)-1-(4-bromophenyl)-2-isobutylcyclopropane-1-carboxylate (17)

General procedure 2 was employed for the cyclopropanation of 4-methylpent-1-ene (257 μL, 2.0 mmol, 10 equiv) with 2,2,2-trichloroethyl 2-(4-bromophenyl)-2-diazoacetate (0.2 mmol, 74.5 mg, 1.0

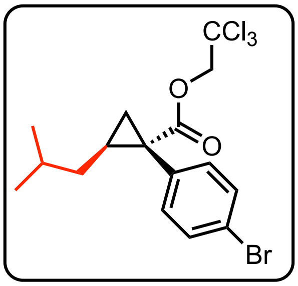

equiv) using Ru_2(*S*-TPPTTL)$_4$BAr^F (6.6 mg, 1 mol %) as catalyst. Purification by column chromatography afforded an oil (67.8 mg, 79%). Spectra matched literature precedent.[13]

^{1}H NMR (400 MHz, $CDCl_3$): δ 7.48 (d, J = 8.5 Hz, 2H), δ 7.20 (d, J = 8.5 Hz, 2H), δ 4.81 (d, J = 11.9 Hz, 1H), δ 4.6 (d, J = 11.9 Hz, 1H), δ 1.99 (m, 1H), δ 1.92 (dd, J = 9.2, 4.2 Hz, 1H), δ 1.70 (dq, J = 13.4, 6.7 Hz, 1H), δ 1.40 (ddd, 13.8, 6.5, 4.3 Hz, 1H), δ 1.22 (dd, 6.9, 4.2 Hz, 1H), δ 0.90 (dd, J = 6.7, 2.7 Hz, 6H), δ 0.36 (ddd, J = 13.8, 9.7, 7.2 Hz, 1H).

HPLC Chiral: Enantiopurity was determined to be 97:3 er by chiral HPLC analysis (R,R-Whelk, 0.0% IPA/Hexanes, 1.0 mL/min, λ=230 nm, RT: Major: 14.4 min, Minor: 27.4 min).

2,2,2-Trichloroethyl (1*S*,2*S*)-1-(4-bromophenyl)-2-((triMethylsilyl)Methyl)cyclopropane-1-carboxylate (18)

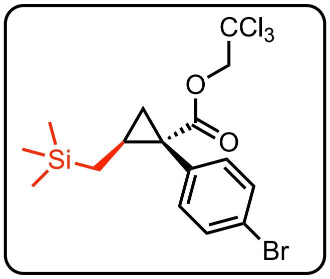

General procedure 2 was employed for the cyclopropanation of allyltrimethylsilane (318 μL, 2.0 mmol, 10 equiv) with 2,2,2-trichloroethyl 2-(4-bromophenyl)-2-diazoacetate (0.2 mmol, 74.5 mg, 1.0 equiv) using Ru_2(*S*-TPPTTL)$_4$BAr^F (6.6 mg, 1 mol %) as catalyst. Purification by column chromatography afforded an oil (71.6 mg, 88%). Spectra matched literature precedent.[13]

^{1}H NMR (400 MHz, $CDCl_3$) δ 7.49 (d, J = 8.4 Hz, 2H), δ 7.19 (d, J = 8.4 Hz, 2H), δ 4.80 (d, J = 11.9 Hz, 1H), δ 4.58 (d, J = 11.9 Hz, 1H), δ 1.97 (m, 2H), δ 1.12 (q, 3.3 Hz, 1H), δ 0.86 (ddd, J = 14.4, 2.7, 1.3 Hz, 1H), δ 0.04 (s, 9H), δ -0.44 (m, 1H).

Chiral HPLC: Enantiopurity was determined to be 96:4 er by chiral HPLC analysis (R,R-Whelk, 0.0% IPA/Hexanes, 1.0 mL/min, λ=230 nm, RT: Major: 14.0 min, Minor: 27.7 min).

2,2,2-Trichloroethyl (1*S*,2*S*)-1-(4-bromophenyl)-2-phenethylcyclopropane-1-carboxylate (19)

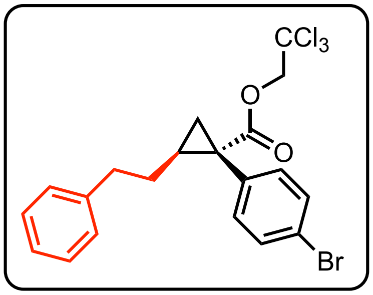

General procedure 2 was employed for the cyclopropanation of but-3-en-1-ylbenzene (300 μL, 2.0 mmol, 10 equiv) with 2,2,2-trichloroethyl 2-(4-bromophenyl)-2-diazoacetate (0.2 mmol, 74.5 mg, 1.0 equiv) using Ru_2(*S*-TPPTTL)$_4$BAr^F (6.6 mg, 1 mol %) as catalyst. Purification by column chromatography afforded an oil (51.6 mg, 60%). Spectra matched literature precedent.[13]

^{1}H NMR (400 MHz, $CDCl_3$): δ 7.48 (d, J = 8.5 Hz, 2H), δ 7.28 (m, 2H), δ 7.21 (d, J = 8.5 Hz, 3H), δ 7.11 (m, 2H), δ 4.81 (d, J = 11.9 Hz, 1H), δ 4.59 (d, J = 11.9 Hz, 1H), δ 2.71 (m, 2H), δ 2.03 (tdd, J = 9.0, 7.0, 5.2 Hz, 1H), δ 1.89 (ddd, J = 9.0, 4.5, 0.70 Hz, 1H), δ 1.70 (m, 1H), δ 1.21 (dd, J = 7.0, 4.5 Hz, 1H), δ 0.97 (dtd, J = 13.9, 9.0, 6.4 Hz, 1H).

Chiral HPLC The enantiopurity was determined to be 88:12 er by chiral HPLC analysis (Chiracel AD-H, 1.0% IPA/Hexanes, 1.0 mL/min, λ=230 nm, RT: Major: 6.6 min, Minor: 7.6 min).

2,2,2-Trichloroethyl (1*S*,2*R*)-1-(4-bromophenyl)-2-(2-(*tert*-butoxy)-2-oxoethyl)cyclopropane-1-carboxylate (20)

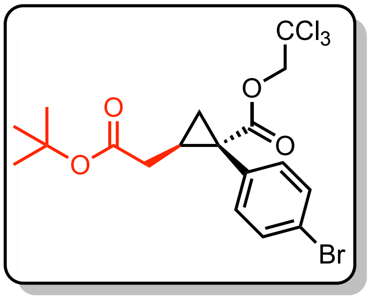

General procedure 2 was employed for the cyclopropanation of *tert*-butyl but-3-enoate (324 μL, 2.0 mmol, 10 equiv) with 2,2,2-trichloroethyl 2-(4-bromophenyl)-2-diazoacetate (0.2 mmol, 74.5 mg, 1.0 equiv) using Ru_2(*S*-TPPTTL)$_4$BAr^F (6.6 mg, 1 mol %) as catalyst. Purification by column chromatography afforded an oil (20.5 mg, 21%):

^{1}H NMR (400 MHz, $CDCl_3$) δ 7.46 (d, J = 8.4 Hz, 2H), 7.18 (d, J = 8.4 Hz, 2H), 4.80 (d, J = 11.9 Hz, 1H), 4.57 (d, J = 11.9 Hz, 1H), 2.31 (dq, J = 9.0, 7.0 Hz, 1H), 2.05-1.86 (m, 3H), 1.42 (s, 9H), 1.28 (dd, J = 7.0, 4.9 Hz, 1H).
^{13}C NMR (101 MHz, $CDCl_3$) δ 171.9, 170.9, 133.7, 133.1, 131.4, 121.9, 94.9, 80.9, 74.4, 36.1, 32.7, 28.1, 24.6, 20.6.
HRMS (+p APCI): calcd for $C_{18}H_{21}BrCl_3O_4$ [M+H] 484.9600 found 484.9497.
IR (neat) 2977, 1728, 1592, 1488, 1446, 1393, 1367, 1328, 1250, 1215, 1152, 1092, 1047, 1011, 945, 908, 852, 807 (cm^{-1}).
Chiral HPLC The enantiopurity was determined to be 96:4 er by chiral HPLC analysis (Chiracel AD-H, 1.0% IPA/Hexanes, 1.0 mL/min, λ=230 nm, RT: Major: 9.0 min, Minor: 9.6 min).

Methyl (1*S*,2*R*)-1-(4-bromophenyl)-2-phenylcyclopropane-1-carboxylate (21)

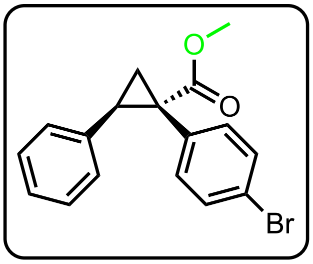

General procedure 1 was employed for the cyclopropanation of styrene (57.5 μL, 0.5 mmol, 2.5 equiv) with methyl 2-(4-bromophenyl)-2-diazoacetate (0.2 mmol, 51.0 mg, 1.0 equiv) using Ru_2(*S*-TPPTTL)$_4$BAr^F (6.6 mg, 1 mol %) as catalyst. Purification by column chromatography afforded a crystalline solid (39.6 mg, 60%). Spectra matched literature precedent.[4]

^{1}H NMR (400 MHz, $CDCl_3$) δ 7.29 (m, 2H), δ 7.12 (m, 3H), δ 6.92 (d, J = 8.0 Hz, 2H), δ 6.81 (dd, J = 6.5, 3.0 Hz, 2H), δ 3.69 (s, 3H), δ 3.15 (dd, J = 9.4, 7.3 Hz, 1H), δ 2.17 (dd, J = 9.4, 5.0 Hz, 1H), 1.87 (dd, J = 7.3, 5.0 Hz, 1H).
Chiral HPLC: The enantiopurity was determined to be 83:17 er by chiral HPLC analysis (Chiracel OD-H, 1.0% IPA/Hexanes, 0.5 mL/min, λ=230 nm, RT: Major: 15.7 min, Minor: 18.7 min).

2,2,2-Trifluoroethyl (1*S*,2*R*)-1-(4-bromophenyl)-2-phenylcyclopropane-1-carboxylate (22)

General procedure 1 was employed for the cyclopropanation of styrene (57.5 μL, 0.5 mmol, 2.5 equiv) with 2,2,2-trifluoroethyl 2-(4-bromophenyl)-2-diazoacetate (0.2 mmol, 64.6 mg, 1.0 equiv)

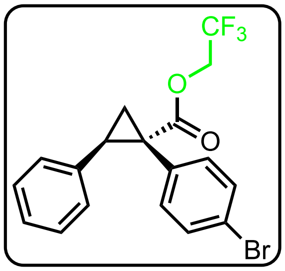

using Ru_2(*S*-TPPTTL)$_4$BAr^F (6.6 mg, 1 mol %) as catalyst. Purification by column chromatography afforded a crystalline solid (51.8 mg, 65%). Spectra matched literature precedent.[3]

^{1}H NMR (400 MHz, $CDCl_3$) δ 7.46 (d, J = 8.5 Hz, 2H), δ 7.14 (d, J = 8.5 Hz, 2H), δ 4.50 (dq, J = 12.7, 8.4 Hz, 1H), δ 4.32 (dq, J = 12.7, 8.4 Hz, 1H), δ 1.89 (tdd, J = 9.0, 6.8, 4.3 Hz, 1H), δ 1.80 (dd, J = 9.0, 4.3 Hz, 1H), δ 1.36 (m, 3H), δ 1.24 (m, 2H), δ 1.17 (dd, J = 6.8, 4.3 Hz, 1H), δ 0.83 (t, J = 7.2 Hz, 3H), δ 0.53 (m, 1H).

Chiral HPLC: The enantiopurity was determined to be 90:10 er by chiral HPLC analysis (Chiracel AD-H, 1.0% IPA/Hexanes, 1.0 mL/min, λ=230 nm, RT: Major: 5.8 min, Minor: 6.6 min).

2,2,2-Trichloroethyl (1*S*,2*R*)-2-phenyl-1-(4-(trifluoroMethyl)phenyl)cyclopropane-1-carboxylate (23)

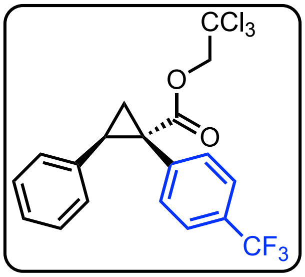

General procedure 1 was employed for the cyclopropanation of styrene (57.5 μL, 0.5 mmol, 2.5 equiv) with 2,2,2-trichloroethyl 2-diazo-2-(4-(trifluoromethyl)phenyl)acetate (0.2 mmol, 72.3 mg, 1.0 equiv) using Ru_2(*S*-TPPTTL)$_4$BAr^F (6.6 mg, 1 mol %) as catalyst. Purification by column chromatography afforded a crystalline solid (74.6 mg, 85%):

^{1}H NMR (400 MHz, $CDCl_3$) δ 7.43 (d, J = 8.0 Hz, 2H), 7.22 (d, J = 8.0 Hz, 2H), 7.15-7.11 (m, 3H), 6.83 (dd, J = 6.4, 3.1 Hz, 2H), 4.87 (d, J = 11.8 Hz, 1H), 4.69 (d, J = 11.8 Hz, 1H), 3.31 (dd, J = 9.4, 7.5 Hz, 1H), 2.37 (dd, J = 9.4, 5.2 Hz, 1H), 2.07 (dd, J = 7.5, 5.2 Hz, 1H).

^{13}C NMR (101 MHz, $CDCl_3$) δ 171.4, 137.9, 134.9, 132.34 128.1, 128.0, 127.0, 124.7, 94.9, 74.4, 36.9, 34.1, 20.1.

^{19}F NMR (376 MHz, $CDCl_3$) δ -62.54.

HRMS (+p APCI) calcd for $C_{19}H_{15}O_2Cl_3F_3$ (M+H) 437.0084 found 437.0084.

IR (neat) 3029, 1734, 1619, 1499, 1455, 1410, 1378, 1321, 1240, 1208, 1153, 1109, 1122, 1095, 1065, 1017, 971, 953, 910, 840, 809 (cm^{-1}).

Chiral HPLC: The enantiopurity was determined to be 88:12 er by chiral HPLC analysis (Chiracel AD-H, 1.0% IPA/Hexanes, 1.0 mL/min, λ=230 nm, RT: Major: 5.5 min, Minor: 7.2 min).

2,2,2-Trichloroethyl (1*S*,2*R*)-1-(4-(*tert*-butyl)phenyl)-2-phenylcyclopropane-1-carboxylate (24)

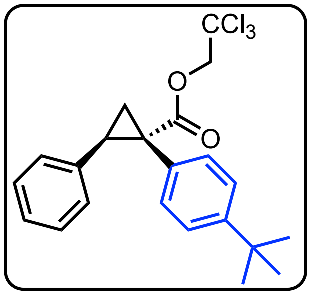

General procedure 1 was employed for the cyclopropanation of styrene (57.5 μL, 0.5 mmol, 2.5 equiv) with 2,2,2-trichloroethyl 2-(4-(tert-butyl)phenyl)-2-diazoacetate (0.2 mmol, 69.9 mg, 1.0 equiv) using Ru_2(*S*-TPPTTL)$_4$BAr^F (6.6 mg, 1 mol %) as catalyst. Purification by column chromatography afforded a crystalline solid (41.3 mg, 49%). Spectra matched literature precedent.[3]

^{1}H NMR (400 MHz, CDC$_3$) δ 7.17 (d, J = 8.4 Hz, 2H), δ 7.09 (m, 3H), δ 7.01 (d, J = 8.4 Hz, 2H), δ 6.81 (m, 2H), δ 4.86 (d, J = 11.9 Hz, 1H), δ

4.68 (d, J = 11.9 Hz, 1H), δ 3.22 (dd, J = 9.4, 7.4 Hz, 1H), δ 2.31 (dd, J = 7.4, 5.1 Hz, 1H), δ 2.00 (dd, J = 9.4, 5.1 Hz, 1H).
Chiral HPLC: The enantiopurity was determined to be 84:16 er by chiral HPLC analysis (Chiracel AD-H, 1.0% IPA/Hexanes, 0.5 mL/min, λ=230 nm, RT: Major: 8.5 min, Minor: 9.9 min).

2,2,2-Trichloroethyl (1*S*,2*R*)-1-(4-fluorophenyl)-2-phenylcyclopropane-1-carboxylate (25)

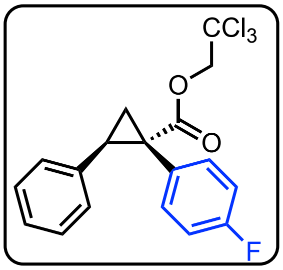

General procedure 1 was employed for the cyclopropanation of styrene (57.5 μL, 0.5 mmol, 2.5 equiv) with 2,2,2-trichloroethyl 2-diazo-2-(4-fluorophenyl)acetate (0.2 mmol, 62.3 mg, 1.0 equiv) using Ru_2(*S*-TPPTTL)$_4$BAr^F (6.6 mg, 1 mol %) as catalyst. Purification by column chromatography afforded a crystalline solid (35 mg, 45%). Spectra matched literature precedent.[3]
^{1}H NMR (400 MHz, $CDCl_3$) δ 7.13 (m, 3H), δ 7.06 (dd, J = 8.8, 5.3 2H), δ 6.83 (m, 4H), δ 4.83 (d, J = 11.9 Hz, 1H), δ 4.68 (d, J = 11.9 Hz, 1H), δ 3.24 (dd, J = 9.4, 7.4 Hz, 1H), δ 2.31 (dd, J = 9.4, 5.2 Hz, 1H), δ 2.00 (dd, J = 7.4, 5.2 Hz, 1H).
Chiral HPLC: The enantiopurity was determined to be 91:9 er by chiral HPLC analysis (Chiracel AD-H, 1.0% IPA/Hexanes, 1.0 mL/min, λ=230 nm, RT: Major: 6.0 min, Minor: 6.5 min).

2,2,2-Trichloroethyl (1*S*,2*R*)-1-(4-methoxyphenyl)-2-phenylcyclopropane-1-carboxylate (26)

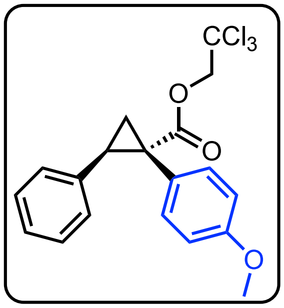

General procedure 1 was employed for the cyclopropanation of styrene (57.5 μL, 0.5 mmol, 2.5 equiv) with 2,2,2-trichloroethyl 2-diazo-2-(4-methoxyphenyl)acetate (0.2 mmol, 64.7 mg, 1.0 equiv) using Ru_2(*S*-TPPTTL)$_4$BAr^F (6.6 mg, 1 mol %) as catalyst. Purification by column chromatography afforded an oil (48.4 mg, 61%). Spectra matched literature precedent.[3]
^{1}H NMR (400 MHz, $CDCl_3$) δ 7.10 (dd, J = 5.2, 1.9 Hz, 3H), δ 6.99 (d, J = 8.2 Hz, 2H), δ 6.82 (m, 2H), δ 6.67 (d, J = 8.2 Hz, 2H), δ 4.85 (d, J = 11.9 Hz, 1H), δ 4.66 (d, J = 11.9 Hz, 1H), δ 3.72 (s, 3H), δ 3.20 (dd, J = 9.5, 7.4 Hz, 1H), δ 2.28 (dd, J = 9.5, 5.1 Hz, 1H), δ 1.97 (dd, J = 7.4, 5.1 Hz, 1H).
Chiral HPLC: The enantiopurity was determined to be 90:10 er by chiral HPLC analysis (Chiracel AD-H, 1.0% IPA/Hexanes, 1.0 mL/min, λ=254 nm, RT: Major: 8.3 min, Minor: 9.3 min).

2,2,2-Trichloroethyl (1*S*,2*R*)-1-([1,1'-biphenyl]-4-yl)-2-phenylcyclopropane-1-carboxylate (27)

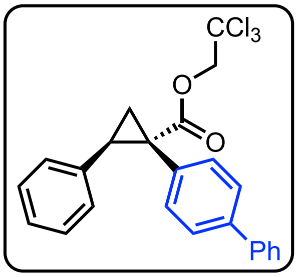

General procedure 1 was employed for the cyclopropanation of styrene (57.5 μL, 0.5 mmol, 2.5 equiv) with 2,2,2-trichloroethyl 2-([1,1'-biphenyl]-4-yl)-2-diazoacetate (0.2 mmol, 73.9 mg, 1.0 equiv) using Ru_2(*S*-TPPTTL)$_4$BAr^F (6.6 mg, 1 mol %) as catalyst. Purification by column chromatography afforded a crystalline solid (79.2 mg, 89%)
^{1}H NMR (400 MHz, CDCl$_3$) δ 7.53 (d, J = 7.0 Hz, 2H), 7.40 (m, 4H), 7.32 (m, 1H), 7.15 (d, J = 8.3 Hz, 2H), 7.09 (dd, J = 5.2, 1.9, 2H), 6.85 (m, 2H), 4.87 (d, J = 11.9, 1H), 4.68 (d, J = 11.9, 1H), 3.26 (dd, J = 9.4, 7.4, 1H), 2.33 (dd, J = 9.4, 5.1, 1H), 2.05 (dd, J = 7.4, 5.1, 1H).
^{13}C NMR (101 MHz, CDCl$_3$): δ 172.1, 140.7, 139.9, 135.7, 132.8, 132.4, 128.7, 128.2, 127.9, 127.3, 127.0, 126.7, 126.4 95.1, 74.4, 36.9, 34.0, 20.3.
IR (neat) 3029, 2952, 1731, 1600, 1487, 1448, 1432, 1377, 1332, 1238, 1208, 1150, 1094, 1051, 1008, 970, 911, 837, 806 (cm^{-1}).
HRMS (+p APCI) calcd for $C_{24}H_{20}O_2Cl_3$ (M+H) 445.0523 found 445.0518.
Chiral HPLC: The enantiopurity was determined to be 89:11 er by chiral HPLC analysis (Chiracel AD-H, 1.0% IPA/Hexanes, 1.0 mL/min, λ=230 nm, RT: Major: 7.9 min, Minor: 8.8 min).

2,2,2-Trichloroethyl (1*S*,2*R*)-1-(naphthalen-2-yl)-2-phenylcyclopropane-1-carboxylate (28)

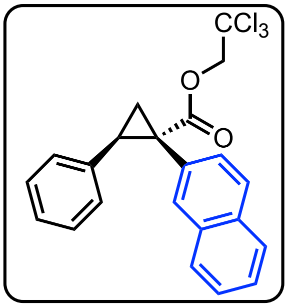

General procedure 1 was employed for the cyclopropanation of styrene (57.5 μL, 0.5 mmol, 2.5 equiv) with 2,2,2-trichloroethyl 2-diazo-2-(naphthalen-2-yl)acetate (0.2 mmol, 68.7 mg, 1.0 equiv) using Ru_2(*S*-TPPTTL)$_4$BAr^F (6.6 mg, 1 mol %) as catalyst. Purification by column chromatography afforded a crystalline solid (43.1 mg, 51%). Spectra matched literature precedent.[3]
^{1}H NMR (400 MHz, 400 CDCl$_3$) δ 7.75 (dt, J = 7.0, 3.9 Hz, 2H), δ 7.69 (s, 1H), δ 7.59 (d, J = 8.5 Hz, 1H), δ 7.46 (m, 2H), δ 7.15 (d, J = 8.5 Hz, 1H), δ 7.06 (m, 3H), δ 6.88 (m, 2H), δ 4.92 (d, J = 11.9 Hz, 1H), δ 4.67 (d, J = 11.9 Hz, 1H), δ 3.34 (dd, J = 9.5, 7.4 Hz, 1H), δ 2.41 (dd, J = 9.5, 5.1 Hz, 1H), δ 2.20 (dd, J = 7.4, 5.1 Hz, 1H).
Chiral HPLC: The enantiopurity was determined to be 94:6 er by chiral HPLC analysis (Chiracel AD-H, 1.0% IPA/Hexanes, 1.0 mL/min, λ=230 nm, RT: Major: 7.7 min, Minor: 8.9 min).

2,2,2-Trichloroethyl (1*S*,2*R*)-1-(3-iodophenyl)-2-phenylcyclopropane-1-carboxylate (29)

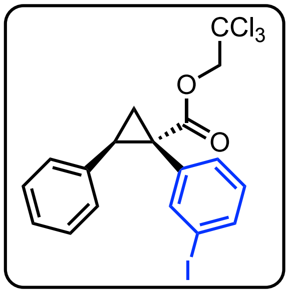

General procedure 1 was employed for the cyclopropanation of styrene (57.5 μL, 0.5 mmol, 2.5 equiv) with 2,2,2-trichloroethyl 2-diazo-2-(3-iodophenyl)acetate (0.2 mmol, 83.9 mg, 1.0 equiv) using Ru_2(*S*-TPPTTL)$_4$BAr^F (6.6 mg, 1 mol %) as catalyst. Purification by column chromatography afforded an oil (79.1 mg, 80%):

^{1}H NMR (400 MHz, CDCl$_3$) δ 7.46 (m, 2H), 7.12 (dd, J = 5.2, 2.0 Hz, 3H), 6.98 (dt, J = 5.2, 2.0 Hz, 1H), 6.83 (m, 3H), 4.85 (d, J = 11.9 Hz, 1H), 4.63 (d, J = 11.9 Hz, 1H), 3.22 (dd, J = 9.4, 7.5 Hz, 1H), 2.27 (dd, J = 9.4, 5.2 Hz, 1H), 2.00 (dd, J = 7.5, 5.2 Hz, 1H).

^{13}C NMR (101 MHz, CDCl$_3$): δ 171.5, 140.9, 136.4, 136.2, 135.1, 131.4, 129.9, 129.3, 128.1, 128.0, 94.9, 93.3, 74.5, 36.6, 34.0, 20.1.

HRMS (+p APCI) calcd for $C_{18}H_{15}O_2Cl_3I$ (M+H) 494.9177 found 494.9176.

IR (neat): 3028, 2951, 1732, 1591, 1560, 1497, 1474, 1454, 1432, 1412, 1376, 1328, 1264, 1240, 1208, 1152, 1103, 1051, 996, 972, 886, 814 (cm^{-1}).

Chiral HPLC: The enantiopurity was determined to be 72:27 er by chiral HPLC analysis (Chiracel OD-H, 0.5% IPA/Hexanes, 1.0 mL/min, λ=230 nm, RT: Major: 14.9 min, Minor: 13.7 min)

2,2,2-Trichloroethyl (1*R*,2*R*)-2-phenyl-1-((*E*)-styryl)cyclopropane-1-carboxylate (30)

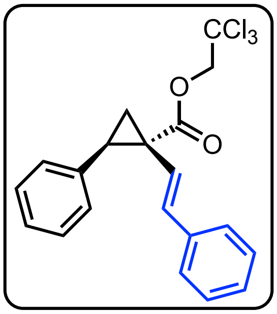

General procedure 1 was employed for the cyclopropanation of styrene (57.5 μL, 0.5 mmol, 2.5 equiv) with 2,2,2-trichloroethyl (*E*)-2-diazo-4-phenylbut-3-enoate (0.2 mmol, 63.9 mg, 1.0 equiv) using Ru_2(*S*-TPPTTL)$_4$BAr^F (6.6 mg, 1 mol %) as catalyst. Purification by column chromatography afforded a crystalline solid (47.5 mg, 50%). Spectra matched literature precedent.[3]

^{1}H NMR (400 MHz, CDCl$_3$) δ 7.29 (m, 2H), δ 7.25 (m, 2H), δ 7.22 (m, 1H), δ 7.20 (td, J = 6.7, 3.4 Hz, 4H), δ 4.91 (d, J = 11.9 Hz, 1H), δ 4.85 (d, J = 11.9 Hz, 1H), δ 3.21 (dd, J = 9.3, 7.4 Hz, 1H), δ 2.23 (dd, J = 9.3, 5.3 Hz, 1H), δ 1.97 (dd, J = 7.4, 5.3 Hz, 1H).

Chiral HPLC: The enantiopurity was determined to be 72:28 er by chiral HPLC analysis (Chiracel AD-H, 1.0% IPA/Hexanes, 1.0 mL/min, λ=230 nm, RT: Major: 6.8 min, Minor: 7.6 min).

2,2,2-Trichloroethyl (1*S*,2*R*)-1-(2-chloropyridin-4-yl)-2-phenylcyclopropane-1-carboxylate (31)

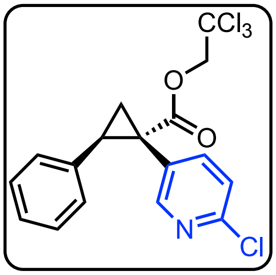

General procedure 1 was employed for the cyclopropanation of styrene (57.5 μL, 0.5 mmol, 2.5 equiv) with 2,2,2-trichloroethyl 2-(6-chloropyridin-3-yl)-2-diazoacetate (0.2 mmol, 65.8 mg, 1.0 equiv) using Ru_2(*S*-TPPTTL)$_4$BAr^F (6.6 mg, 1 mol %) as catalyst. Purification by column chromatography afforded a crystalline solid (31.3 mg, 39%). Spectra matched literature precedent.[3]

^{1}H NMR (400 MHz, $CDCl_3$) δ 8.16 (d, J = 2.5 Hz, 1H), δ 7.29 (m, 1H), δ 7.16 (dq, J = 4.7, 2.2 Hz, 3H), δ 7.08 (d, J = 8.0 Hz, 1H), 6.86 (dd, J = 7.4, 2.2 Hz, 2H), δ 4.86 (d, J = 11.9 Hz, 1H), δ 4.68 (d, J = 11.9 Hz, 1H), δ 3.30 (dd, J = 9.4, 7.5 Hz, 1H), δ 2.37 (dd, J = 9.4, 5.4 Hz, 1H), δ 2.07 (dd, J = 7.5, 5.4 Hz, 1H).

Chiral HPLC: The enantiopurity was determined to be 82:18 er by chiral HPLC analysis (Chiracel OD-H, 1.0% IPA/Hexanes, 1.0 mL/min, λ=230 nm, RT: Major: 14.8 min, Minor: 19.2 min).

2,2,2-Trichloroethyl (1*S*,2*R*)-1-(3-methoxyphenyl)-2-phenylcyclopropane-1-carboxylate (32)

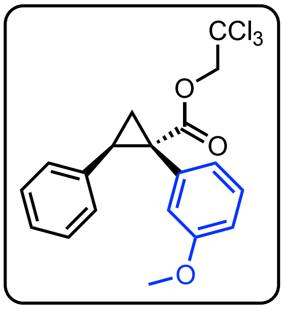

General procedure 1 was employed for the cyclopropanation of styrene (57.5 μL, 0.5 mmol, 2.5 equiv) with 2,2,2-trichloroethyl 2-diazo-2-(3-methoxyphenyl)acetate (0.2 mmol, 64.7 mg, 1.0 equiv) using Ru_2(*S*-TPPTTL)$_4$BAr^F (6.6 mg, 1 mol %) as catalyst. Purification by column chromatography afforded an oil (61.3 mg, 77%). Spectra matched literature precedent.[14]

^{1}H NMR (400 MHz, $CDCl_3$) δ 7.10 (dd, J = 5.1, 1.9 Hz, 3H), δ 7.05 (t, J = 7.9 Hz, 1H), δ 6.83 (m, 2H), δ 6.69 (t, 6.3 Hz, 2H), δ 6.56 (m, 1H), δ 4.87 (d, J = 11.8 Hz, 1H), δ 4.65 (d, J = 11.8 Hz, 1H), δ 3.59 (s, 3H), δ 3.21 (dd, J = 9.4, 7.5 Hz, 1H), δ 2.27 (d, J = 9.4, 5.2 Hz, 1H), δ 2.00 (dd, J = 7.5, 5.1 Hz, 1H).

Chiral HPLC: The enantiopurity was determined to be 79:21 er by chiral HPLC analysis (Chiracel AD-H, 1.0% IPA/Hexanes, 1.0 mL/min, λ=230 nm, RT: Major: 8.0 min, Minor: 7.1 min).

Methyl (1*S*,2*S*)-1-(4-bromophenyl)-2-butylcyclopropane-1-carboxylate (33)

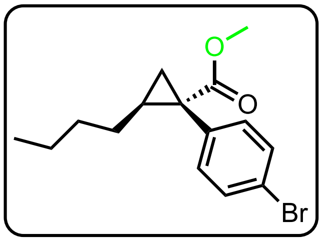

General procedure 2 was employed for the cyclopropanation of Hex-1-ene (250 μL, 2.0 mmol, 10 equiv) with methyl 2-(4-bromophenyl)-2-diazoacetate (0.2 mmol, 74.5 mg, 1.0 equiv) using Ru_2(*S*-TPPTTL)$_4$BAr^F (6.6 mg, 1 mol %) as catalyst. Purification by column chromatography afforded a clear colorless oil (51.0 mg, 41%).

^{1}H NMR (400 MHz, $CDCl_3$) δ 7.46 (d, J = 8.5 Hz, 2H), 7.16 (d, J = 8.5 Hz, 2H), 3.63 (s, 3H), 1.84 (tdd, J = 9.0, 6.7, 4.3 Hz, 1H), 1.73 (dd, J =

9.0, 4.1 Hz, 1H), 1.42-1.32 (m, 3H), 1.31-1.19 (m, 2H), 1.07 (dd, J = 6.7, 4.1 Hz, 1H), 0.84 (t, J = 7.2 Hz, 3H), 0.51 (ddd, J = 11.9, 9.7, 7.8 Hz, 1H).
^{13}C NMR (101 MHz, $CDCl_3$): δ 174.7, 135.5, 133.1, 132.1, 131.2, 121.1, 52.4, 33.1, 31.3, 30.0, 28.8, 22.4, 21.8, 14.0.
HRMS (+p APCI) calcd for $C_{15}H_{20}O_2Br$ (M+H) 311.0641 found 311.0638.
IR (neat): 2953, 2928, 2857, 1717, 1488, 1433, 1393, 1337, 1261, 1243, 1218, 1193, 1168, 1099, 1071, 1047, 1010, 961, 890, 872, 822 (cm^{-1}).
Chiral HPLC: The enantiopurity was determined to be 79:21 er by chiral HPLC analysis (S,S, Whelk, 1.0% IPA/Hexanes, 1.0 mL/min, λ=230 nm, RT: Major: 24.2 min, Minor: 14.7 min).

2,2,2-Trifluoroethyl (1*S*,2*S*)-1-(4-bromophenyl)-2-butylcyclopropane-1-carboxylate (34)

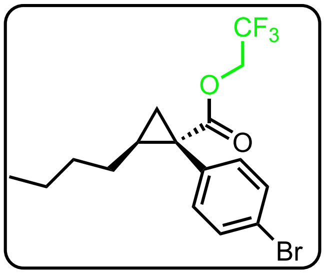

General procedure 2 was employed for the cyclopropanation of Hex-1-ene (250 μL, 2.0 mmol, 10 equiv) with 2,2,2-trifluoroethyl 2-(4-bromophenyl)-2-diazoacetate (0.2 mmol, 64.6 mg, 1.0 equiv) using Ru_2(*S*-TPPTTL)$_4$BAr^F (6.6 mg, 1 mol %) as catalyst. Purification by column chromatography afforded an oil (53.8 mg, 71%).
^{1}H NMR (400 MHz, $CDCl_3$) δ 7.46 (d, J = 8.5 Hz, 2H), 7.14 (d, J = 8.5 Hz, 2H), 4.50 (dq, J = 12.7, 8.4 Hz, 1H), 4.32 (dq, J = 12.7, 8.4 Hz, 1H), 1.89 (tdd, J = 9.0, 6.8, 4.3 Hz, 1H), 1.80 (dd, J = 9.0, 4.3 Hz, 1H), 1.42-1.31 (m, 3H), 1.29-1.20 (m, 2H), 1.17 (dd, J = 6.8, 4.3 Hz, 1H), 0.83 (t, J = 7.2 Hz, 3H), 0.53 (m, 1H).
^{13}C NMR (101 MHz, $CDCl_3$) δ 172.6, 134.3, 132.9, 131.3, 124.2, 121.5, 60.7 (q, J = 36.59), 32.8, 31.2, 29.9, 29.7, 22.4, 22.1, 13.9.
^{19}F NMR (376 MHz, $CDCl_3$) δ -73.93.
HRMS (+p APCI) calcd for $C_{16}H_{19}O_2BrF_3$ (M+H) 379.0515 found 379.0511.
IR (neat) 2958, 2929, 2859, 1736, 1539, 1489, 1440, 1395, 1283, 1258, 1238, 1159, 1099, 1074, 1011, 976, 821 (cm^{-1}).
Chiral HPLC: The enantiopurity was determined to be 95:5 er by chiral HPLC analysis (Chiracel OD-H, 1.0% IPA/Hexanes, 1.0 mL/min, λ=230 nm, RT: Major: 5.4 min, Minor: 4.3 min).

2,2,2-Trichloroethyl (1*S*,2*S*)-2-butyl-1-(4-(trifluoroMethyl)phenyl)cyclopropane-1-carboxylate (35)

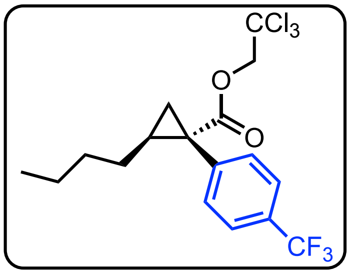

General procedure 2 was employed for the cyclopropanation of Hex-1-ene (250 μL, 2.0 mmol, 10 equiv) with 2,2,2-trichloroethyl 2-diazo-2-(4-(trifluoromethyl)phenyl)acetate (0.2 mmol, 72.3 mg, 1.0 equiv) using Ru_2(*S*-TPPTTL)$_4$BAr^F (6.648 mg, 1 mol %) as catalyst. Purification by column chromatography afforded a clear colorless oil (59.9 mg, 74%):
^{1}H NMR (400 MHz, $CDCl_3$): δ 7.62 (d, J = 8.0 Hz, 2H), 7.46 (d, J = 8.0 Hz, 2H), 4.82 (d, J = 11.9 Hz, 1H), 4.59 (d, J = 11.9 Hz, 1H), 2.00

(ddd, J = 9.0, 6.5, 4.2 Hz, 1H), 1.94 (dd, J = 9.0, 4.2 Hz, 1H), 1.47-1.35 (m, 3H), 1.27 (dt, J = 10.0, 6.2 Hz, 3H), 0.85 (t, J = 7.3 Hz, 3H), 0.56 (dt, J = 11.5, 7.3 Hz, 1H).
^{13}C NMR (101 MHz, $CDCl_3$): δ 172.3, 139.4, 131.8, 129.7, 129.4, 124.9 (q, J = 3.94 Hz) 122.8, 94.9, 74.3, 33.4, 31.2, 30.0, 22.4, 21.9, 13.9.
^{19}F NMR (376 MHz, $CDCl_3$) δ -62.49.
HRMS (+p APCI) calcd for $C_{17}H_{19}O_2Cl_3F_3$ (M+H) 417.0397 found 417.0392.
IR (neat): 2958, 2993, 2860, 1735, 1619, 1448, 1410, 1368, 1322, 1260, 1238, 1161, 1123, 1108, 1046, 1017, 975, 953, 888, 842, 809 (cm^{-1}).
Chiral HPLC: The enantiopurity was determined to be 90:10 er by chiral HPLC analysis (Chiracel AD-H, 1.0% IPA/Hexanes, 1.0 mL/min, λ=210 nm, RT: Major: 3.8 min, Minor: 4.3 min).

2,2,2-Trichloroethyl (1*S*,2*S*)-2-butyl-1-(4-(*tert*-butyl)phenyl)cyclopropane-1-carboxylate (36)

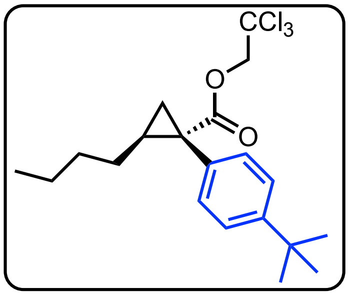

General procedure 2 was employed for the cyclopropanation of Hex-1-ene (250 µL, 2.0 mmol, 10 equiv) with 2,2,2-trichloroethyl 2-(4-(tert-butyl)phenyl)-2-diazoacetate (0.2 mmol, 69.9 mg, 1.0 equiv) using Ru_2(*S*-TPPTTL)$_4$BAr^F (6.6 mg, 1 mol %) as catalyst. Purification by column chromatography afforded a clear colorless oil (63.3 mg, 87%).
^{1}H NMR (400 MHz, $CDCl_3$) δ 7.34 (d, J = 8.4 Hz, 2H), 7.23 (d, J = 8.4 Hz, 2H), 4.79 (d, J = 12.0 Hz, 1H), 4.56 (d, J = 12.0 Hz, 1H), 1.93 (tdd, J = 8.9, 6.7, 4.5 Hz, 1H), 1.85 (dd, J = 9.1, 4.1 Hz, 1H), 1.47-1.35 (m, 3H), 1.32 (s, 9H), 1.21 (dd, J = 6.7, 4.1 Hz, 1H), 0.83 (J = 7.2 Hz, 3H), 0.71-0.59 (m, 1H).
^{13}C NMR (101 MHZ, $CDCl_3$) δ 173.2, 150.1, 132.1, 131.0, 124.9, 95.2, 74.2, 34.5, 33.1, 31.3, 29.9, 29.5, 22.4, 21.7, 14.0.
HRMS (+p ACPI) calcd for $C_{20}H_{28}O_2Cl_3$ (M+H) 405.1149 found 405.1151.
IR (neat) 2956, 2930, 2861, 1734, 1514, 1460, 1392, 1363, 1258, 1237, 1163, 1111, 1045, 1016, 969, 889, 833, 806 (cm^{-1}).
Chiral HPLC: The enantiopurity was determined to be 88:12 er by chiral HPLC analysis (Chiracel AD-H, 0.5% IPA/Hexanes, 1.0 mL/min, λ=210 nm, RT: Major: 7.1 min, Minor: 7.6 min).

2,2,2-Trichloroethyl (1*S*,2*S*)-2-butyl-1-(4-fluorophenyl)cyclopropane-1-carboxylate (37)

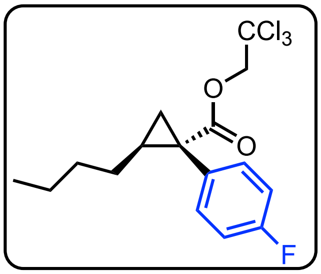

General procedure 2 was employed for the cyclopropanation of Hex-1-ene (250 µL, 2.0 mmol, 10 equiv) with 2,2,2-trichloroethyl 2-diazo-2-(4-fluorophenyl)acetate (0.2 mmol, 62.3 mg, 1.0 equiv) using Ru_2(*S*-TPPTTL)$_4$BAr^F (6.6 mg, 1 mol %) as catalyst. Purification by column chromatography afforded an oil (40.9 mg, 56%).
^{1}H NMR (400 MHz, $CDCl_3$) δ 7.35-7.25 (m, 2H), 7.04 (t, J = 8.7 Hz, 2H), 4.80 (d, J = 11.9 H, 1H), 4.58 (d, J = 11.9 Hz, 1H), 1.95 (tdd, J = 11.0, 4.8, 3.2 Hz, 1H), 1.88 (dd, J = 9.0, 4.0 Hz, 1H), 1.48-1.33 (m, 3H), 1.33-1.23 (m, 2H), 1.21 (dd, J = 6.7, 4.0 Hz, 1H), 0.85 (t, J = 7.2 Hz, 3H), 0.67-0.56 (m, 1H).

^{13}C NMR (101 MHz, CDCl$_3$) δ 172.9, 163.3, 160.8, 133.0, 131.1, 115.0, 114.8, 95.1, 74.3, 32.8, 31.2, 29.96 29.6, 22.4, 22.1, 14.0.
^{19}F NMR (376 MHz, CDCl$_3$) δ -115.05.
HRMS (+p APCI): calcd for $C_{16}H_{19}O_2Cl_3F$ (M+H) 367.0429 found 367.0430.
IR (neat) 2955, 2928, 2858, 1732, 1605, 1513, 1439, 1367, 1311, 1258, 1235, 1159, 1103, 1046, 1014, 970, 908, 889, 838, 806 (cm^{-1}).
Chiral HPLC: The enantiopurity was determined to be 96:4 er by chiral HPLC analysis (Chiracel AD-H, 1.0% IPA/Hexanes, 1.0 mL/min, λ=210 nm, RT: Major: 4.1 min, Minor: 4.5 min).

2,2,2-Trichloroethyl (1*S*,2*S*)-2-butyl-1-(4-methoxyphenyl)cyclopropane-1-carboxylate (38)

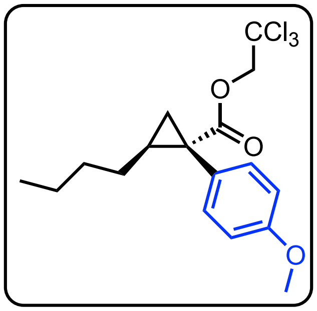

General procedure 2 was employed for the cyclopropanation of Hex-1-ene (250 μL, 2.0 mmol, 10 equiv) with 2,2,2-trichloroethyl 2-diazo-2-(3-methoxyphenyl)acetate (0.2 mmol, 64.7 mg, 1.0 equiv) using Ru_2(*S*-TPPTTL)$_4$BAr^F (6.6 mg, 1 mol %) as catalyst. Purification by column chromatography afforded an oil (43.5 mg, 57%)
^{1}H NMR (400 MHz, CDCl$_3$) δ 7.22 (d, J = 8.7 Hz, 2H), 6.86 (d, J = 8.7 Hz, 2H), 4.79 (d, J = 12.0 Hz, 1H), 4.55 (d, J = 12.0 Hz, 1H) 3.81 (s, 3H), 1.95-1.86 (m, 1H), 1.83 (dd, J = 9.1, 3.9 Hz, 1H), 1.44-1.32 (m, 3H), 1.25 (pd, J = 7.8, 3.2 Hz, 2H), 1.17 (dd, J = 6.7, 4.0 Hz, 1H), 0.83 (t, J = 7.3 Hz, 3H), 0.67-0.56 (m, 1H).
^{13}C NMR (101 MHz, CDCl$_3$) δ 173.4, 158.7, 132.5, 127.4, 113.4, 95.2, 74.2, 55.2, 32.8, 31.3, 29.9, 29.6, 22.4, 22.0, 14.0.
HRMS (+p APCI) calcdd for $C_{17}H_{21}Cl_3O_3$ (M+H) 379.0629 found 379.0629.
IR (neat) 2954, 2929, 2857, 1731, 1611, 1581, 1514, 1455, 1367, 1332, 1292, 1246, 1159, 1102, 1035, 969, 888, 833 (cm^{-1}).
Chiral HPLC The enantiopurity was determined to be 76:24 er by chiral HPLC analysis (Chiracel AD-H, 1.0% IPA/Hexanes, 1.0 mL/min, λ=280 nm, RT: Major: 5.3 min, Minor: 5.9 min).

2,2,2-Trichloroethyl (1*S*,2*S*)-1-([1,1'-biphenyl]-4-yl)-2-butylcyclopropane-1-carboxylate (39)

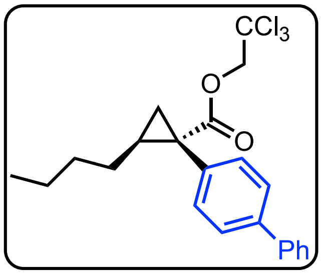

General procedure 2 was employed for the cyclopropanation of Hex-1-ene (250 μL, 2.000 mmol, 10 equiv) with 2,2,2-trichloroethyl 2-diazo-2-(4-fluorophenyl)acetate (0.2 mmol, 73.9 mg, 1.0 equiv) using Ru_2(*S*-TPPTTL)$_4$BAr^F (6.6 mg, 1 mol %) as catalyst. Purification by column chromatography afforded a clear colorless oil (42.6 mg, 50%).
^{1}H NMR (400 MHz, CDCl$_3$) δ 7.62 (d, J = 8.3 Hz, 2H), 7.60 (d, J = 8.6 Hz, 2H) 7.48 (d, J = 7.6 Hz, 2H), 7.43-7.33 (m, 3H), 4.85 (d, J = 12.0 Hz, 1H), 4.61 (d, J = 12.0 Hz, 1H), 2.00 (m, 1H), 1.92 (dd, J = 9.1, 4.1 Hz, 1H), 1.51-1.37 (m, 3H), 1.29 (pd, J = 8.5, 5.8 Hz, 3H), 0.86 (t, J = 7.2 Hz, 3H), 0.74-0.64 (m, 1H).
^{13}C NMR (101, CDCl$_3$) δ 173.1, 140.8, 140.1, 134.4, 131.8, 128.8, 127.3, 127.1, 126.7, 95.2, 74.2, 33.3, 31.3, 30.0, 29.7, 22.4, 21.9, 14.0.
HRMS (+p APCI) calcdd for $C_{22}H_{24}Cl_3O_2$ (M+H) 425.0836 found 425.0830.

IR (neat): 3029, 2954, 2928, 2857, 1732, 1599, 1521, 1488, 1447, 1366, 1332, 1257, 1236, 1101, 1046, 1008, 969, 907, 888, 840, 807 (cm^{-1}).
Chiral HPLC: The enantiopurity was determined to be 90:10 er by chiral HPLC analysis (Chiracel OD-H, 1.0% IPA/Hexanes, 1.0 mL/min, λ=230 nm, RT: Major: 6.6 min, Minor: 5.3 min).

2,2,2-Trichloroethyl (1*S*,2*S*)-2-butyl-1-(naphthalen-2-yl)cyclopropane-1-carboxylate (40)

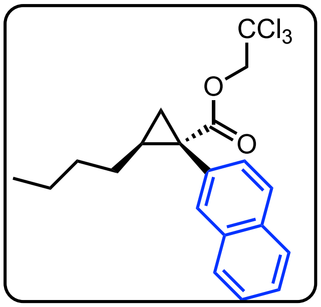

General procedure 2 was employed for the cyclopropanation of Hex-1-ene (250 μL, 2.0 mmol, 10 equiv) with 2,2,2-trichloroethyl 2-diazo-2-(naphthalen-2-yl)acetate (0.2 mmol, 68.7 mg, 1.0 equiv) using Ru_2(*S*-TPPTTL)$_4$BAr^F (6.6 mg, 1 mol %) as catalyst. Purification by column chromatography afforded an oil (46.3 mg, 58%). Spectra matched literature precedent.[13]
^{1}H NMR (400 MHz, $CDCl_3$) δ 7.83 (m, 3H), δ 7.75 (d, J = 1.7 Hz, 1H), δ 7.48 (m, 3H), δ 4.86 (d, J = 11.9 Hz, 1H), δ 4.55 (d, J = 11.9 Hz, 1H), δ 2.03 (tdd, J = 9.0, 6.4, 4.3 Hz, 1H), δ 1.95 (dd, J = 9.0, 4.3 Hz, 1H), δ 1.40 (ddt, J = 14.7, 9.0, 6.4 Hz, 4H), δ 1.22 (tdd, J = 14.7, 7.3, 1.3 Hz, 2H), δ 0.81 (t, J = 7.3, 3H), δ 0.62 (m, 1H).
Chiral HPLC: The enantiopurity was determined to be 97:3 er by chiral HPLC analysis (Chiracel AD-H, 1.0% IPA/Hexanes, 1.0 mL/min, λ=280 nm, RT: Major: 4.9 min, Minor: 5.4 min).

2,2,2-Trichloroethyl (1*S*,2*S*)-2-butyl-1-(3-iodophenyl)cyclopropane-1-carboxylate (41)

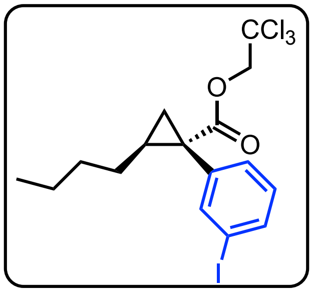

General procedure 2 was employed for the cyclopropanation of Hex-1-ene (250 μL, 2.0 mmol, 10 equiv) with 2,2,2-trichloroethyl 2-diazo-2-(3-iodophenyl)acetate (0.2 mmol, 83.9 mg, 1.0 equiv) using Ru_2(*S*-TPPTTL)$_4$BAr^F (6.6 mg, 1 mol %) as catalyst. Purification by column chromatography afforded an oil (62.8 mg, 63%).
^{1}H NMR (400 MHz, $CDCl_3$) δ 7.70 (t, J = 1.7 Hz, 1H), 7.64 (ddd, J = 7.8, 1.7, 1.0 Hz, 1H), 7.35-7.27 (m, 1H), 7.09 (t, J = 7.8 Hz, 1H), 4.84 (d, J = 11.9 Hz, 1H), 4.55 (d, J = 11.90 Hz, 1H), 1.94 (ddt, J = 8.9, 6.7, 4.6 Hz, 1H), 1.87 (dd, J = 9.0, 4.2 Hz, 1H), 1.40 (ttd, J = 8.9, 4.4, 1.9 Hz, 3H), 1.33-1.25 (m, 2H), 1.22 (dd, J = 6.8, 4.2 Hz, 1H), 0.86 (t, J = 7.3 Hz, 3H), 0.68-0.56 (m, 1H).
^{13}C NMR (101 MHz, $CDCl_3$) δ 172.4, 140.5, 137.7, 136.4, 130.8, 129.6, 95.0, 93.7, 74.3, 33.2, 31.2, 29.9, 29.8, 22.4, 21.9, 14.0.
HRMS (+p APCI) calcdd for $C_{16}H_{19}Cl_3IO_2$ (M+H) 474.9490 found 474.9486.
IR (neat): 2954, 2927, 2856, 1732, 1590, 1560, 1466, 1411, 1366, 1326, 1264, 1237, 1161, 1105, 1045, 995, 975, 893, 813 (cm^{-1}).
Chiral HPLC: The enantiopurity was determined to be 89:11 er by chiral HPLC analysis (Chiracel OD-H, 1.0% IPA/Hexanes, 0.25 mL/min, λ=230 nm, RT: Major: 19.0 min, Minor: 17.0 min).

2,2,2-Trichloroethyl (1*S*,2*S*)-2-butyl-1-((*E*)-styryl)cyclopropane-1-carboxylate (42)

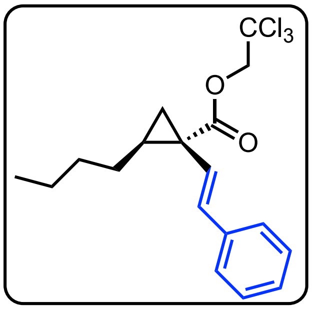

General procedure 2 was employed for the cyclopropanation of Hex-1-ene (250 μL, 2.0 mmol, 10 equiv) with 2,2,2-trichloroethyl (*E*)-2-diazo-4-phenylbut-3-enoate (0.2 mmol, 63.9 mg, 1.0 equiv) using Ru_2(*S*-TPPTTL)$_4$BAr^F (6.6 mg, 1 mol %) as catalyst. Purification by column chromatography afforded an oil (4.7 mg, 6%):

^{1}H NMR (400 MHz, CDCl$_3$) δ 7.40 (dd, J = 8.32, 1.40 Hz, 2H), 7.36-7.30 (m, 2H), 7.26-7.21 (m, 2H), 6.67 (d, J = 16.0 Hz, 1H), 6.40 (d, J = 16.0 Hz, 1H), 4.80 (d, J = 11.9 Hz, 1H), 4.71 (d, J = 11.9 Hz, 1H), 1.84-1.72 (m, 2H), 1.38-1.27 (m, 6H), 1.27-1.19 (m, 2H), 0.86 (t, J = 7.1 Hz, 3H).

^{13}C NMR (101 MHz, CDCl$_3$) δ 172.1, 137.0, 132.4, 128.6, 127.5, 126.3, 123.6, 95.2, 74.2, 32.6, 31.5, 30.4, 27.8, 22.3, 19.9, 14.0.

HRMS (+p ACPI) clac for $C_{18}H_{22}O_2Cl_3$ (M+H) 375.0680 found 375.0678.

IR (neat): 3025, 2954, 2926, 2856, 1733, 1600, 1494, 1448, 1368, 1235, 1212, 1142, 1046, 964, 812, (cm^{-1}).

Chiral HPLC: The enantiopurity was determined to be 92:8 er by chiral HPLC analysis (Chiracel OD-H, 1.0% IPA/Hexanes, 1.0 mL/min, λ=230 nm, RT: Major: 15.6 min, Minor: 7.0 min).

2,2,2-Trichloroethyl (1*S*,2*S*)-2-butyl-1-(2-chloropyridin-4-yl)cyclopropane-1-carboxylate (43)

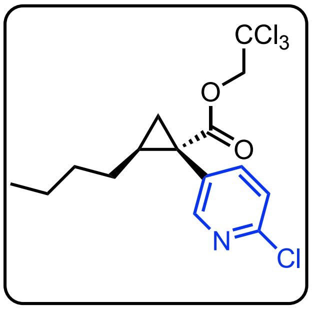

A modified General procedure 2 was employed with the temperature set to 40 °C for the cyclopropanation of Hex-1-ene (250 μL, 2.0 mmol, 10 equiv) with 2,2,2-trichloroethyl 2-(6-chloropyridin-3-yl)-2-diazoacetate (0.2 mmol, 65.8 mg, 1.0 equiv) using Ru_2(*S*-TPPTTL)$_4$BAr^F (6.6 mg, 1 mol %) as catalyst. Purification by column chromatography afforded a clear colorless oil (54.4 mg, 67%).

Spectra matched literature precedent.[13]

^{1}H NMR (400 MHz, CDCl$_3$): δ 8.35 (m, 1H), δ 7.64 (dd, J = 8.2, 2.5 Hz, 1H), δ 7.33 (dd, J = 8.2, 0.7 Hz, 1H), δ 4.80 (d, J = 11.9 Hz, 1H), δ 4.59 (d, J = 11.9 Hz, 1H), δ 1.97 (m, 2H), δ 1.40 (m, 3H), δ 1.26 (m, 3H), δ 0.85 (t, J = 7.31 Hz, 3H), δ 0.60 (m, 1H).

Chiral HPLC The enantiopurity was determined to be 89:11 er by chiral HPLC analysis (Chiracel OD-H, 1.0% IPA/Hexanes, 1.0 mL/min, λ=254 nm, RT: Major: 6.5 min, Minor: 4.0 min).

2,2,2-Trichloroethyl (1*S*,2*S*)-2-butyl-1-(3-methoxyphenyl)cyclopropane-1-carboxylate (44)

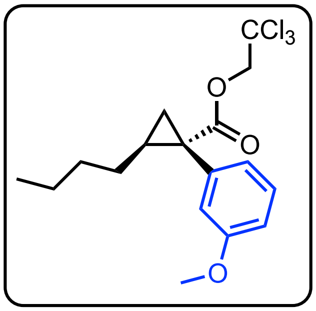

General procedure 2 was employed for the cyclopropanation of Hex-1-ene (250 µL, 2.0 mmol, 10 equiv) 2,2,2-trichloroethyl 2-diazo-2-(4-methoxyphenyl)acetate (0.2 mmol, 64.7 mg, 1.0 equiv) using Ru_2(*S*-TPPTTL)$_4$BAr^F (6.6 mg, 1 mol %) as catalyst. Purification by column chromatography afforded a clear colorless oil (48.8 mg, 54%):

^{1}H NMR (400 MHz, $CDCl_3$) δ 7.29 (d, J = 8.8 Hz, 1H), δ 6.94 (m, 1H), δ 6.89 (m, 1H), δ 6.87 (m, 1H), δ 4.86 (d, J = 11.9 Hz, 1H), δ 4.59 (d, J = 11.9 Hz, 1H), δ 3.85 (s, 3H), δ 1.97 (tdd, J = 9.1, 6.8, 4.3 Hz, 1H), δ 1.87 (dd, J = 9.1, 4.3 Hz, 1H), δ 1.43 (m, 3H), δ 1.30 (m, 2H), δ 1.25 (m, 1H), δ 0.87 (t, J = 7.3 Hz, 3H), 0.67 (m, 1H).

^{13}C NMR (101 MHz, $CDCl_3$) δ 173.0, 159.2, 136.8, 128.9, 123.9, 117.2, 112.9, 95.2, 74.2, 55.2, 33.6, 31.3, 29.9, 29.6, 22.0, 22.4, 14.0.

HRMS (+p APCI) calcd for $C_{17}H_{21}Cl_3O_3$ (M+H) 378.0551 found 378.0549.

IR (neat) 2955, 2930, 2858, 1732, 1601, 1489, 1451, 1433, 1367, 1336, 1286, 1258, 1237, 1155, 1103, 1042, 978, 913, 861, 834, 803 (cm^{-1}).

Chiral HPLC The enantiopurity was determined to be 88:12 er by chiral HPLC analysis (R,R, Whelk 1.0% IPA/Hexanes, 1.0 mL/min, λ=230 nm, RT: Major: 6.5 min, Minor: 9.6 min).

Catalyst Synthesis

Synthesis of Cu_2(*S*-TPPTTL)$_4$

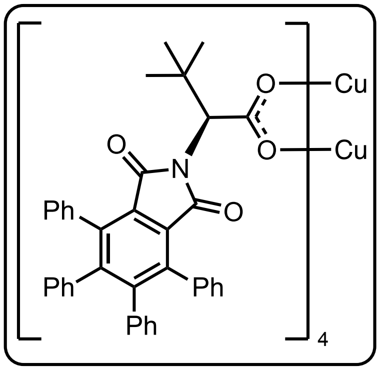

In a dry, 25 mL round-bottomed flask, copper(II) acetate (50 mg, 0.13 mmol) and *S*-TPPTTL ligand (620 mg, 0.630 mmol) were dissolved in chlorobenzene (6.3 mL) to yield a blue suspension. The flask was fitted with a soxhelet extractor filled with sand, K_2CO_3, and glass wool. The top of the extractor was fitted with a reflux condenser. The solution was heated to reflux in an aluminum block at 165°C under nitrogen atmosphere to yield a dark blue solution, with acetic acid being removed from the condensate by the K_2CO_3. After refluxing for 20 h, the condenser and reflux condenser were removed, and replaced by a short path distillation apparatus. The chlorobenzene was removed by distillation at atmospheric pressure. The dark blue residue was dissolved in CH_2Cl_2 and washed twice with water, dried over anhydrous Na_2SO_4, filtered, concentrated onto silica gel, and quickly chromatographed via flash column chromatography (0-40% EtOAc/Hexanes). The blue fractions with no ligand by TLC were combined and evaporated to afford a blue powdery solid (51% yield, 335mg, 0.14 mmol). The solid was suspended in MeCN (4ml) and left in a vial to slowly evaporate over several months. Once light blue crystals had formed, they were harvested from the liquor and analyzed by X-ray crystallography to confirm compound identity. To remove the axial MeCN ligands, the crystals were dried in a vial under high vacuum for several weeks

to afford dark blue crystals. Progress of axial ligand removal can be clearly observed with the distinct color change in the complex.

Synthesis of Co_2(*S*-TPPTTL)$_4$

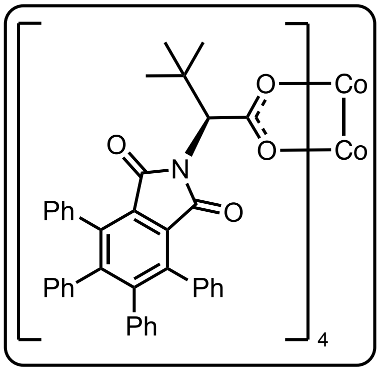

In a nitrogen-filled glovebox, a solution of KHMDS (200 mg, 1.00 mmol) in THF (6 mL) was added dropwise to a solution of *S*-TPPTTL (566 mg, 1.00 mmol) in THF (6 mL) and stirred for 5 mins. To this mixture, a partially dissolved suspension of $CoCl_2$ (64.9 mg, 0.50 mmol) in THF (10 mL) was transferred dropwise over 5 mins, resulting in a deep purple colored mixture that was stirred for 16h at r.t. At this time, all volatiles were removed *in vacuo* and resulting powder was reconstituted in DCM (25 mL), then filtered. All volatiles were removed from the filtrate *in vacuo,* affording a magenta-colored power (532 mg).

HRMS (+p ESI): Calcd for $C_{152}H_{121}O_{16}N_4{}^{59}Co_2$ 2375.7436, found 2375.7428.
IR (neat): 3409, 2057, 2957, 1769, 1707, 1601, 1515, 1442, 1372, 1347, 1264, 1239, 1183, 1120, 1072, 1026, 944, 912, 895, 866, 808 (cm^{-1}).

Ru_2(*S*-TPPTTL)$_4$Cl (4-Cl)

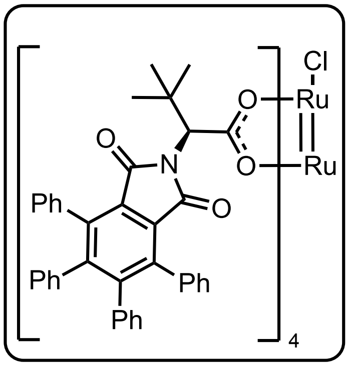

Into a 25 mL round bottom flask equipped with a stir bar was added TPPTTL ligand (0.955 g, 1.74 mmol, 8 equiv) and $Ru_2(OAc)_4Cl$ (0.100 g, 0.218 mmol, 1 equiv). Then 20 mL of chlorobenzene was added. The flask was fitted to a Soxhlet extractor, and the thimble was charged with glass wool, K_2CO_3, and a small layer of sand. The reaction was heated to 168 °C and a rigorous reflux was observed. The reaction was left for 24 h. The reaction was monitored by TLC. Once a brown moving spot was seen on TLC, the reaction was stopped, the solvent removed, and the product was dry loaded onto silica gel and subjected to flash chromatography (0-13% EtOAc/Hex). The product eluted as a brown band to afford a brown solid upon concentration (0.391 mg, 83% yield). Crystals suitable for X-ray crystallography were grown from the slow evaporation of layered hexane over toluene. No NMR data are available for this compound due to its paramagnetic character. The key data for the structural characterization were obtained by HRMS and X-ray crystallography.

HRMS (+p ESI): Calcd for $C_{152}H_{120}O_{16}N_4{}^{96}Ru_2$ (M-Cl) 2448.6846 found 2448.6907
IR (neat): 3057, 2958, 1772, 1716, 1602, 1499, 1481, 1442, 1400, 1365, 1343, 1300, 1266, 1182, 1118, 1086, 1074, 1027, 1000, 944, 898, 864, 835, 803 (cm^{-1}).

Ru$_2$(*S*-TPPTTL)$_4$BAr^F (4-BAr^F)

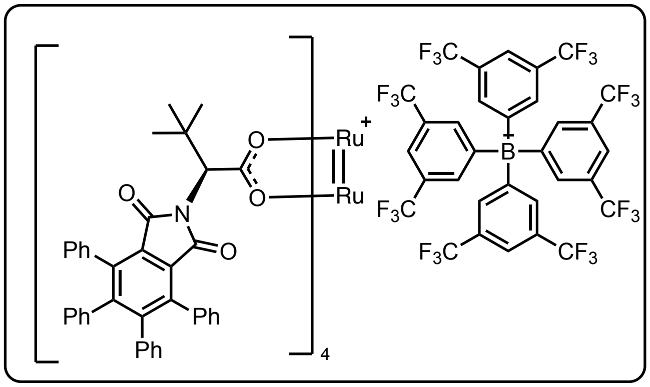

To a 20 mL vial was equipped with a stir bar was added Ru$_2$(*S*-TPPTTL)$_4$Cl (250 mg, 0.10 mmol, 1.0 equiv) which was subsequently dissolved in 1.00 mL of DCM. Then, NaBAr^F (88.8 mg, 0.10 mmol, 1.0 equiv) was added in one portion. The reaction was left to stir for 24 h, at which point the resulting solution was passed through a short silica plug (1:1 DCM/EtOAc eluent), the solution was concentrated and dried to afford a brown/orange solid (308 mg, 93% yield).

HRMS (+p ESI): Calcd for $C_{152}H_{120}N_4O_{16}Ru_2^+$ (M^+) 2448.6846 found 2448.6870
HRMS (-p ESI): Calcd for $C_{32}H_{12}BF_{24}^-$ (M^-) 862.0691 found 863.0716.
IR (neat): 3059, 2969, 1772, 1717, 1652, 1607, 1479, 1443, 1414, 1336, 1354, 1305, 1275, 1163, 1123, 1027, 1000, 944, 912, 886, 838, 803 (cm^{-1}).

Ru$_2$(*S*-PTTL)$_4$Cl (5-Cl)

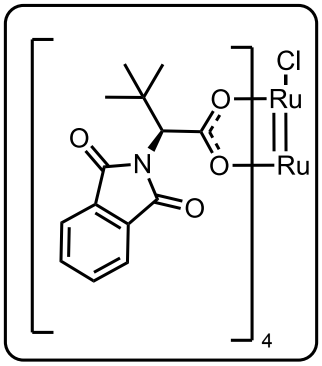

Into a 25 mL round bottom flask equipped with a stir bar was added PTTL ligand (0.441 g, 1.69 mmol, 8 equiv) and Ru$_2$(OAc)$_4$Cl (0.100 g, 0.211 mmol, 1 equiv). Then 16 mL of chlorobenzene was added. The flask was fitted to a Soxhlet extractor, and the thimble was charged with glass wool, K_2CO_3, and a small layer of sand. The reaction was heated to 168 °C and a rigorous reflux was observed. The reaction was left for 24 hrs. The reaction was monitored by TLC. Once a brown moving spot was seen on TLC, the reaction was stopped, the solvent removed, and the product was dry loaded onto silica gel and subjected to flash chromatography (0-3% Methanol/DCM). The product eluted as a brown band and afforded a brown solid upon concentration (71.4 mg, Yield 27%). Crystals suitable for X-ray crystallography were grown from the vapor diffusion of acetonitrile into a solution of toluene.

HR-MS: Calcd for $C_{56}H_{56}O_{16}N_4{}^{96}Ru_2$ (M-Cl) 1232.1838 found 1232.1866.
IR (neat): 2963, 1776, 1715, 1611, 1504, 1481, 1467, 1380, 1333, 1294, 1189, 1106, 1087, 1043, 995, 900, 871 (cm^{-1}).

Ru_2(*S*-PTTL)$_4$BAr^F (5-BAr^F)

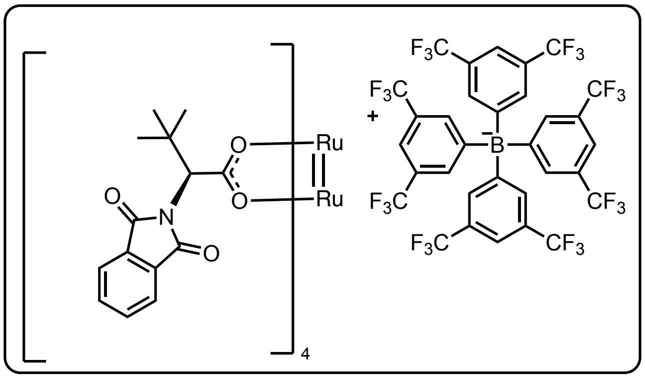

To a 20 mL vial was equipped with a stir bar was added Ru_2(S-PTTL)$_4$Cl (165.5 mg, 0.129 mmol, 1.0 equiv) which was subsequently dissolved in 0.50 mL of DCM. Then, NaBAr^F (120.4 mg, 0.1359 mmol, 1 equiv) was added in one portion. The reaction was left to stir for 24 h, at which point the resulting solution was passed through a short silica plug (1:1 DCM/EtOAc eluent), the solution was concentrated and dried to afford a brown/orange solid (265 mg, 97% yield)

HRMS (+p ESI): Calcd for $C_{56}H_{56}O_{16}N_4{}^{96}Ru_2$ (M^+) 1232.1838 found 1232.1859

HRMS (-p ESI): Calcd for $C_{32}H_{12}BF_{24}^-$ (M^-) 862.0691 found 862.0707.

IR (neat): 2970, 1778, 1717, 1610, 1480, 1469, 1382, 1352, 1273, 1119, 1042, 998, 933, 900, 887, 871, 838 (cm^{-1}).

Ru_2(*S*-PTAD)$_4$Cl (6-Cl)

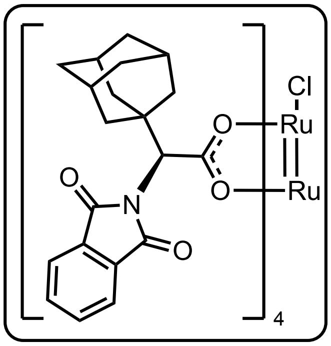

Into a 25 mL round bottom flask equipped with a stir bar was added PTAD ligand (0.344 g, 1.01 mmol, 8 equiv) and $Ru_2(OAc)_4Cl$ (60.0 mg, 0.126 mmol, 1 equiv). Then 16 mL of chlorobenzene was added. The flask was fitted to a Soxhlet extractor, and the thimble was charged with glass wool, K_2CO_3, and a small layer of sand. The reaction was heated to 168 °C and a rigorous reflux was observed. The reaction was left for 24 hrs. The reaction was monitored by TLC. Once a brown moving spot was seen on TLC, the reaction was stopped, the solvent removed, and the product was dry loaded onto silica gel and subjected to flash chromatography (0-3% DCM/Methanol). The product eluted as a brown band and afforded a brown solid upon concentration (90.0 mg, Yield 45%). Crystals suitable for X-ray crystallography were grown from the vapor diffusion of acetonitrile into a solution of toluene.

HRMS (+p ESI): Calcd for $C_{80}H_{80}O_{16}N_4{}^{96}Ru_2$ (M-Cl) 1544.3716 found 1544.3709.

IR (neat): 2902, 2848, 1775, 1712, 1611, 1497, 1468, 1447, 1380, 1349, 1332, 1314, 1301, 1287, 1265, 1239, 1172, 1112, 1094, 1086, 1045, 994, 969, 944, 898, 876 (cm^{-1}).

Ru_2(*S*-PTAD)$_4$BAr^F (6-BAr^F)

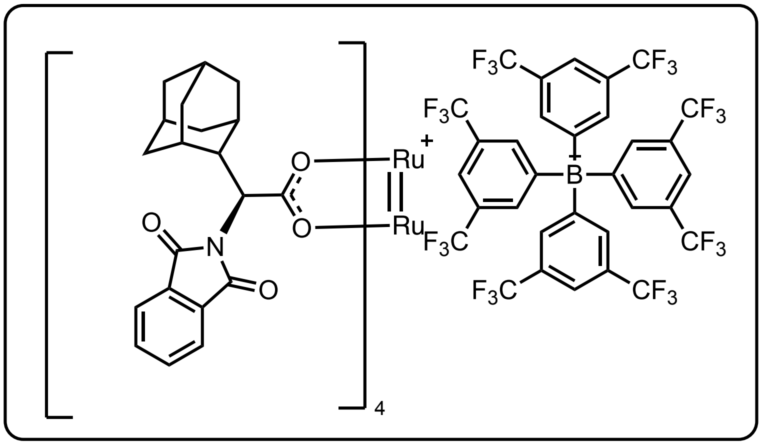

To a 20 mL vial was equipped with a stir bar was added Ru_2(*S*-PTAD)$_4$Cl (66.3 mg, 0.041 mmol, 1.0 equiv) which was subsequently dissolved in 0.50 mL of DCM. Then, NaBAr^F (38.8 mg, 0.0438 mmol, 1.05 equiv) was added in one portion. The reaction was left to stir for 24 h, at which point the resulting solution was passed through a short silica plug (1:1 DCM/EtOAc eluent), the solution was concentrated and dried to afford a brown/orange solid (76 mg, 75% Yield)

HRMS (+p ESI): Calcd for $C_{80}H_{80}O_{16}N_4{}^{96}Ru_2$ (M^+) 1544.3716 found 1544.3717.

HRMS (-p ESI): Calcd for $C_{32}H_{12}BF_{24}^-$ (M^-) 862.0691 found 862.0709

IR (neat): 2908, 2854, 1778, 1720, 1611, 1470, 1408, 1382, 1353, 1276, 1125, 1045, 944, 898, 839 (cm^{-1}).

Ru_2(*S*-TCPTAD)$_4$Cl (7-Cl)

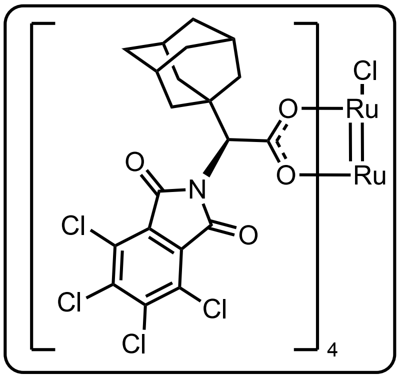

Into a 25 mL round bottom flask equipped with a stir bar was added TCPTAD ligand (0.379 g, 0.794 mmol, 8 equiv) and $Ru_2(OAc)_4Cl$ (47.0 mg, 0.099 mmol, 1 equiv). Then 16 mL of chlorobenzene was added. The flask was fitted to a Soxhlet extractor, and the thimble was charged with glass wool, K_2CO_3, and a small layer of sand. The reaction was heated to 168 °C and a rigorous reflux was observed. The reaction was left for 24 hrs. The reaction was monitored by TLC. Once a brown moving spot was seen on TLC, the reaction was stopped, the solvent removed, and the product was dry loaded onto silica gel and subjected to flash chromatography (0-14% Hexanes/Ethyl Acetate). The product eluted as a brown band and afforded a brown solid upon concentration (115.0 mg, Yield 54%). Crystals suitable for X-ray crystallography were grown from the vapor diffusion of acetonitrile into a solution of toluene.

HRMS (+p ESI): Calcd for $C_{100}H_{80}Cl_{20}N_5O_{20}Ru_2$ (M-Cl+TCPTAD Ligand) 2561.7310 found 2561.7453
IR (neat): 3333, 2903, 2849, 1780, 1720, 1448, 1385, 1368, 1355, 1344, 1328, 1297, 1263, 1198, 1174, 1123, 1098, 1045, 976, 936, 868 (cm^{-1}).

Ru_2(*S*-TCPTAD)$_4$BAr^F (7-BAr^F)

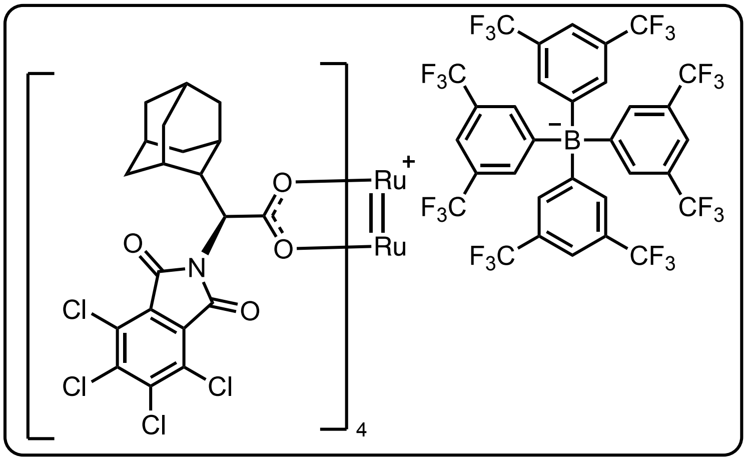

To a 20 mL vial was equipped with a stir bar was added Ru_2(S-TCPTAD)$_4$Cl (227 mg, 0.106 mmol, 1.0 equiv) which was subsequently dissolved in 1.00 mL of DCM. Then, NaBAr^F (98.6 mg, 0.111 mmol, 1.05 equiv) was added in one portion. The reaction was left to stir for 24 h, at which point the resulting solution was passed through a short silica plug (1:1 DCM/EtOAc eluent), the solution was concentrated and dried to afford a brown/orange solid (237 mg, 87% Yield)
HRMS (+p ESI): Calcd for $C_{80}H_{64}Cl_{16}N_4O_{16}{}^{96}Ru_2$ (M^+) 2087.7480 found 2087.7690
HRMS (-p ESI): Calcd for $C_{32}H_{12}BF_{24}^-$ (M^-) 862.0691 found 862.0689
IR (neat): 2906, 2854, 1781, 1724, 1665, 1610, 1478, 1447, 1408, 1384, 1368, 1353, 1328, 1315, 1275, 1199, 1161, 1122, 1045, 996, 886, 838, 827, 809 (cm^{-1}).

Ru_2(*S*-NTTL)$_4$Cl (8-Cl)

Into a 25 mL round bottom flask equipped with a stir bar was added NTTL ligand (1.04 g, 3.35 mmol, 8 equiv) and $Ru_2(OAc)_4Cl$ (200.0 mg, 0.419 mmol, 1 equiv). Then 16 mL of chlorobenzene was added. The flask was fitted to a Soxhlet extractor, and the thimble was charged with glass wool, K_2CO_3, and a small layer of sand. The reaction was heated to 168 °C and a rigorous reflux was observed. The reaction was left for 24 hrs. The reaction was monitored by TLC. Once a brown moving spot was seen on TLC, the reaction was stopped, the solvent removed, and the product was dry loaded onto silica gel and subjected to flash chromatography (0-4% DCM/Methanol). The

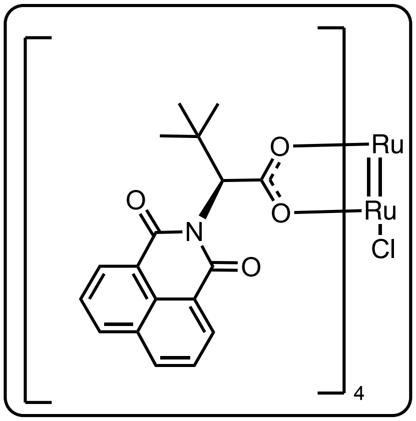

product eluted as a brown band and afforded a brown solid upon concentration (268.3 mg, Yield 43%). Crystals suitable for X-ray crystallography were grown from slow evaporation of HFIP.
HRMS (+p ESI): Calcd for $C_{72}H_{64}O_{16}N_4{}^{96}Ru_2$ (M-Cl) 1432.2464 found 1432.2525.
IR (neat): 2957, 1702, 1665, 1628, 1588, 1480, 1435, 1411, 1398, 1375, 1356, 1338, 1299, 1237, 1179, 1149, 1111, 1075, 1029, 996, 905, 865, 846 (cm^{-1}).

Ru_2(*S*-NTTL)$_4$BAr^F (8-BAr^F)

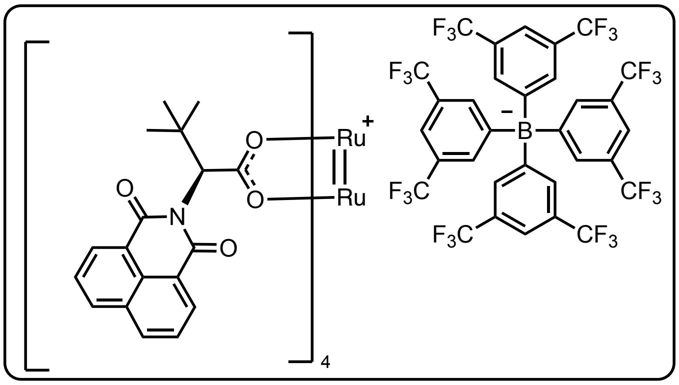

To a 20 mL vial was equipped with a stir bar was added Ru_2(S-NTTL)$_4$Cl (100 mg, 0.067 mmol, 1.0 equiv) to which 0.50 mL of DCM was added. This resulted in a cloudy brown/red solution. Then, NaBAr^F (65.8 mg, 0.074 mmol, 1.1 equiv) was added in one portion, and an immediately the soltuoin went to a clear, dark orange color. The reaction was left to stir for 24 h, at which point the resulting solution was passed through a short silica plug (1:1 DCM/EtOAc eluent), the solution was concentrated and dried to afford a brown/orange solid (153.6 mg, 99% Yield)
HRMS (+p ESI): Calcd for $C_{72}H_{64}O_{16}N_4{}^{96}Ru_2$ (M^+) 1432.2460 found 1432.2488.
HRMS (-p ESI): Calcd for $C_{32}H_{12}BF_{24}^-$ (M^-) 862.0691 found 862.0704.
IR (neat): 2969, 1703, 1666, 1558, 1479, 1436, 1411, 1399, 1375, 1352, 1300, 1273, 1237, 1178, 1119, 1076, 1039, 996, 931, 905, 886, 866, 845, 839 (cm^{-1}).

Catalyst HR-MS

All reported HR-MS values fall below the 5 ppm delta threshold except for $Ru_2(TCPTAD)_4Cl$ (Δ = 5.45 ppm) and $Ru_2(TCPTAD)_4BAr^F$ (Δ = 9.99 ppm). Reported masses were taken from the lowest isotopic peak which could be the cause for the error observed in these two complexes. When taking the average isotopic peak the calculated Δ value is significantly lowered to below the 5 ppm threshold.

Co_2(*S*-TPPTTL)$_4$ (Top, observed. Bottom, simulated)

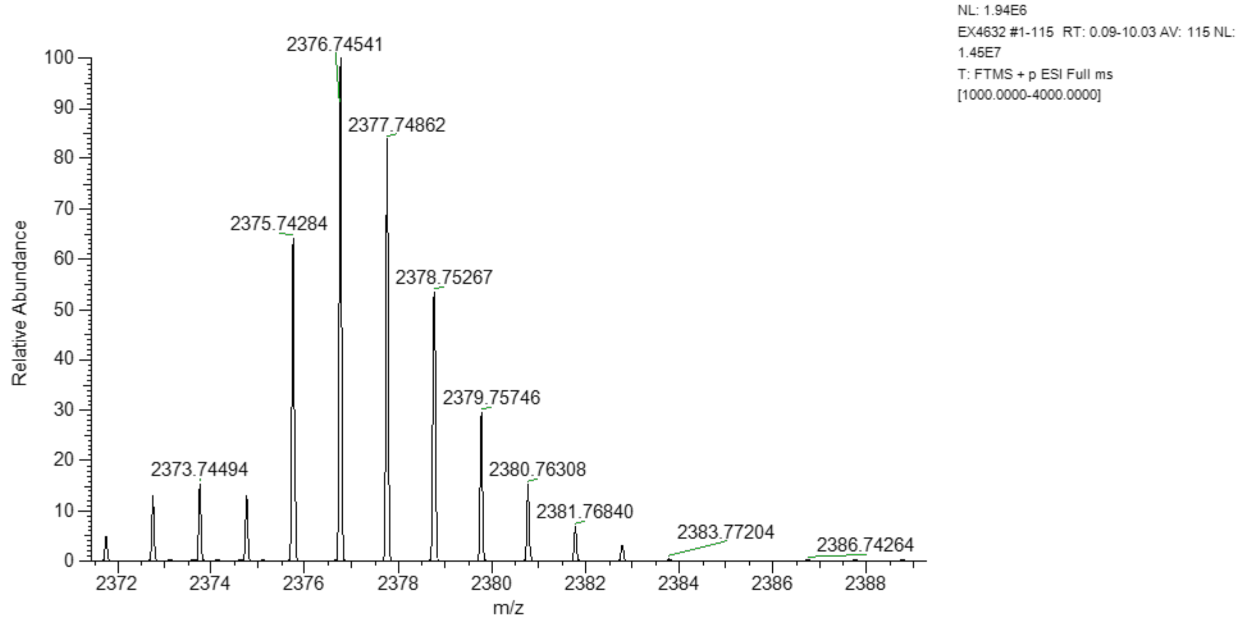

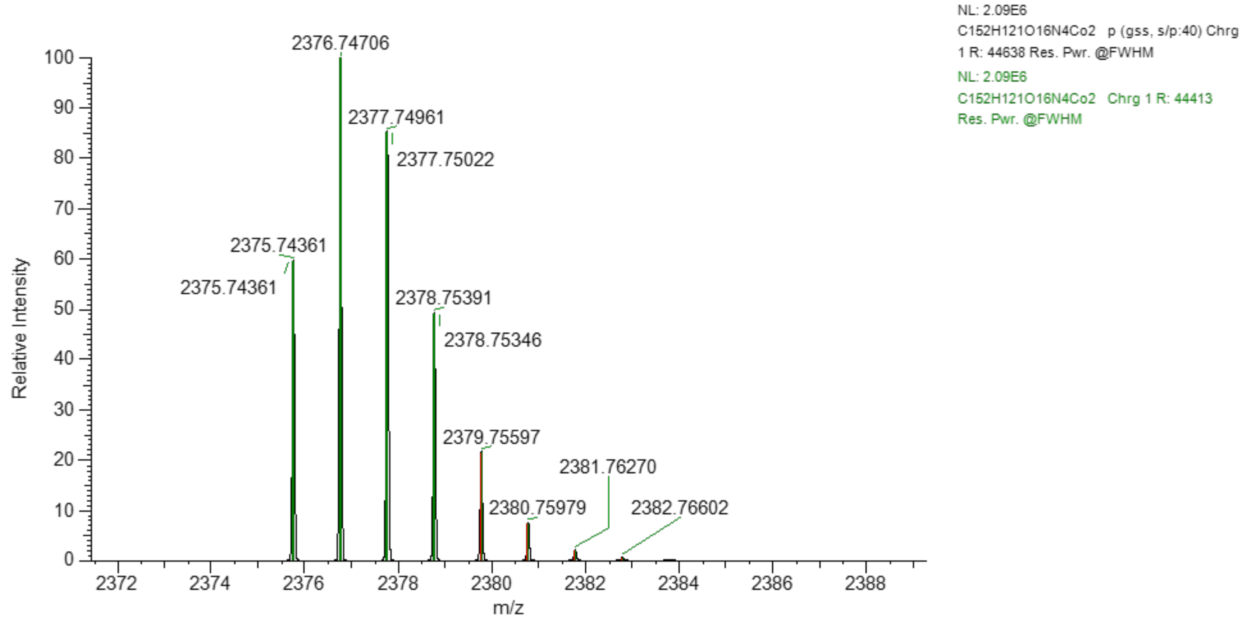

Ru_2(*S*-TPPTTL)$_4$Cl (4-Cl) (Top, observed. Bottom, simulated)

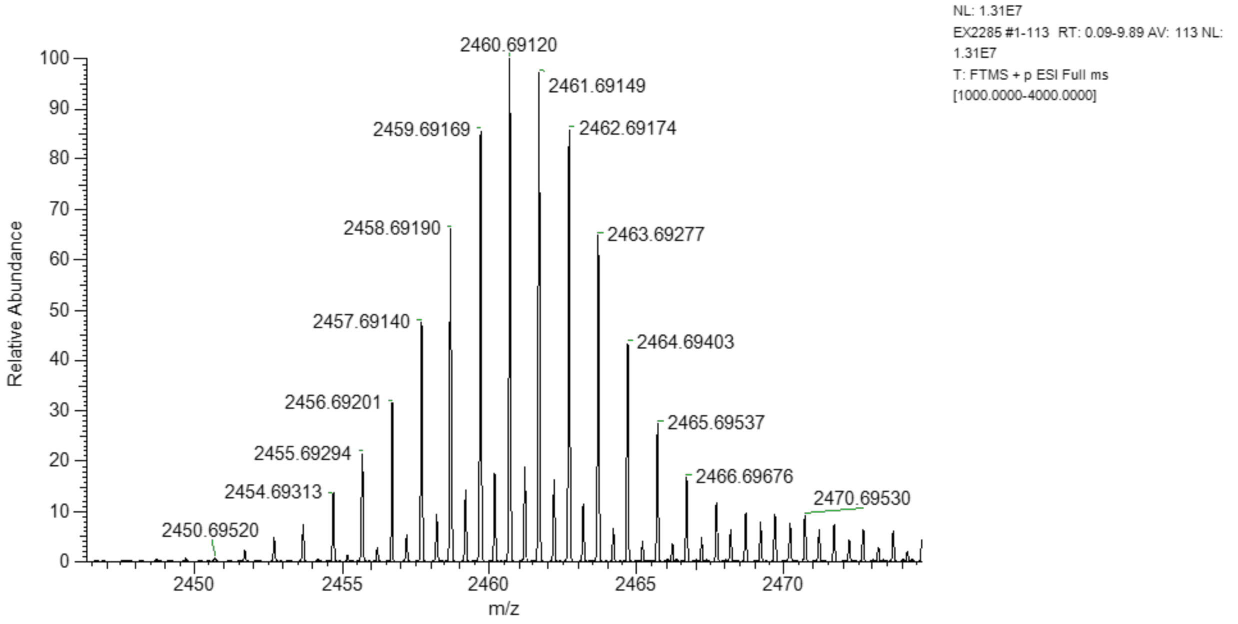

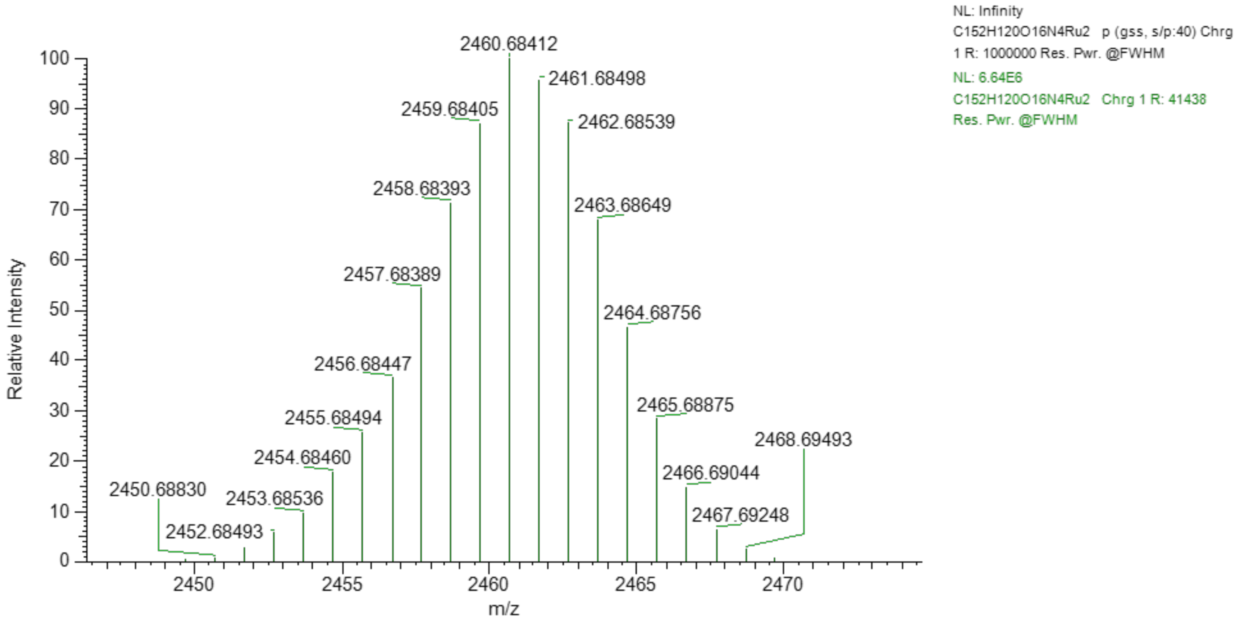

Ru$_2$(*S*-TPPTTL)$_4$BAr^F (4-BAr^F) (Top, observed. Bottom, simulated)

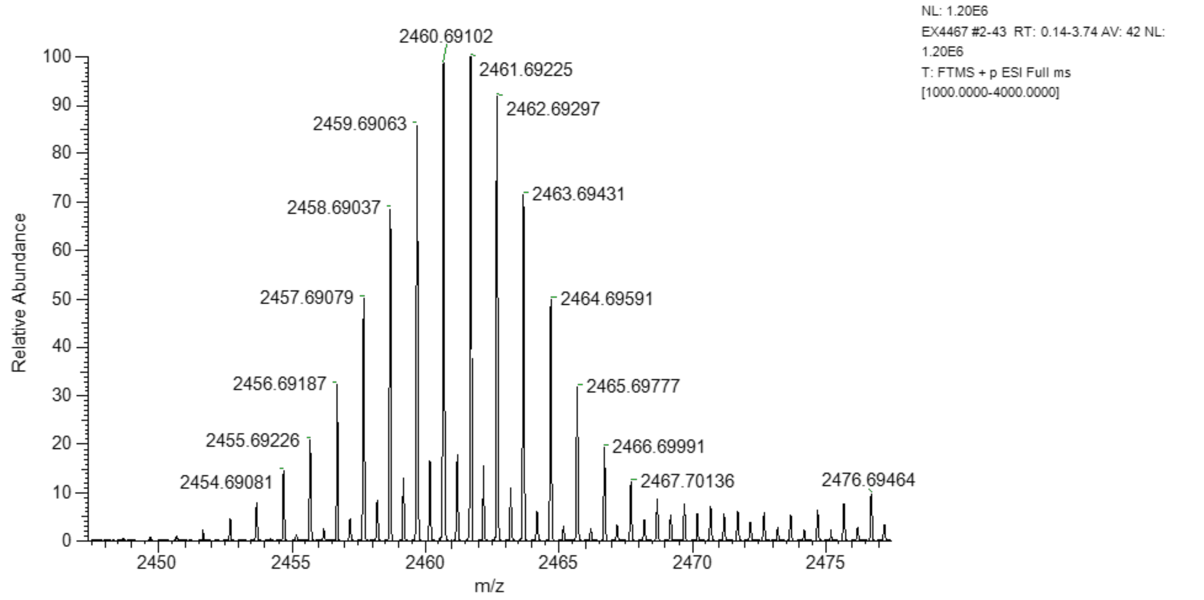

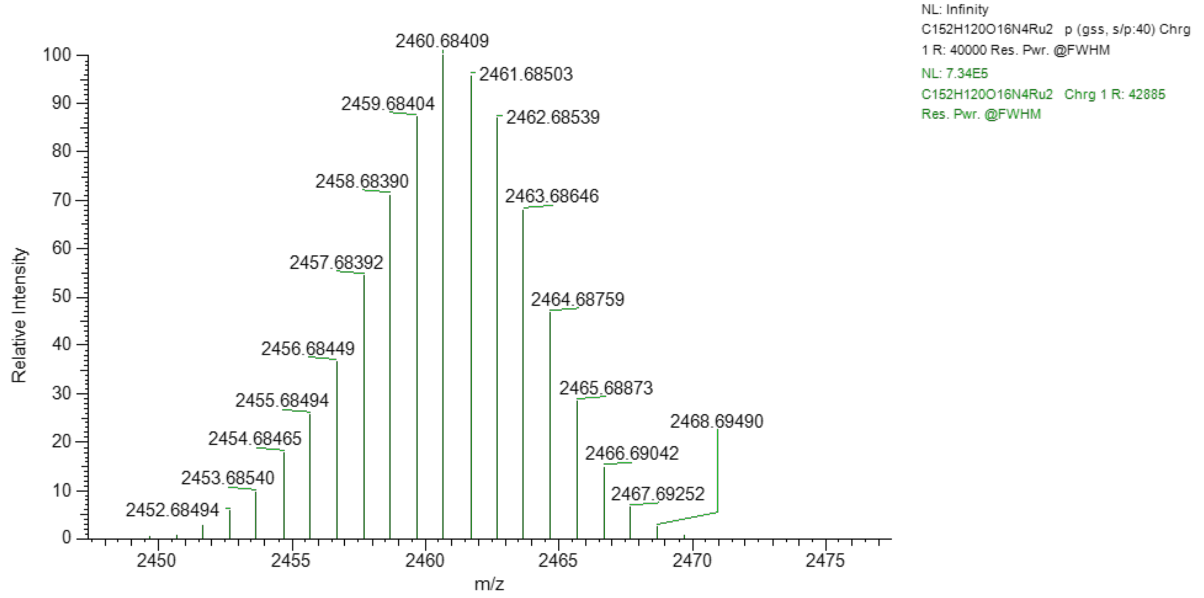

BAr^F Counter Ion (Top, observed. Bottom, simulated)

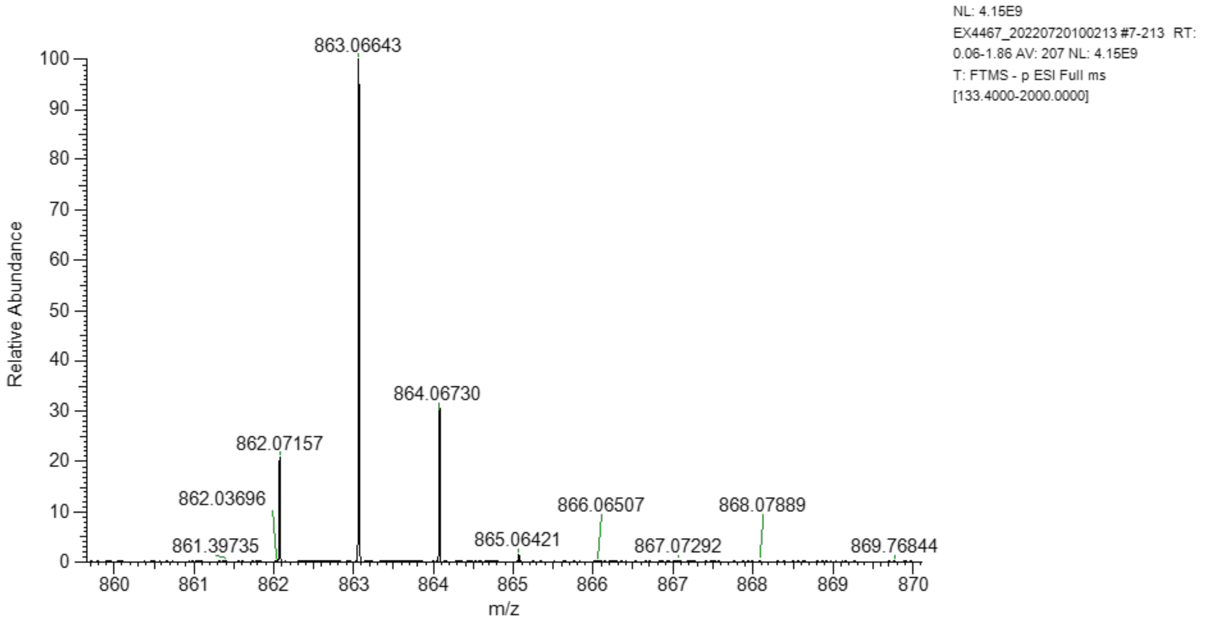

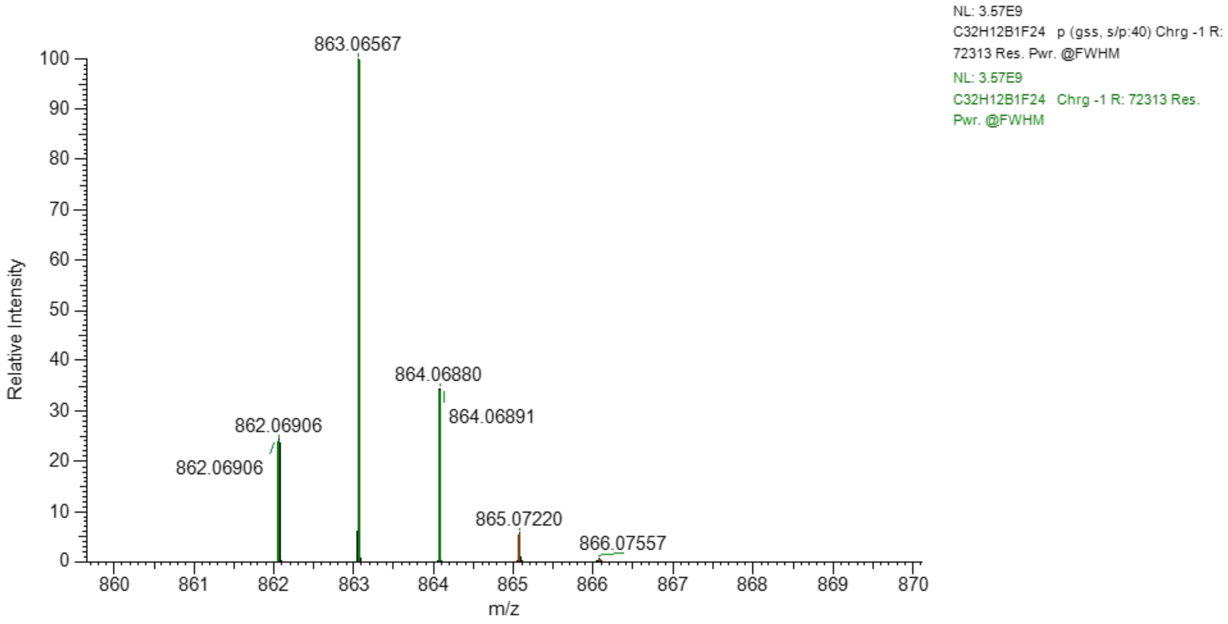

Ru$_2$(*S*-PTTL)$_4$Cl (5-Cl) (Top, observed. Bottom, simulated)

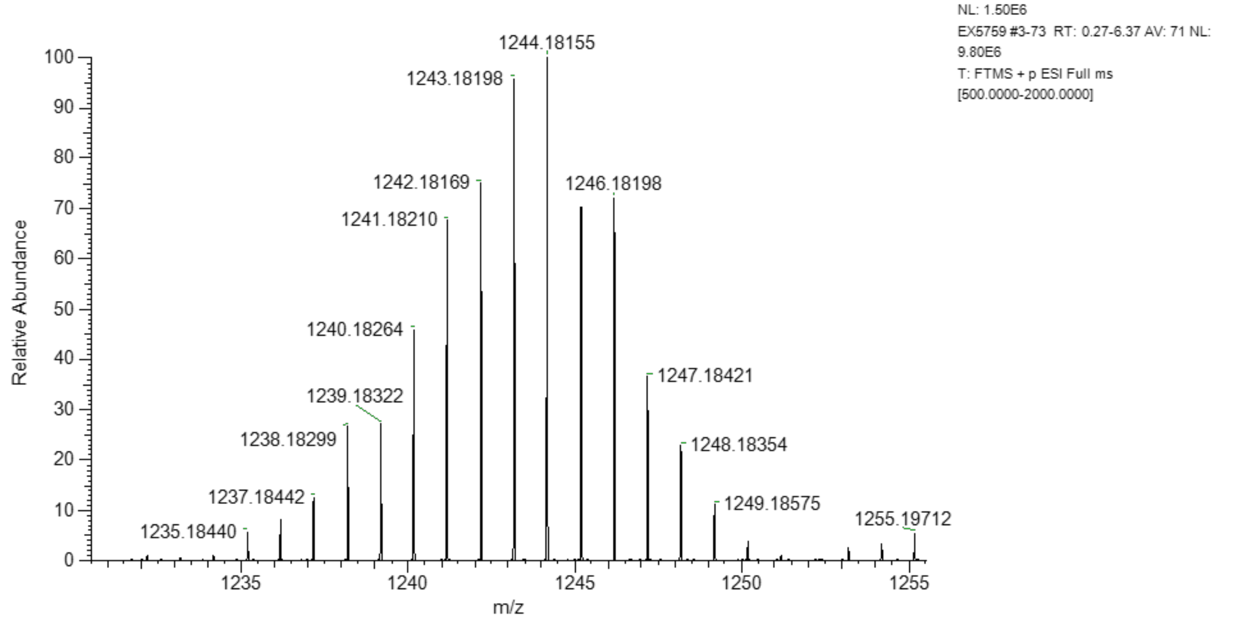

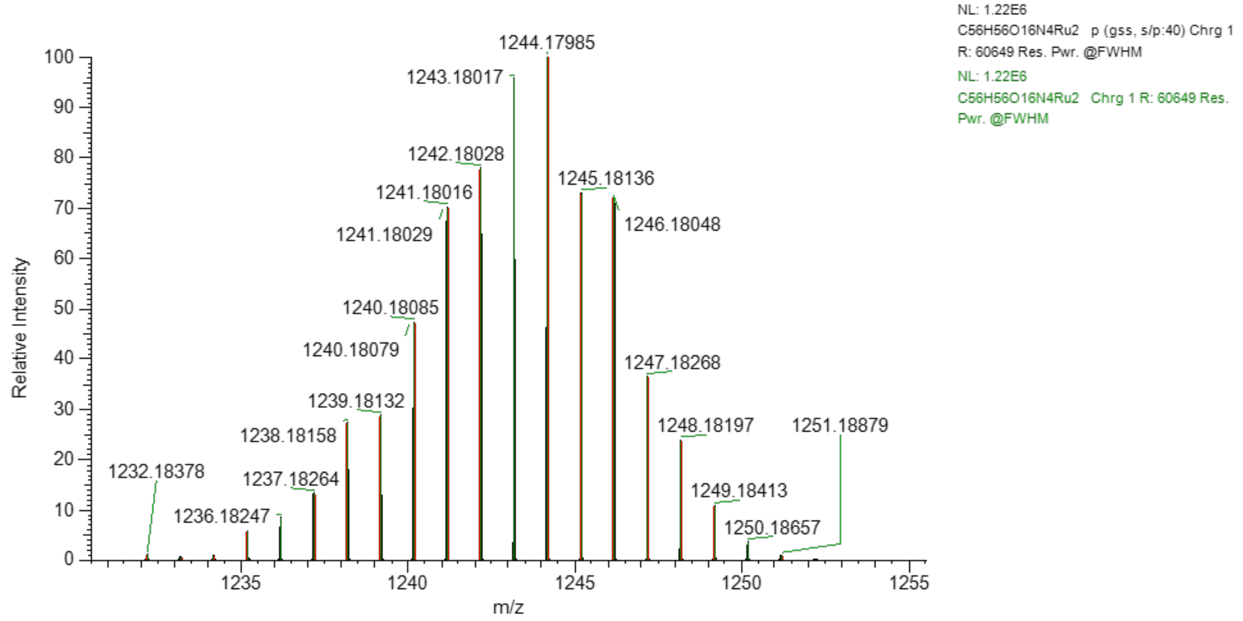

Ru_2(*S*-PTTL)$_4$BAr^F (5-BAr^F) (Top, observed. Bottom, simulated)

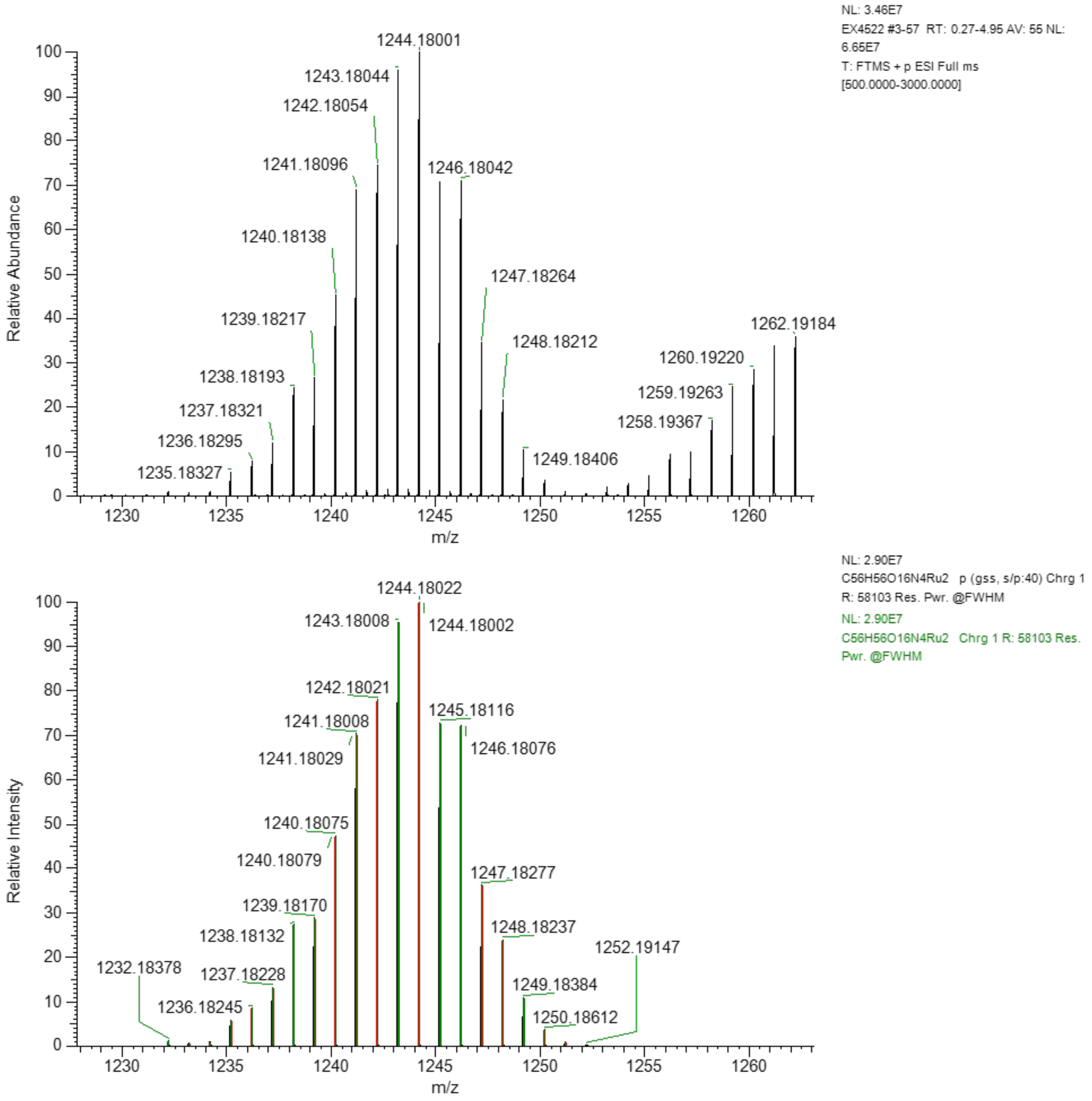

BAr^F Counterion (Top, observed. Bottom, simulated)

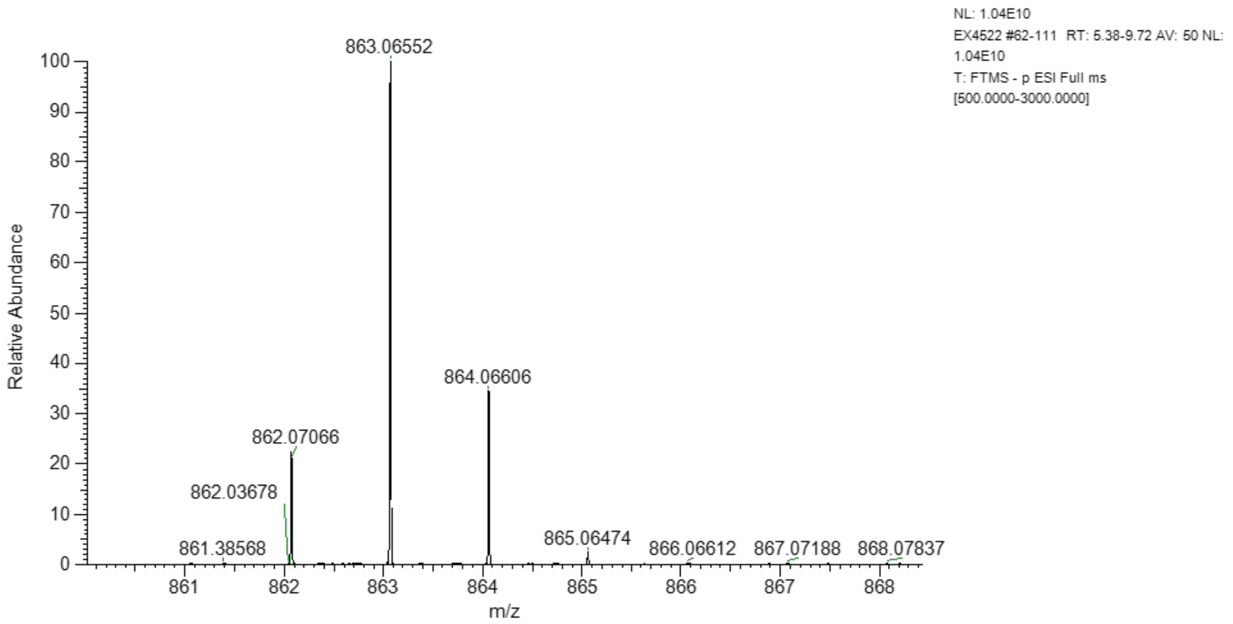

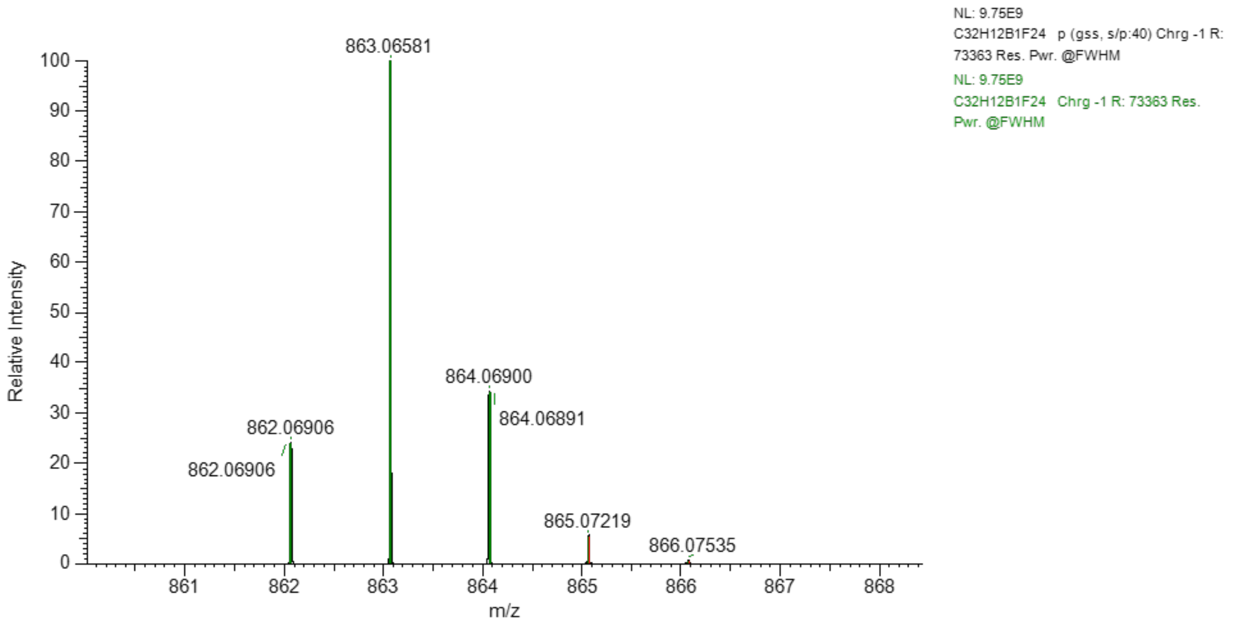

Ru_2(*S*-PTAD)$_4$Cl (6-Cl) (Top, observed. Bottom, simulated)

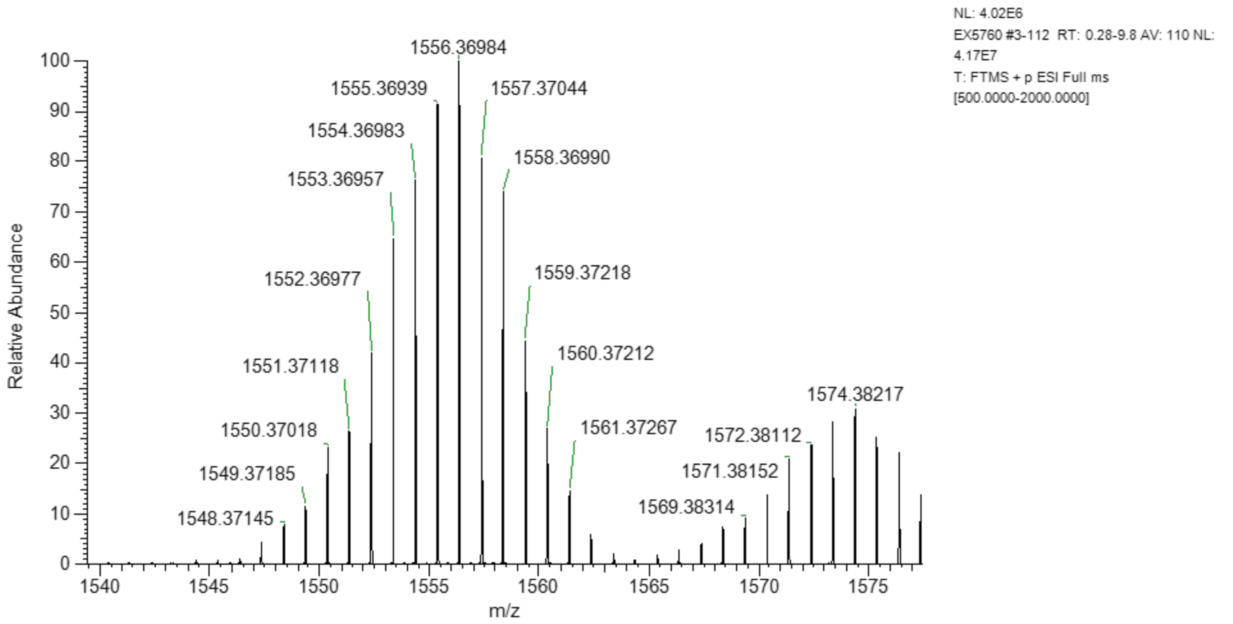

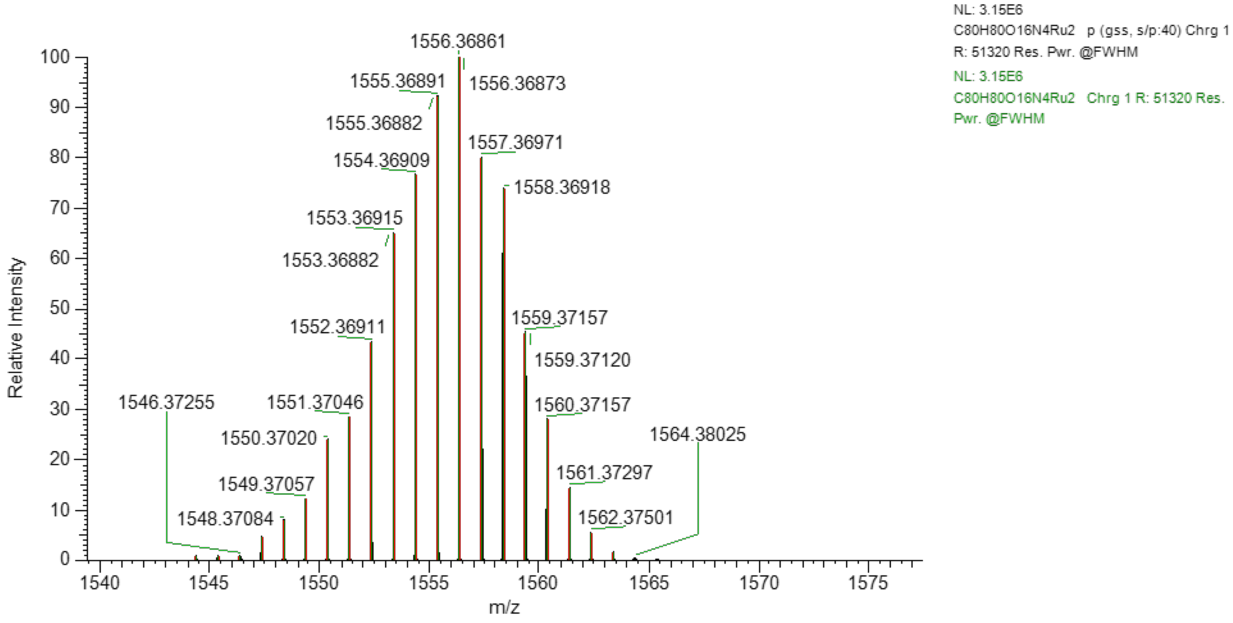

Ru_2(*S*-PTAD)$_4$BAr^F (6-BAr^F) (Top, observed. Bottom, simulated)

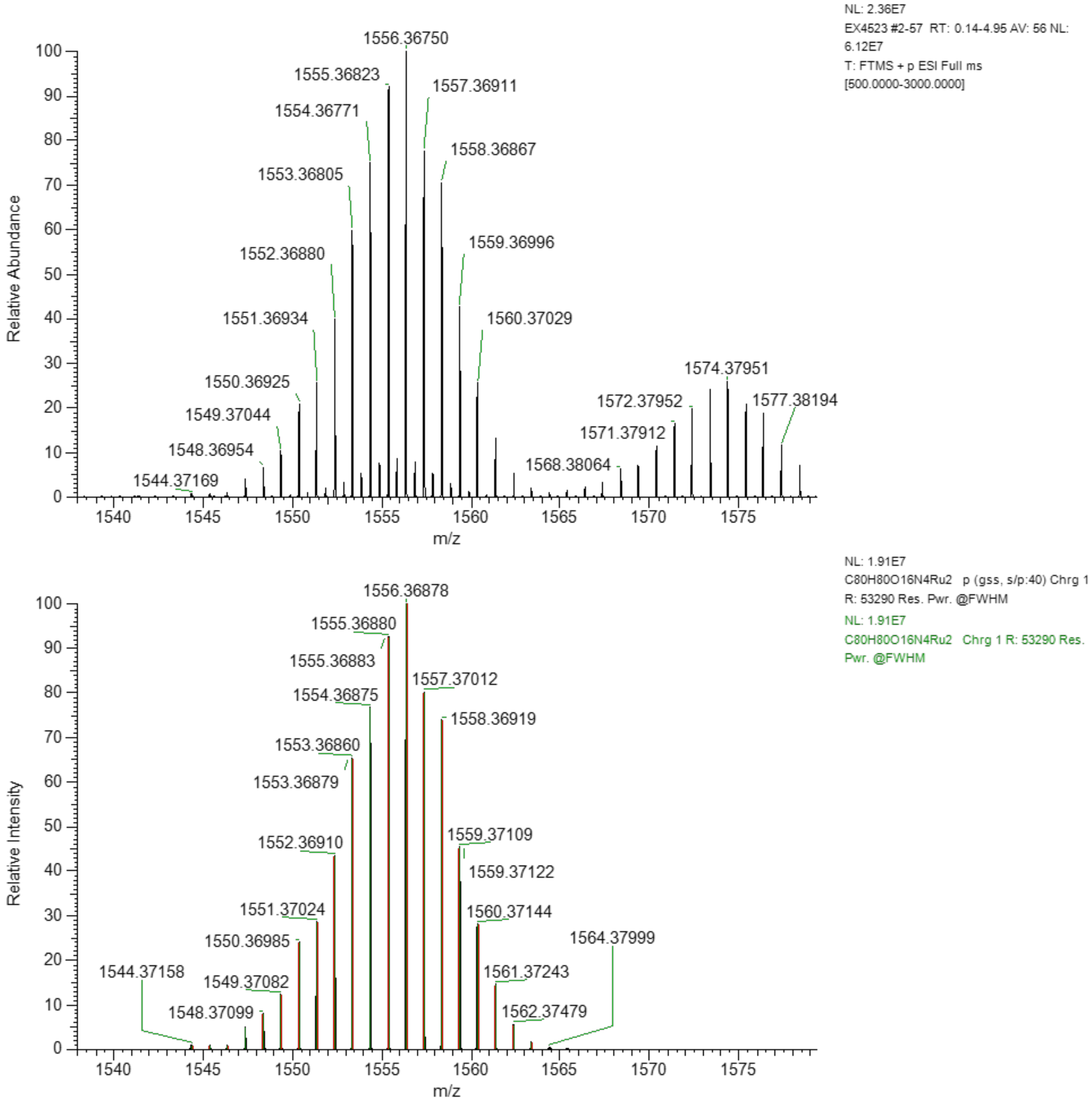

BAr^F Counterion (Top, observed. Bottom, simulated)

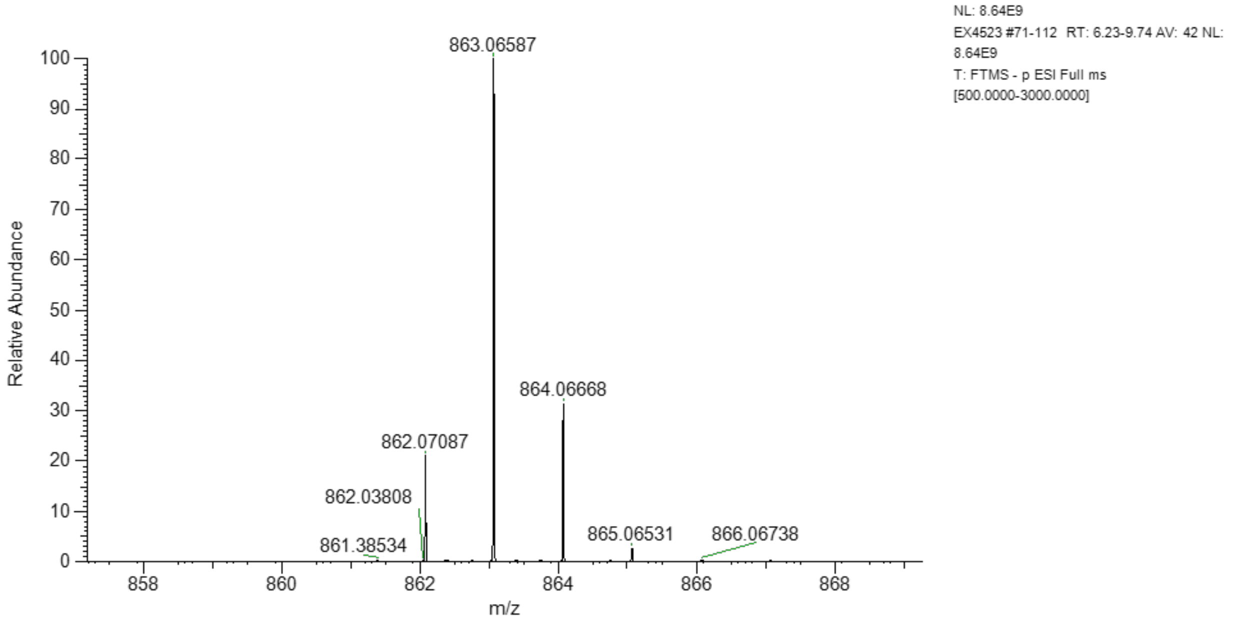

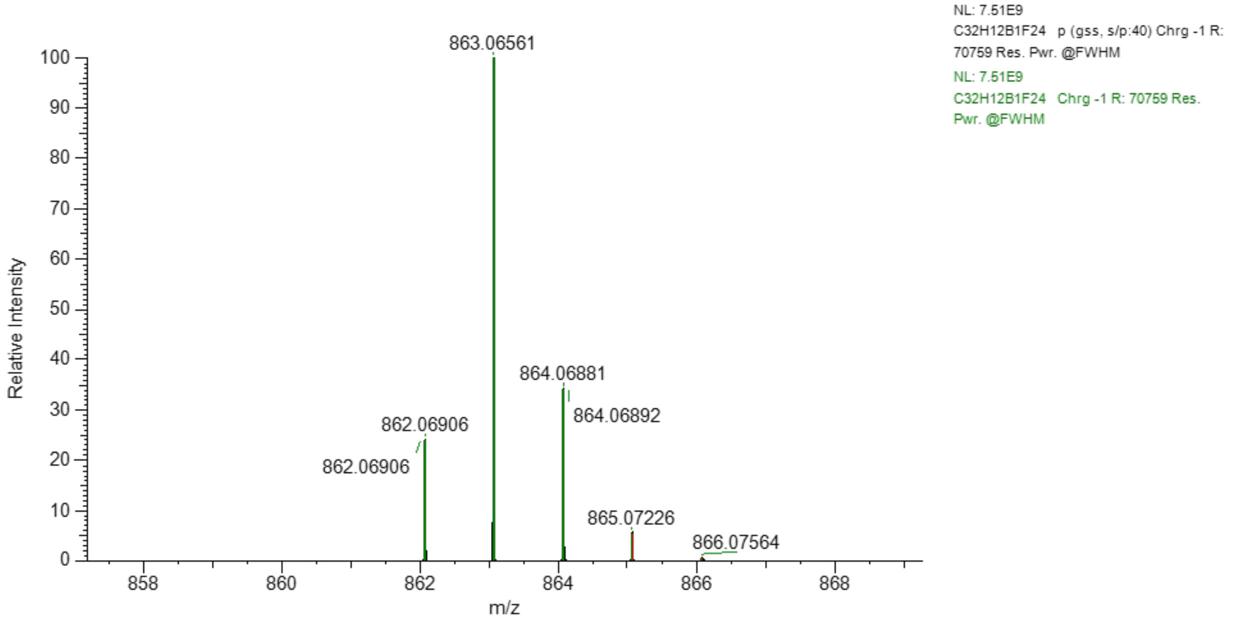

Ru_2(*S*-TCPTAD)$_4$Cl (7-Cl) (Top, observed. Bottom, simulated)

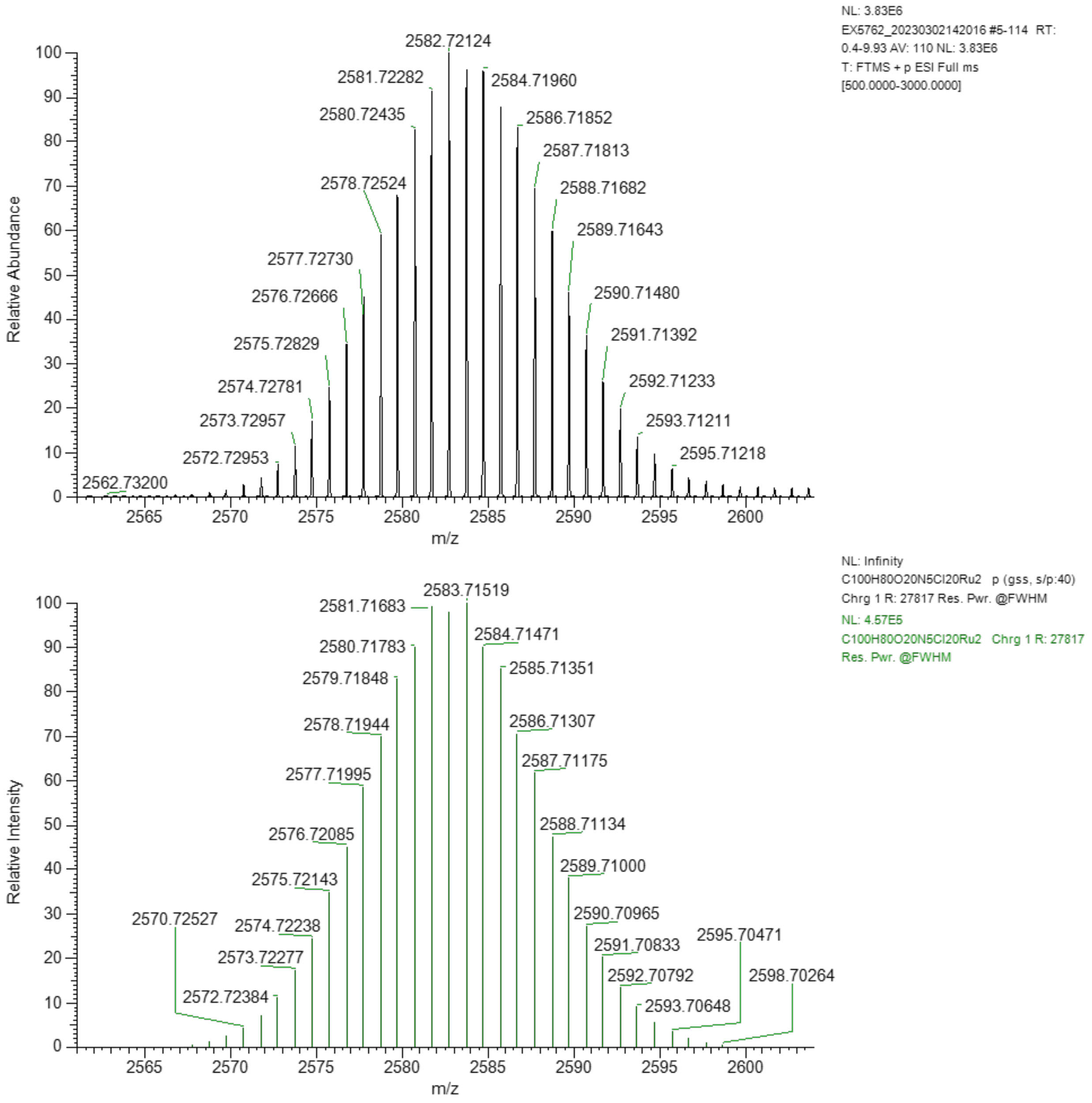

Ru$_2$(*S*-TCPTAD)$_4$BAr^F (7-BAr^F) (Top, observed. Bottom, simulated)

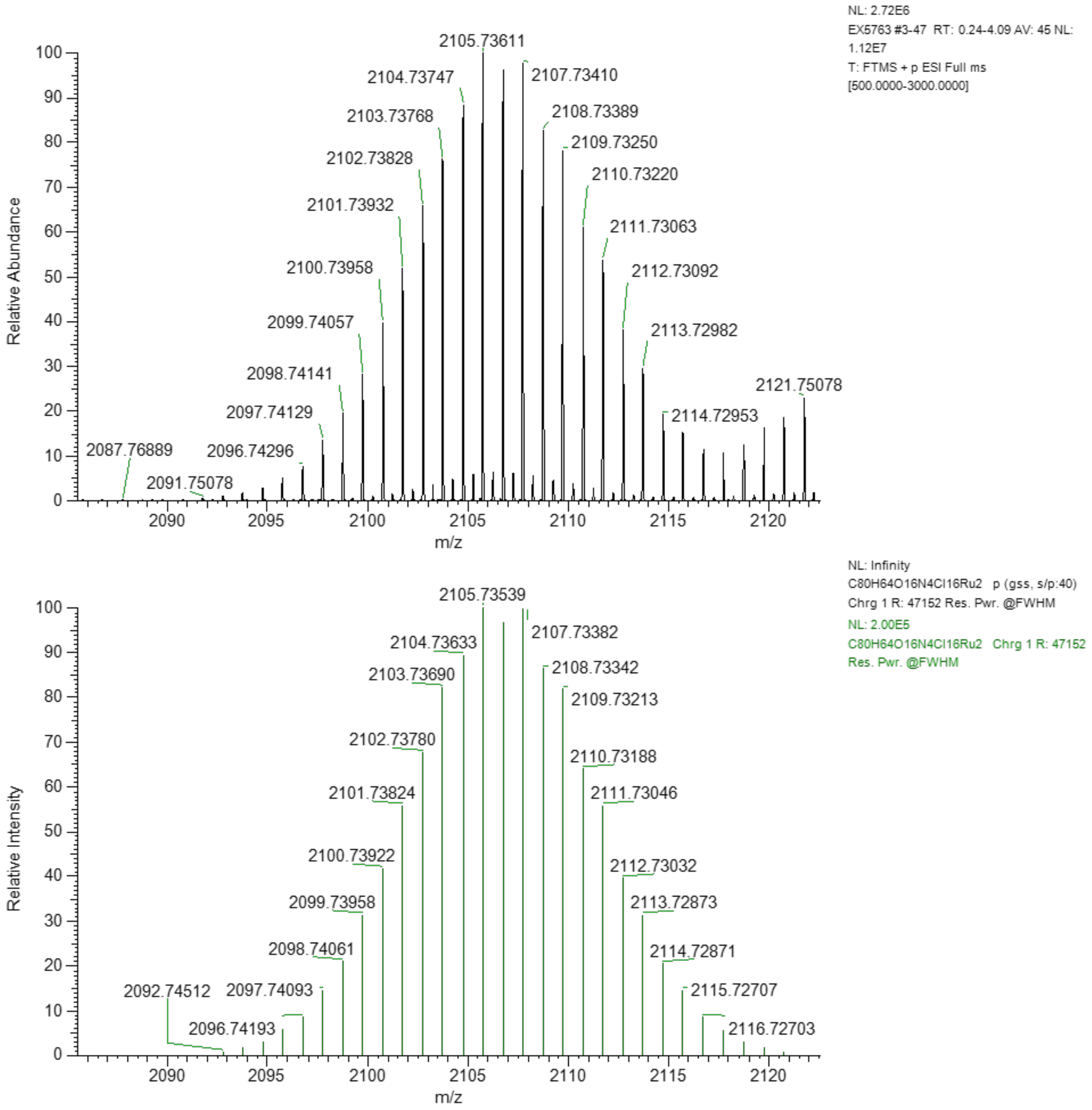

BAr^F Counterion (Top, observed. Bottom, simulated)

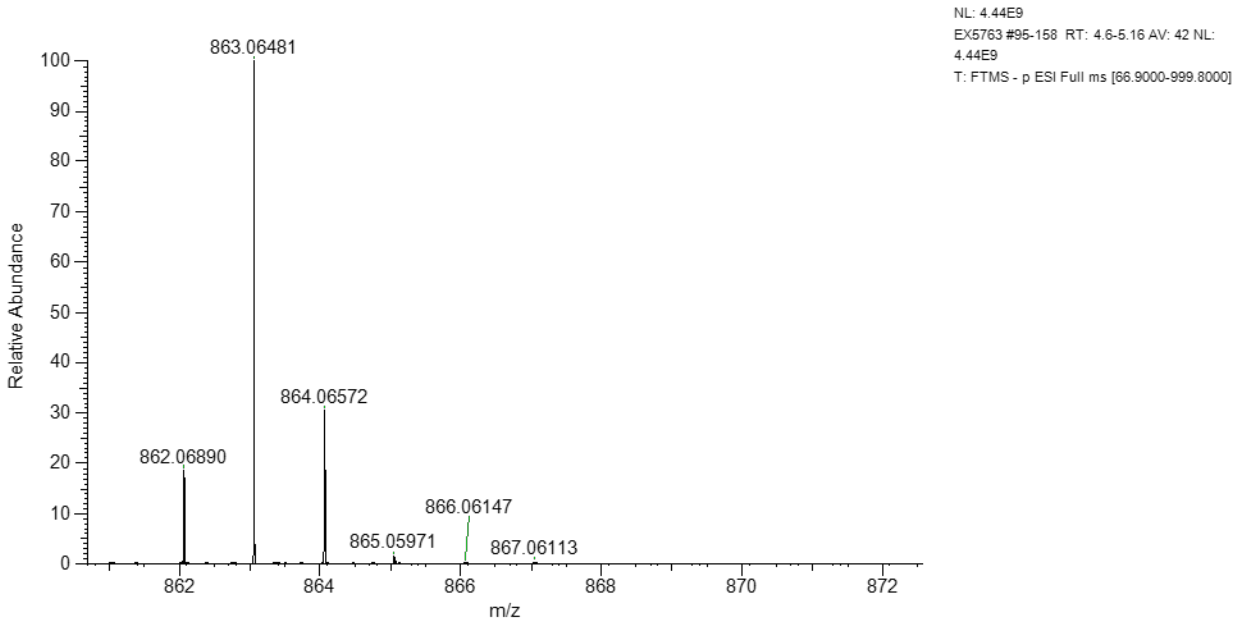

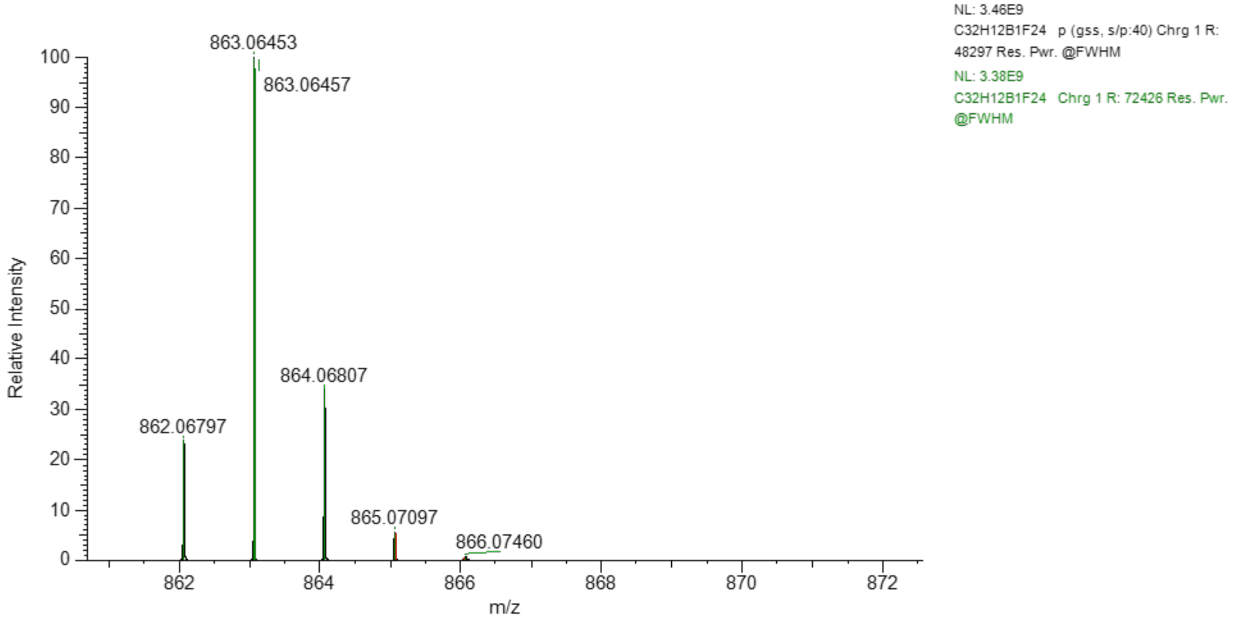

Ru$_2$(*S*-NTTL)$_4$Cl (8-Cl) (Top, observed. Bottom, simulated)

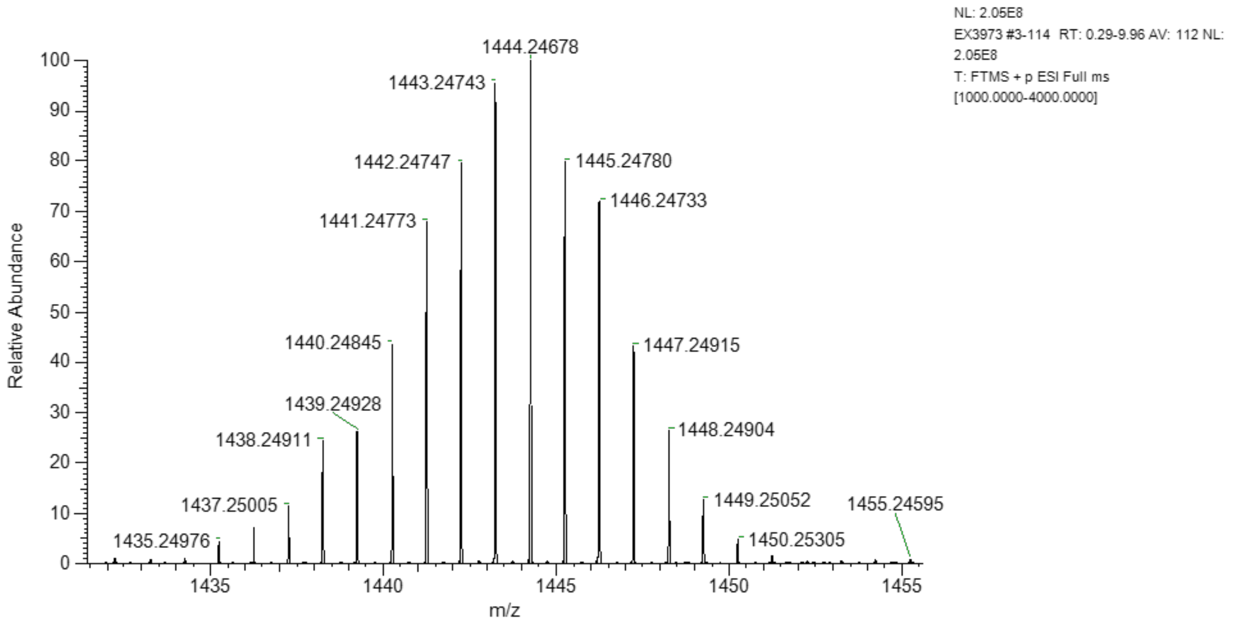

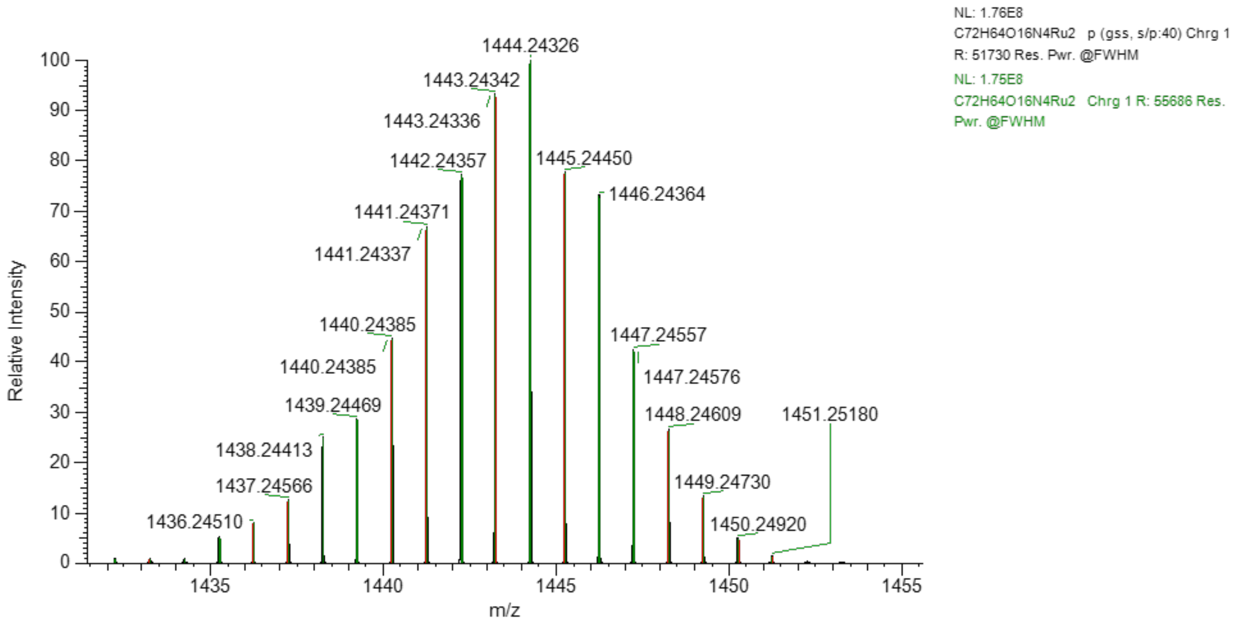

Ru_2(*S*-NTTL)$_4$BAr^F (8-BAr^F) (Top, observed. Bottom, simulated)

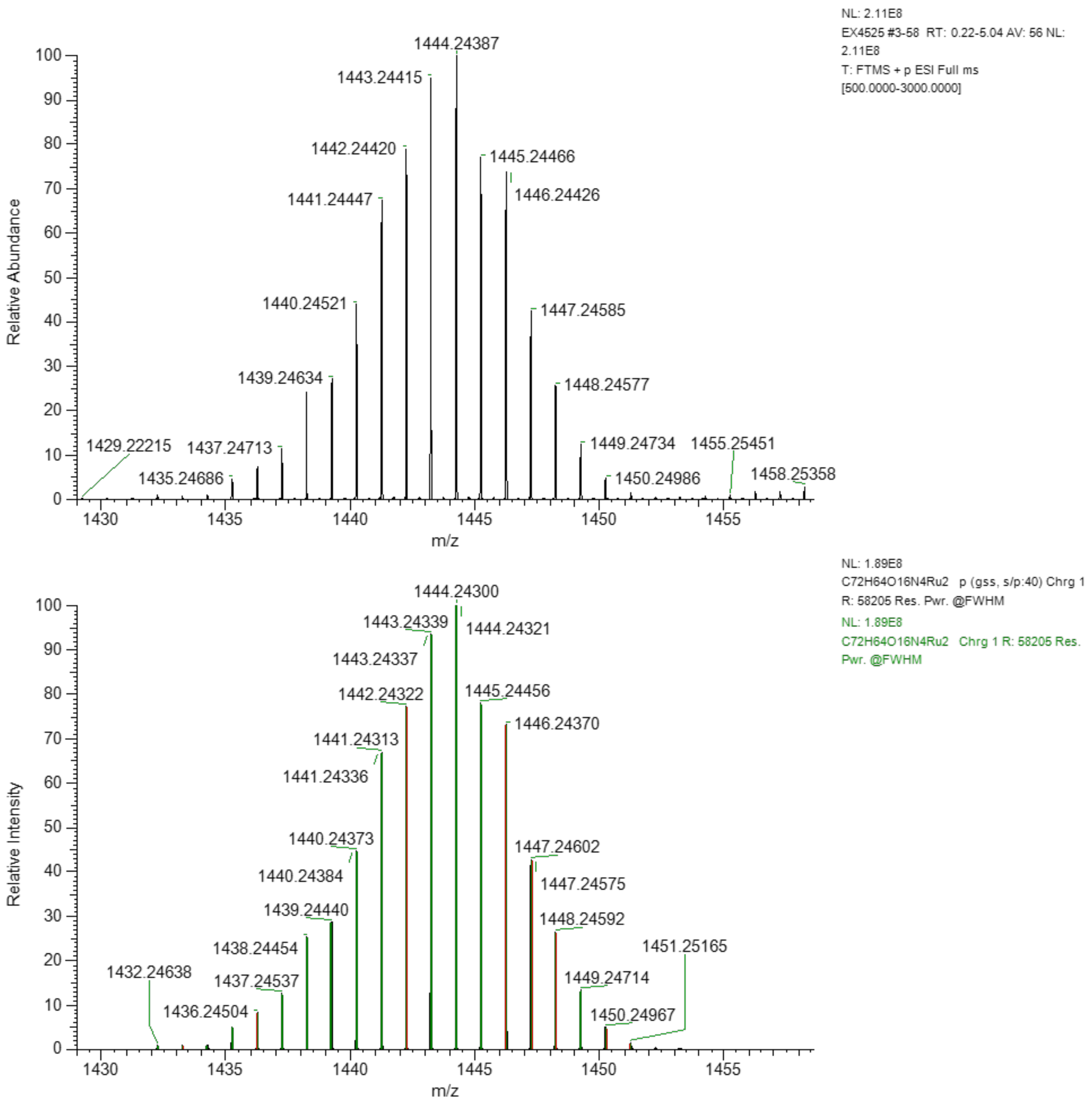

BAr^F Counterion (Top, observed. Bottom, simulated)

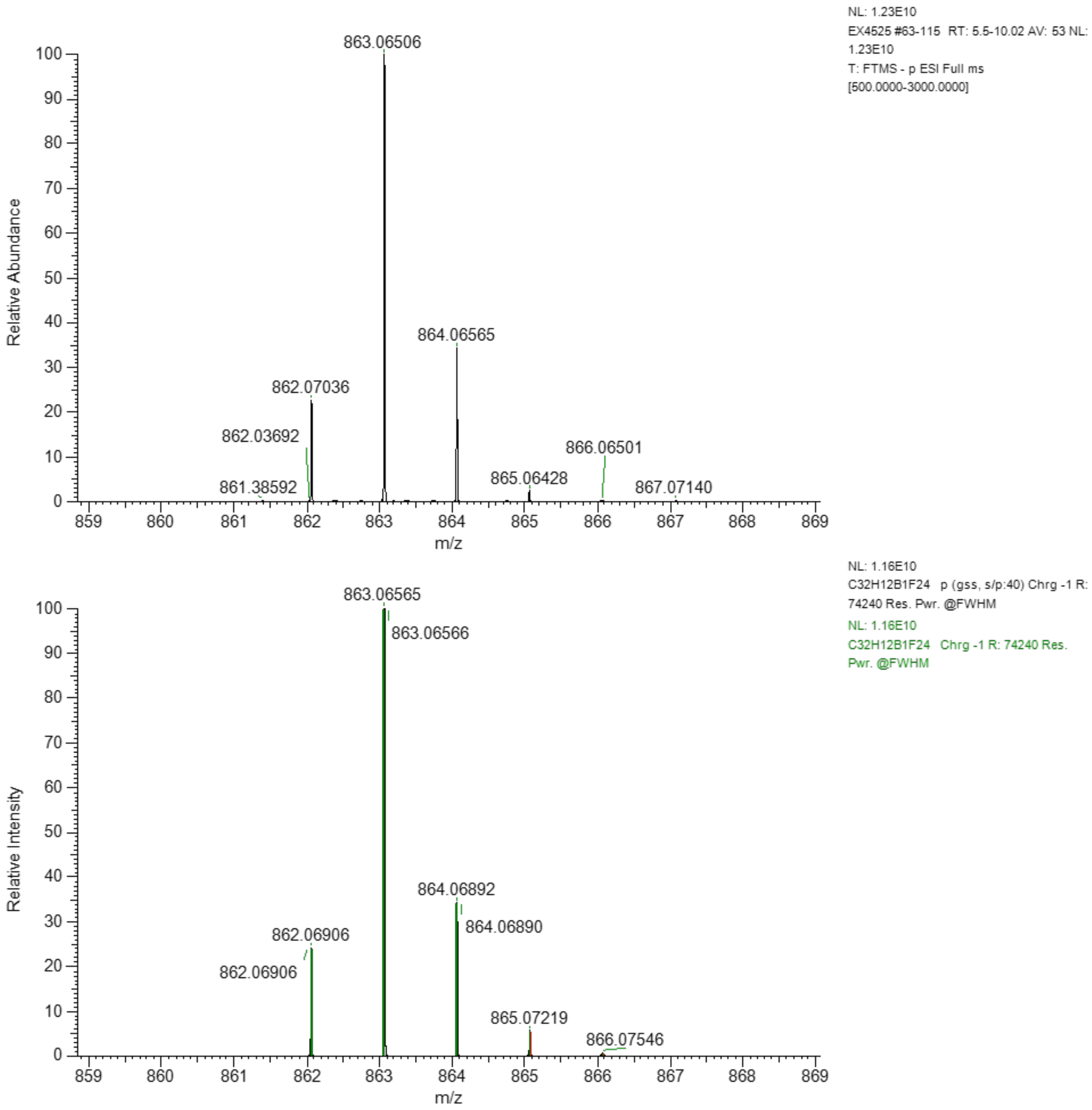

General Procedure for copper-catalyzed cyclopropanation:

In a flame-dried, 16ml vial, equipped with a magnetic stir bar and 4Å activated molecular sieves, Cu_2(S-TPPTTL)$_4$ (24 mg, 9.9 μmol, 5.0mol %) and styrene (52 mg, 0.50 mmol, 5.0 equiv columned through a small silica plug immediately prior to reaction) were added under an inert N2 atmosphere. The vial contents were then suspended in dry DCM (1.0 ml) at room temperature. Then 2,2,2-trichloroethyl 2-(4-bromophenyl)-2-diazoacetate (37 mg, 0.10 mmol, 1.0 equiv) was dissolved in dry DCM solvent (1 ml) under argon and added dropwise to the reaction mixture via syringe. The solution was stirred overnight at room temperature and reaction progress was monitored by IR (disappearance of stretch at 2100 cm-1 signifies reaction completion). For catalyst which was dried under high-vac for several weeks, the reaction was able to complete after 24h stirring at room temperature. For catalyst that was directly used from the crystallization bearing MeCN axial ligands, no reaction occurred after 24h at Rt. In this case, the reaction was heated to 40°C and stirred overnight, after which time it had become green in coloration and the reaction had finished. Upon reaction completion, the solution was passed through a celite filter to remove molecular sieves and the solvent was removed in vacuo. The crude residue was purified based on Rf by flash column chromatography (TLC developed in 5% EtOAc/hexanes, Rf: styrene=0.60 non-CAM stain active, cyclopropane product: 0.45 as a CAM stain active dark blue spot). Pure product fractions were combined, and solvent was evaporated to yield a white crystalline solid. In all cases the material obtained was racemic by chiral HPLC.

General Procedure for cobalt-catalyzed cyclopropanation:

To a flame dried vial equipped with a stir bar and 4Å MS under inert atmosphere was added catalyst (10mol %) and substrate (0.5 mmol, 2.5 equiv) which was subsequently dissolved in 2 mL of DCM. Then, the diazo compound (0.20 mmol, 1.0 equiv) was dissolved in 2 mL of DCM and added to the reaction vial over a period of 2 h using a syringe pump. The reaction was run at room temperature or 40 °C for 18 h. Once completed the reaction solution was passed through a small silica plug to remove cobalt catalyst, concentrated in vacuo, and purified through flash chromatography (0-18% Hexanes/diethyl ether) to afford the desired product.

React IR Experiments

General Procedure for react IR Experiments:
An oven dried three-neck round bottom flask equipped with a stir bar and 4Å MS was fitted to the React IR 45m probe and backfilled with nitrogen 3 times. Then, DCM (11 mL) was added to the flask and was equilibrated for 15 minutes. Styrene (xx equiv) and aryldiazoacetate (0.600 mmol) were added to the flask sequentially and the flask was left to equilibrate for 15 min. The React IR was set to monitor the diazo stretching vibration at 2300 cm^{-1}. After the equilibration, the catalysts (xxmol %) was dissolved in 1 mL of DCM and added to the reaction flask in one portion. After completion of reaction (monitored by the disappearance of the peak at 2300 cm^{-1}), the reaction was concentrated and purified through flash chromatography (0% hexanes/diethyl ether, 0-18% hexanes/diethyl ether) to afford a crystalline solid.

HPLC Chromatographs

HPLC chromatographs for copper-catalyzed cyclopropanation:

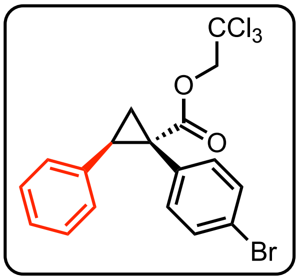

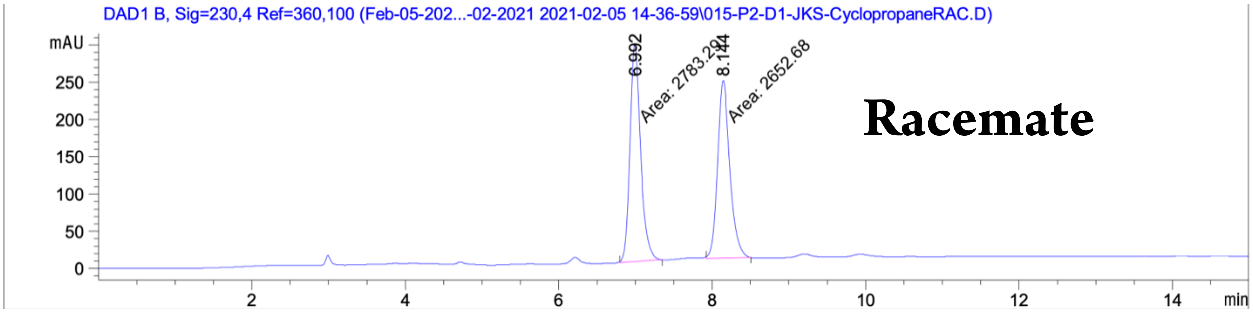

Copper catalyzed cyclopropanation, 24°C from dried catalyst

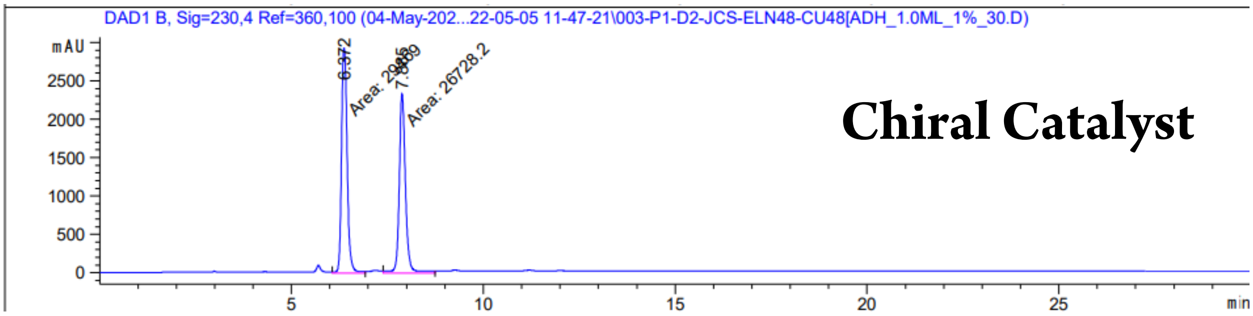

Signal 2: DAD1 B, Sig=230,4 Ref=360,100

Peak #	RetTime [min]	Type	Width [min]	Area [mAU*s]	Height [mAU]	Area %
1	6.372	MM	0.1675	2.94690e4	2931.42407	52.4385
2	7.885	MM	0.1908	2.67282e4	2334.88330	47.5615

Copper catalyzed cyclopropanation, 40°C from MeCN crystallized catalyst

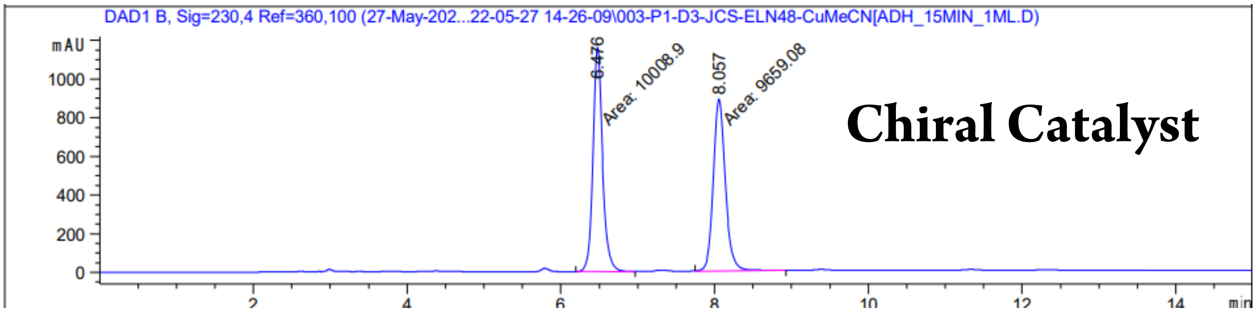

Signal 2: DAD1 B, Sig=230,4 Ref=360,100

Peak #	RetTime [min]	Type	Width [min]	Area [mAU*s]	Height [mAU]	Area %
1	6.476	MM	0.1438	1.00089e4	1159.90820	50.8894
2	8.057	MM	0.1811	9659.08398	889.05890	49.1106
Totals :				1.96680e4	2048.96710	

HPLC chromatographs for cobalt-catalyzed cyclopropanation

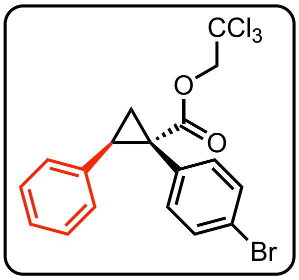

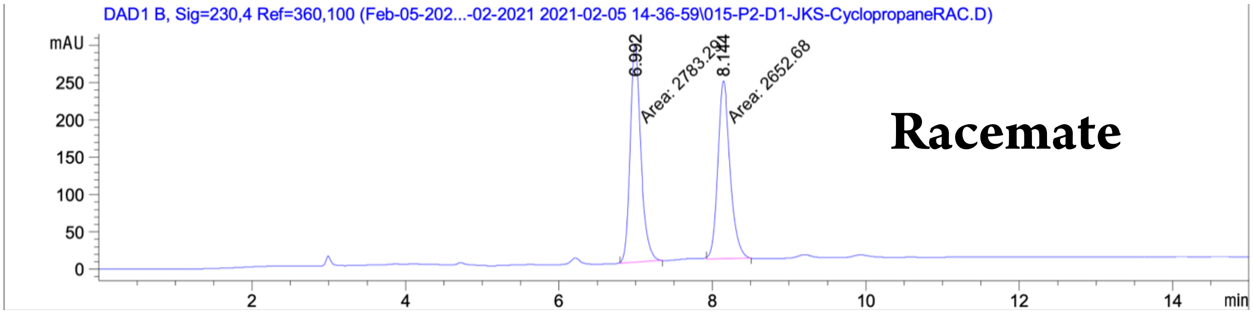

Cobalt-catalyzed reaction at 25 °C

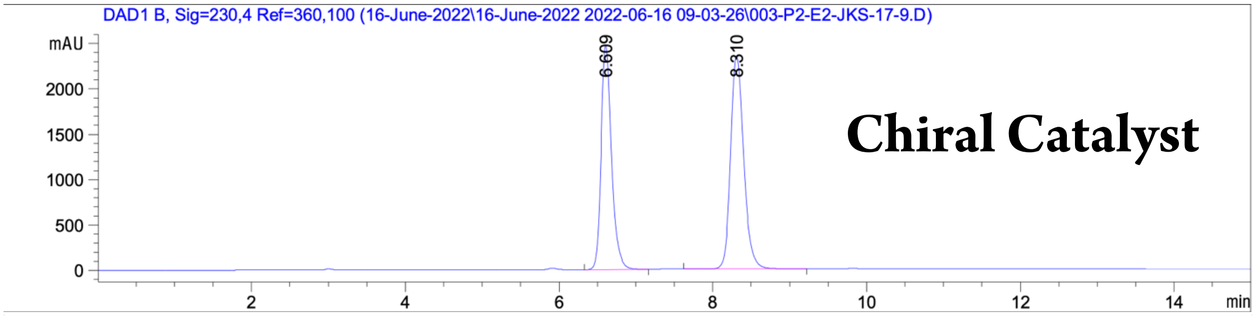

Signal 2: DAD1 B, Sig=230,4 Ref=360,100

Peak #	RetTime [min]	Type	Width [min]	Area [mAU*s]	Height [mAU]	Area %
1	6.609	BB	0.1436	2.32320e4	2472.58936	45.7450
2	8.310	BB	0.1808	2.75539e4	2350.05371	54.2550
Totals :				5.07859e4	4822.64307	

Cobalt-catalyzed reaction at 40 °C

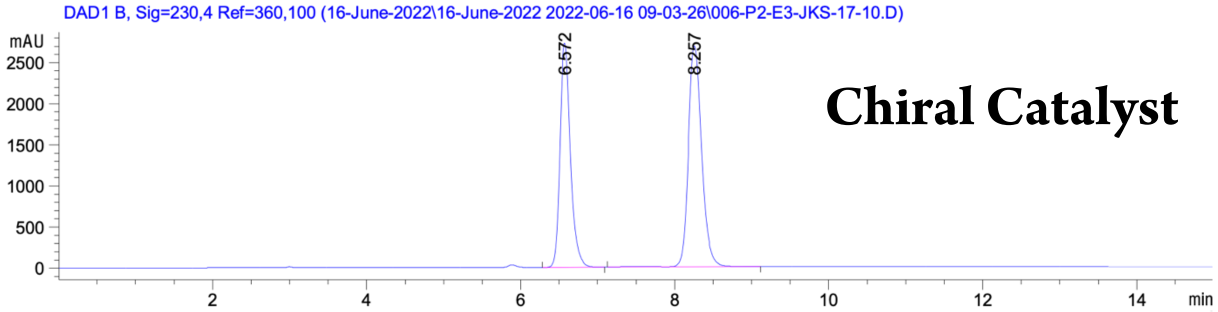

Signal 2: DAD1 B, Sig=230,4 Ref=360,100

Peak #	RetTime [min]	Type	Width [min]	Area [mAU*s]	Height [mAU]	Area %
1	6.572	BB	0.1462	2.57793e4	2729.63623	44.4686
2	8.257	VB R	0.1831	3.21926e4	2686.88770	55.5314
Totals :				5.79720e4	5416.52393	

Chromatographs for catalyst screen for cyclopropanation of styrene

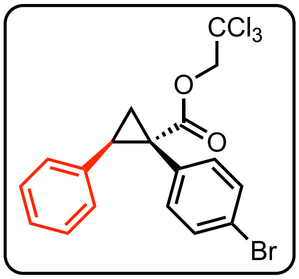

Racemate

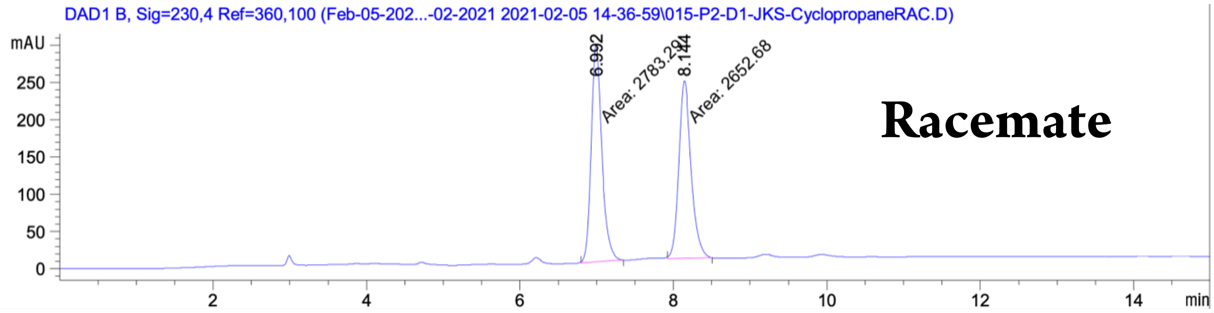

Signal 2: DAD1 B, Sig=230,4 Ref=360,100

Peak #	RetTime [min]	Type	Width [min]	Area [mAU*s]	Height [mAU]	Area %
1	6.992	MM	0.1590	2783.28638	291.76401	51.2013
2	8.144	MM	0.1852	2652.68018	238.66949	48.7987

Reaction with **4-Cl**

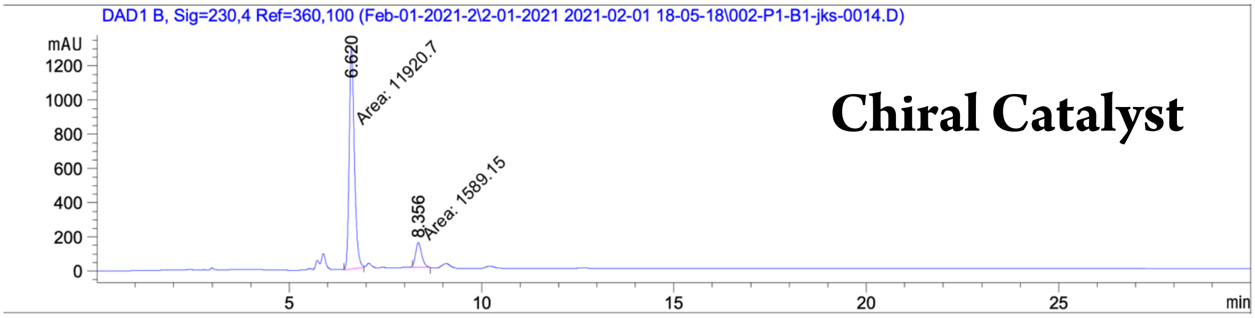

Signal 2: DAD1 B, Sig=230,4 Ref=360,100

Peak #	RetTime [min]	Type	Width [min]	Area [mAU*s]	Height [mAU]	Area %
1	6.620	MM	0.1522	1.19207e4	1305.00964	88.2371
2	8.356	MM	0.1831	1589.15454	144.62219	11.7629
Totals :				1.35099e4	1449.63184	

Reaction with **4-BAr**[F]

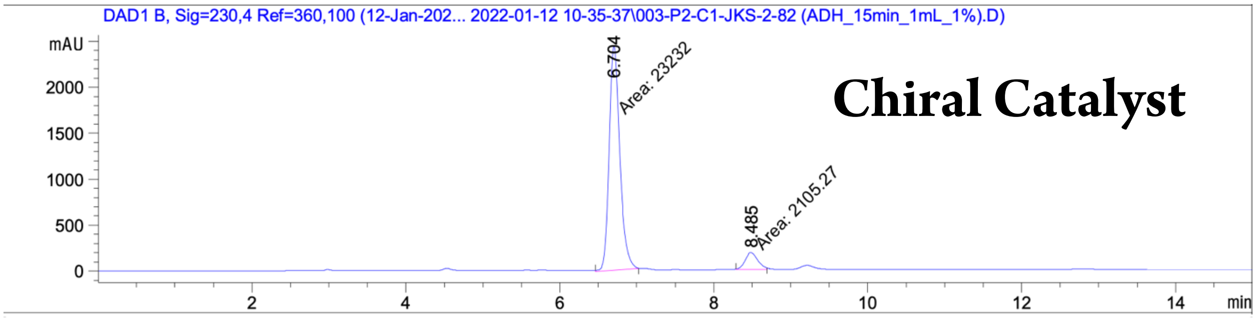

Signal 2: DAD1 B, Sig=230,4 Ref=360,100

Peak #	RetTime [min]	Type	Width [min]	Area [mAU*s]	Height [mAU]	Area %
1	6.704	MM	0.1586	2.32320e4	2441.86987	91.6910
2	8.485	MM	0.1902	2105.26636	184.43390	8.3090
Totals :				2.53373e4	2626.30377	

Reaction with **5-Cl**

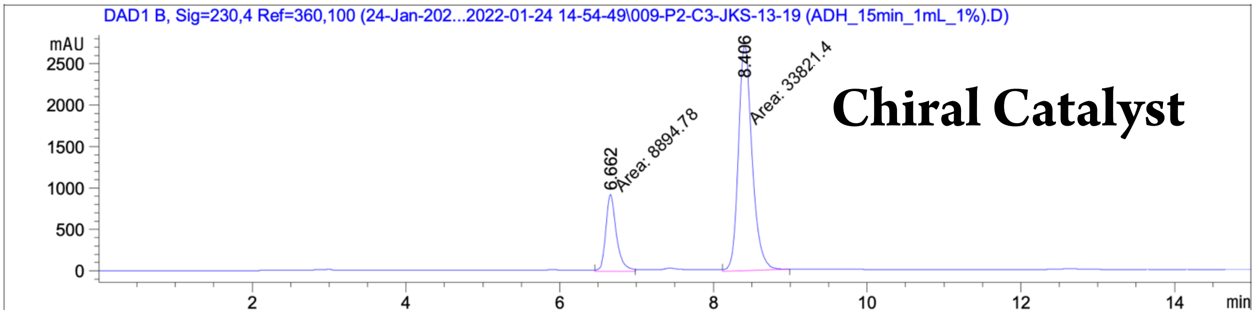

Signal 2: DAD1 B, Sig=230,4 Ref=360,100

Peak #	RetTime [min]	Type	Width [min]	Area [mAU*s]	Height [mAU]	Area %
1	6.662	MM	0.1601	8894.77539	925.95435	20.8230
2	8.406	MM	0.2079	3.38214e4	2711.37109	79.1770
Totals :				4.27162e4	3637.32544	

Reaction with **5-BAr^F^**

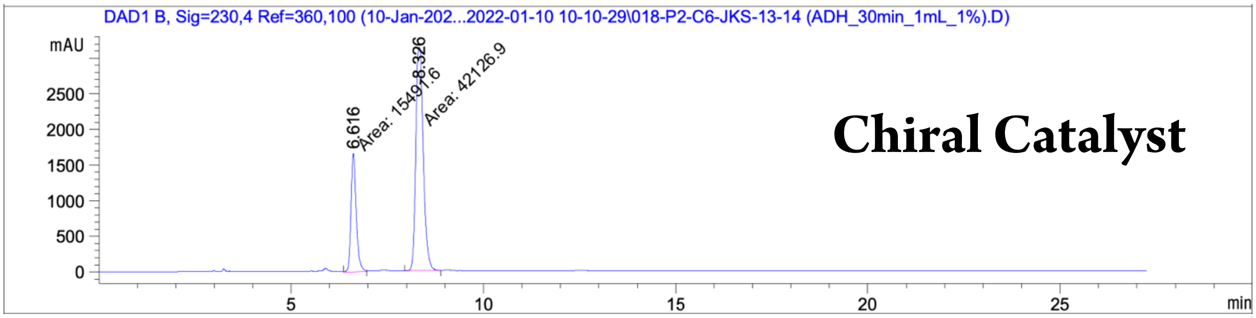

Signal 2: DAD1 B, Sig=230,4 Ref=360,100

Peak #	RetTime [min]	Type	Width [min]	Area [mAU*s]	Height [mAU]	Area %
1	6.616	MM	0.1553	1.54916e4	1662.19495	26.8865
2	8.326	MM	0.2245	4.21269e4	3128.14209	73.1135
Totals :				5.76185e4	4790.33704	

Reaction with **6-Cl**

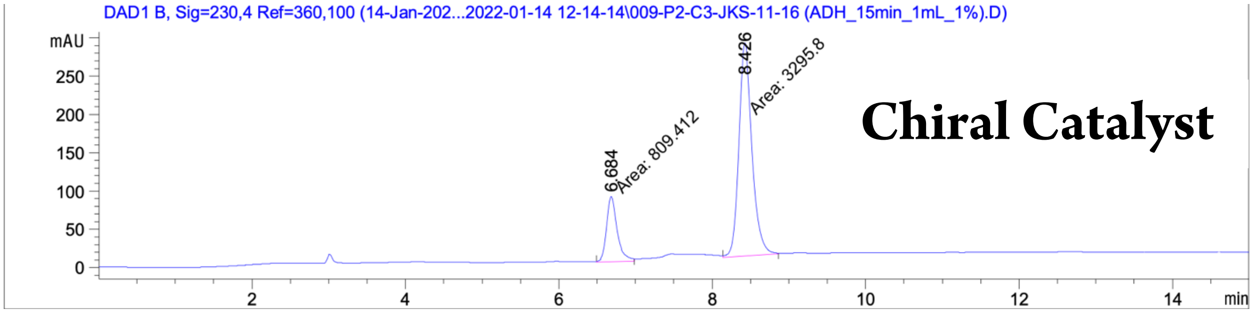

Signal 2: DAD1 B, Sig=230,4 Ref=360,100

Peak #	RetTime [min]	Type	Width [min]	Area [mAU*s]	Height [mAU]	Area %
1	6.684	MM	0.1585	809.41248	85.12662	19.7167
2	8.426	MM	0.1973	3295.80225	278.45493	80.2833
Totals :				4105.21472	363.58154	

Reaction with **6-BAr^F^**

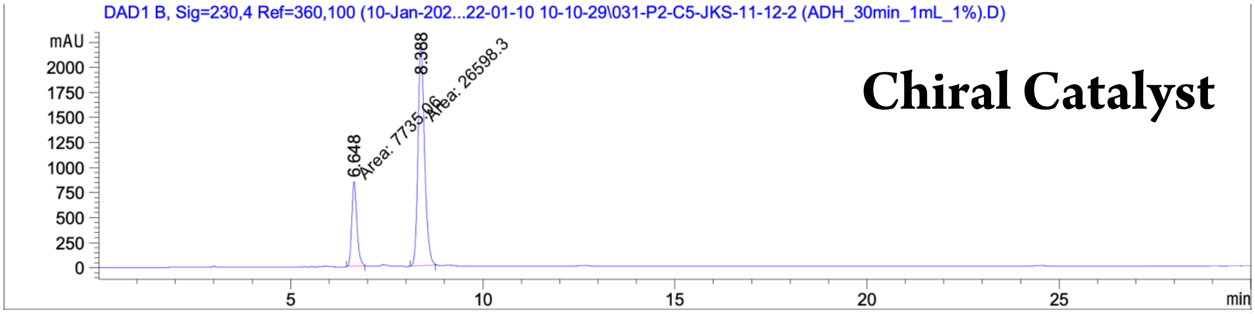

Signal 2: DAD1 B, Sig=230,4 Ref=360,100

Peak #	RetTime [min]	Type	Width [min]	Area [mAU*s]	Height [mAU]	Area %
1	6.648	MM	0.1527	7735.06250	844.47351	22.5293
2	8.388	MM	0.1992	2.65983e4	2225.34326	77.4707
Totals :				3.43334e4	3069.81677	

Reaction with **7-Cl**

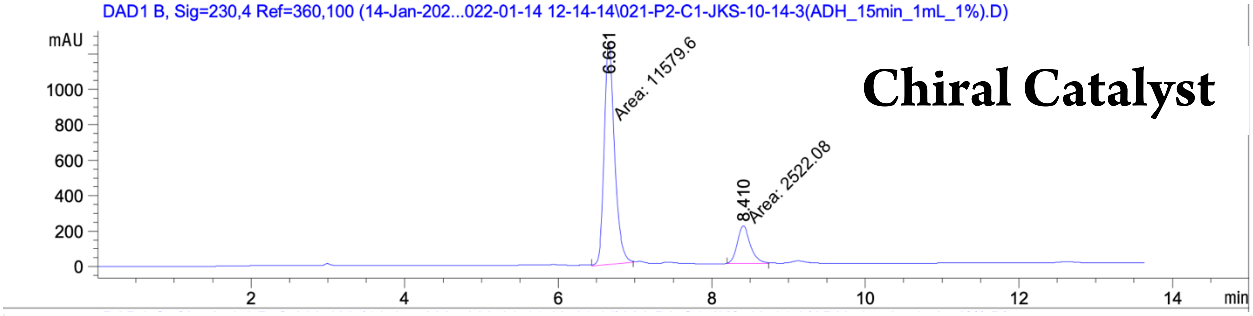

Signal 2: DAD1 B, Sig=230,4 Ref=360,100

Peak #	RetTime [min]	Type	Width [min]	Area [mAU*s]	Height [mAU]	Area %
1	6.661	MM	0.1535	1.15796e4	1257.49841	82.1150
2	8.410	MM	0.1975	2522.08228	212.81627	17.8850

Reaction with **7-BAr^F**

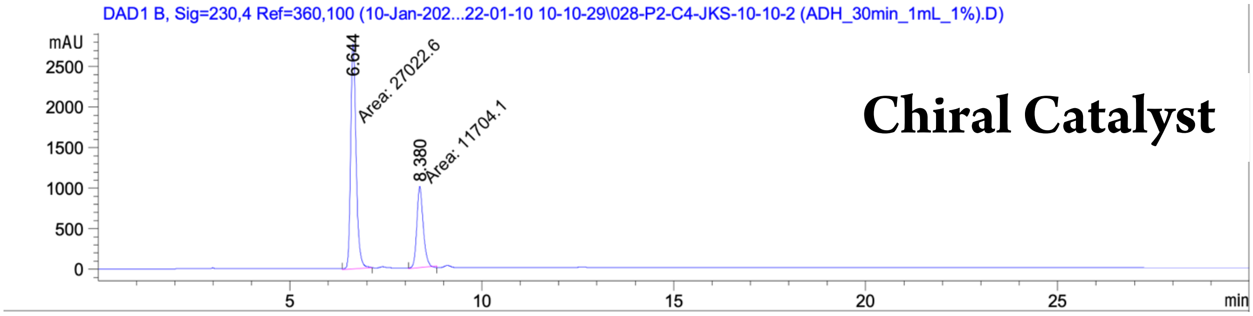

Signal 2: DAD1 B, Sig=230,4 Ref=360,100

Peak #	RetTime [min]	Type	Width [min]	Area [mAU*s]	Height [mAU]	Area %
1	6.644	MM	0.1621	2.70226e4	2777.73608	69.7776
2	8.380	MM	0.1940	1.17041e4	1005.47302	30.2224
Totals :				3.87267e4	3783.20911	

Reaction with **8-Cl**

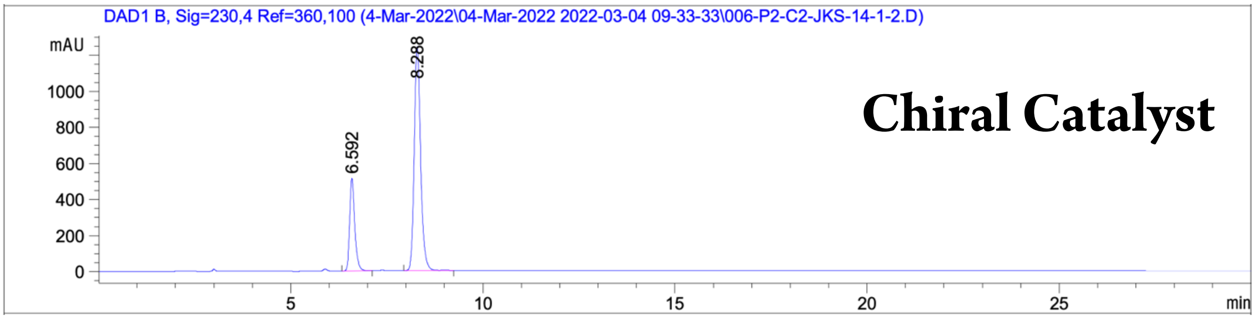

Signal 2: DAD1 B, Sig=230,4 Ref=360,100

Peak #	RetTime [min]	Type	Width [min]	Area [mAU*s]	Height [mAU]	Area %
1	6.592	BB	0.1364	4692.95605	514.33899	24.6941
2	8.288	BV R	0.1741	1.43114e4	1243.37061	75.3059
Totals :				1.90044e4	1757.70959	

Reaction with **8-BAr^F**

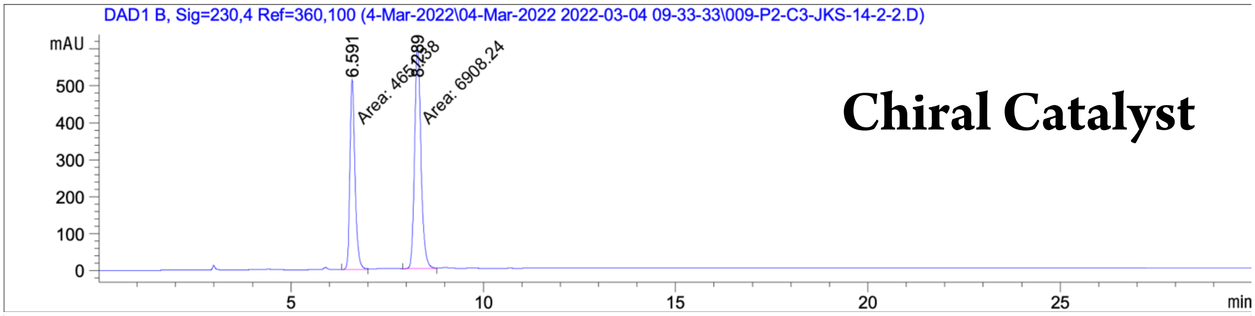

Signal 2: DAD1 B, Sig=230,4 Ref=360,100

Peak #	RetTime [min]	Type	Width [min]	Area [mAU*s]	Height [mAU]	Area %
1	6.591	MM	0.1507	4651.38281	514.31866	40.2382
2	8.289	MM	0.1907	6908.24219	603.70685	59.7618
Totals :				1.15596e4	1118.02551	

Reaction with **S18**

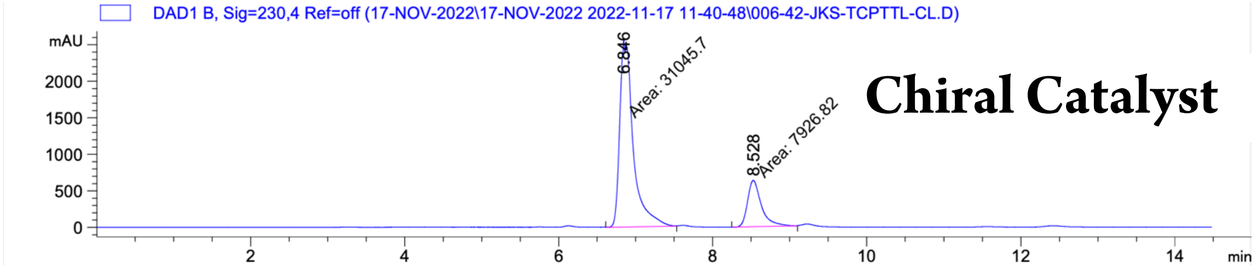

Signal 2: DAD1 B, Sig=230,4 Ref=off

Peak #	RetTime [min]	Type	Width [min]	Area [mAU*s]	Height [mAU]	Area %
1	6.846	MM	0.2024	3.10457e4	2555.91040	79.6605
2	8.528	MM	0.2077	7926.82373	636.00977	20.3395
Totals :				3.89725e4	3191.92017	

Reaction with **S19**

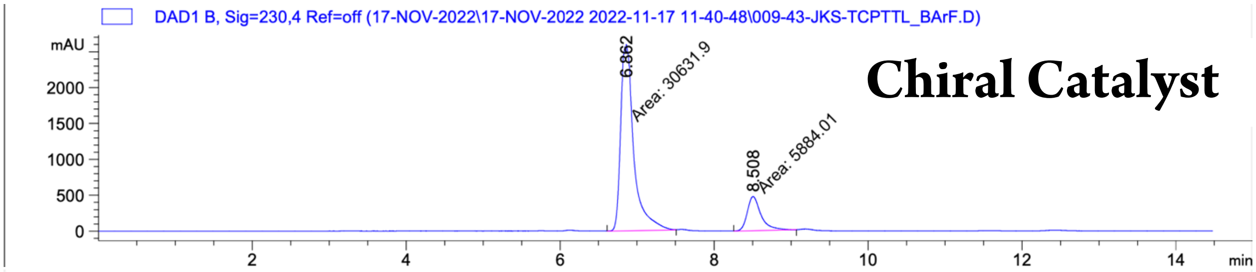

Signal 2: DAD1 B, Sig=230,4 Ref=off

Peak #	RetTime [min]	Type	Width [min]	Area [mAU*s]	Height [mAU]	Area %
1	6.862	MM	0.1965	3.06319e4	2598.30859	83.8864
2	8.508	MM	0.2059	5884.01465	476.25830	16.1136
Totals :				3.65159e4	3074.56689	

Chromatograph Substrate Scope for HPLC

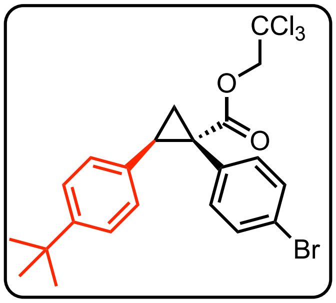

Compound **9**

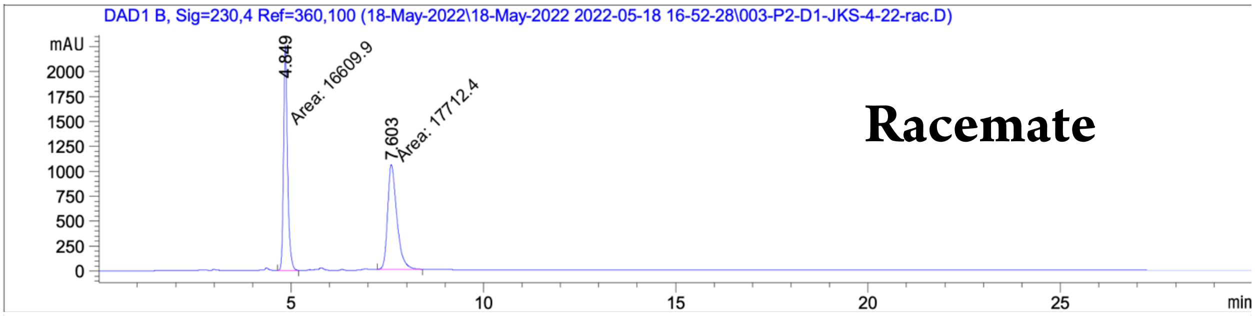


```
Signal 2: DAD1 B, Sig=230,4 Ref=360,100

Peak RetTime Type  Width      Area      Height      Area
  #   [min]        [min]    [mAU*s]     [mAU]        %
----|-------|----|-------|----------|----------|--------|
   1   4.849 MM    0.1230 1.66099e4  2250.49829  48.3940
   2   7.603 MM    0.2808 1.77124e4  1051.24963  51.6060
```

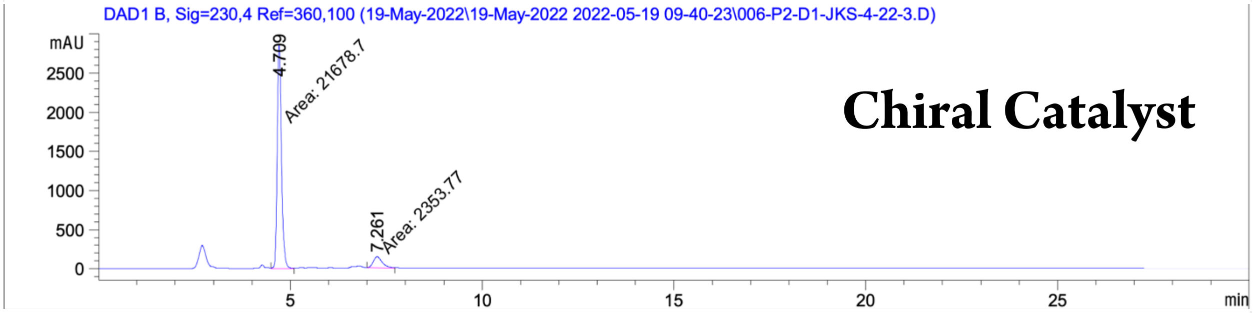


```
Signal 2: DAD1 B, Sig=230,4 Ref=360,100

Peak RetTime Type  Width      Area      Height      Area
  #   [min]        [min]    [mAU*s]     [mAU]        %
----|-------|----|-------|----------|----------|--------|
   1   4.709 MM    0.1262 2.16787e4  2863.48511  90.2059
   2   7.261 MM    0.2738 2353.76660  143.26881   9.7941

Totals :                  2.40325e4  3006.75392
```

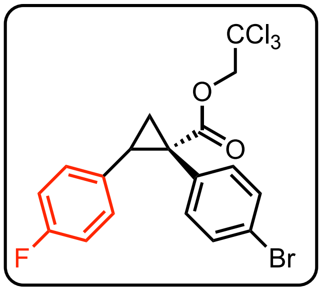

Compound **10**

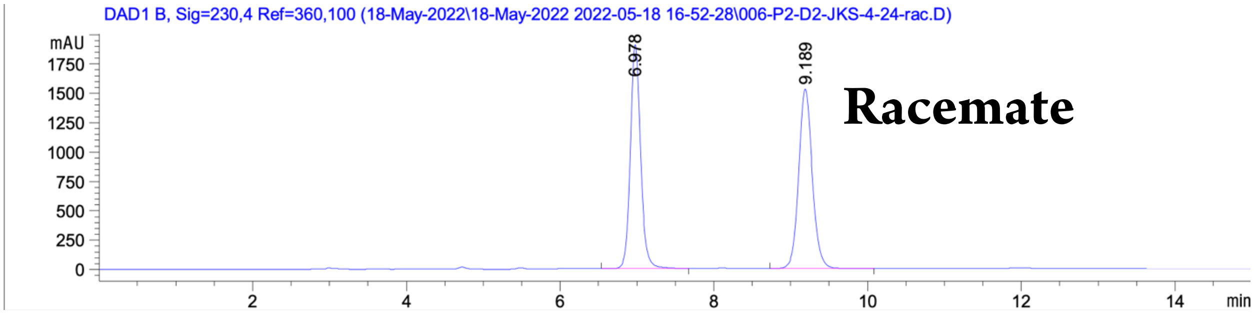

Signal 2: DAD1 B, Sig=230,4 Ref=360,100

Peak #	RetTime [min]	Type	Width [min]	Area [mAU*s]	Height [mAU]	Area %
1	6.978	VB R	0.1389	1.74849e4	1906.98328	48.2823
2	9.189	BB	0.1890	1.87290e4	1527.97461	51.7177

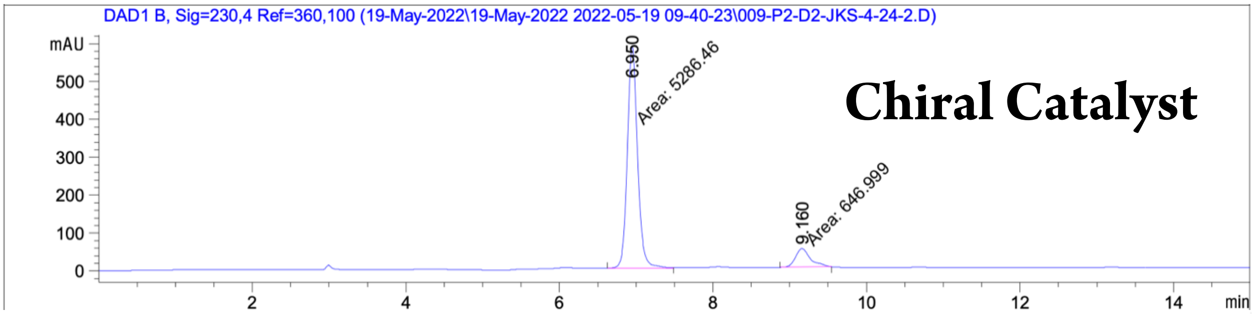

Signal 2: DAD1 B, Sig=230,4 Ref=360,100

Peak #	RetTime [min]	Type	Width [min]	Area [mAU*s]	Height [mAU]	Area %
1	6.950	MM	0.1502	5286.46191	586.70062	89.0958
2	9.160	MM	0.2222	646.99884	48.54053	10.9042

Totals : 5933.46075 635.24115

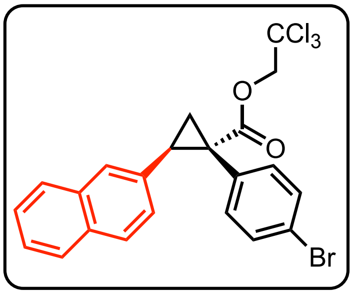

Compound **11**

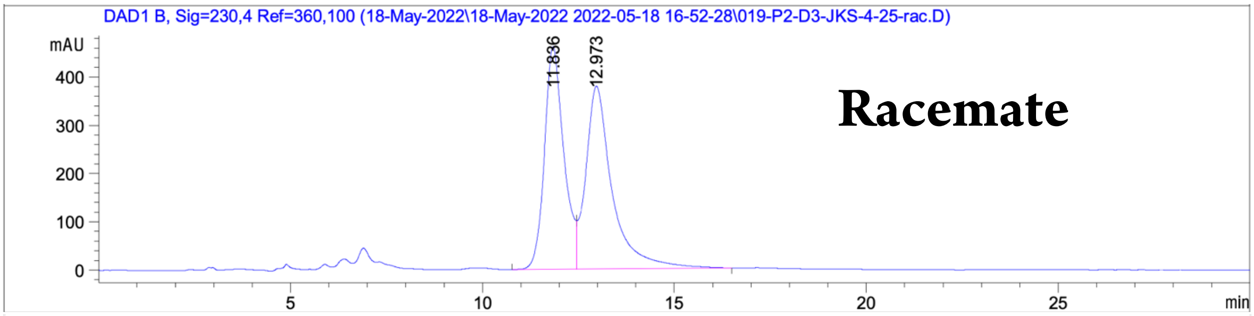

Signal 2: DAD1 B, Sig=230,4 Ref=360,100

Peak #	RetTime [min]	Type	Width [min]	Area [mAU*s]	Height [mAU]	Area %
1	11.836	BV	0.5196	1.61937e4	461.28143	47.0500
2	12.973	VB	0.6888	1.82244e4	379.11612	52.9500
Totals :				3.44181e4	840.39755	

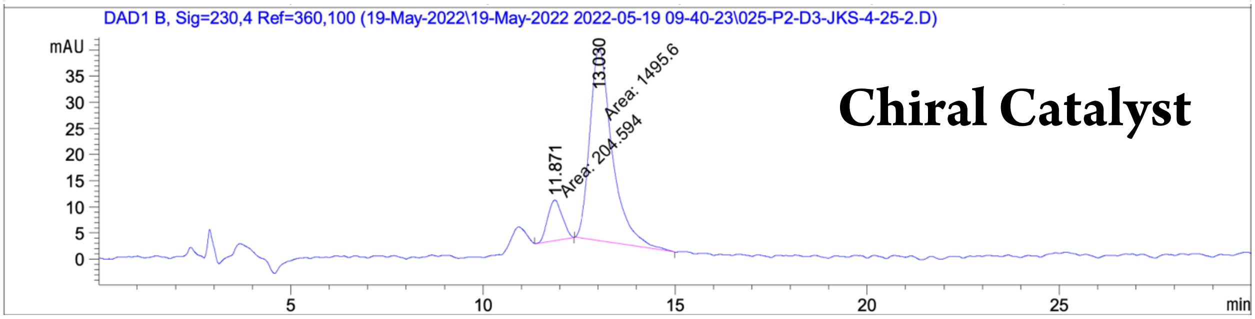

Signal 2: DAD1 B, Sig=230,4 Ref=360,100

Peak #	RetTime [min]	Type	Width [min]	Area [mAU*s]	Height [mAU]	Area %
1	11.871	MM	0.4387	204.59421	7.77239	12.0336
2	13.030	MM	0.6782	1495.59827	36.75165	87.9664
Totals :				1700.19247	44.52404	

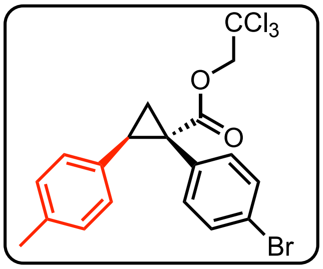

Compound **12**

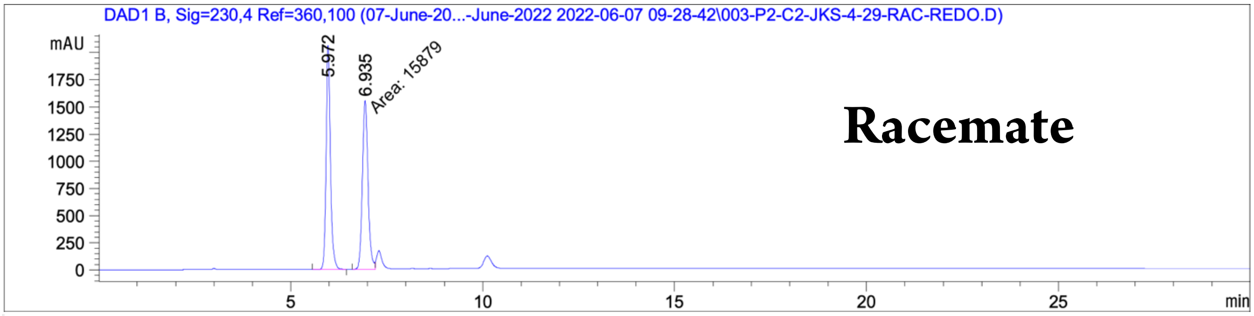

Signal 2: DAD1 B, Sig=230,4 Ref=360,100

Peak #	RetTime [min]	Type	Width [min]	Area [mAU*s]	Height [mAU]	Area %
1	5.972	BB	0.1201	1.62831e4	2062.40088	50.6283
2	6.935	MM	0.1705	1.58790e4	1552.09851	49.3717

Totals : 3.21621e4 3614.49939

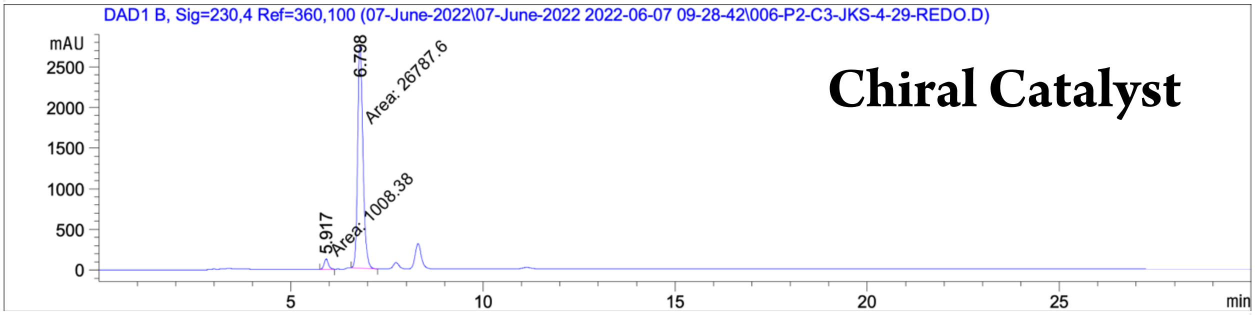

Signal 2: DAD1 B, Sig=230,4 Ref=360,100

Peak #	RetTime [min]	Type	Width [min]	Area [mAU*s]	Height [mAU]	Area %
1	5.917	MM	0.1319	1008.38159	127.42646	3.6278
2	6.798	MM	0.1625	2.67876e4	2747.04321	96.3722

Totals : 2.77960e4 2874.46967

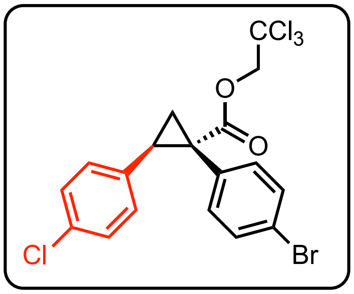

Compound **13**

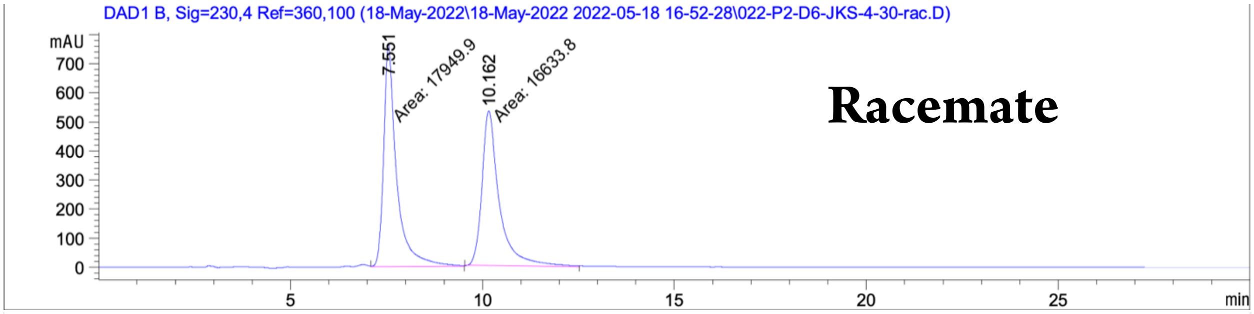

Signal 2: DAD1 B, Sig=230,4 Ref=360,100

Peak #	RetTime [min]	Type	Width [min]	Area [mAU*s]	Height [mAU]	Area %
1	7.551	MM	0.3896	1.79499e4	767.80005	51.9028
2	10.162	MM	0.5212	1.66338e4	531.93866	48.0972
Totals :				3.45837e4	1299.73871	

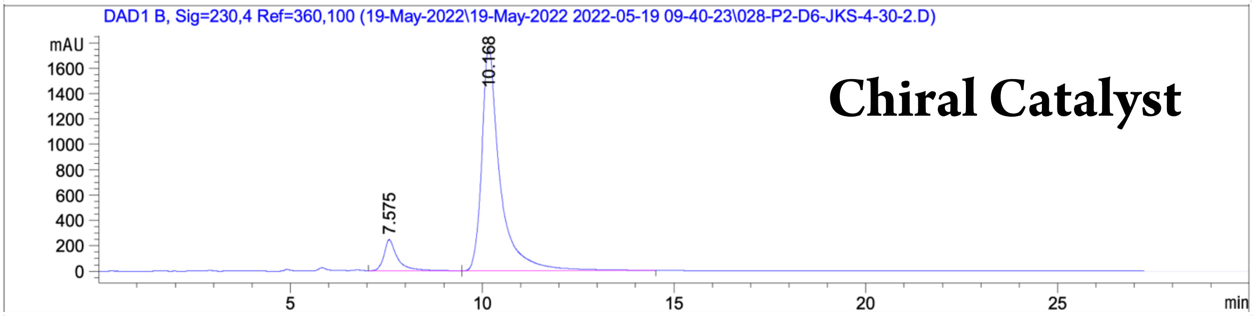

Signal 2: DAD1 B, Sig=230,4 Ref=360,100

Peak #	RetTime [min]	Type	Width [min]	Area [mAU*s]	Height [mAU]	Area %
1	7.575	BB	0.3460	6058.09277	247.13155	9.3882
2	10.168	BB	0.4721	5.84707e4	1766.87756	90.6118
Totals :				6.45288e4	2014.00911	

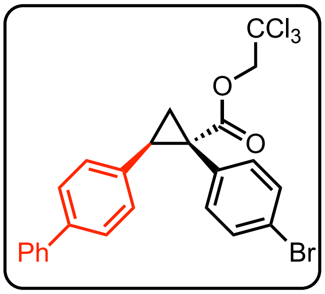

Compound **14**

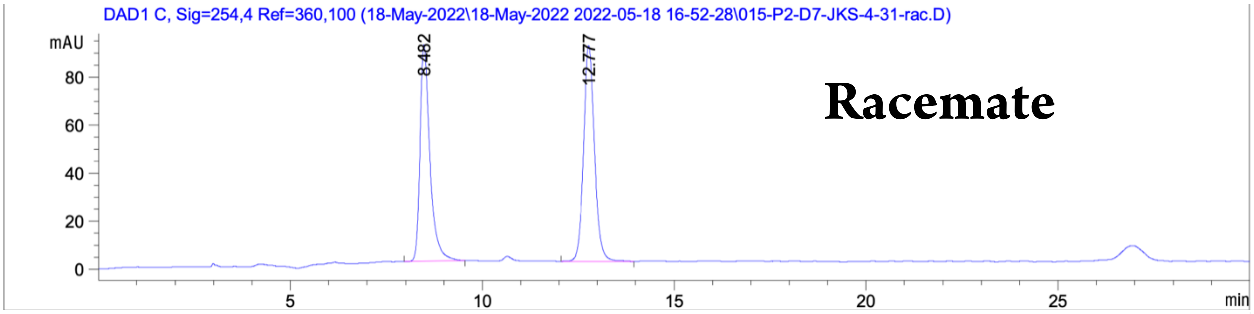

Signal 3: DAD1 C, Sig=254,4 Ref=360,100

Peak #	RetTime [min]	Type	Width [min]	Area [mAU*s]	Height [mAU]	Area %
1	8.482	BB	0.2667	1614.15369	90.24125	47.4621
2	12.777	BB	0.3039	1786.77844	89.89777	52.5379
Totals :				3400.93213	180.13902	

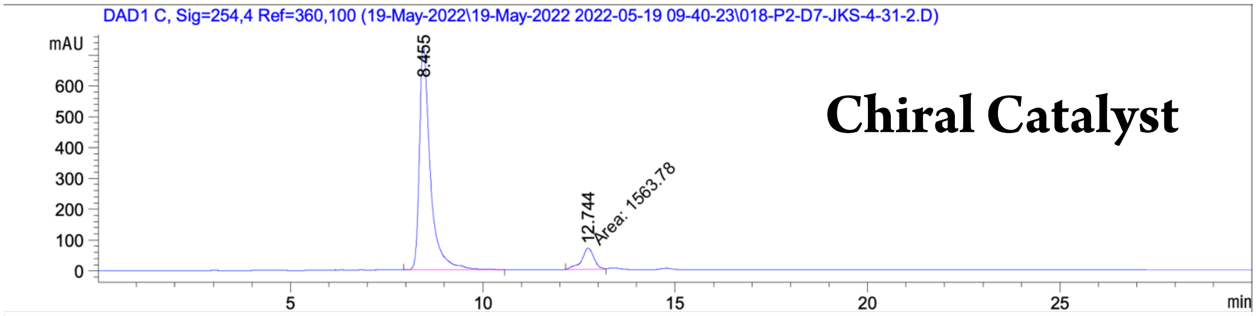

Signal 3: DAD1 C, Sig=254,4 Ref=360,100

Peak #	RetTime [min]	Type	Width [min]	Area [mAU*s]	Height [mAU]	Area %
1	8.455	BB	0.2869	1.42714e4	727.39502	90.1246
2	12.744	MM	0.3777	1563.77612	69.00871	9.8754
Totals :				1.58351e4	796.40372	

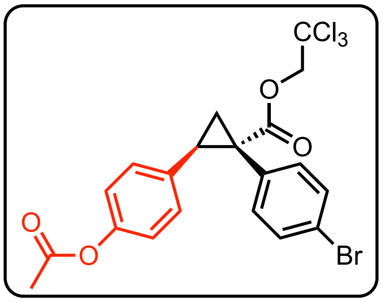

Compound **15**

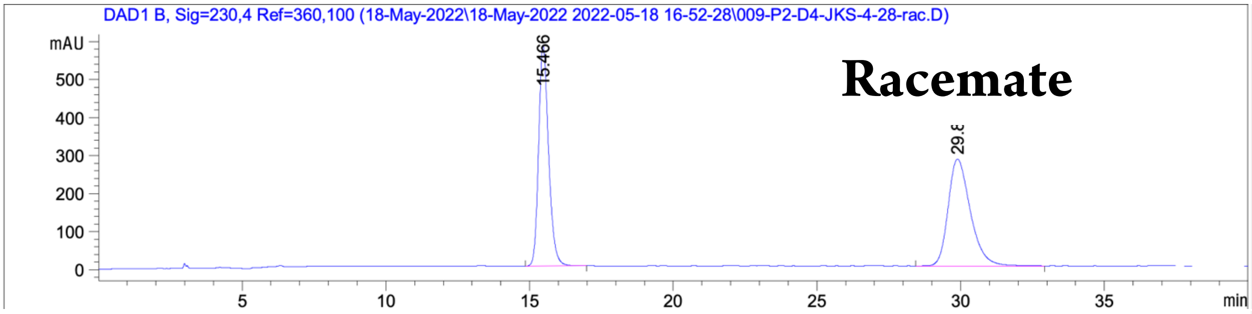

Signal 2: DAD1 B, Sig=230,4 Ref=360,100

Peak #	RetTime [min]	Type	Width [min]	Area [mAU*s]	Height [mAU]	Area %
1	15.466	BB	0.3694	1.40169e4	581.17523	48.0877
2	29.889	BB	0.8214	1.51317e4	281.38800	51.9123

Totals : 2.91486e4 862.56323

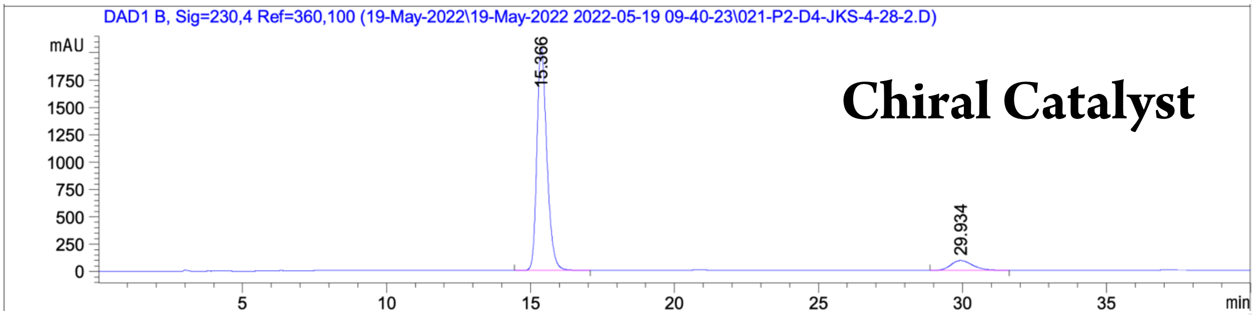

Signal 2: DAD1 B, Sig=230,4 Ref=360,100

Peak #	RetTime [min]	Type	Width [min]	Area [mAU*s]	Height [mAU]	Area %
1	15.366	BB	0.3690	4.91710e4	2041.31836	91.0851
2	29.934	BB	0.8177	4812.54932	90.60869	8.9149

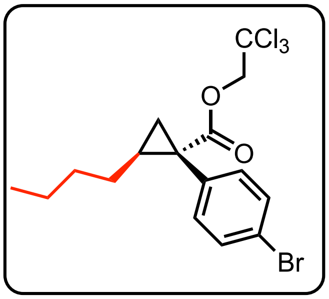

Compound **16**

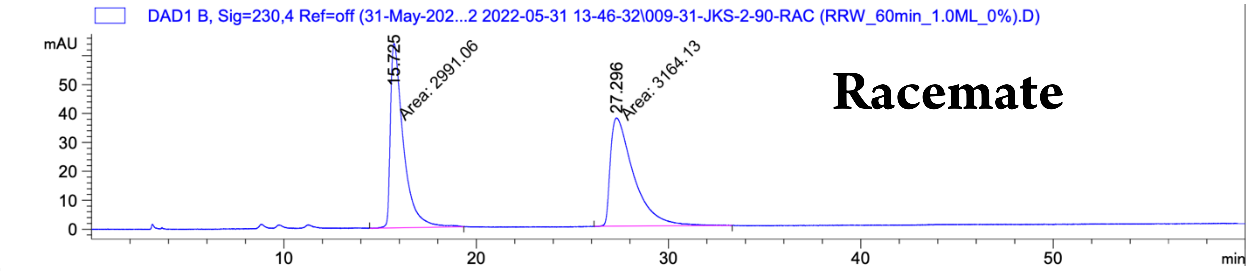


```
Signal 2: DAD1 B, Sig=230,4 Ref=off

Peak RetTime Type  Width     Area      Height     Area
  #   [min]        [min]   [mAU*s]     [mAU]       %
----|-------|----|-------|----------|----------|--------|
   1  15.725 MM    0.7814 2991.06396   63.79838  48.5941
   2  27.296 MM    1.4065 3164.13354   37.49349  51.4059

Totals :                  6155.19751  101.29187
```

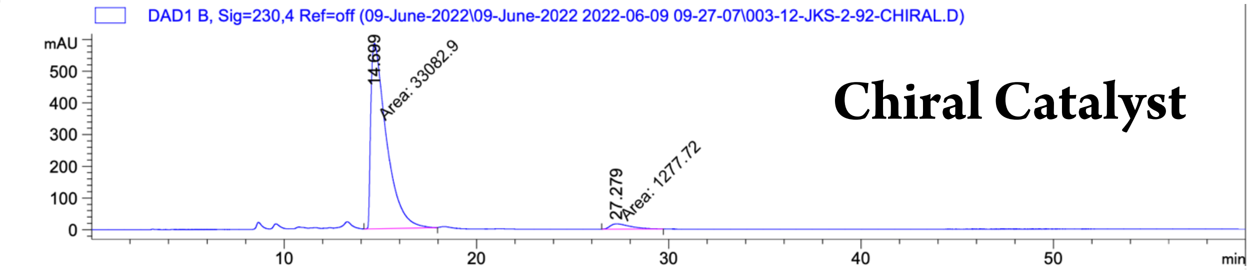


```
Signal 2: DAD1 B, Sig=230,4 Ref=off

Peak RetTime Type  Width     Area      Height     Area
  #   [min]        [min]   [mAU*s]     [mAU]       %
----|-------|----|-------|----------|----------|--------|
   1  14.699 MM    0.9411 3.30829e4   585.92126  96.2814
   2  27.279 MM    1.2558 1277.71973   16.95697   3.7186

Totals :                  3.43607e4   602.87823
```

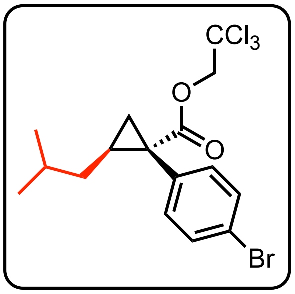

Compound **17**

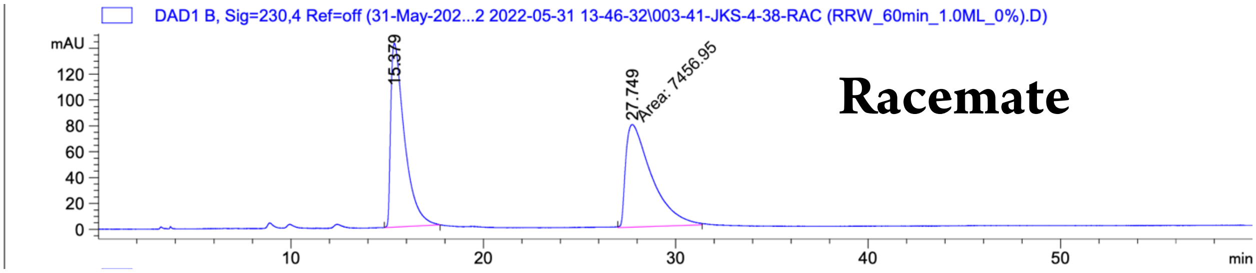

Signal 2: DAD1 B, Sig=230,4 Ref=off

Peak #	RetTime [min]	Type	Width [min]	Area [mAU*s]	Height [mAU]	Area %
1	15.379	BV R	0.5819	7056.59131	142.20984	48.6207
2	27.749	MM	1.5673	7456.95361	79.29698	51.3793

Totals : 1.45135e4 221.50682

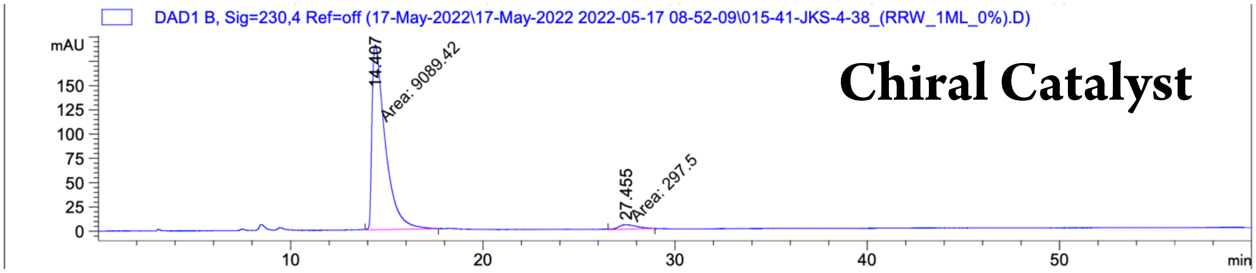

Signal 2: DAD1 B, Sig=230,4 Ref=off

Peak #	RetTime [min]	Type	Width [min]	Area [mAU*s]	Height [mAU]	Area %
1	14.407	MM	0.7962	9089.41895	190.27261	96.8307
2	27.455	MM	1.0848	297.49957	4.57061	3.1693

Totals : 9386.91852 194.84322

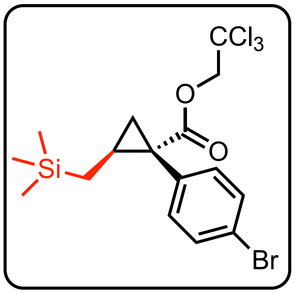

Compound **18**

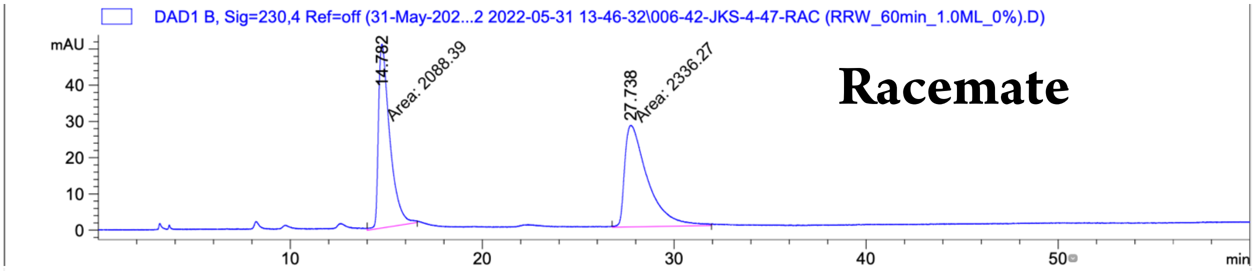

Signal 2: DAD1 B, Sig=230,4 Ref=off

Peak #	RetTime [min]	Type	Width [min]	Area [mAU*s]	Height [mAU]	Area %
1	14.782	MM	0.6861	2088.39307	50.73406	47.1990
2	27.738	MM	1.3863	2336.26611	28.08833	52.8010
Totals :				4424.65918	78.82239	

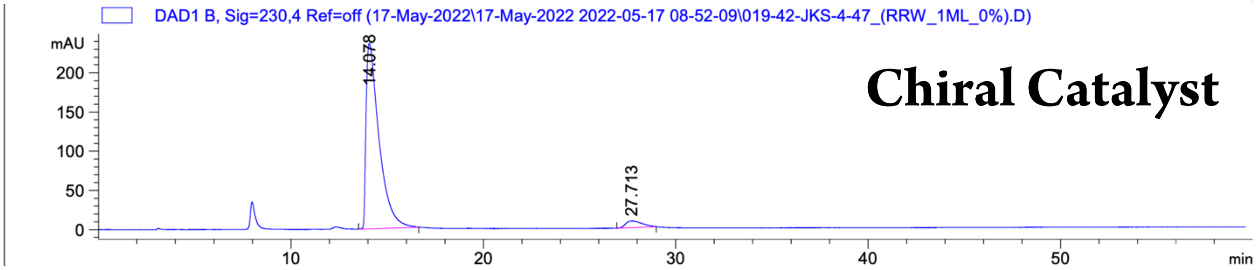

Signal 2: DAD1 B, Sig=230,4 Ref=off

Peak #	RetTime [min]	Type	Width [min]	Area [mAU*s]	Height [mAU]	Area %
1	14.078	VV R	0.5502	1.10325e4	236.72404	95.6580
2	27.713	BV R	0.6836	500.77664	8.58471	4.3420
Totals :				1.15332e4	245.30875	

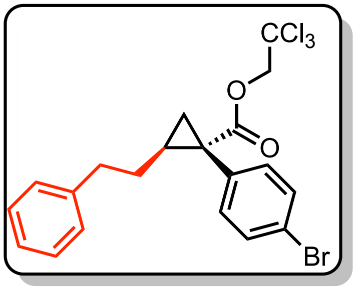

Compound **19**

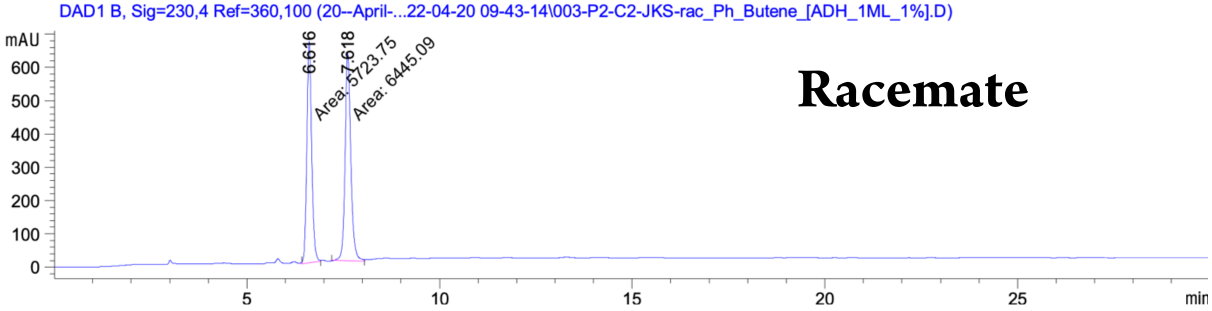

Peak #	RetTime [min]	Type	Width [min]	Area [mAU*s]	Height [mAU]	Area %
1	6.616	MM	0.1433	5723.75439	665.78522	47.0362
2	7.618	MM	0.1730	6445.08643	620.92499	52.9638

Totals : 1.21688e4 1286.71021

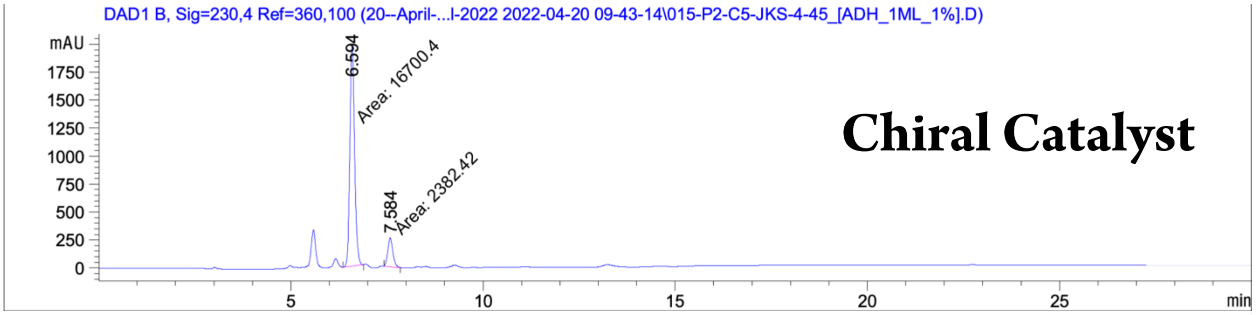

Signal 2: DAD1 B, Sig=230,4 Ref=360,100

Peak #	RetTime [min]	Type	Width [min]	Area [mAU*s]	Height [mAU]	Area %
1	6.594	MM	0.1404	1.67004e4	1982.03979	87.5154
2	7.584	MM	0.1542	2382.41650	257.46539	12.4846

Totals : 1.90829e4 2239.50519

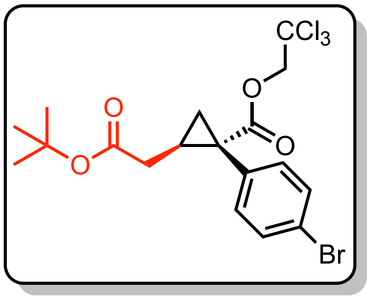

Compound **20**

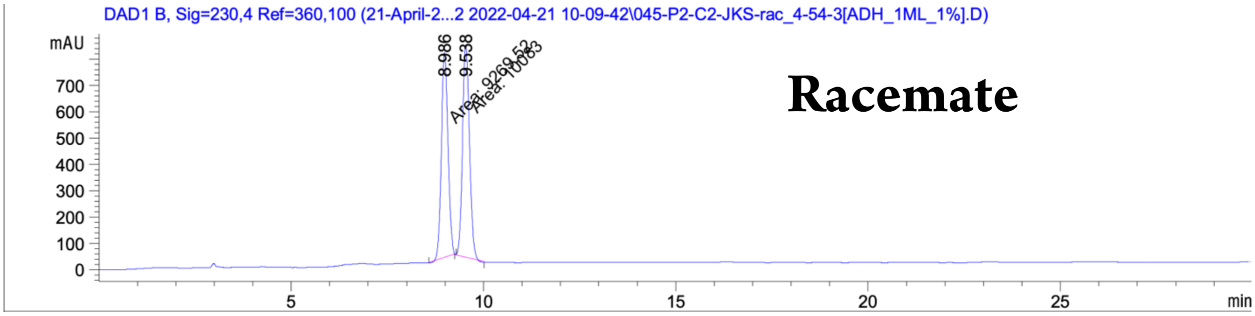

Signal 2: DAD1 B, Sig=230,4 Ref=360,100

Peak #	RetTime [min]	Type	Width [min]	Area [mAU*s]	Height [mAU]	Area %
1	8.986	MM	0.1992	9269.52246	775.65826	47.8982
2	9.538	MM	0.2084	1.00830e4	806.36584	52.1018
Totals :				1.93525e4	1582.02411	

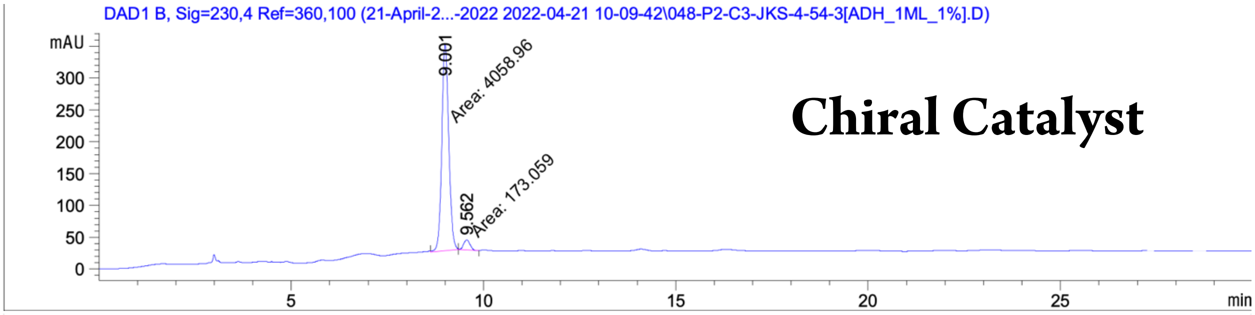

Peak #	RetTime [min]	Type	Width [min]	Area [mAU*s]	Height [mAU]	Area %
1	9.001	MM	0.2083	4058.95850	324.79349	95.9107
2	9.562	MM	0.1856	173.05934	15.53821	4.0893
Totals :				4232.01784	340.33170	

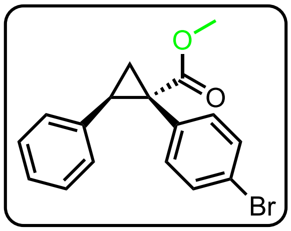

Compound **21**

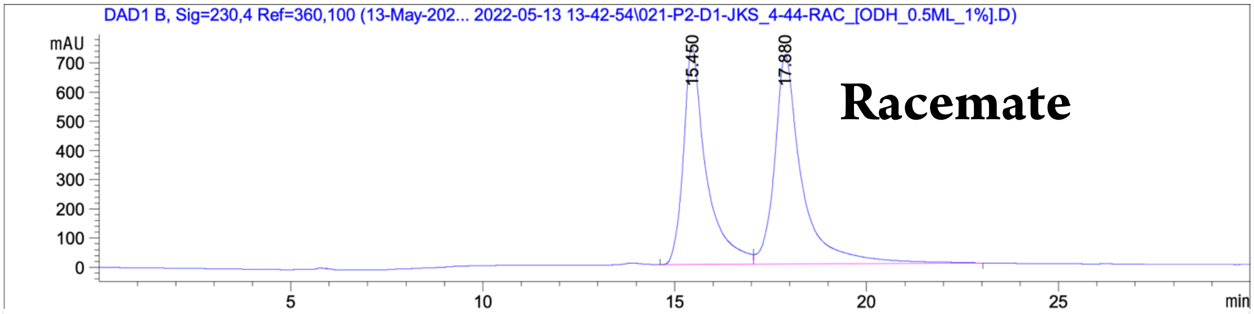

Signal 2: DAD1 B, Sig=230,4 Ref=360,100

Peak #	RetTime [min]	Type	Width [min]	Area [mAU*s]	Height [mAU]	Area %
1	15.450	BV	0.5793	3.08433e4	750.74835	46.4418
2	17.880	VB	0.6983	3.55696e4	717.59296	53.5582

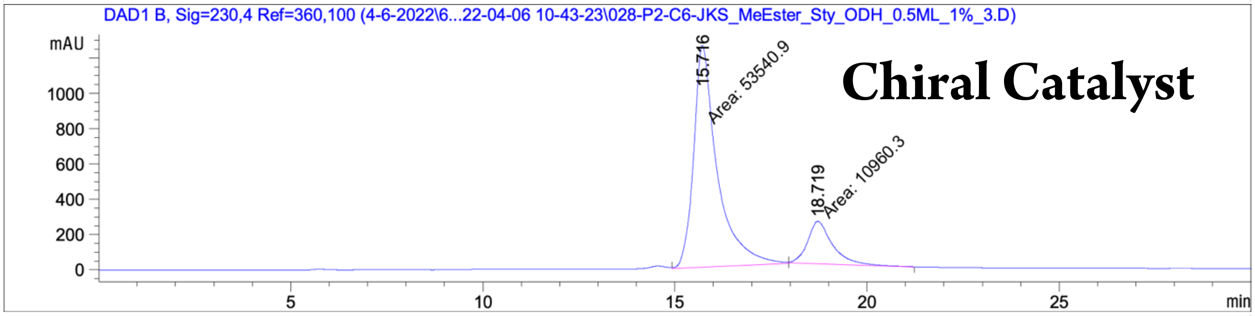

Signal 2: DAD1 B, Sig=230,4 Ref=360,100

Peak #	RetTime [min]	Type	Width [min]	Area [mAU*s]	Height [mAU]	Area %
1	15.716	MM	0.7105	5.35409e4	1255.95544	83.0076
2	18.719	MM	0.7607	1.09603e4	240.13960	16.9924
Totals :				6.45013e4	1496.09505	

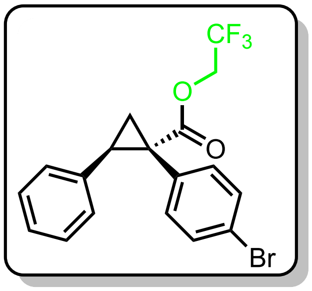

Compound **22**

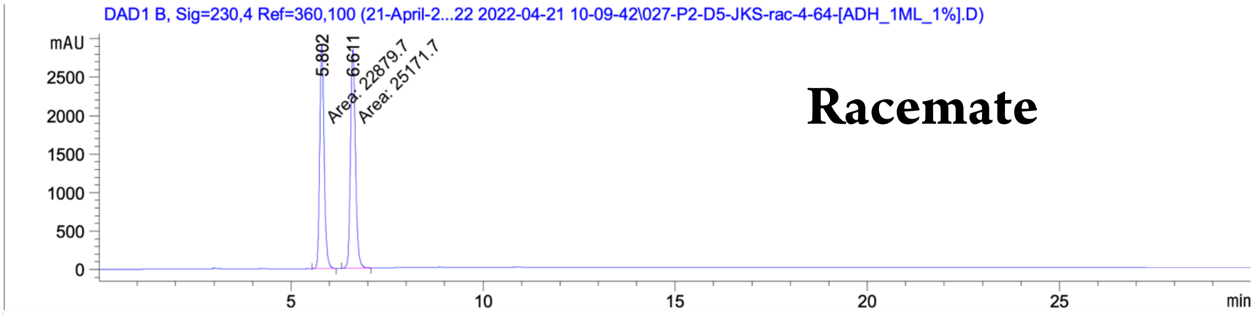

Signal 2: DAD1 B, Sig=230,4 Ref=360,100

Peak #	RetTime [min]	Type	Width [min]	Area [mAU*s]	Height [mAU]	Area %
1	5.802	MM	0.1308	2.28797e4	2914.32568	47.6151
2	6.611	MM	0.1476	2.51717e4	2842.84131	52.3849
Totals :				4.80514e4	5757.16699	

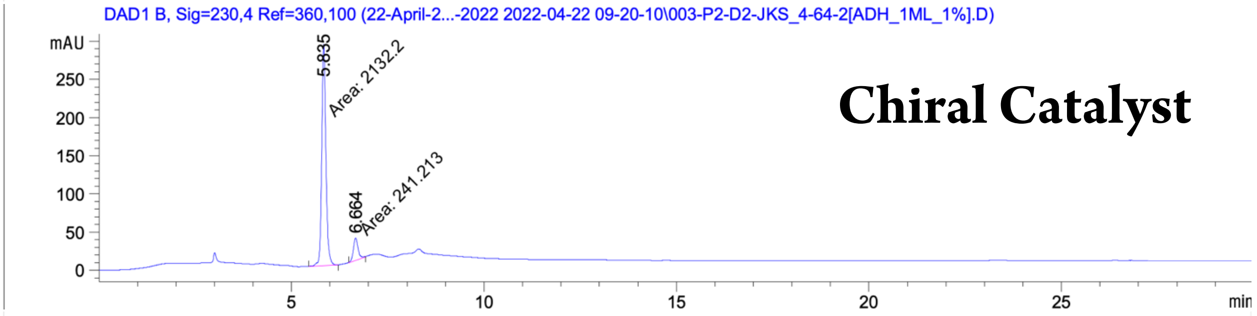

Signal 2: DAD1 B, Sig=230,4 Ref=360,100

Peak #	RetTime [min]	Type	Width [min]	Area [mAU*s]	Height [mAU]	Area %
1	5.835	MM	0.1228	2132.19556	289.37891	89.8369
2	6.664	MM	0.1378	241.21251	29.17617	10.1631
Totals :				2373.40807	318.55508	

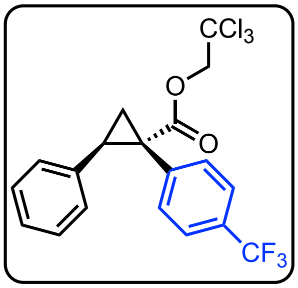

Compound **23**

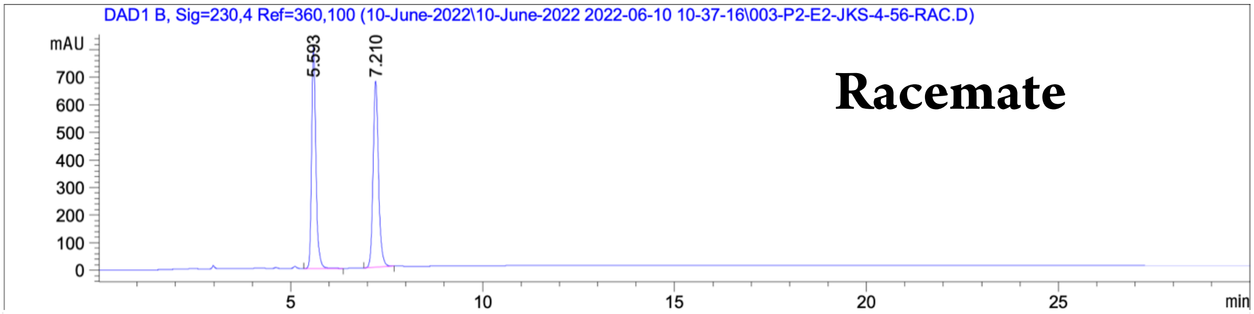

Signal 2: DAD1 B, Sig=230,4 Ref=360,100

Peak #	RetTime [min]	Type	Width [min]	Area [mAU*s]	Height [mAU]	Area %
1	5.593	BB	0.1148	6162.12988	809.81104	48.3796
2	7.210	BB	0.1475	6574.90234	675.65918	51.6204

Totals : 1.27370e4 1485.47021

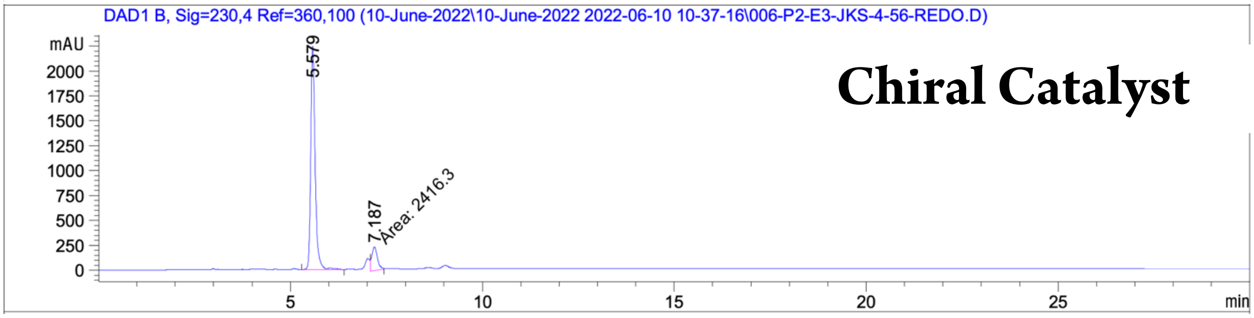

Signal 2: DAD1 B, Sig=230,4 Ref=360,100

Peak #	RetTime [min]	Type	Width [min]	Area [mAU*s]	Height [mAU]	Area %
1	5.579	BV R	0.1166	1.76200e4	2244.50024	87.9404
2	7.187	MM	0.1683	2416.29834	239.29219	12.0596

Totals : 2.00363e4 2483.79243

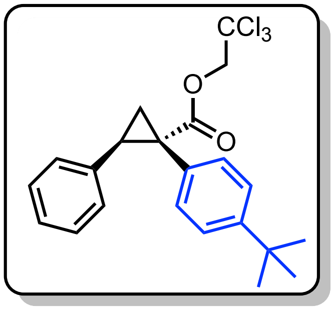

Compound **24**

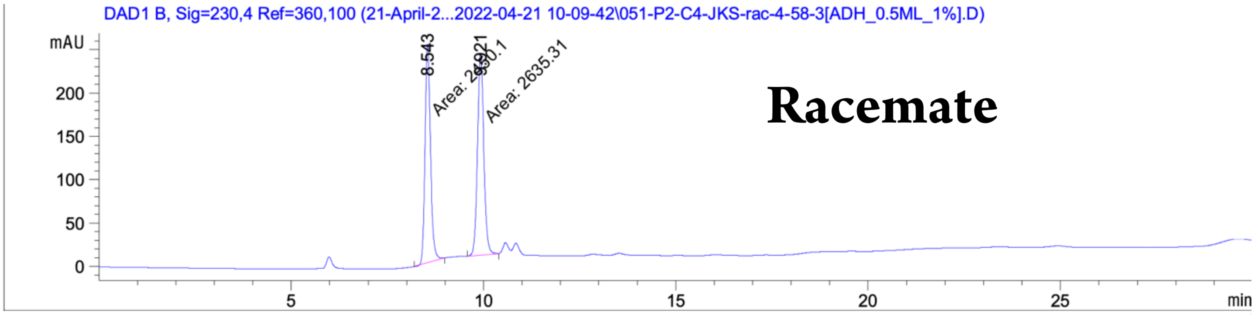


```
Signal 2: DAD1 B, Sig=230,4 Ref=360,100

Peak RetTime Type  Width     Area      Height     Area
  #   [min]        [min]   [mAU*s]     [mAU]       %
----|-------|----|-------|----------|----------|--------|
   1   8.543 MM    0.1619 2450.09912  252.23808  48.1790
   2   9.921 MM    0.1888 2635.30811  232.61414  51.8210

Totals :                  5085.40723  484.85222
```

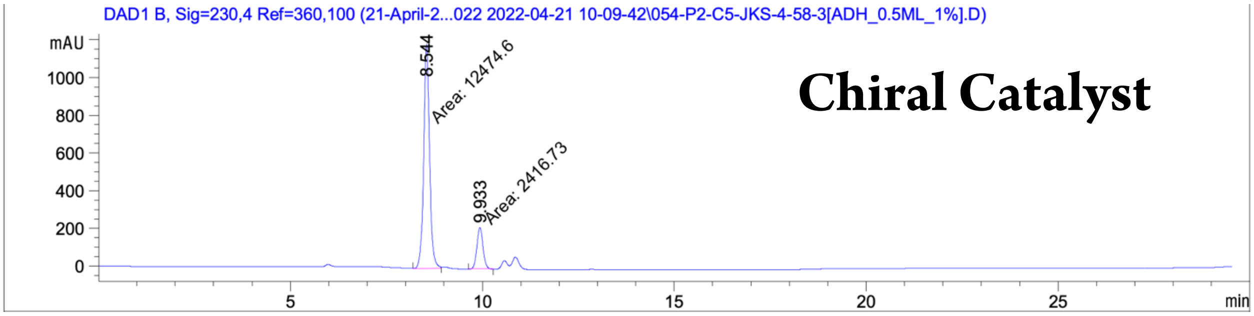


```
Signal 2: DAD1 B, Sig=230,4 Ref=360,100

Peak RetTime Type  Width     Area      Height     Area
  #   [min]        [min]   [mAU*s]     [mAU]       %
----|-------|----|-------|----------|----------|--------|
   1   8.544 MM    0.1751 1.24746e4 1187.05261  83.7709
   2   9.933 MM    0.1828 2416.72705  220.29327  16.2291

Totals :                  1.48913e4 1407.34589
```

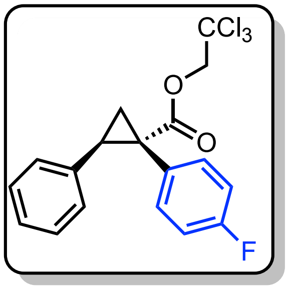

Compound **25**

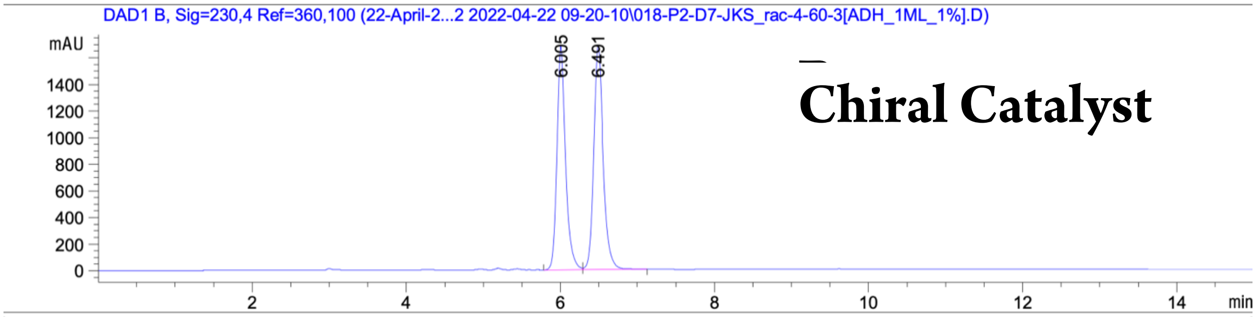

Signal 2: DAD1 B, Sig=230,4 Ref=360,100

Peak #	RetTime [min]	Type	Width [min]	Area [mAU*s]	Height [mAU]	Area %
1	6.005	BV	0.1183	1.33428e4	1686.38867	48.0813
2	6.491	VB	0.1299	1.44078e4	1683.29883	51.9187
Totals :				2.77506e4	3369.68750	

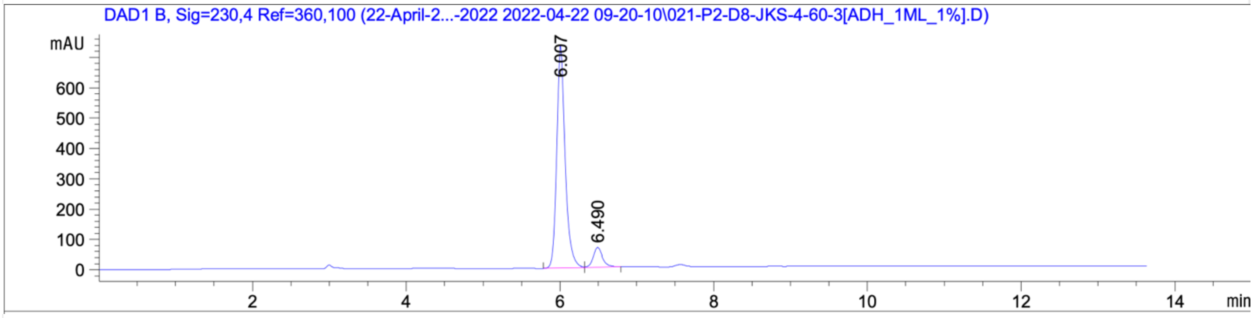

Signal 2: DAD1 B, Sig=230,4 Ref=360,100

Peak #	RetTime [min]	Type	Width [min]	Area [mAU*s]	Height [mAU]	Area %
1	6.007	BV	0.1168	5722.35840	735.22961	91.2220
2	6.490	VB	0.1282	550.64423	65.43978	8.7780
Totals :				6273.00262	800.66940	

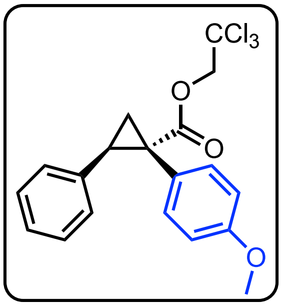

Compound **26**

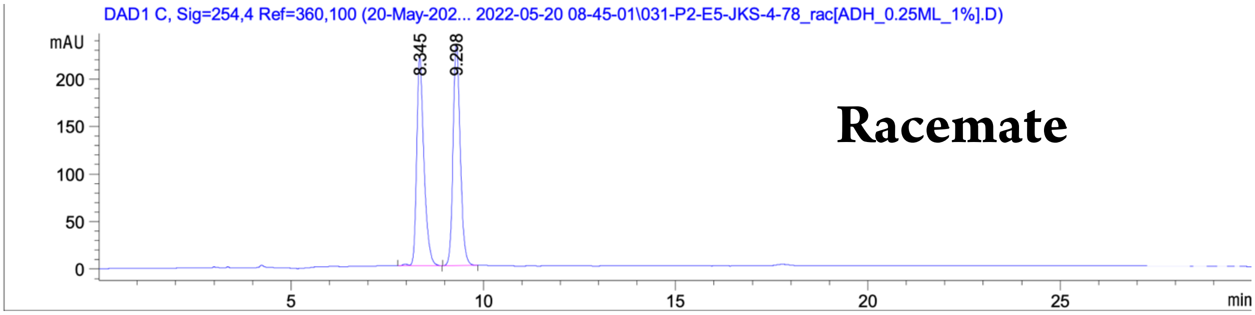

Signal 3: DAD1 C, Sig=254,4 Ref=360,100

Peak #	RetTime [min]	Type	Width [min]	Area [mAU*s]	Height [mAU]	Area %
1	8.345	VB R	0.1994	2910.02563	219.93542	49.0378
2	9.298	BB	0.1976	3024.21924	232.75323	50.9622

Totals : 5934.24487 452.68866

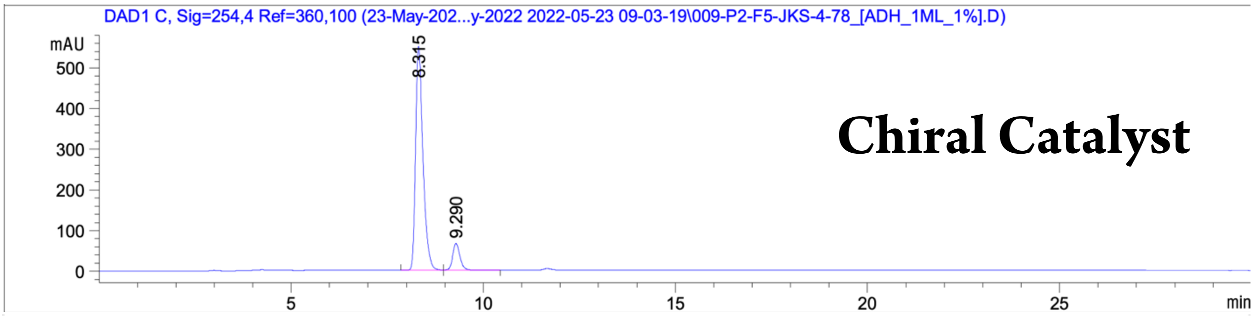

Signal 3: DAD1 C, Sig=254,4 Ref=360,100

Peak #	RetTime [min]	Type	Width [min]	Area [mAU*s]	Height [mAU]	Area %
1	8.315	BB	0.2015	7328.62402	549.76813	89.6529
2	9.290	BB	0.1972	845.81879	65.27042	10.3471

Totals : 8174.44281 615.03855

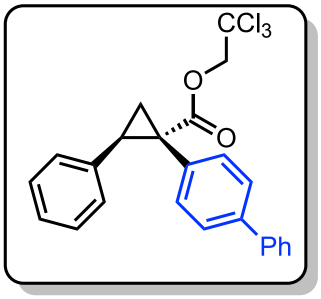

Compound **27**

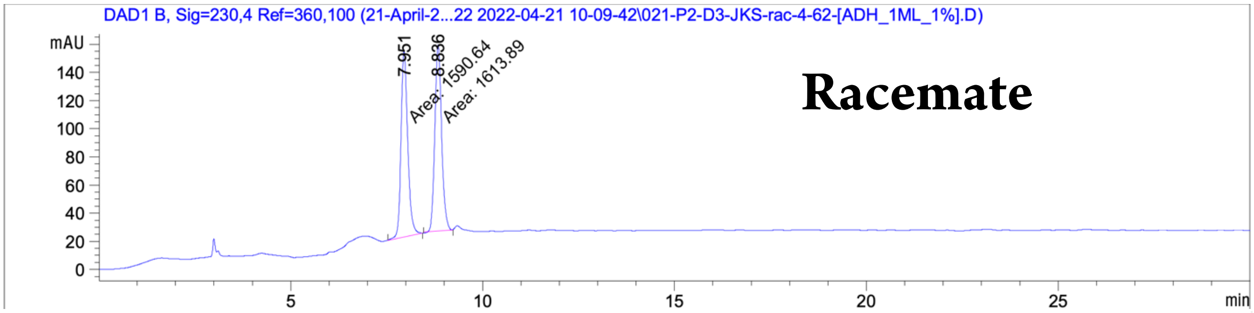

Signal 2: DAD1 B, Sig=230,4 Ref=360,100

Peak #	RetTime [min]	Type	Width [min]	Area [mAU*s]	Height [mAU]	Area %
1	7.951	MM	0.2007	1590.64087	132.11815	49.6373
2	8.836	MM	0.2031	1613.88806	132.43649	50.3627

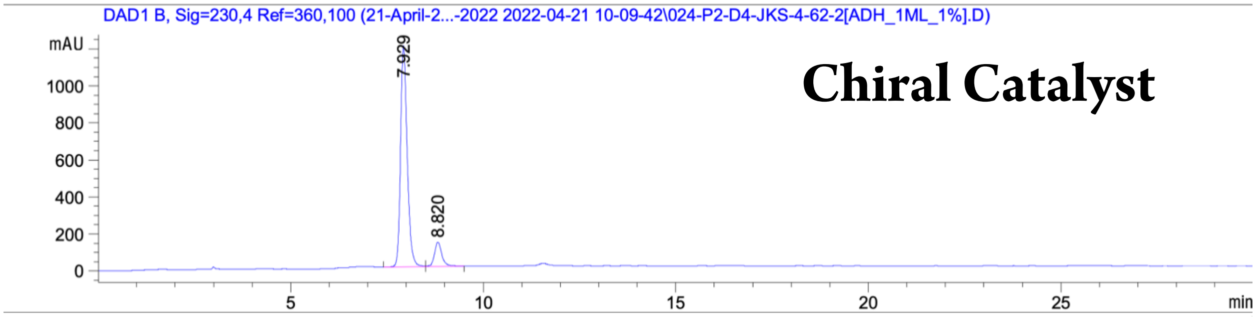

Signal 2: DAD1 B, Sig=230,4 Ref=360,100

Peak #	RetTime [min]	Type	Width [min]	Area [mAU*s]	Height [mAU]	Area %
1	7.929	BV	0.1817	1.43243e4	1196.35999	89.1717
2	8.820	VB	0.1989	1739.42395	131.02821	10.8283

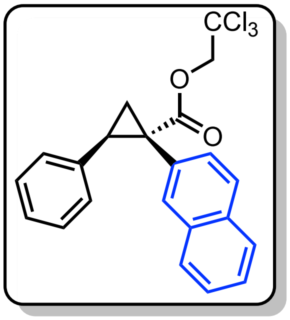

Compound **28**

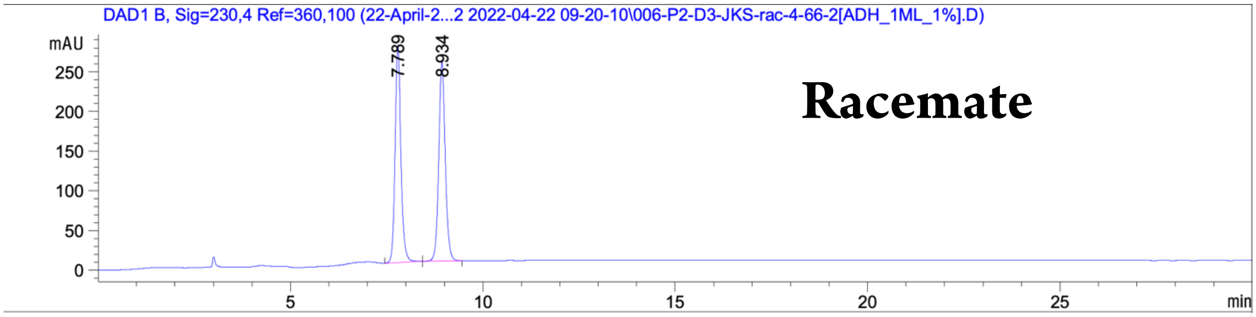

Signal 2: DAD1 B, Sig=230,4 Ref=360,100

Peak #	RetTime [min]	Type	Width [min]	Area [mAU*s]	Height [mAU]	Area %
1	7.789	BB	0.1586	2872.49951	273.36664	49.1969
2	8.934	BB	0.1808	2966.28003	252.93414	50.8031

Totals : 5838.77954 526.30078

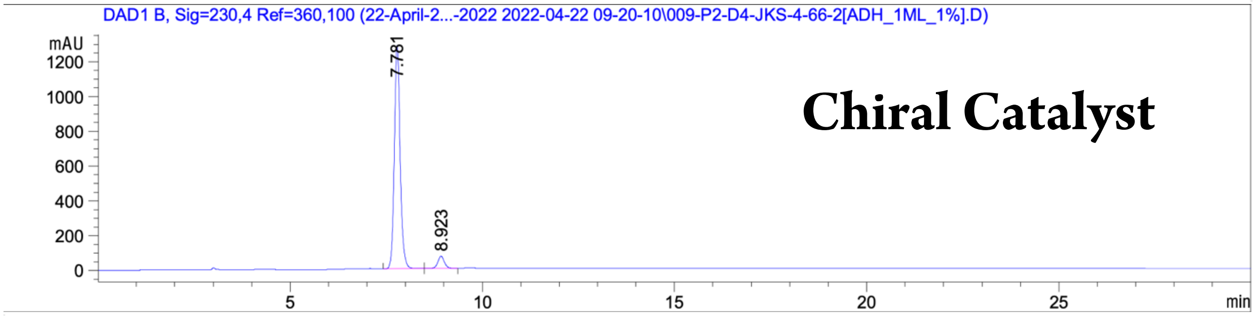

Signal 2: DAD1 B, Sig=230,4 Ref=360,100

Peak #	RetTime [min]	Type	Width [min]	Area [mAU*s]	Height [mAU]	Area %
1	7.781	BB	0.1607	1.35192e4	1285.34973	94.1376
2	8.923	BB	0.1790	841.90424	71.71494	5.8624

Totals : 1.43611e4 1357.06467

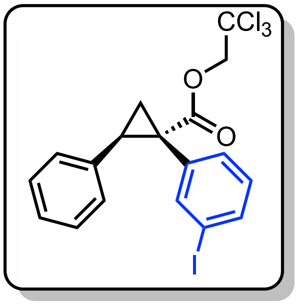

Compound **29**

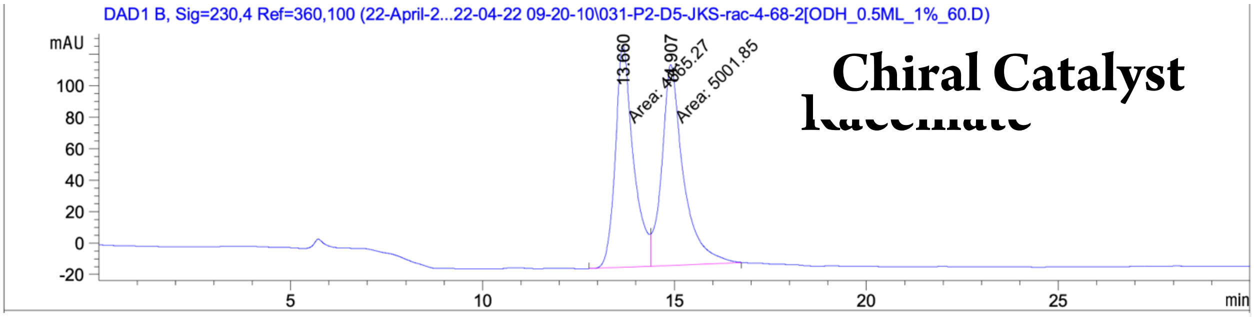

Signal 2: DAD1 B, Sig=230,4 Ref=360,100

Peak #	RetTime [min]	Type	Width [min]	Area [mAU*s]	Height [mAU]	Area %
1	13.660	MF	0.5490	4665.26953	141.61685	48.2591
2	14.907	FM	0.6527	5001.85059	127.72429	51.7409
Totals :				9667.12012	269.34114	

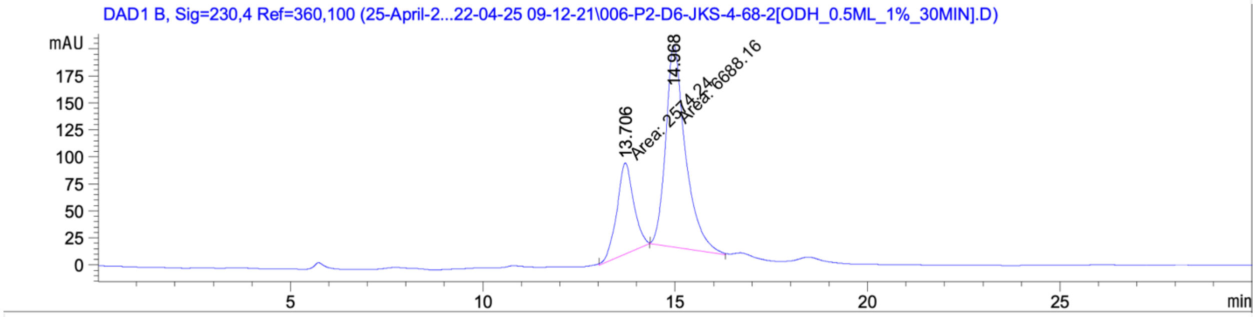

Signal 2: DAD1 B, Sig=230,4 Ref=360,100

Peak #	RetTime [min]	Type	Width [min]	Area [mAU*s]	Height [mAU]	Area %
1	13.706	MM	0.5089	2574.24121	84.30233	27.7924
2	14.968	MM	0.5958	6688.15820	187.10114	72.2076
Totals :				9262.39941	271.40347	

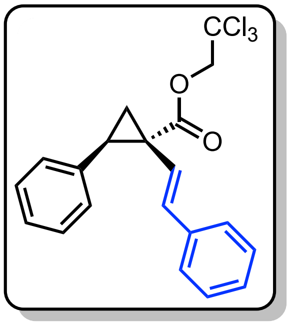

Compound **30**

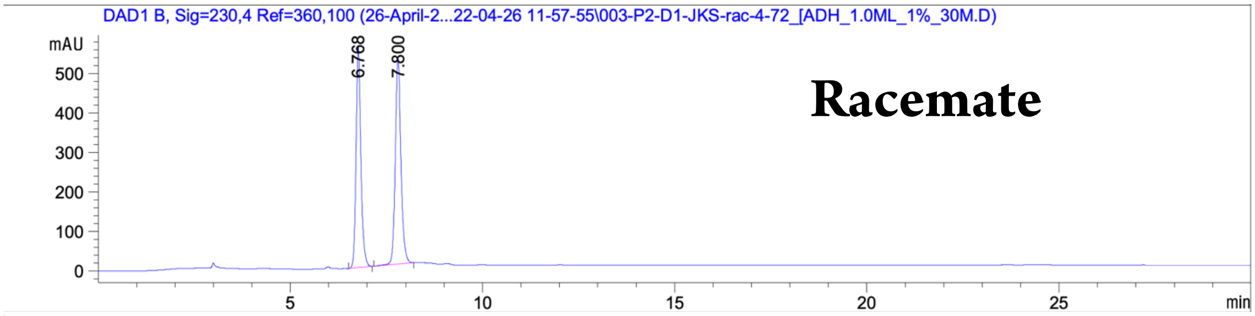

Signal 2: DAD1 B, Sig=230,4 Ref=360,100

Peak #	RetTime [min]	Type	Width [min]	Area [mAU*s]	Height [mAU]	Area %
1	6.768	BB	0.1328	4944.46826	561.41315	47.9950
2	7.800	BB	0.1558	5357.57715	521.66431	52.0050
Totals :				1.03020e4	1083.07745	

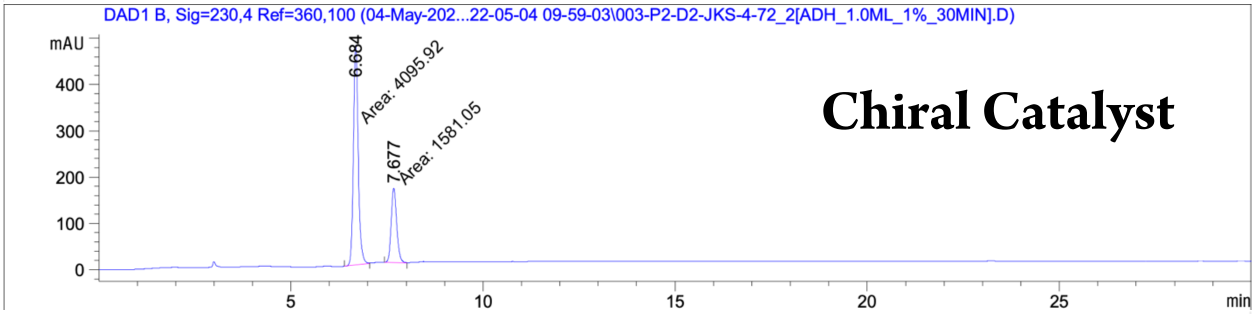

Signal 2: DAD1 B, Sig=230,4 Ref=360,100

Peak #	RetTime [min]	Type	Width [min]	Area [mAU*s]	Height [mAU]	Area %
1	6.684	MM	0.1437	4095.92334	475.20187	72.1497
2	7.677	MM	0.1634	1581.05249	161.25577	27.8503
Totals :				5676.97583	636.45764	

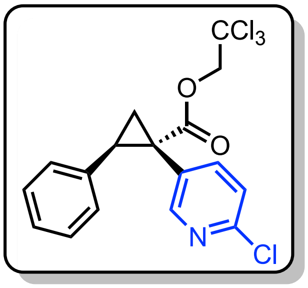

Compound **31**

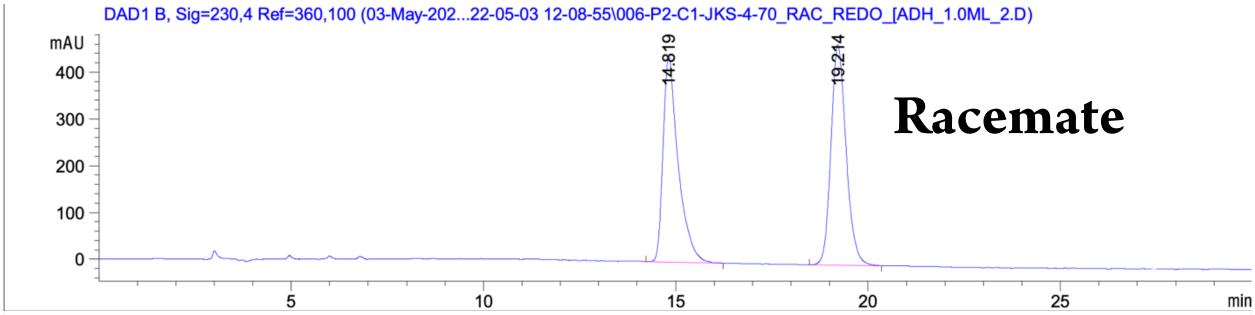

Signal 2: DAD1 B, Sig=230,4 Ref=360,100

Peak #	RetTime [min]	Type	Width [min]	Area [mAU*s]	Height [mAU]	Area %
1	14.819	BV R	0.4017	1.20413e4	439.43500	48.3026
2	19.214	BB	0.4192	1.28876e4	471.10703	51.6974

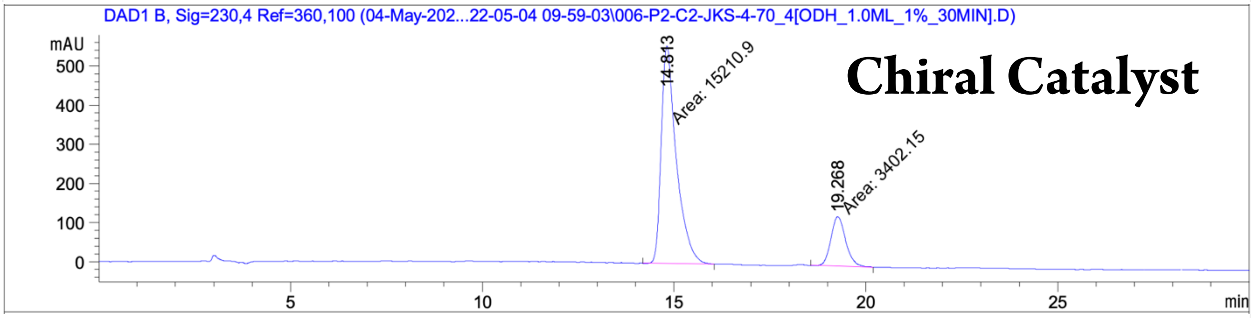

Signal 2: DAD1 B, Sig=230,4 Ref=360,100

Peak #	RetTime [min]	Type	Width [min]	Area [mAU*s]	Height [mAU]	Area %
1	14.813	MM	0.4559	1.52109e4	556.06555	81.7217
2	19.268	MM	0.4493	3402.15479	126.20539	18.2783
Totals :				1.86131e4	682.27094	

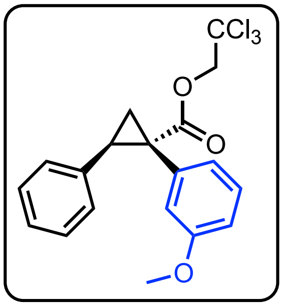

Compound **32**

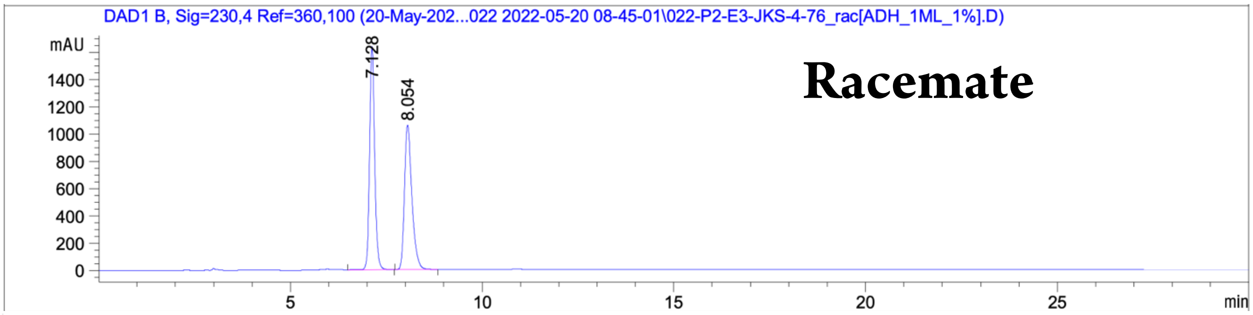

Signal 2: DAD1 B, Sig=230,4 Ref=360,100

Peak #	RetTime [min]	Type	Width [min]	Area [mAU*s]	Height [mAU]	Area %
1	7.128	VB R	0.1376	1.48454e4	1637.45007	51.0205
2	8.054	BB	0.2050	1.42515e4	1058.95117	48.9795

Totals : 2.90968e4 2696.40125

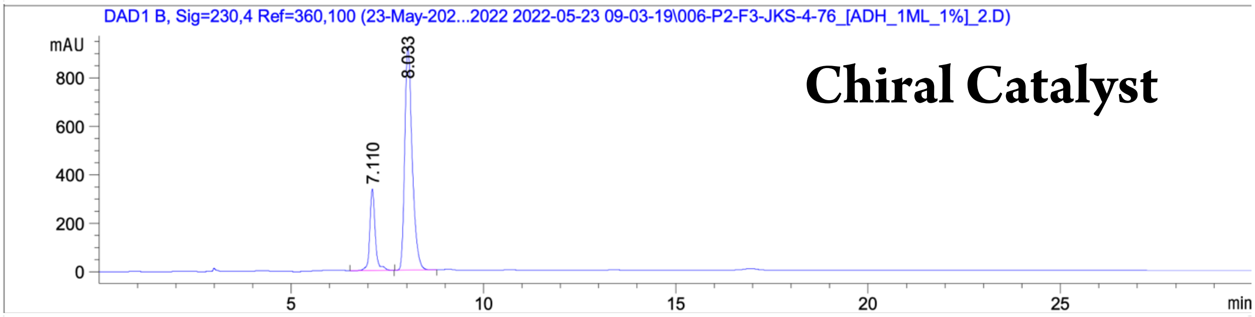

Signal 2: DAD1 B, Sig=230,4 Ref=360,100

Peak #	RetTime [min]	Type	Width [min]	Area [mAU*s]	Height [mAU]	Area %
1	7.110	BV R	0.1417	3231.21802	336.11502	20.6020
2	8.033	BB	0.2057	1.24528e4	921.41992	79.3980

Totals : 1.56840e4 1257.53494

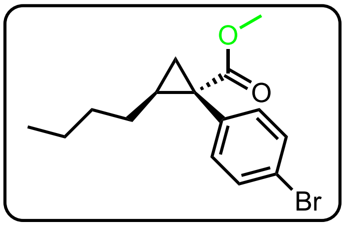

Compound **33**

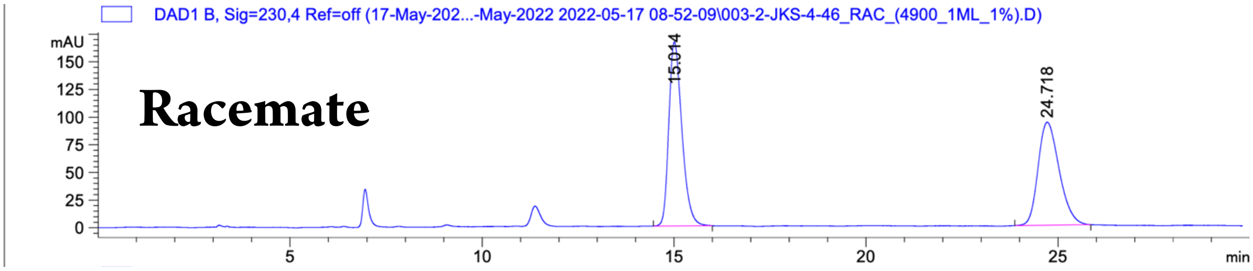

Signal 2: DAD1 B, Sig=230,4 Ref=off

Peak #	RetTime [min]	Type	Width [min]	Area [mAU*s]	Height [mAU]	Area %
1	15.014	BV R	0.3357	3931.14502	166.36209	52.4483
2	24.718	BV R	0.4530	3564.13403	93.13728	47.5517
Totals :				7495.27905	259.49937	

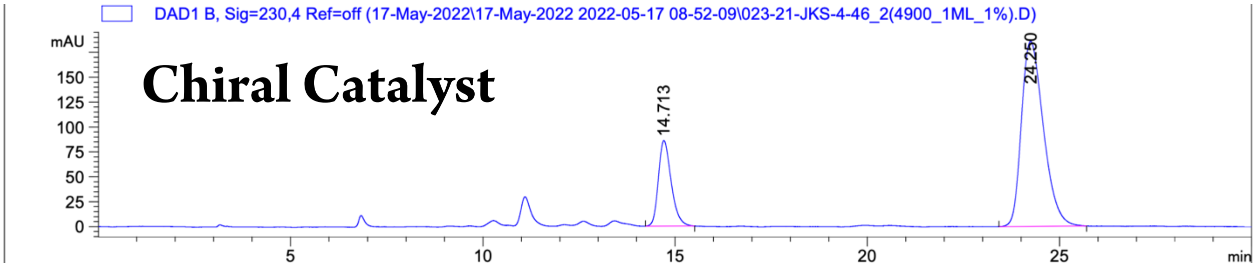

Signal 2: DAD1 B, Sig=230,4 Ref=off

Peak #	RetTime [min]	Type	Width [min]	Area [mAU*s]	Height [mAU]	Area %
1	14.713	BV R	0.3004	1986.03662	85.83268	21.1844
2	24.250	VV R	0.4685	7388.94775	185.78978	78.8156
Totals :				9374.98438	271.62246	

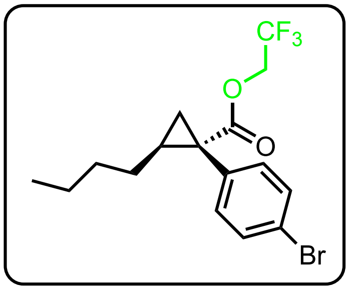

Compound **34**

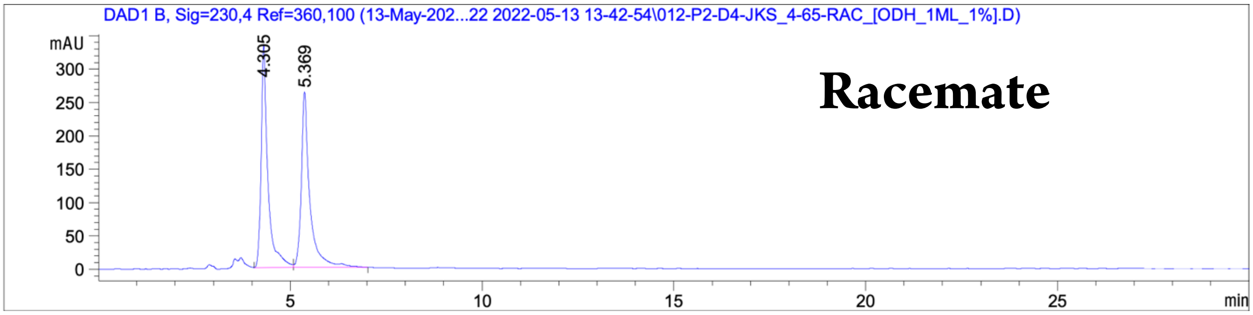


```
Signal 2: DAD1 B, Sig=230,4 Ref=360,100

Peak RetTime Type  Width     Area      Height     Area
  #    [min]        [min]   [mAU*s]     [mAU]       %
----|-------|----|-------|----------|----------|--------|
   1   4.305 BV    0.1713 4091.61035  333.14453  50.0894
   2   5.369 VV R  0.2130 4076.99976  263.24207  49.9106

Totals :                  8168.61011  596.38660
```

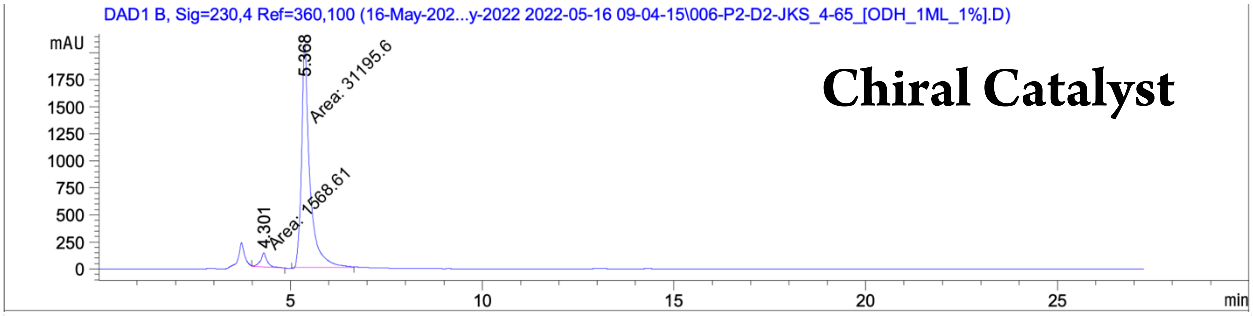


```
Signal 2: DAD1 B, Sig=230,4 Ref=360,100

Peak RetTime Type  Width     Area      Height     Area
  #    [min]        [min]   [mAU*s]     [mAU]       %
----|-------|----|-------|----------|----------|--------|
   1   4.301 MM    0.2002 1568.60901  130.61334   4.7876
   2   5.368 MM    0.2526 3.11956e4  2058.07764  95.2124

Totals :                  3.27642e4  2188.69098
```

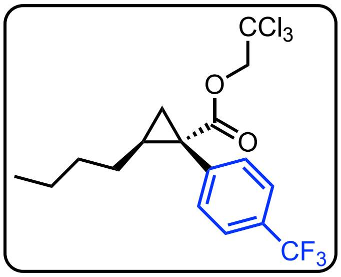

Compound **35**

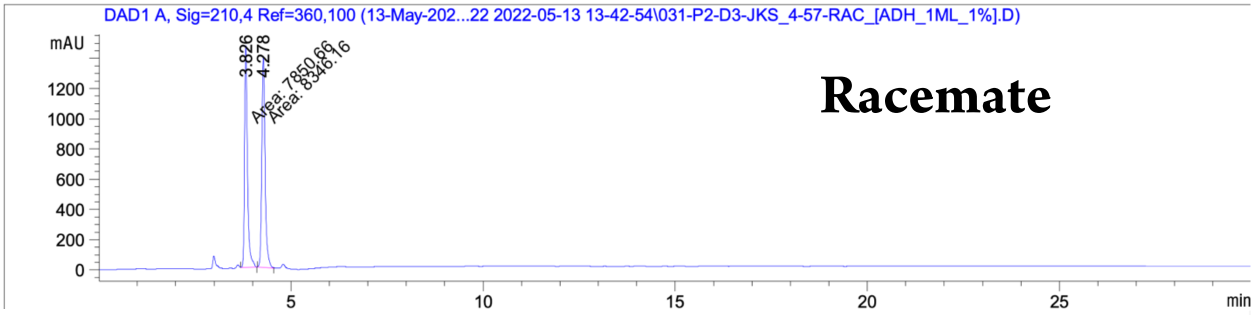

Signal 1: DAD1 A, Sig=210,4 Ref=360,100

Peak #	RetTime [min]	Type	Width [min]	Area [mAU*s]	Height [mAU]	Area %
1	3.826	MM	0.0889	7850.65771	1472.00183	48.4704
2	4.278	MM	0.1001	8346.16016	1389.61646	51.5296
Totals :				1.61968e4	2861.61829	

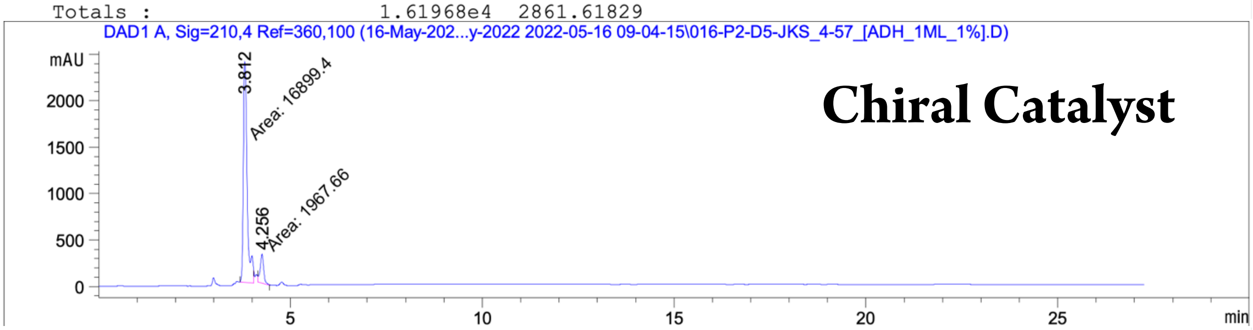

Signal 1: DAD1 A, Sig=210,4 Ref=360,100

Peak #	RetTime [min]	Type	Width [min]	Area [mAU*s]	Height [mAU]	Area %
1	3.812	MM	0.1187	1.68994e4	2372.62671	89.5709
2	4.256	MM	0.1060	1967.66187	309.25751	10.4291
Totals :				1.88671e4	2681.88422	

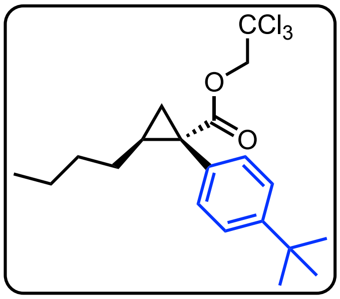

Compound **36**

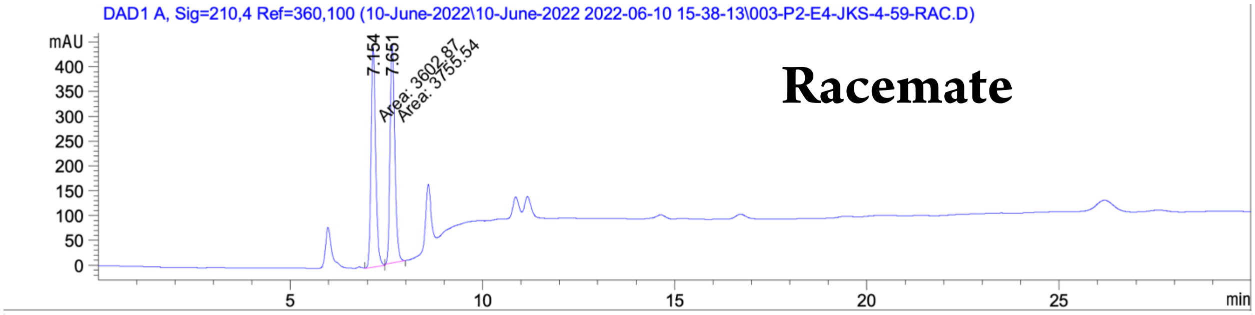


```
Signal 1: DAD1 A, Sig=210,4 Ref=360,100
```

Peak #	RetTime [min]	Type	Width [min]	Area [mAU*s]	Height [mAU]	Area %
1	7.154	MM	0.1355	3602.86768	443.24615	48.9626
2	7.651	MM	0.1414	3755.54053	442.57819	51.0374
Totals :				7358.40820	885.82434	

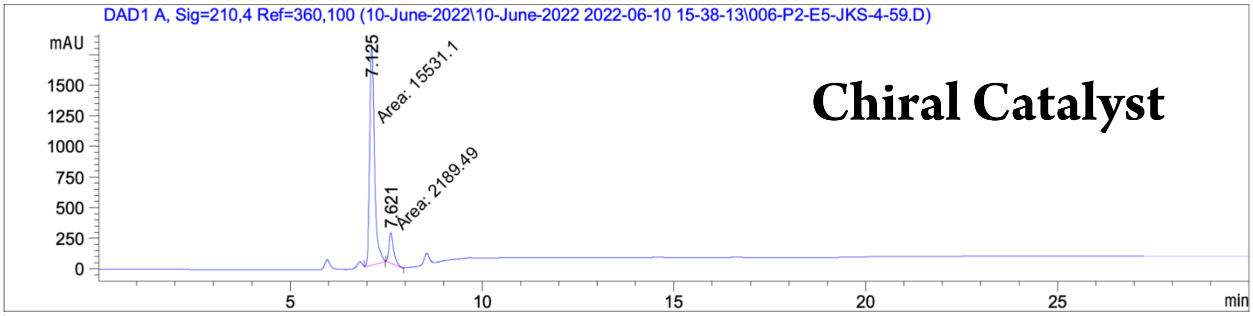


```
Signal 1: DAD1 A, Sig=210,4 Ref=360,100
```

Peak #	RetTime [min]	Type	Width [min]	Area [mAU*s]	Height [mAU]	Area %
1	7.125	MM	0.1439	1.55311e4	1798.51465	87.6444
2	7.621	MM	0.1465	2189.48853	249.00909	12.3556
Totals :				1.77206e4	2047.52374	

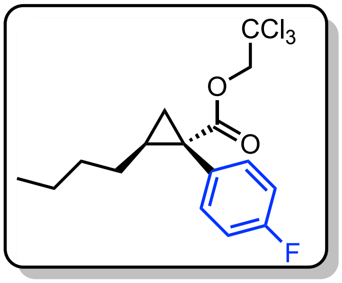

Compound **37**

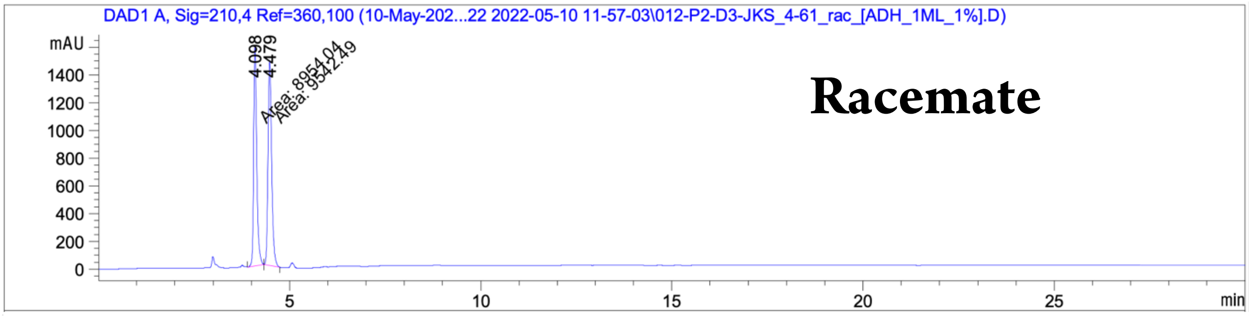

Signal 1: DAD1 A, Sig=210,4 Ref=360,100

Peak #	RetTime [min]	Type	Width [min]	Area [mAU*s]	Height [mAU]	Area %
1	4.098	MM	0.0938	8954.04492	1590.51208	48.4093
2	4.479	MM	0.1076	9542.48633	1477.88611	51.5907

Totals : 1.84965e4 3068.39819

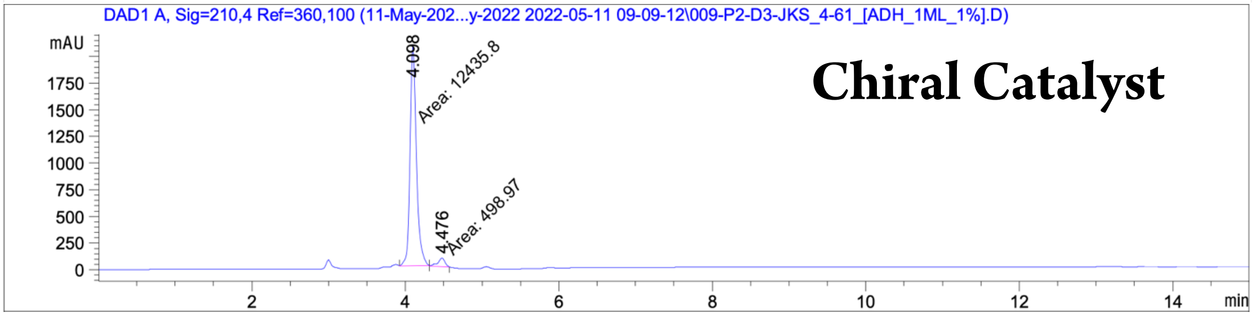

Peak #	RetTime [min]	Type	Width [min]	Area [mAU*s]	Height [mAU]	Area %
1	4.098	MM	0.1001	1.24358e4	2070.53101	96.1424
2	4.476	MM	0.1041	498.96985	79.91113	3.8576

Totals : 1.29348e4 2150.44214

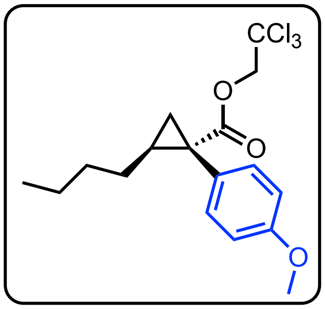

Compound **38**

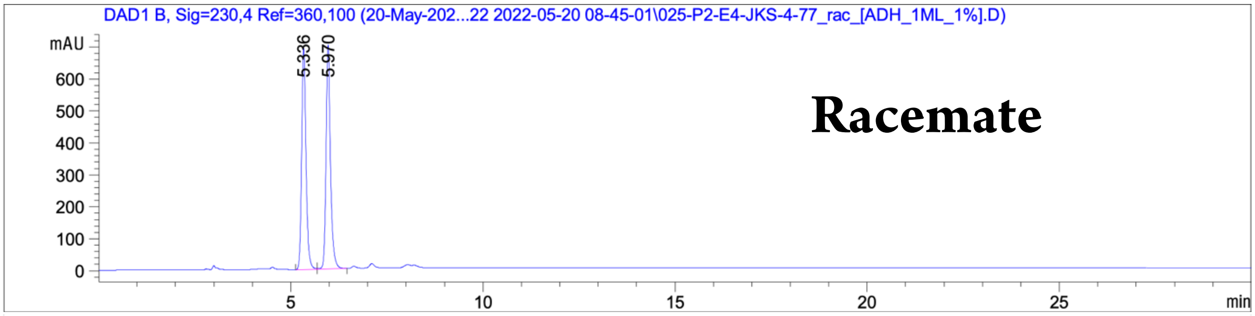

Signal 2: DAD1 B, Sig=230,4 Ref=360,100

Peak #	RetTime [min]	Type	Width [min]	Area [mAU*s]	Height [mAU]	Area %
1	5.336	BV	0.1160	5351.30273	693.85547	48.3801
2	5.970	VV	0.1230	5709.66260	701.35504	51.6199

Totals : 1.10610e4 1395.21051

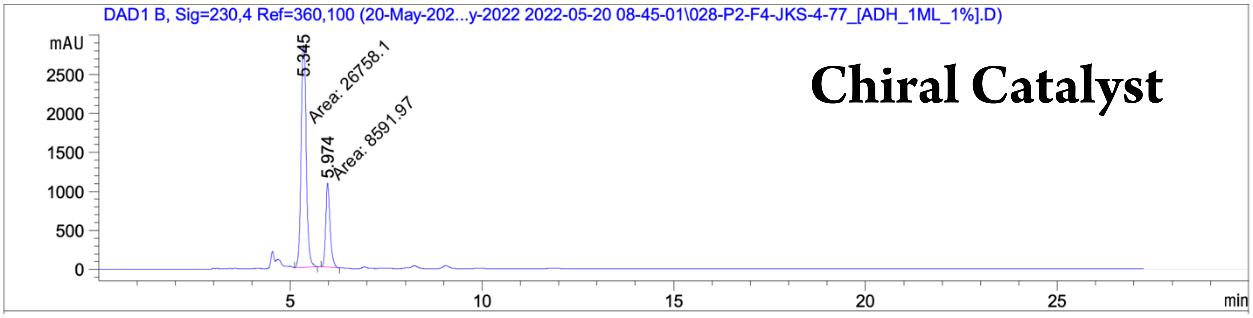

Signal 2: DAD1 B, Sig=230,4 Ref=360,100

Peak #	RetTime [min]	Type	Width [min]	Area [mAU*s]	Height [mAU]	Area %
1	5.345	MM	0.1563	2.67581e4	2853.75342	75.6946
2	5.974	MM	0.1326	8591.97461	1080.03687	24.3054

Totals : 3.53501e4 3933.79028

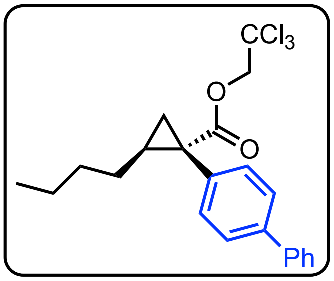

Compound **39**

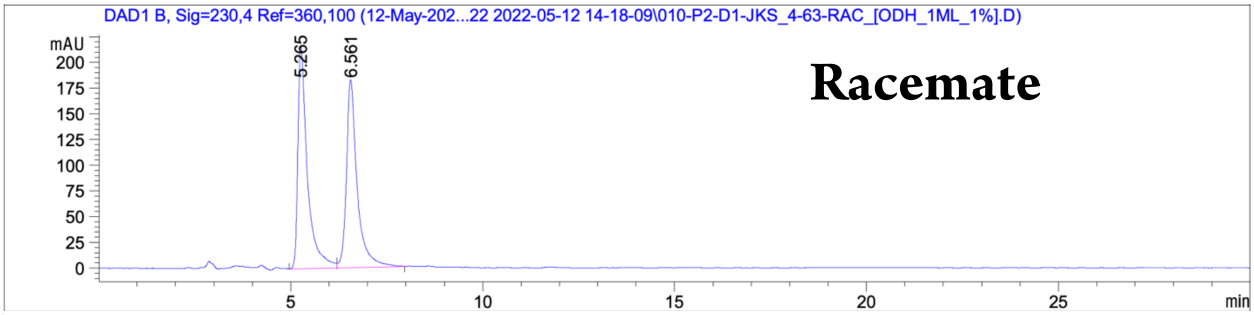

Signal 2: DAD1 B, Sig=230,4 Ref=360,100

Peak #	RetTime [min]	Type	Width [min]	Area [mAU*s]	Height [mAU]	Area %
1	5.265	BV	0.2455	3705.11035	216.69075	51.0951
2	6.561	VB	0.2801	3546.28491	183.00114	48.9049
Totals :				7251.39526	399.69189	

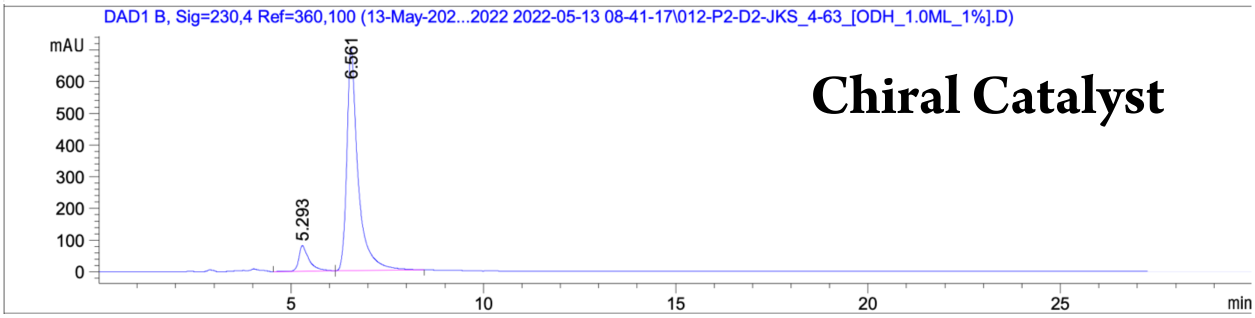

Signal 2: DAD1 B, Sig=230,4 Ref=360,100

Peak #	RetTime [min]	Type	Width [min]	Area [mAU*s]	Height [mAU]	Area %
1	5.293	BV	0.2642	1563.71777	81.38562	9.8170
2	6.561	VB	0.2916	1.43649e4	705.43890	90.1830
Totals :				1.59286e4	786.82452	

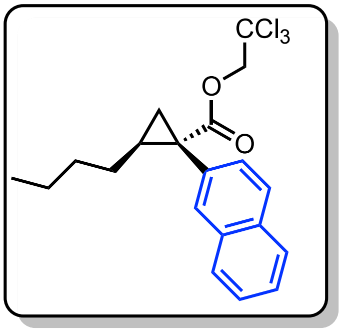

Compound **40**

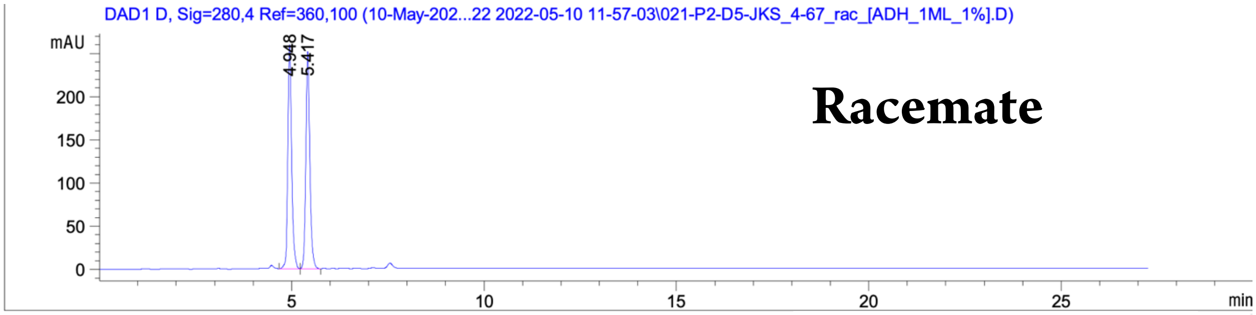


```
Signal 4: DAD1 D, Sig=280,4 Ref=360,100

Peak RetTime Type  Width     Area      Height     Area
  #   [min]        [min]   [mAU*s]     [mAU]        %
----|-------|----|-------|----------|----------|--------|
   1   4.948 BB    0.1040 1782.91492  260.08020  48.9374
   2   5.417 BB    0.1123 1860.34387  251.58144  51.0626

Totals :                  3643.25879  511.66164
```

Chiral Catalyst

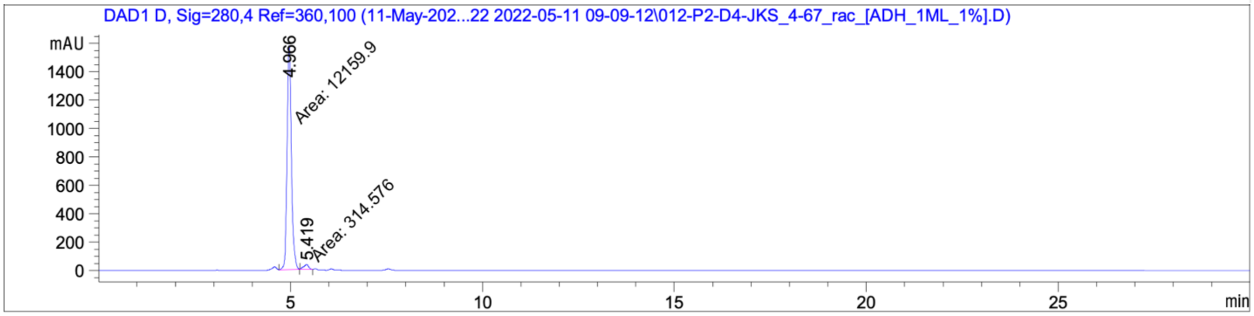


```
Signal 4: DAD1 D, Sig=280,4 Ref=360,100

Peak RetTime Type  Width     Area      Height     Area
  #   [min]        [min]   [mAU*s]     [mAU]        %
----|-------|----|-------|----------|----------|--------|
   1   4.966 MM    0.1282 1.21599e4  1580.68201  97.4782
   2   5.419 MM    0.1499  314.57635   34.97344   2.5218

Totals :                  1.24744e4  1615.65545
```

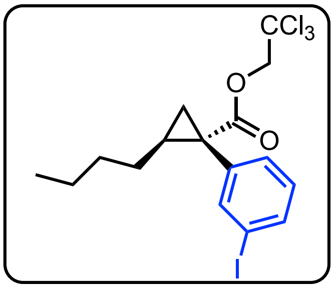

Compound **41**

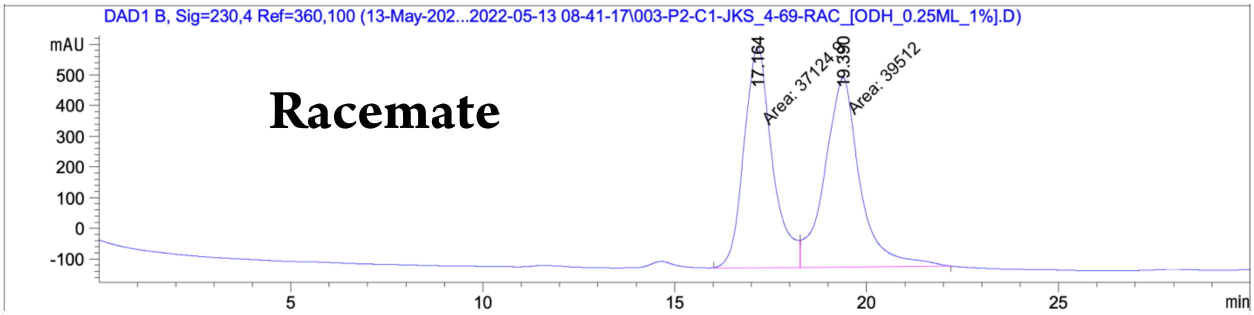

Signal 2: DAD1 B, Sig=230,4 Ref=360,100

Peak #	RetTime [min]	Type	Width [min]	Area [mAU*s]	Height [mAU]	Area %
1	17.164	MF	0.8576	3.71249e4	721.49799	48.4426
2	19.390	FM	1.0662	3.95120e4	617.63428	51.5574
Totals :				7.66369e4	1339.13226	

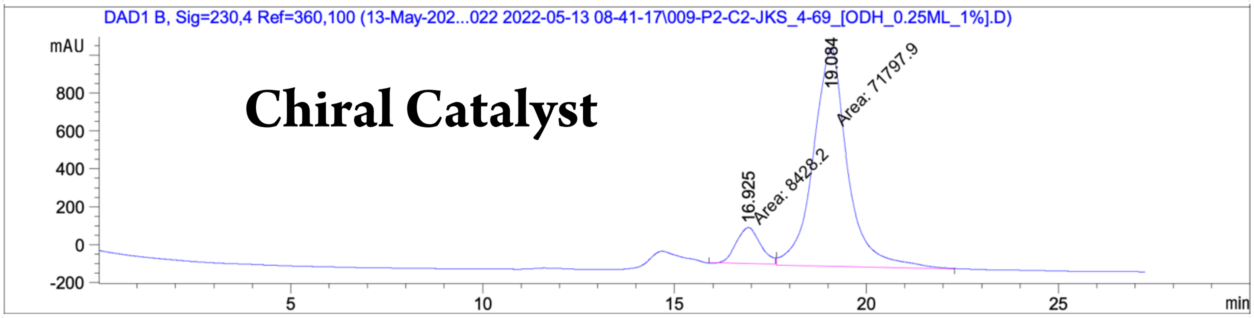

Signal 2: DAD1 B, Sig=230,4 Ref=360,100

Peak #	RetTime [min]	Type	Width [min]	Area [mAU*s]	Height [mAU]	Area %
1	16.925	MM	0.7379	8428.19922	190.36365	10.5056
2	19.084	MM	1.0385	7.17979e4	1152.28149	89.4944
Totals :				8.02261e4	1342.64514	

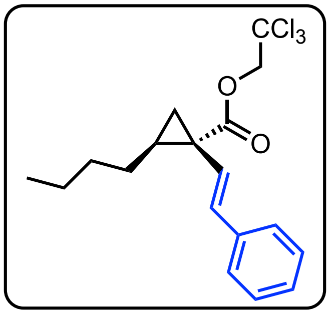

Compound **42**

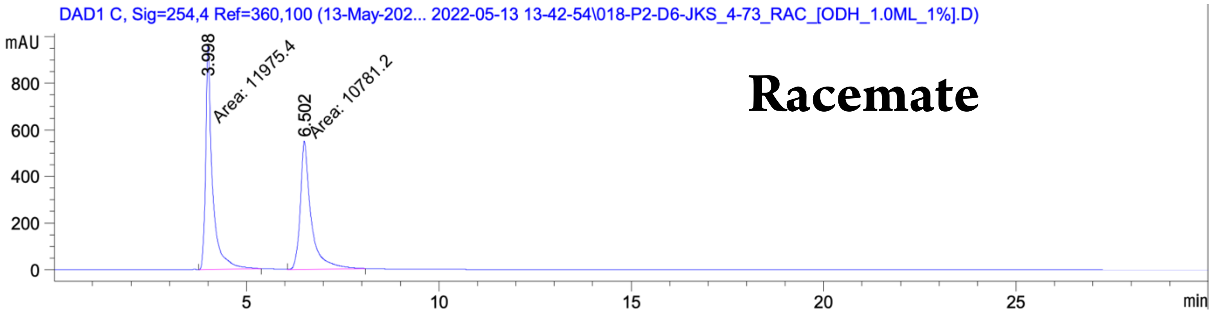

Signal 3: DAD1 C, Sig=254,4 Ref=360,100

Peak #	RetTime [min]	Type	Width [min]	Area [mAU*s]	Height [mAU]	Area %
1	3.998	MM	0.2064	1.19754e4	966.92511	52.6238
2	6.502	MM	0.3262	1.07812e4	550.84894	47.3762
Totals :				2.27565e4	1517.77405	

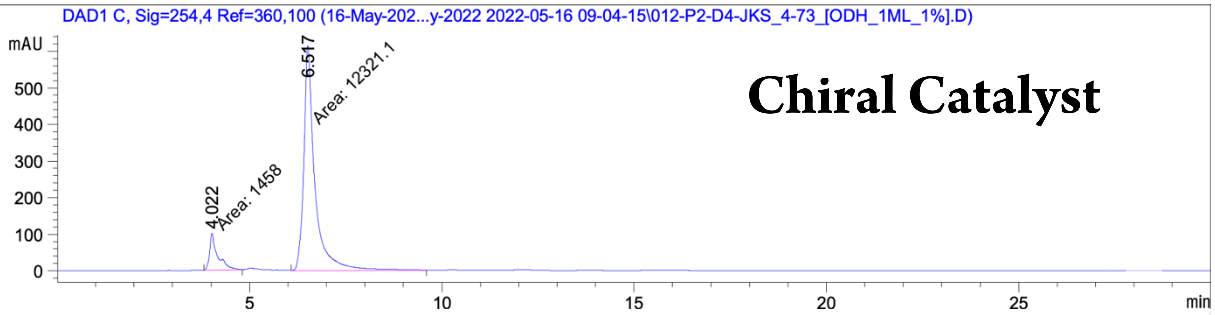

Signal 3: DAD1 C, Sig=254,4 Ref=360,100

Peak #	RetTime [min]	Type	Width [min]	Area [mAU*s]	Height [mAU]	Area %
1	4.022	MM	0.2419	1457.99976	100.45798	10.5812
2	6.517	MM	0.3345	1.23211e4	613.97955	89.4188
Totals :				1.37791e4	714.43753	

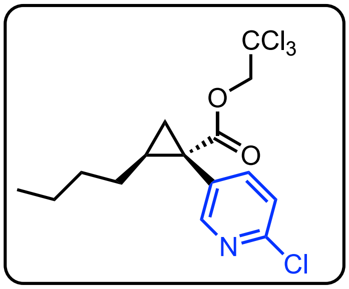

Compound **43**

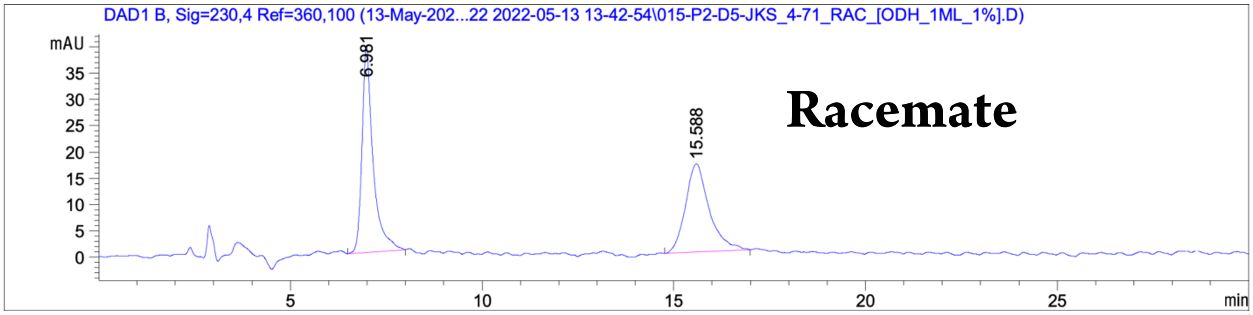

Signal 2: DAD1 B, Sig=230,4 Ref=360,100

Peak #	RetTime [min]	Type	Width [min]	Area [mAU*s]	Height [mAU]	Area %
1	6.981	BB	0.2864	791.49957	39.40199	52.2334
2	15.588	BB	0.6274	723.81415	16.77467	47.7666
Totals :				1515.31372	56.17666	

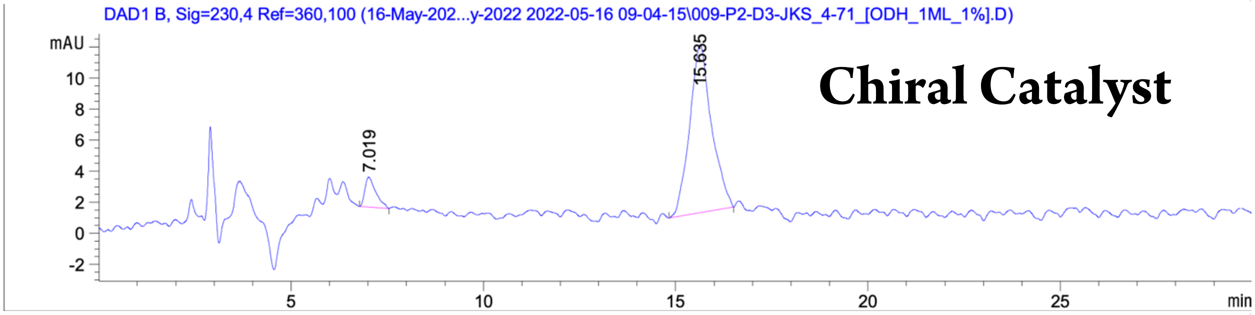

Signal 2: DAD1 B, Sig=230,4 Ref=360,100

Peak #	RetTime [min]	Type	Width [min]	Area [mAU*s]	Height [mAU]	Area %
1	7.019	BB	0.2797	38.09735	1.93466	8.1562
2	15.635	BB	0.5507	428.99942	10.81679	91.8438
Totals :				467.09677	12.75145	

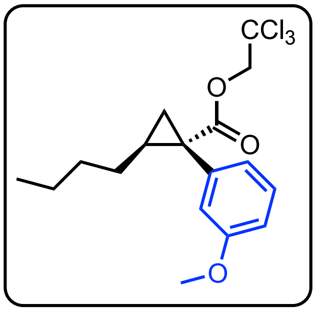

Compound **44**

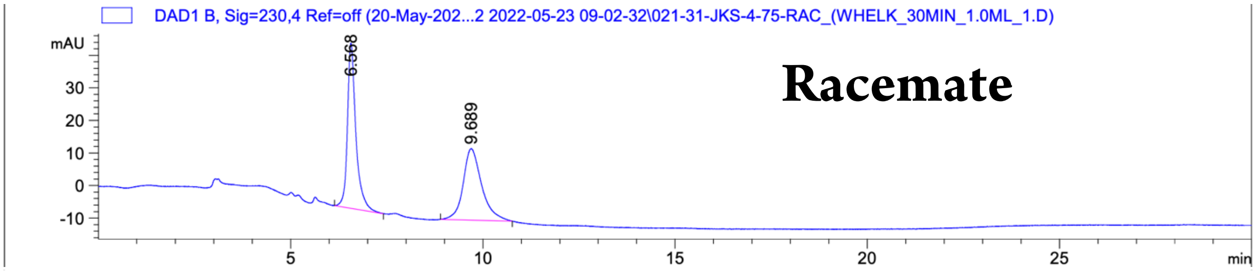

Signal 2: DAD1 B, Sig=230,4 Ref=off

Peak #	RetTime [min]	Type	Width [min]	Area [mAU*s]	Height [mAU]	Area %
1	6.568	BV R	0.2142	777.20831	50.73787	51.2671
2	9.689	VB R	0.3941	738.78949	21.95587	48.7329
Totals :				1515.99780	72.69374	

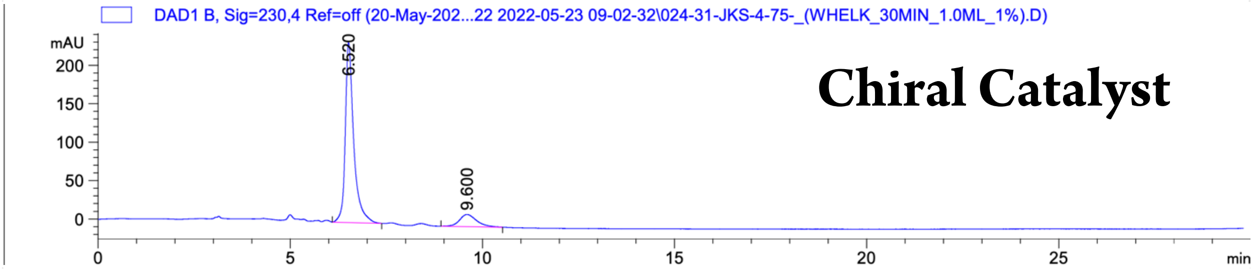

Signal 2: DAD1 B, Sig=230,4 Ref=off

Peak #	RetTime [min]	Type	Width [min]	Area [mAU*s]	Height [mAU]	Area %
1	6.520	BB	0.2230	3580.29175	234.00452	87.8815
2	9.600	VV R	0.3647	493.70880	15.92250	12.1185
Totals :				4074.00055	249.92702	

HPLC Chromatographs for React IR Experiments

React IR experiments varying the equivalents of styrene:

5.0 equivalents of styrene, 1.0mol % catalyst loading.

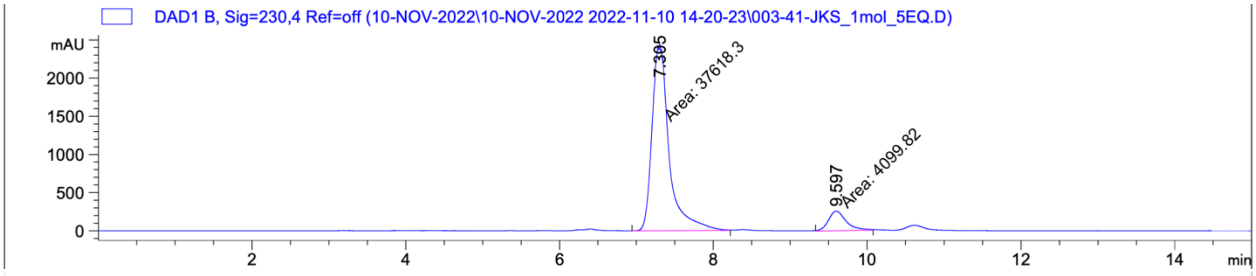

Signal 2: DAD1 B, Sig=230,4 Ref=off

Peak #	RetTime [min]	Type	Width [min]	Area [mAU*s]	Height [mAU]	Area %
1	7.305	MM	0.2570	3.76183e4	2440.00342	90.1726
2	9.597	MM	0.2671	4099.82324	255.80693	9.8274
Totals :				4.17181e4	2695.81035	

3.0 equivalents of styrene, 1.0mol % catalyst loading.

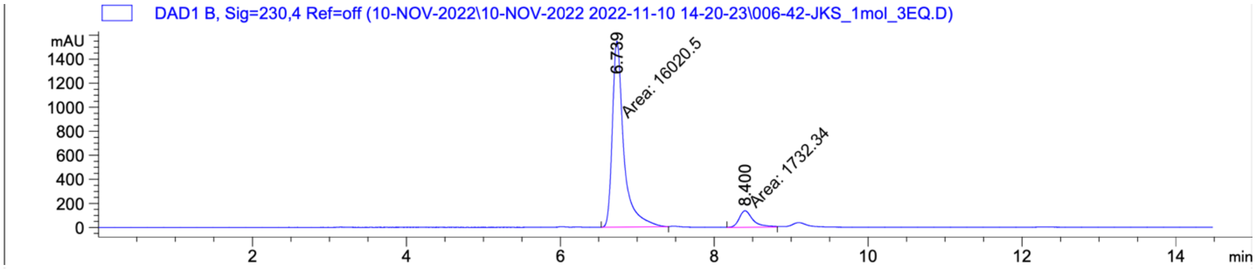

Signal 2: DAD1 B, Sig=230,4 Ref=off

Peak #	RetTime [min]	Type	Width [min]	Area [mAU*s]	Height [mAU]	Area %
1	6.739	MM	0.1722	1.60205e4	1550.22693	90.2419
2	8.400	MM	0.2082	1732.33948	138.68996	9.7581
Totals :				1.77528e4	1688.91689	

1.5 equivalents of styrene, 1.0mol % catalyst loading.

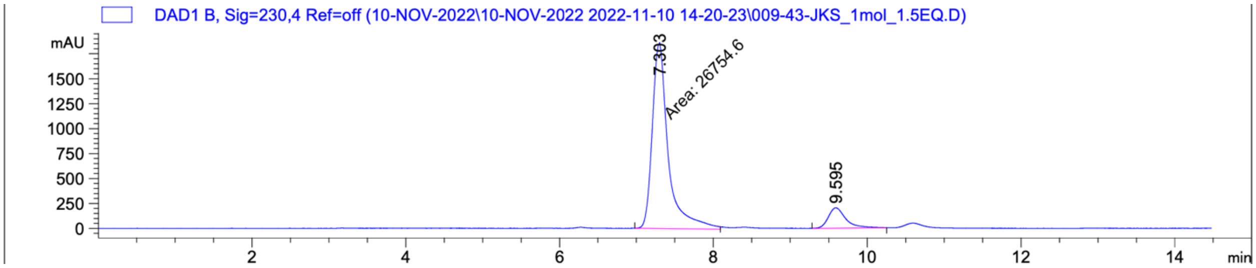

Signal 2: DAD1 B, Sig=230,4 Ref=off

Peak #	RetTime [min]	Type	Width [min]	Area [mAU*s]	Height [mAU]	Area %
1	7.303	MM	0.2392	2.67546e4	1864.29041	89.2488
2	9.595	BV R	0.1873	3222.95435	203.96759	10.7512
Totals :				2.99775e4	2068.25800	

React IR experiments varying the catalyst loading

3.0 equivalents of styrene, **0.5mol %** catalyst loading.

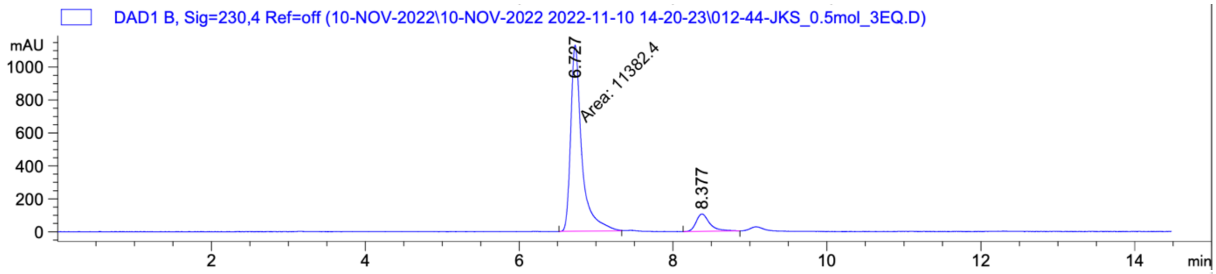

Signal 2: DAD1 B, Sig=230,4 Ref=off

Peak #	RetTime [min]	Type	Width [min]	Area [mAU*s]	Height [mAU]	Area %
1	6.727	MM	0.1678	1.13824e4	1130.75708	89.8516
2	8.377	BV R	0.1437	1285.59961	105.85095	10.1484
Totals :				1.26680e4	1236.60803	

3.0 equivalents of styrene, **0.1 mol %** catalyst loading.

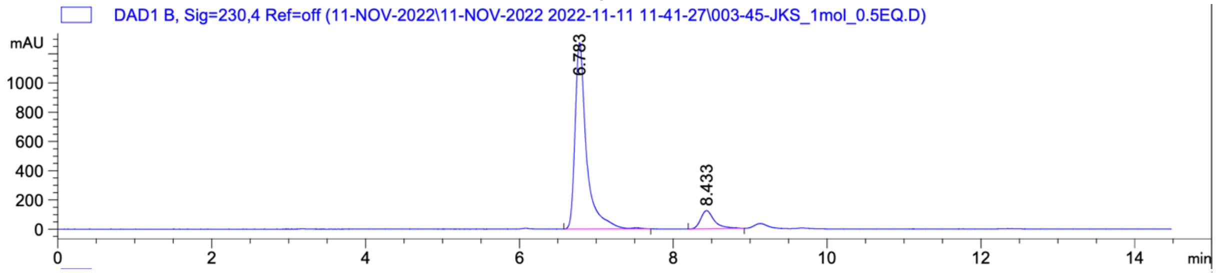

Signal 2: DAD1 B, Sig=230,4 Ref=off

Peak #	RetTime [min]	Type	Width [min]	Area [mAU*s]	Height [mAU]	Area %
1	6.783	BV R	0.1393	1.32190e4	1275.17456	89.6836
2	8.433	VV R	0.1499	1520.59167	124.54695	10.3164
Totals :				1.47396e4	1399.72151	

NMR Spectra of Novel Compounds

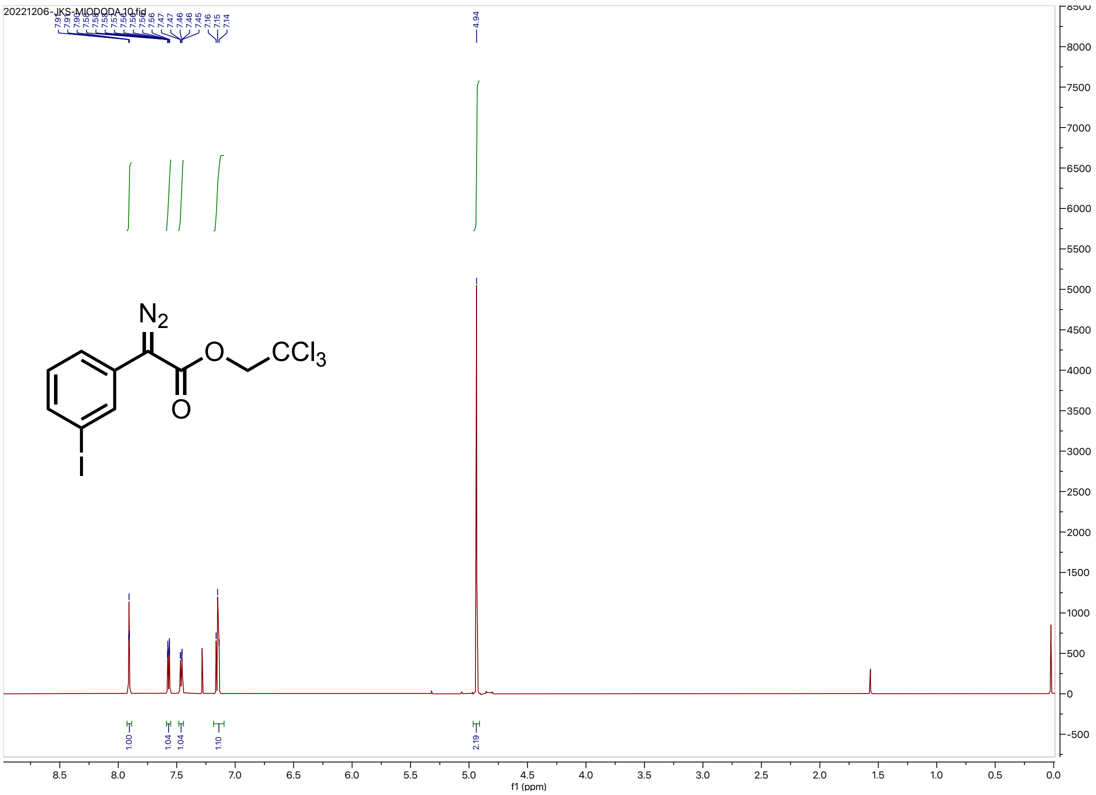

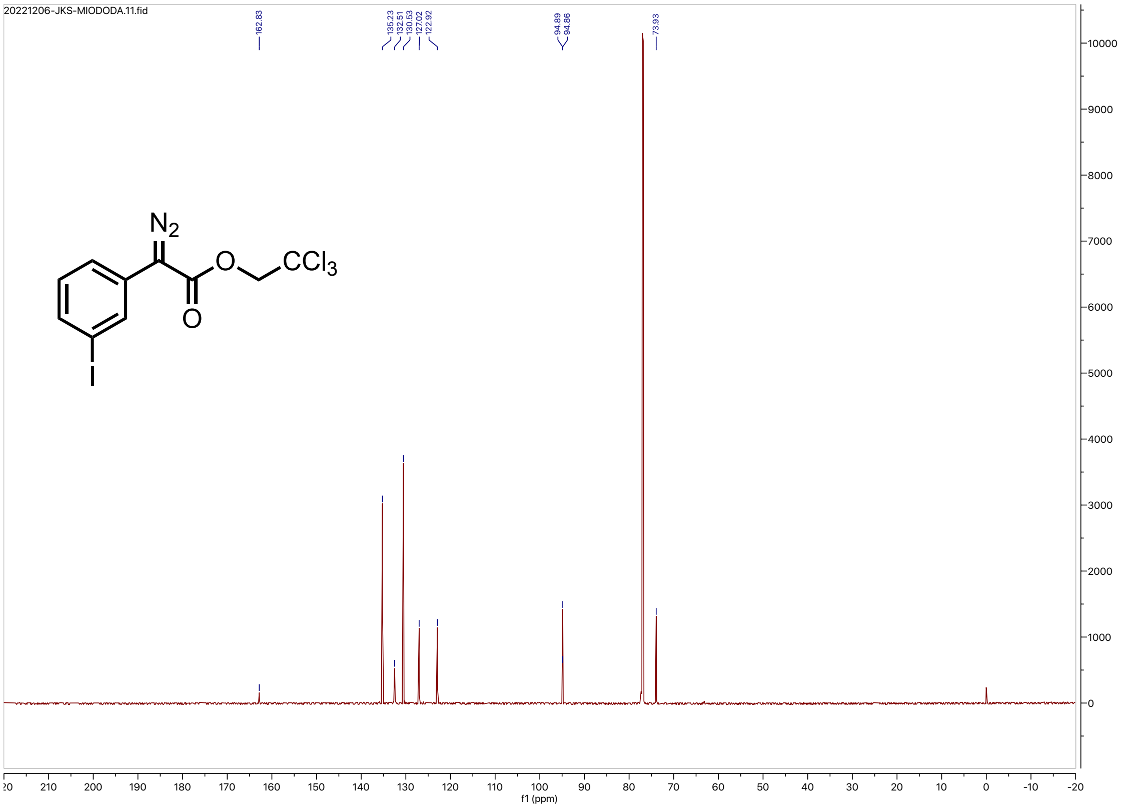

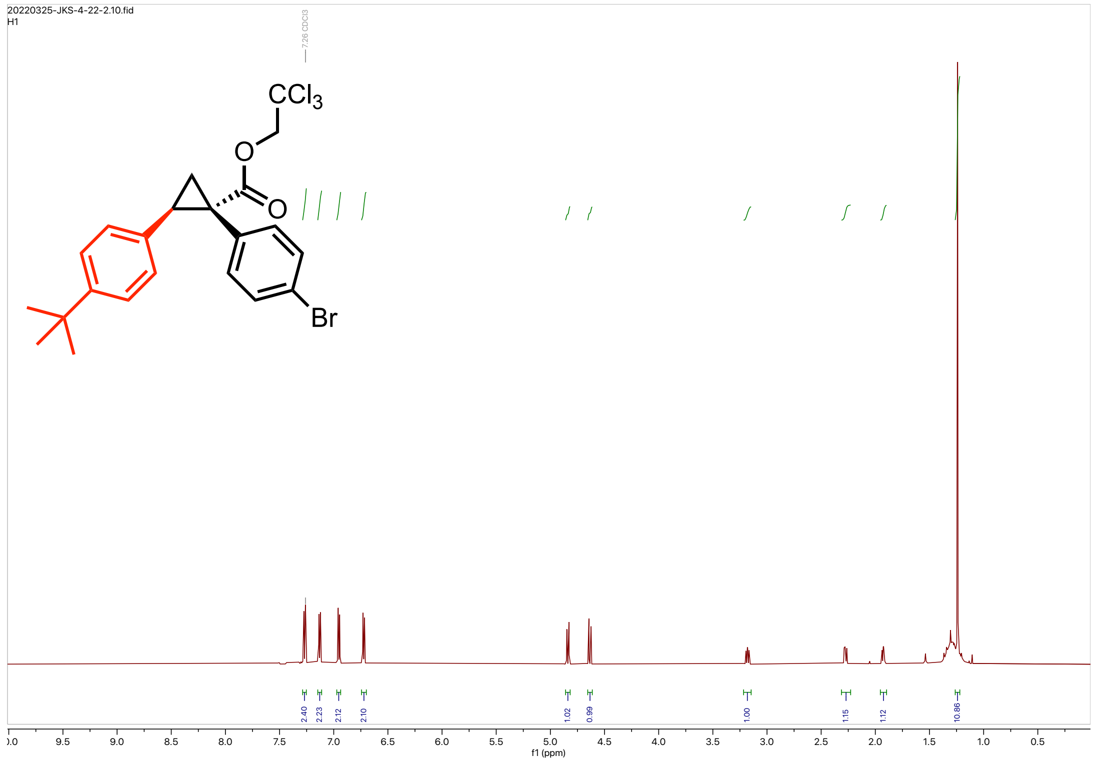

20220325-JKS-4-22-2.10.fid
H1
7.26 CDCl3
CCl3
O
O
Br
2.40
2.23
2.12
2.10
1.02
0.99
1.00
1.15
1.12
10.86
f1 (ppm)

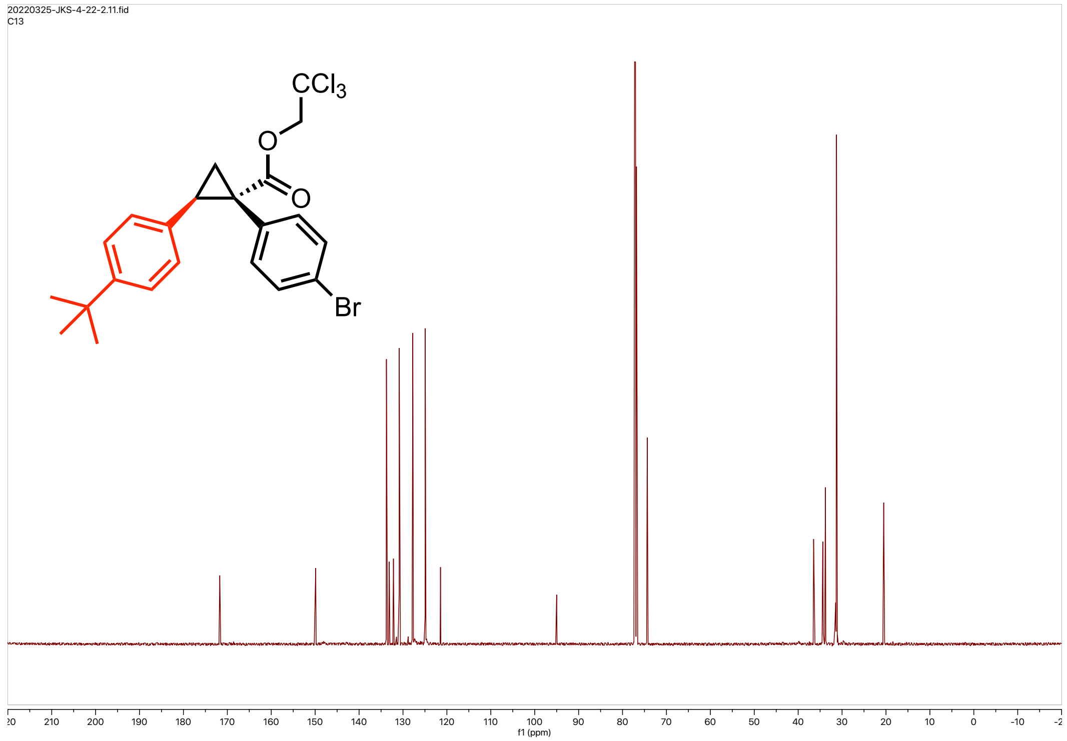

20220325-JKS-4-22-2.11.fid
C13
CCl3
O
O
Br
f1 (ppm)

20220318-JKS-4-29-clean.10.fid

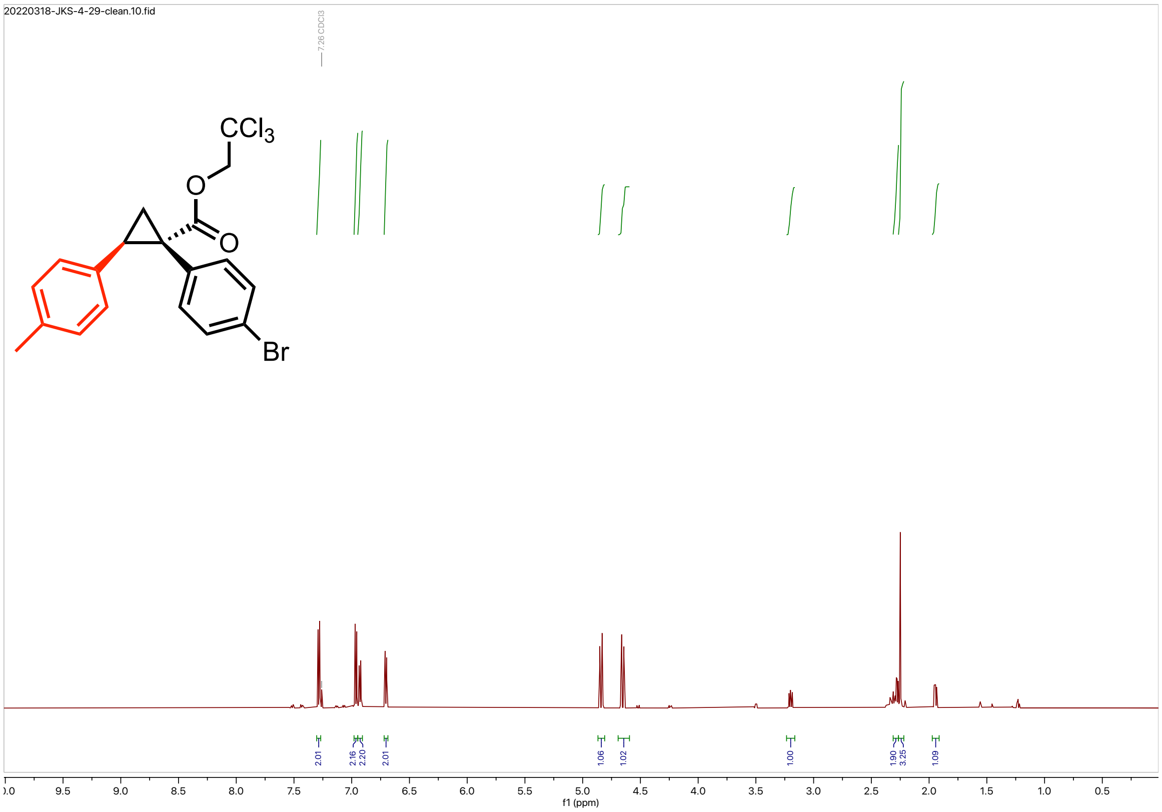

20220318-JKS-4-29-clean.11.fid

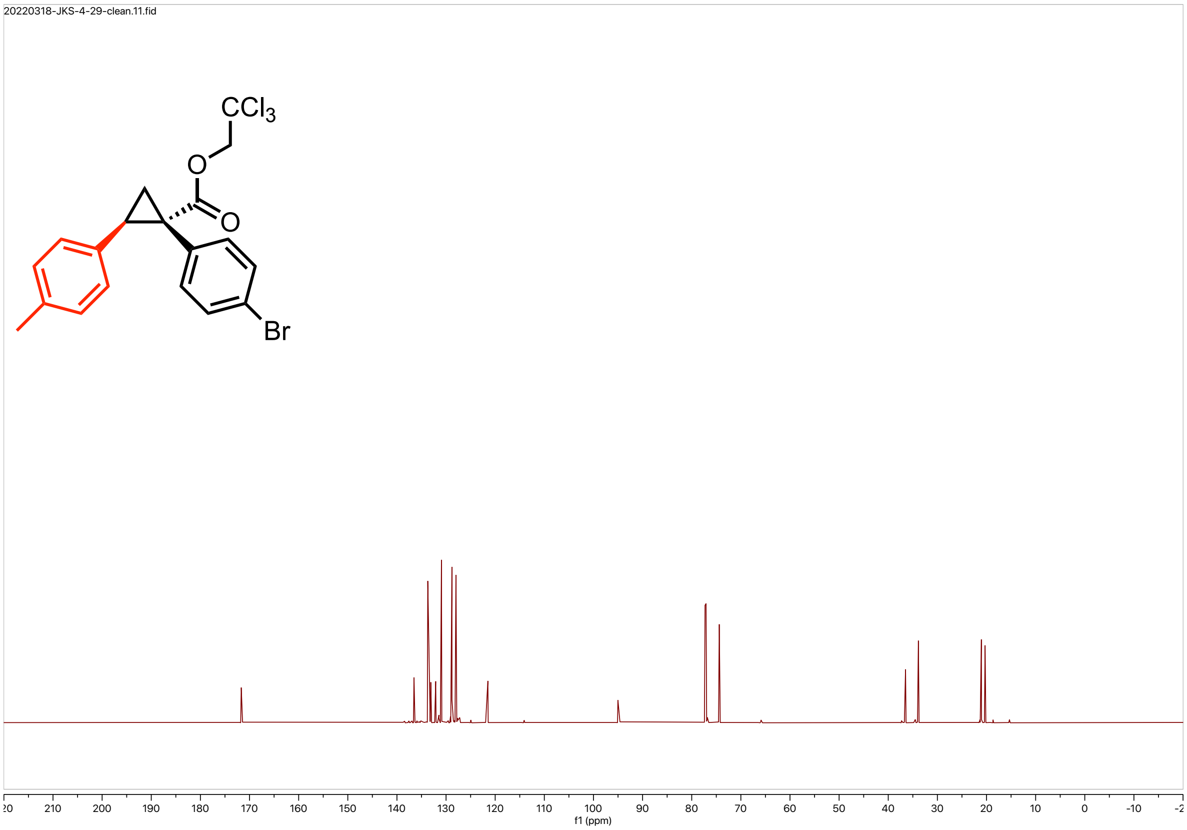

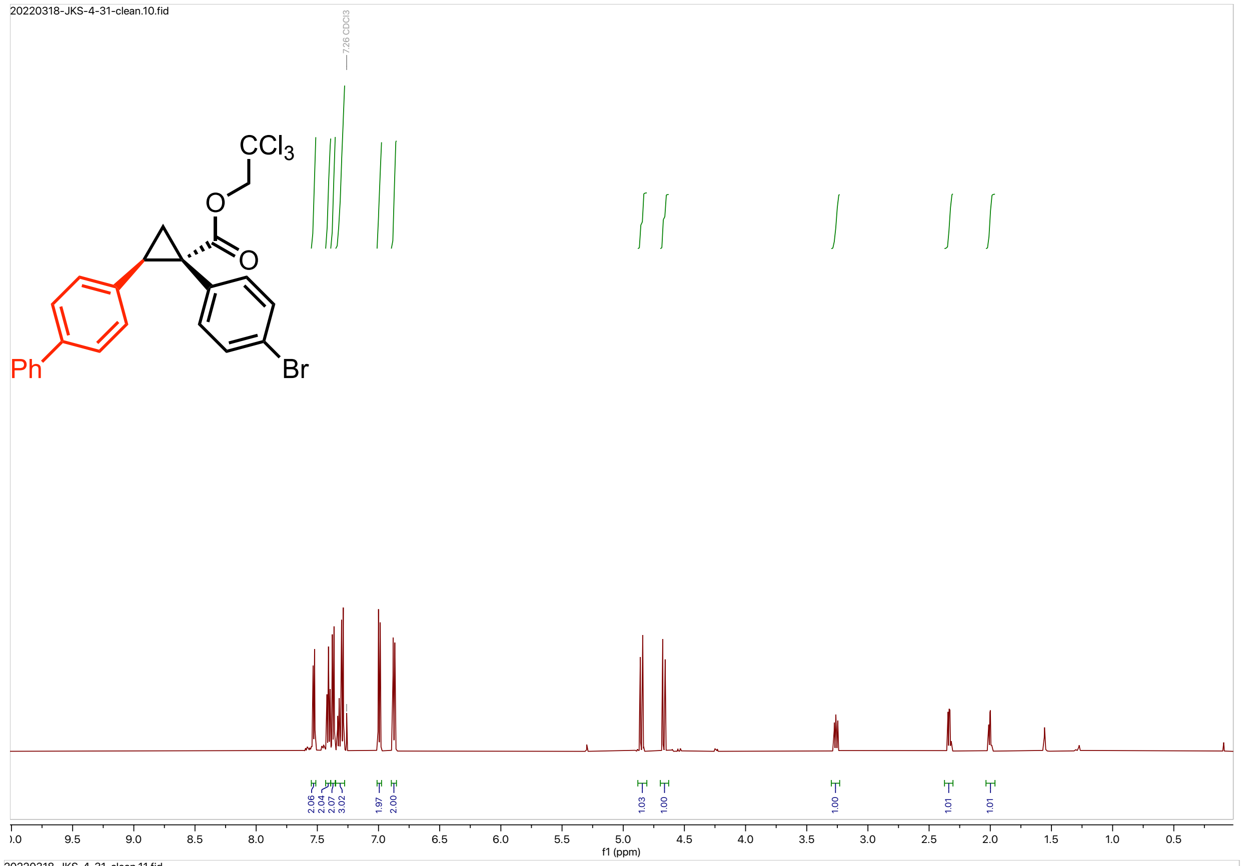
20220318-JKS-4-31-clean.10.fid
7.26 CDCl3
CCl3
O
O
Ph
Br
2.06
2.04
2.07
3.02
1.97
2.00
1.03
1.00
1.00
1.01
1.01
f1 (ppm)

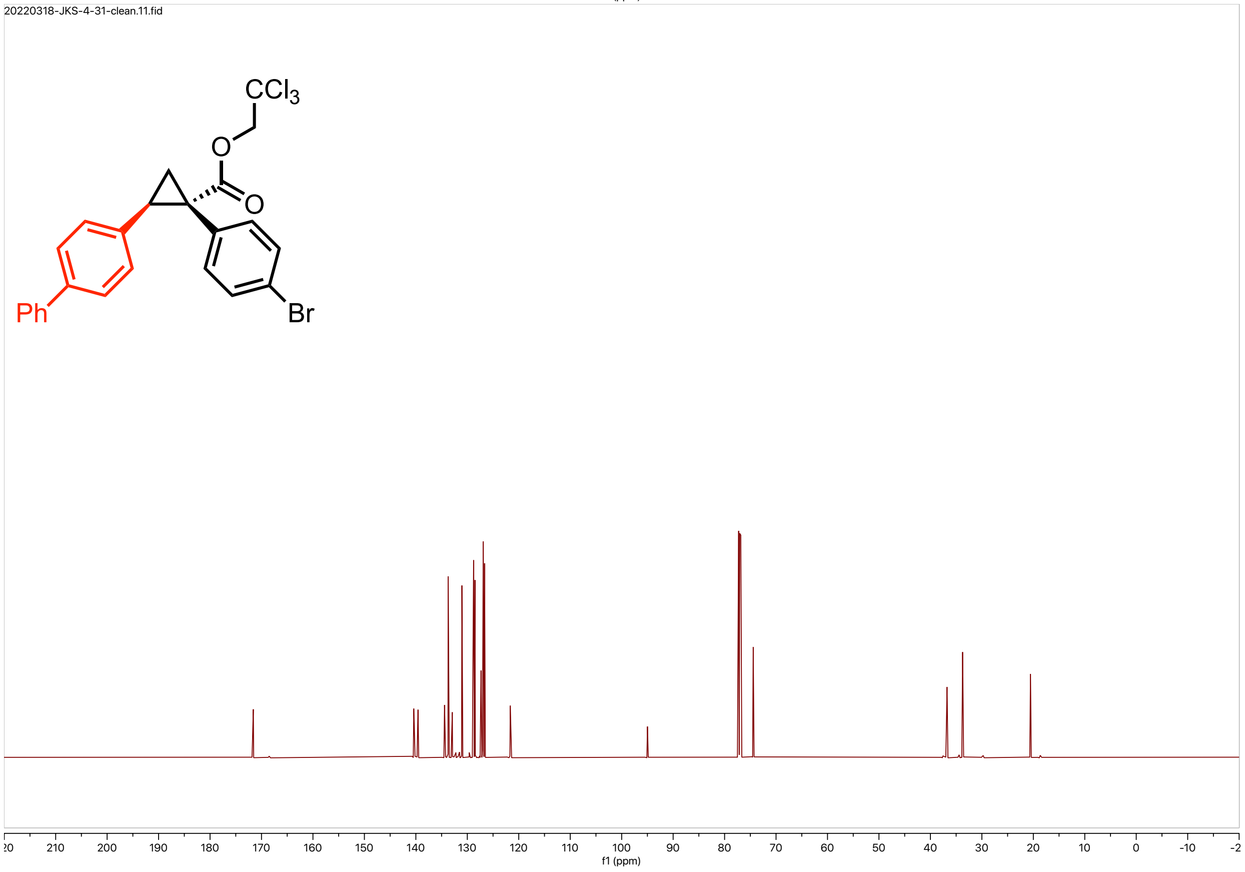
20220318-JKS-4-31-clean.11.fid
CCl3
O
O
Ph
Br
f1 (ppm)

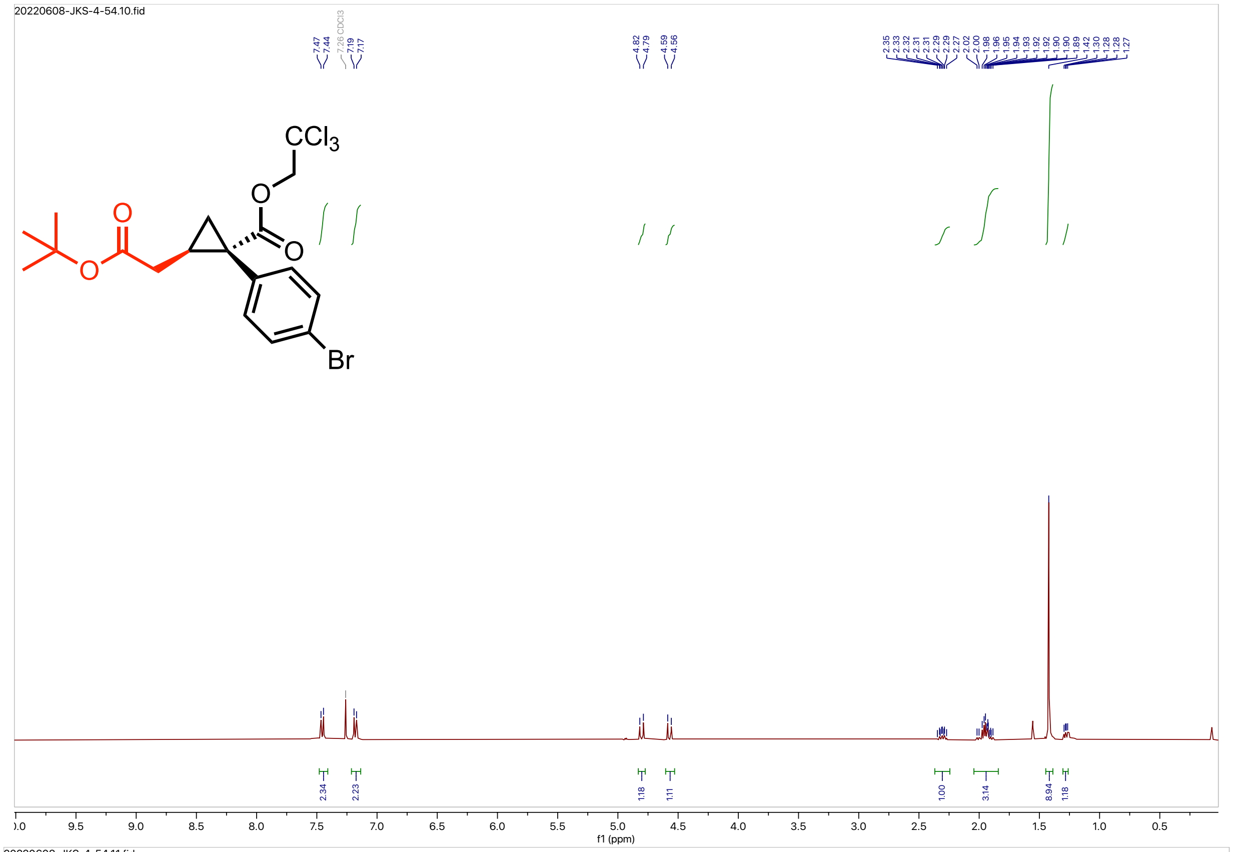
20220608-JKS-4-54.10.fid
f1 (ppm)

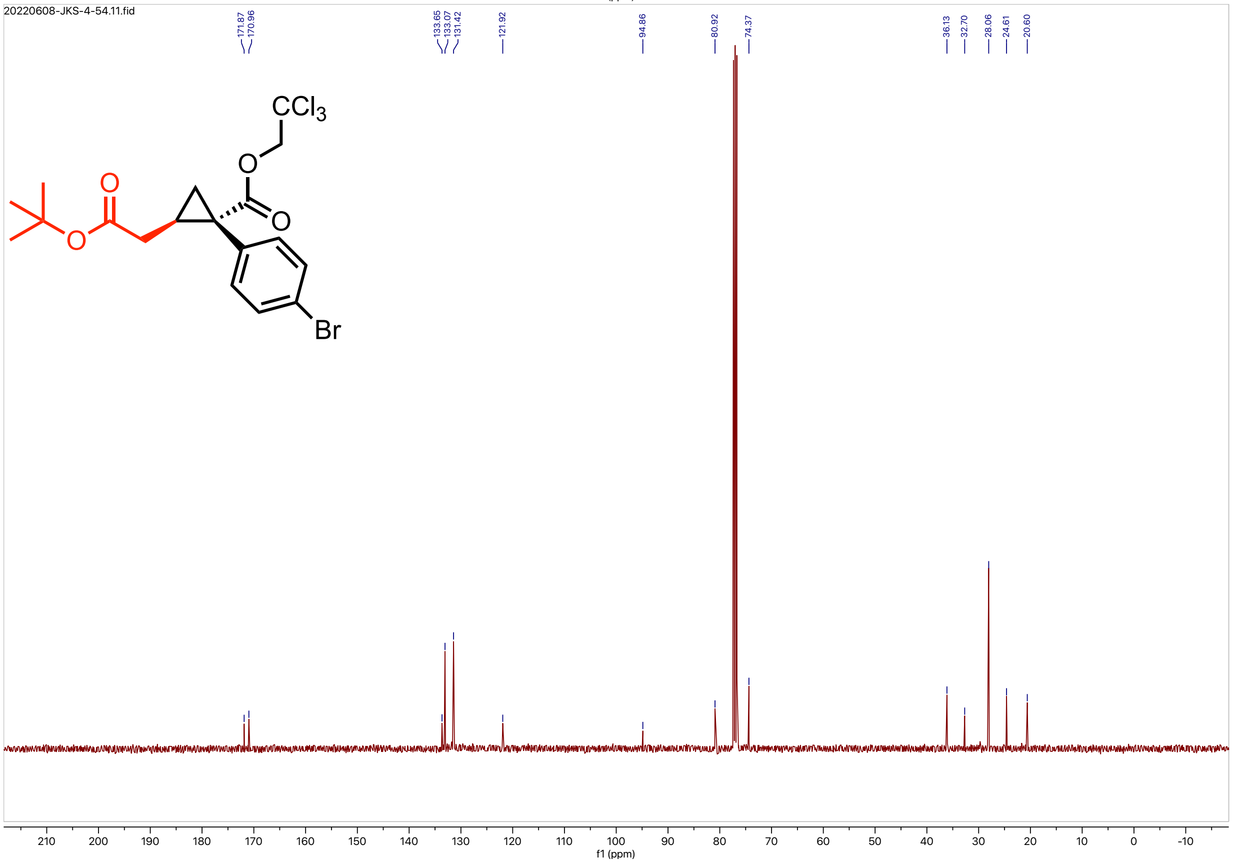
20220608-JKS-4-54.11.fid
f1 (ppm)

20220601-JKS-4-62-CLEAN.10.fid

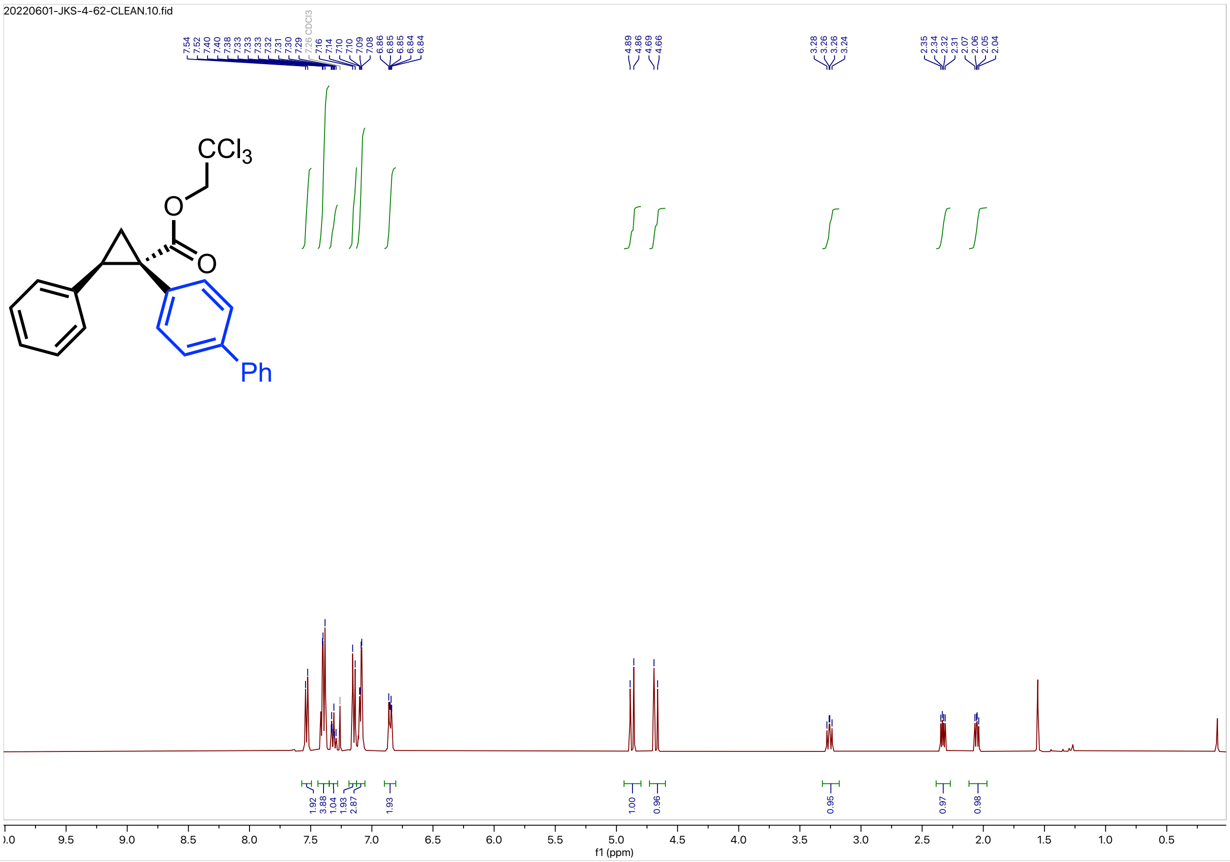

20220601-JKS-4-62_C13.10.fid

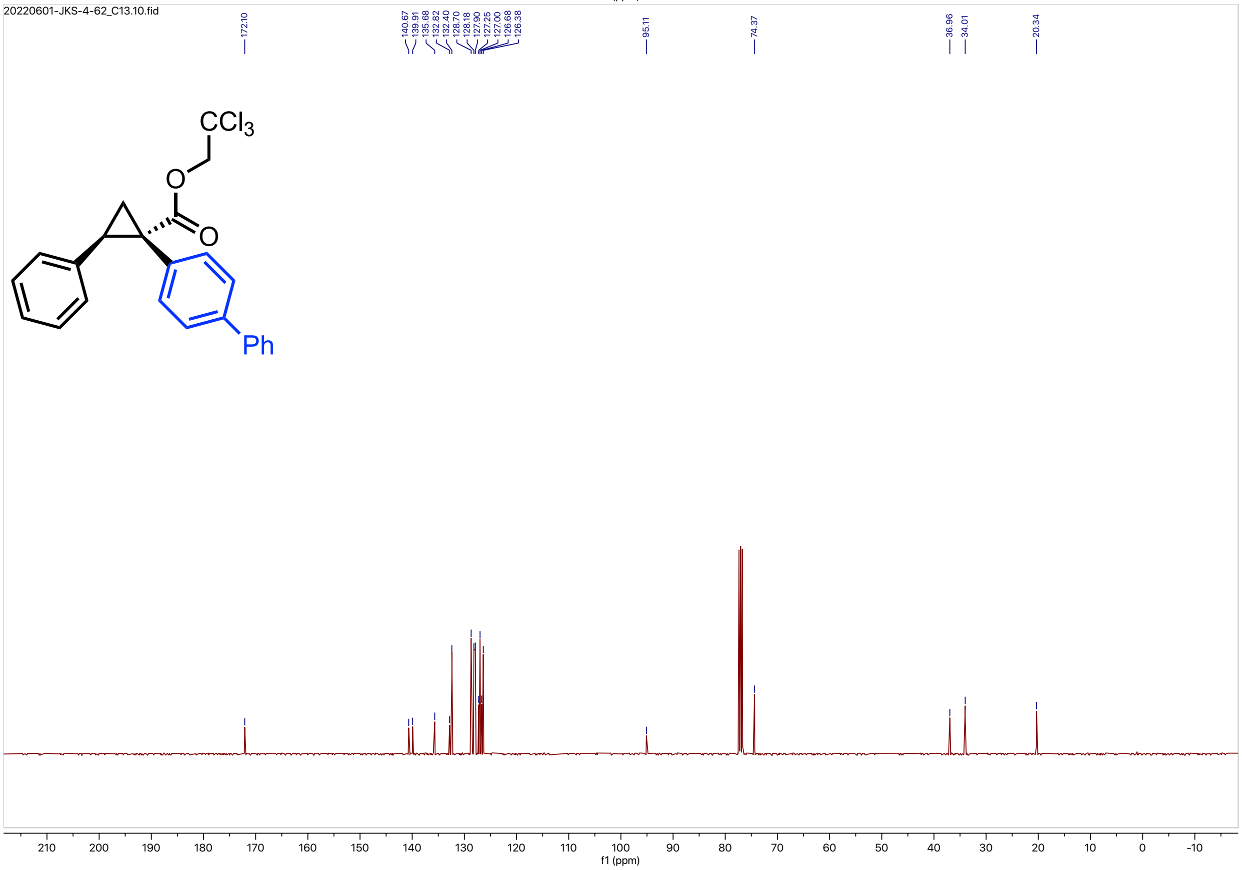

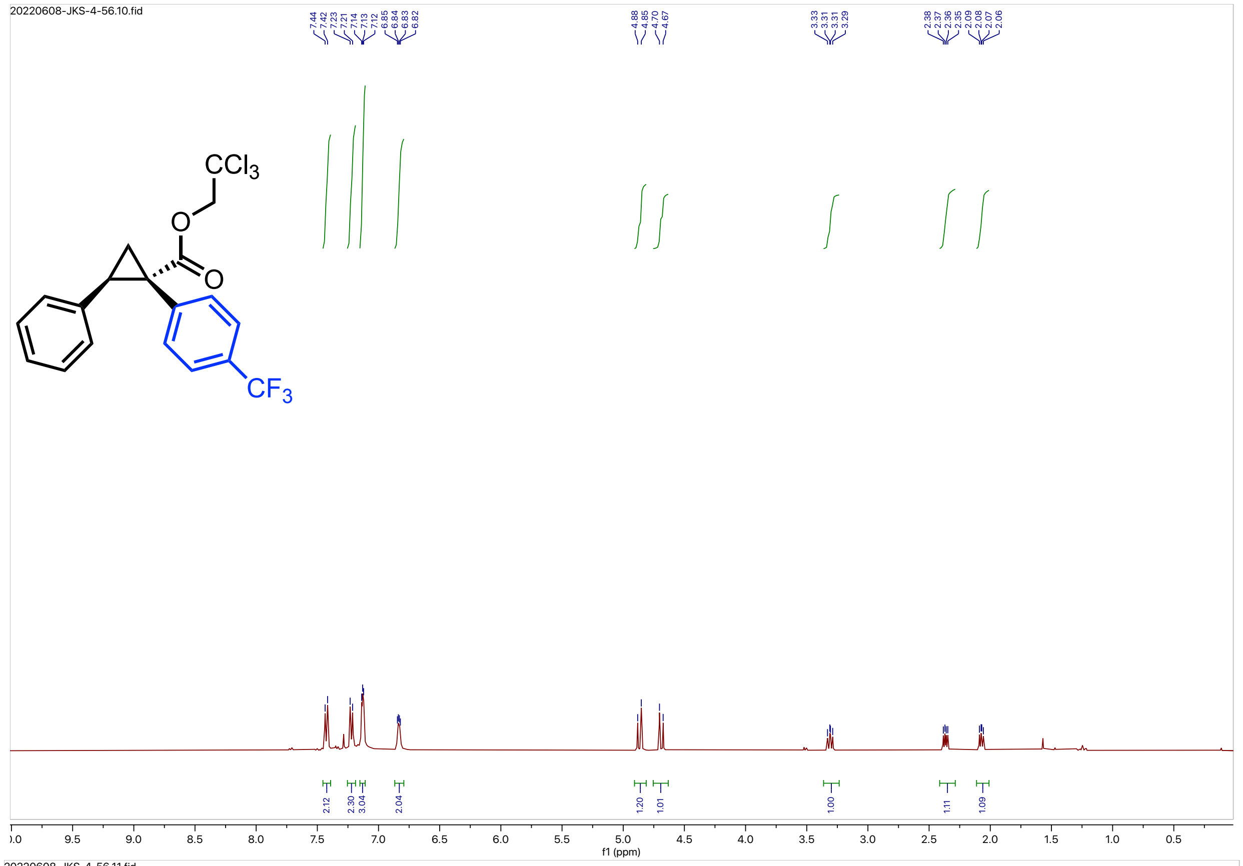
20220608-JKS-4-56.10.fid
CCl3
O
O
CF3
f1 (ppm)

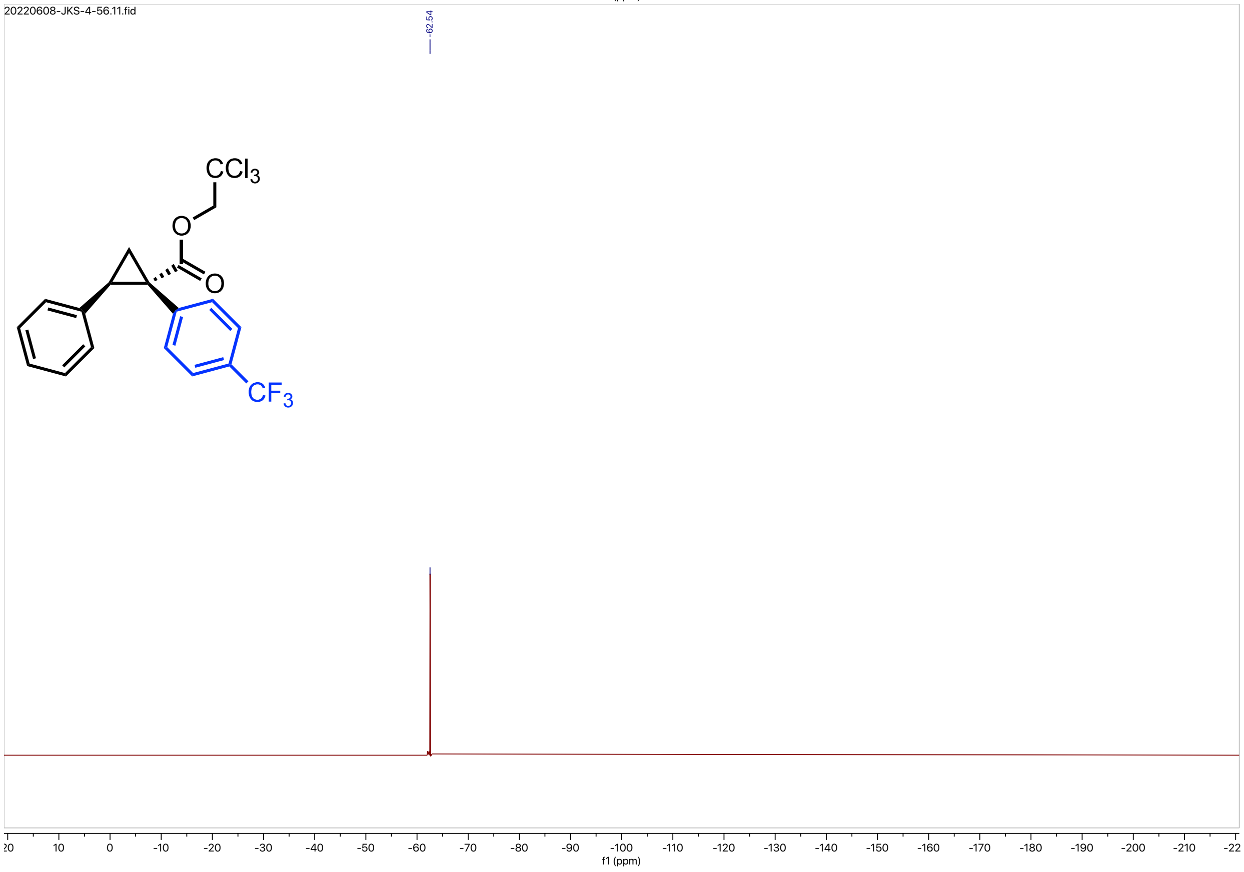
20220608-JKS-4-56.11.fid
-62.54
CCl3
O
O
CF3
f1 (ppm)

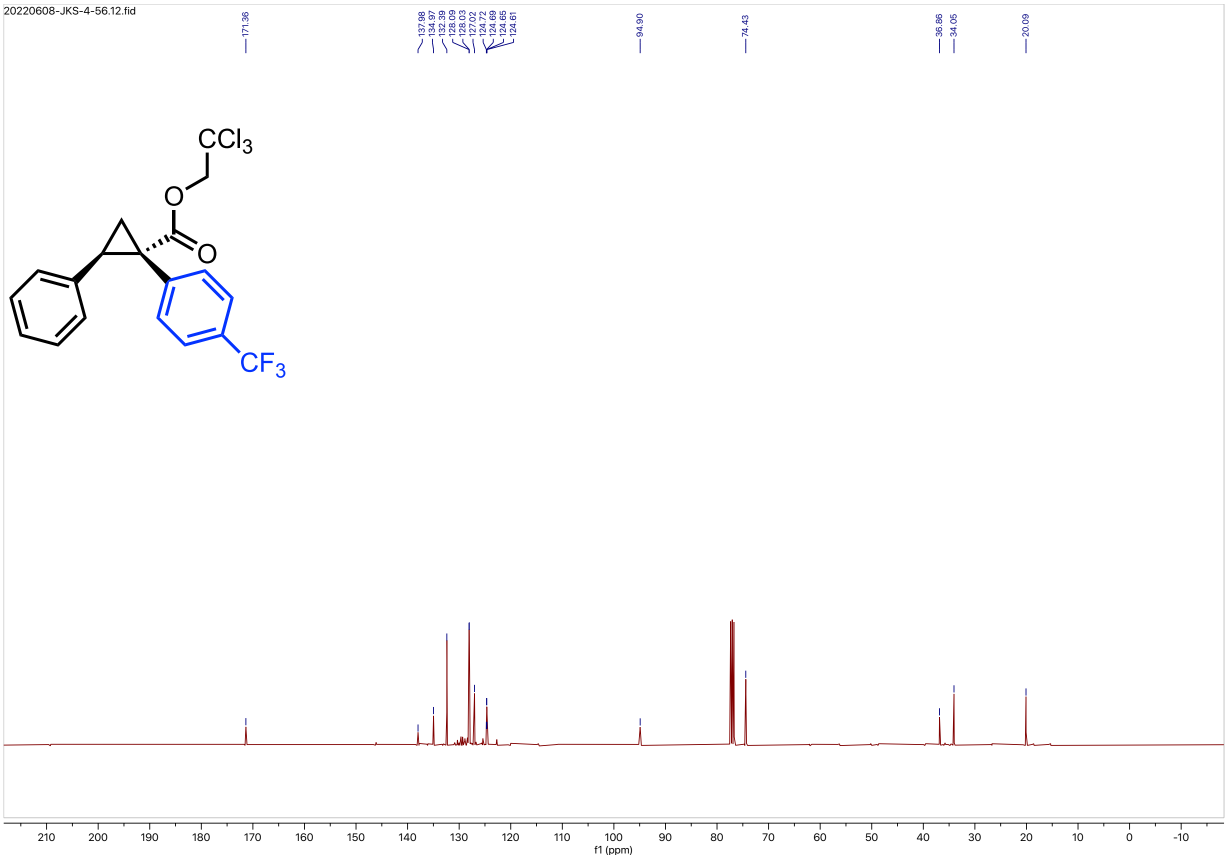
20220608-JKS-4-56.12.fid
171.36
137.98
134.97
132.39
128.09
128.03
127.02
124.72
124.69
124.65
124.61
94.90
74.43
36.86
34.05
20.09
CCl3
O
O
CF3
210
200
190
180
170
160
150
140
130
120
110
100
90
80
70
60
50
40
30
20
10
0
-10
f1 (ppm)

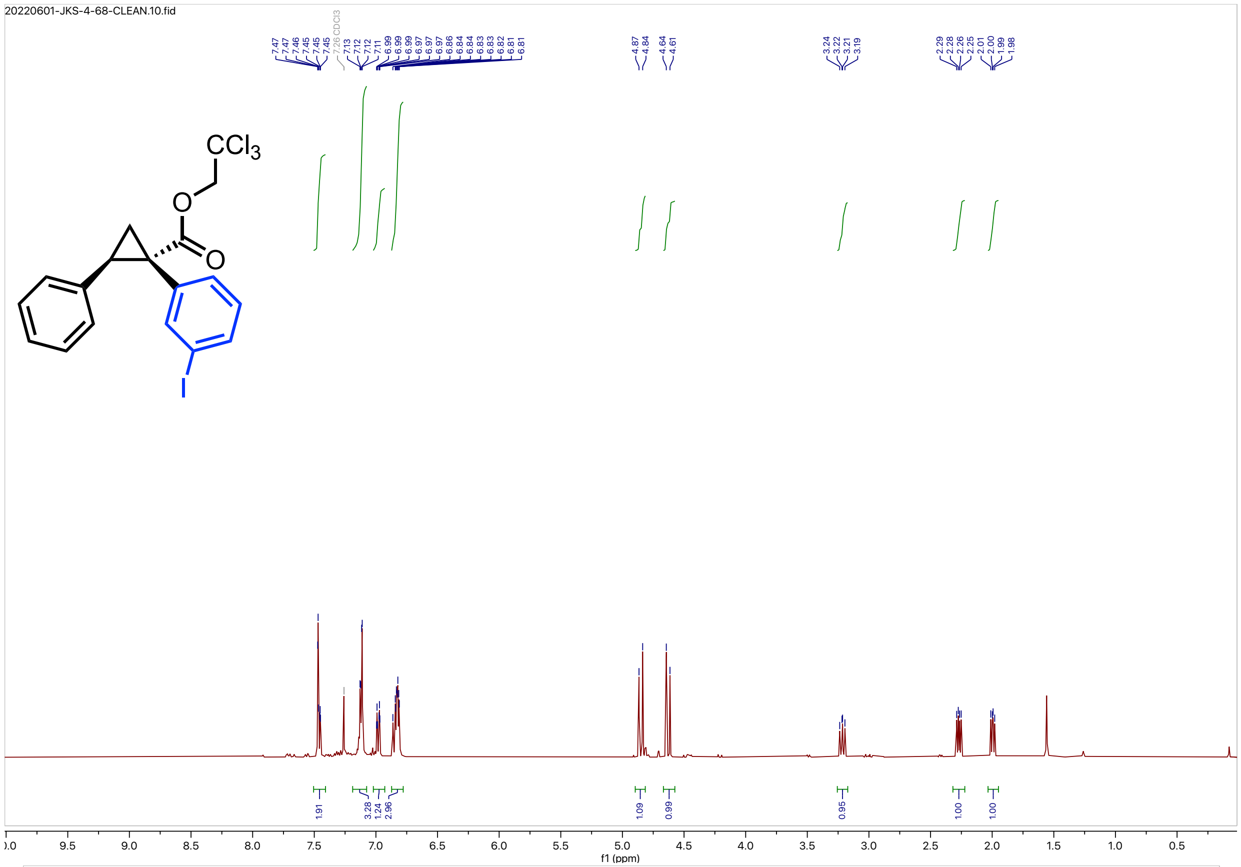
20220601-JKS-4-68-CLEAN.10.fid
CCl3
O
O
I
f1 (ppm)

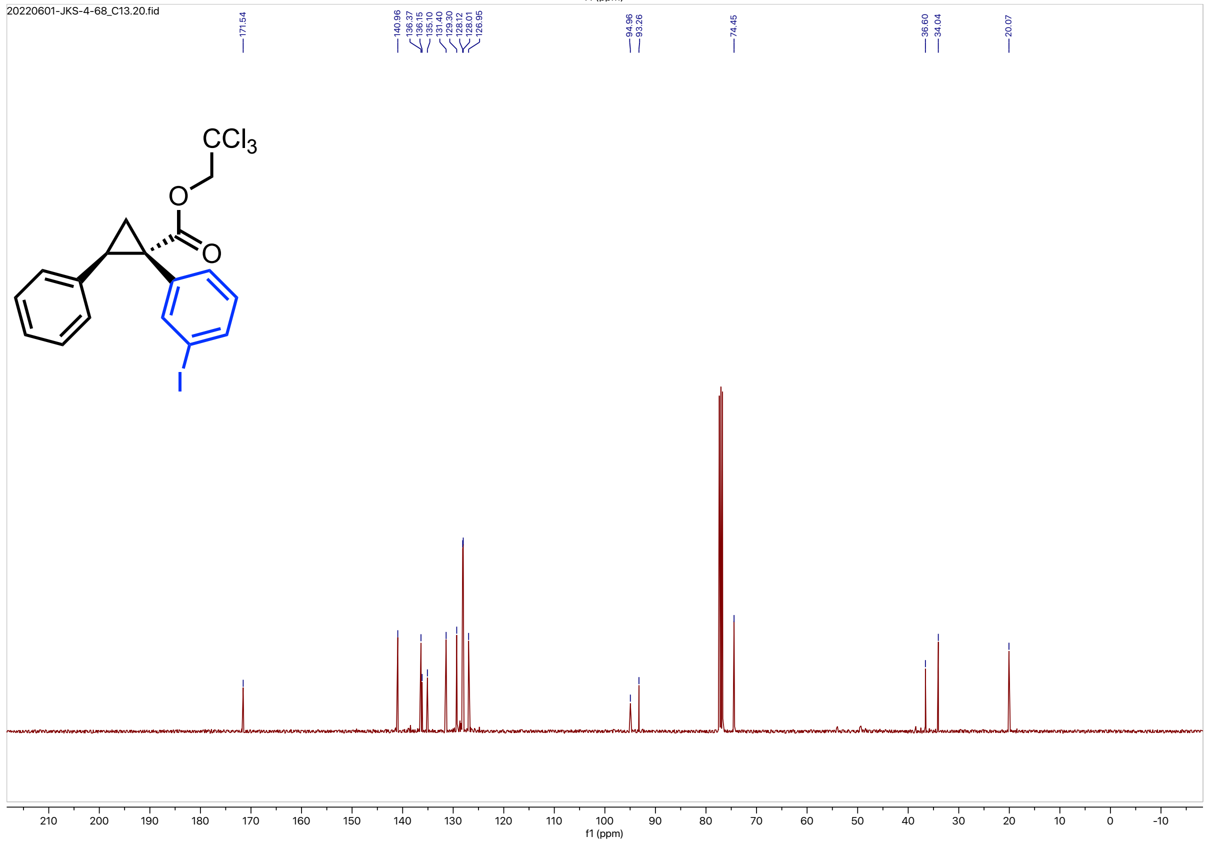
20220601-JKS-4-68_C13.20.fid
171.54
140.96
136.37
136.15
135.10
131.40
129.30
128.12
128.01
126.95
94.96
93.26
74.45
36.60
34.04
20.07
CCl3
O
O
I
f1 (ppm)

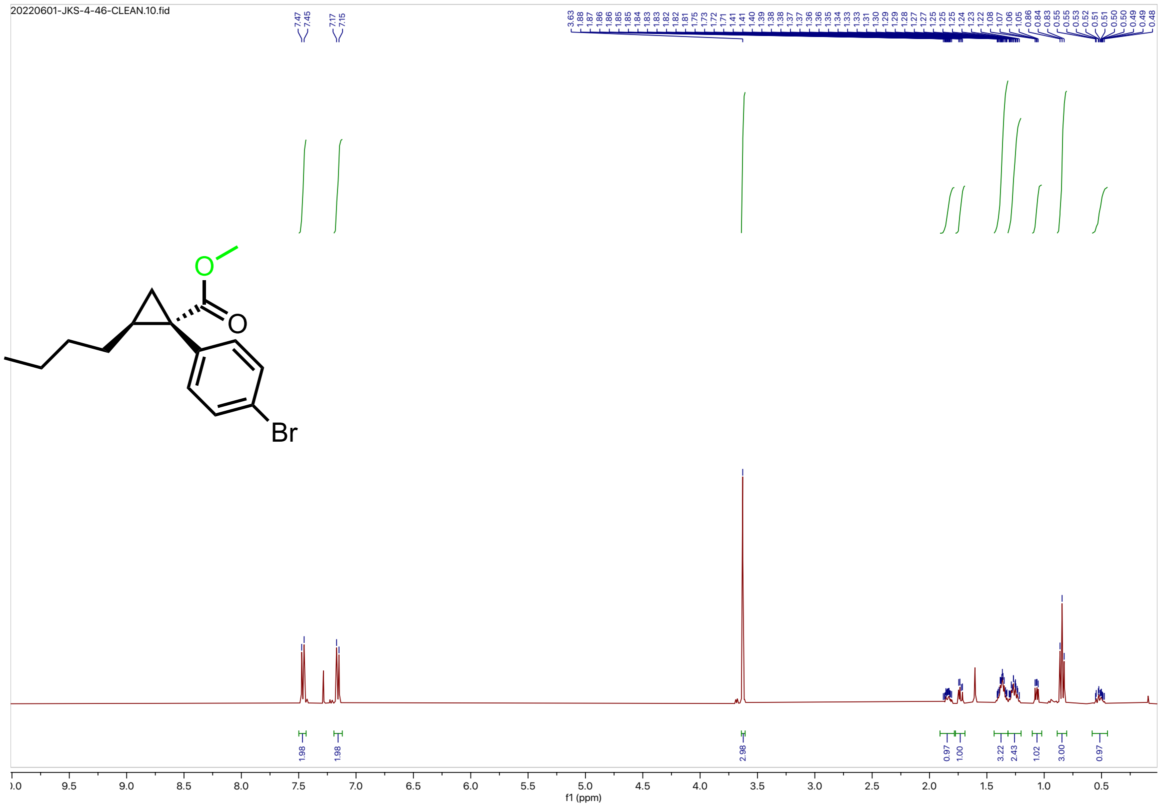
20220601-JKS-4-46-CLEAN.10.fid
Br
f1 (ppm)

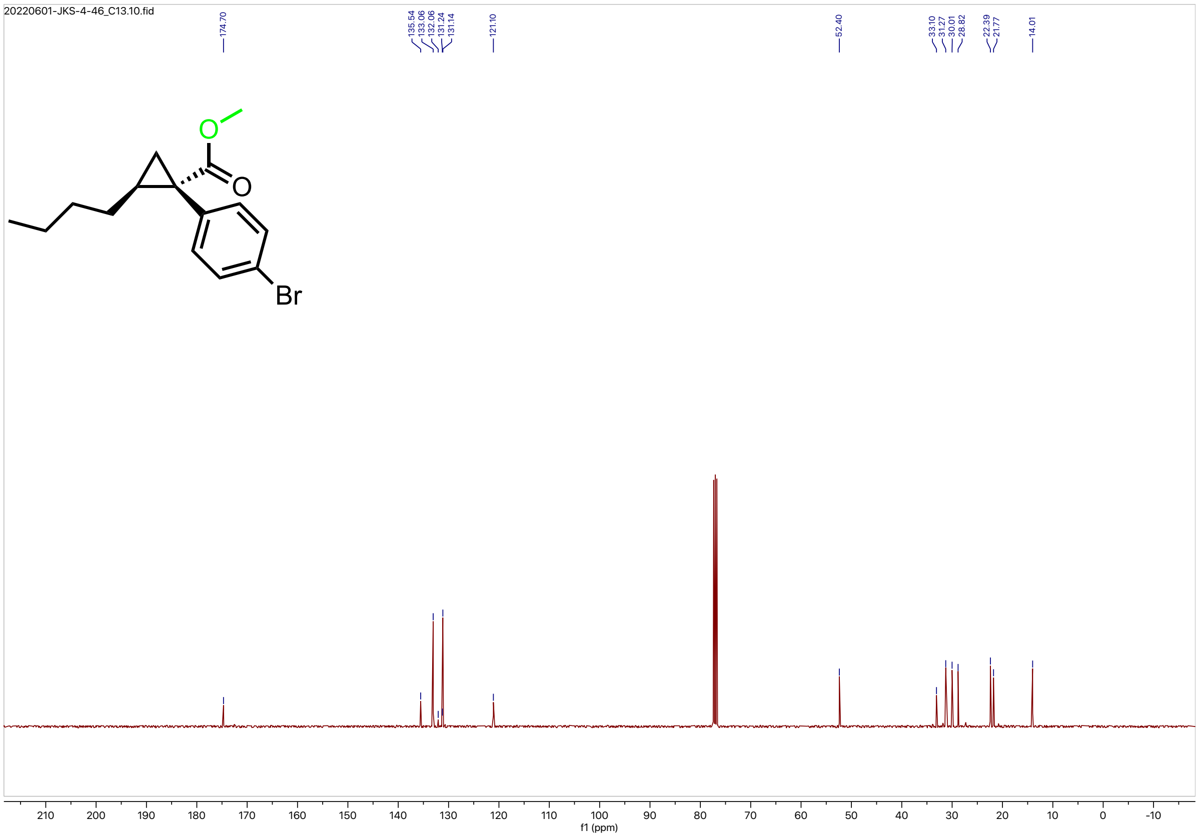
20220601-JKS-4-46_C13.10.fid
174.70
135.54
133.06
132.06
131.24
131.14
121.10
52.40
33.10
31.27
30.01
28.82
22.39
21.77
14.01
Br
f1 (ppm)

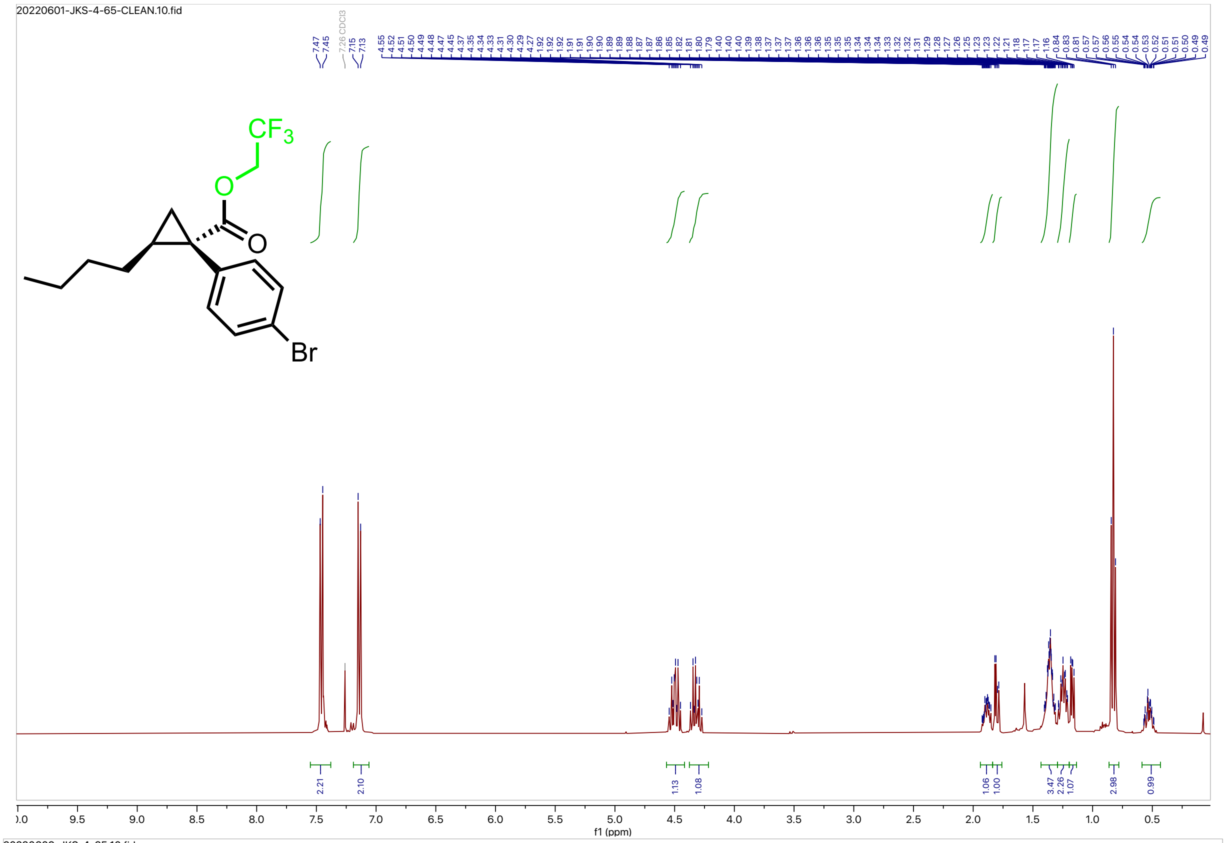

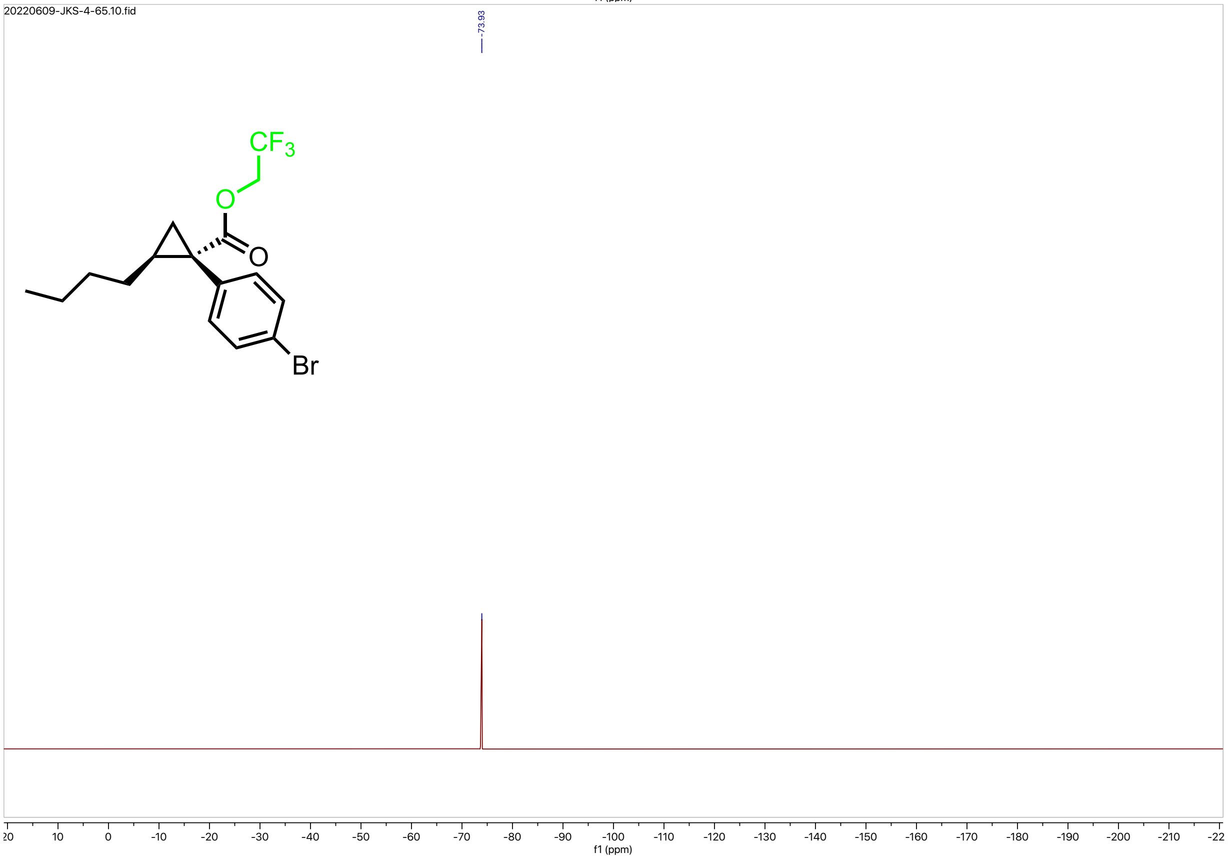

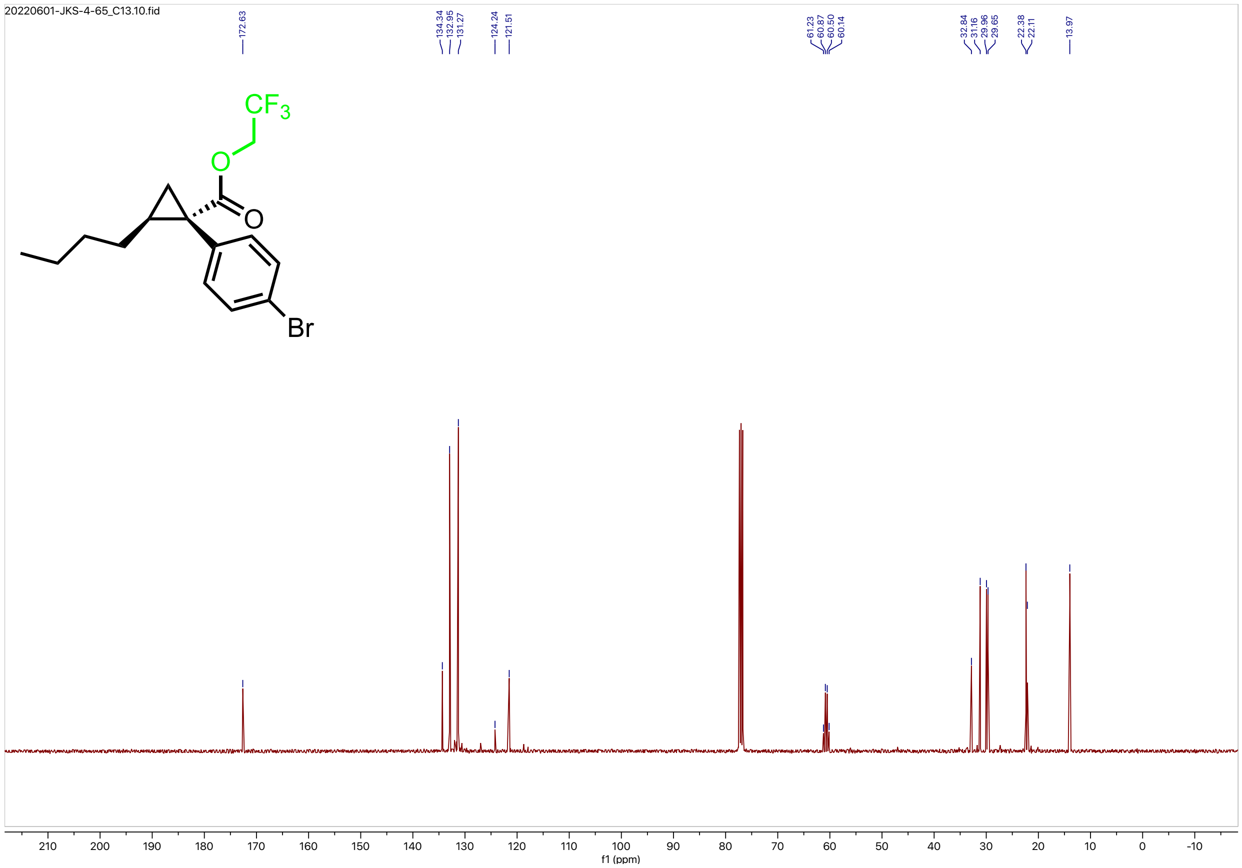
20220601-JKS-4-65_C13.10.fid
172.63
134.34
132.95
131.27
124.24
121.51
61.23
60.87
60.50
60.14
32.84
31.16
29.96
29.65
22.38
22.11
13.97
CF3
O
O
Br
f1 (ppm)

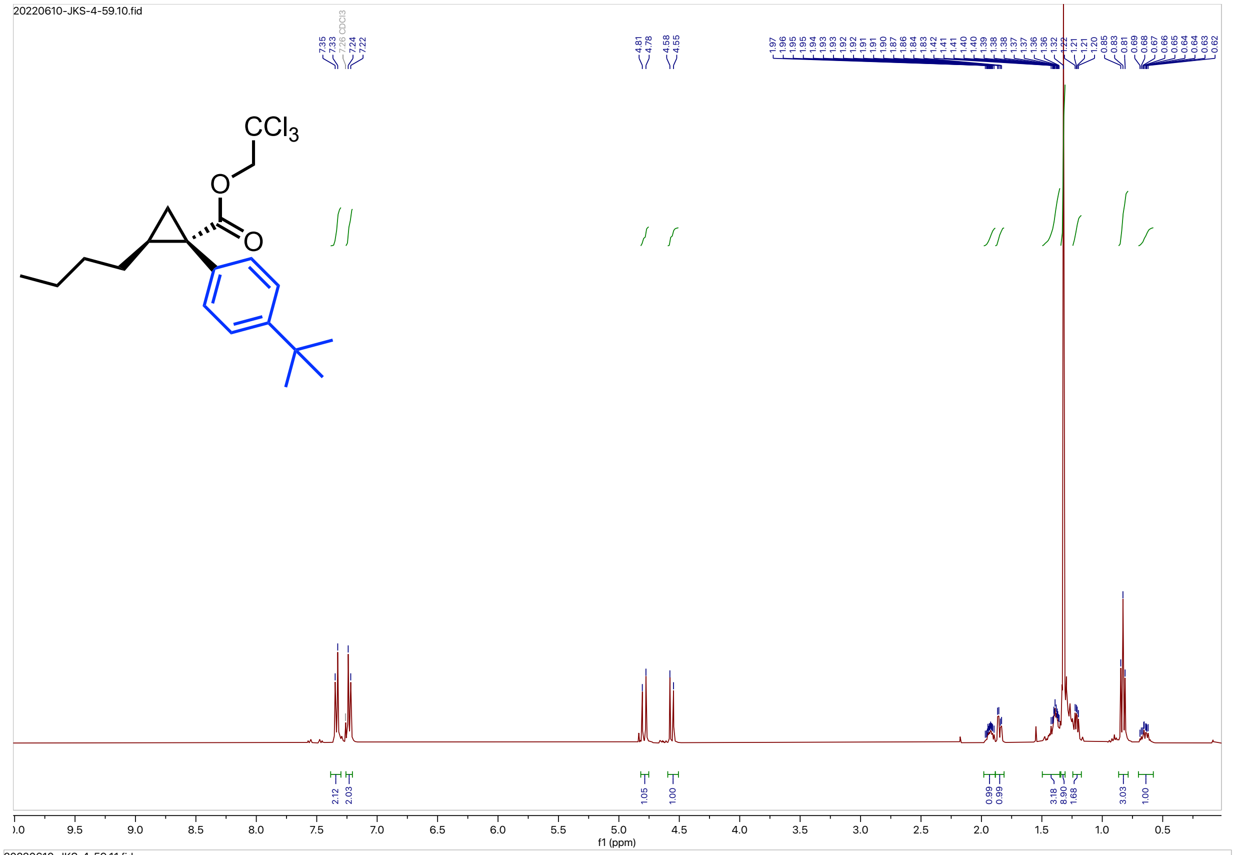
20220610-JKS-4-59.10.fid
CCl3
O
O
f1 (ppm)

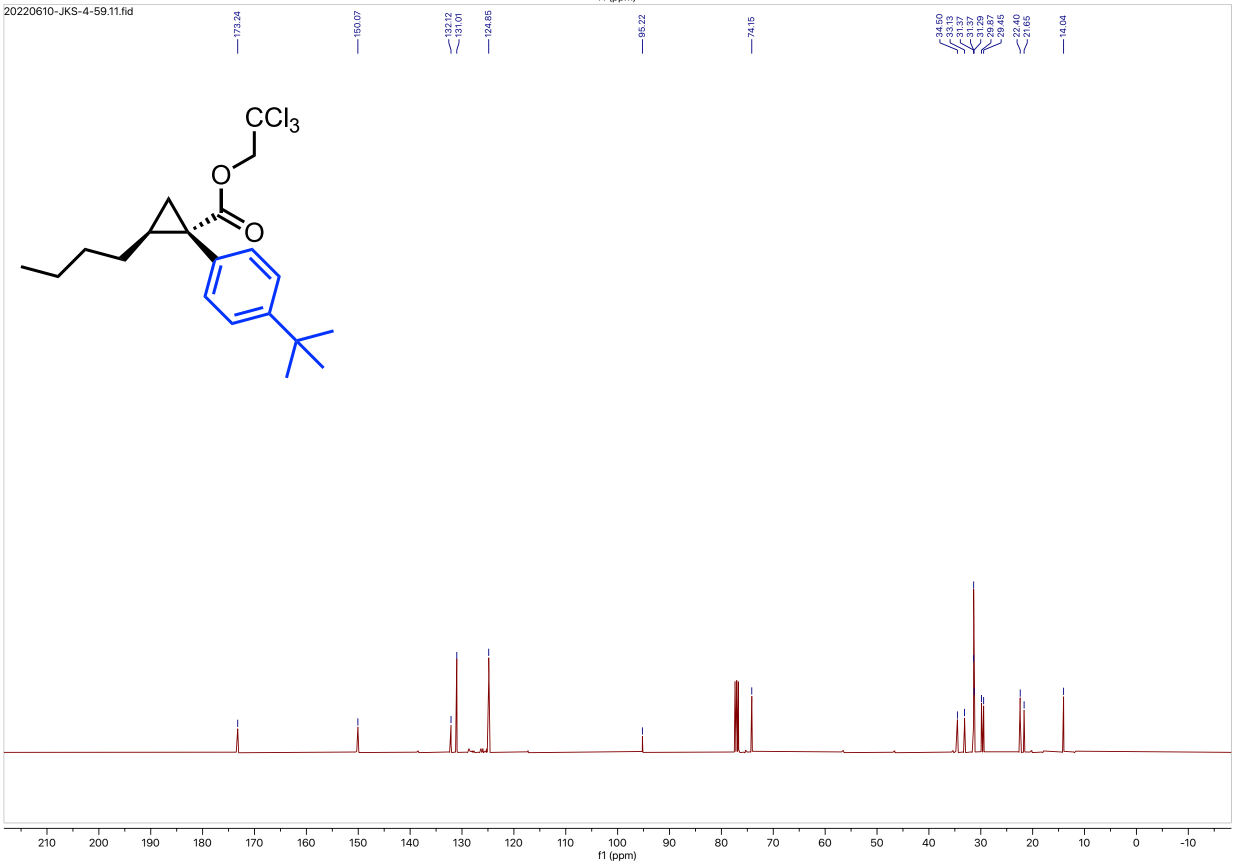
20220610-JKS-4-59.11.fid
173.24
150.07
132.12
131.01
124.85
95.22
74.15
34.50
33.13
31.37
31.29
29.87
29.45
22.40
21.65
14.04
CCl3
O
O
f1 (ppm)

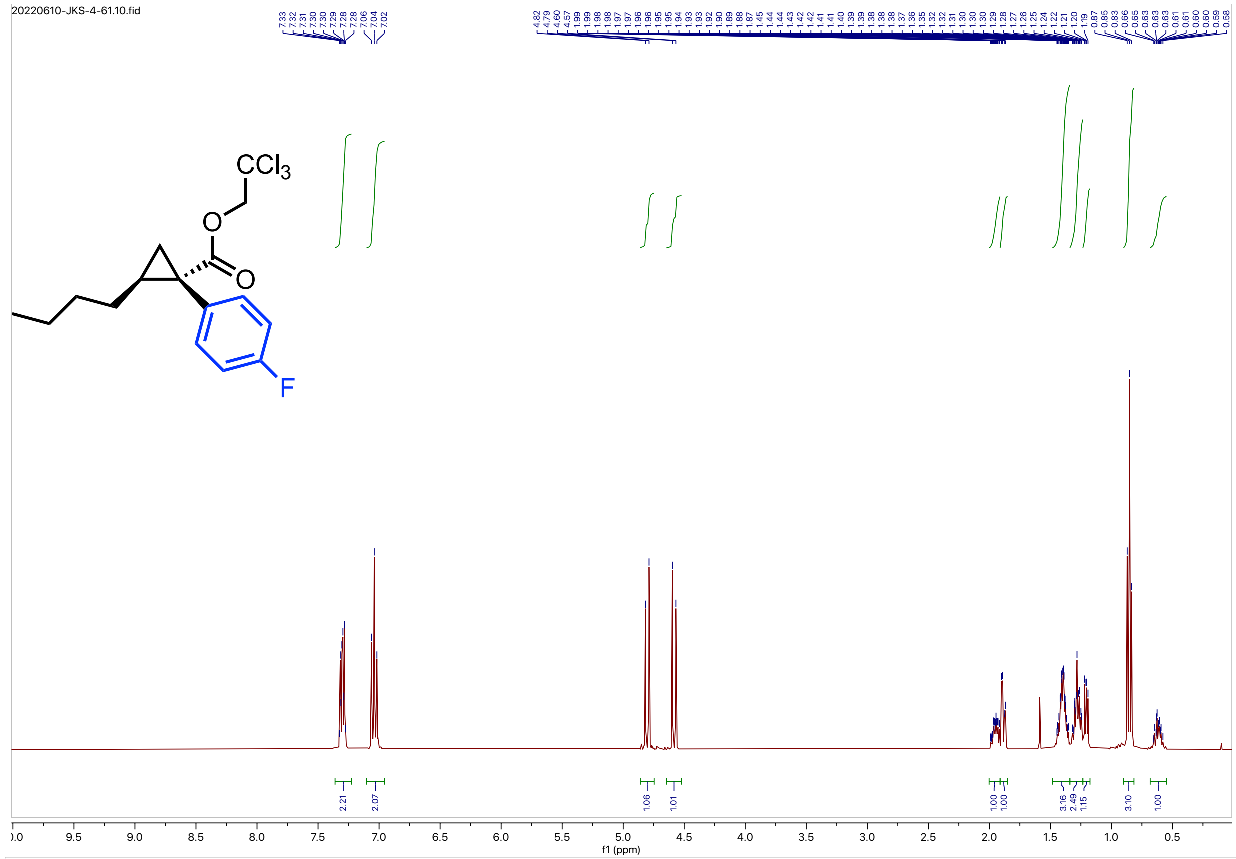
20220610-JKS-4-61.10.fid
CCl3
O
O
F
f1 (ppm)

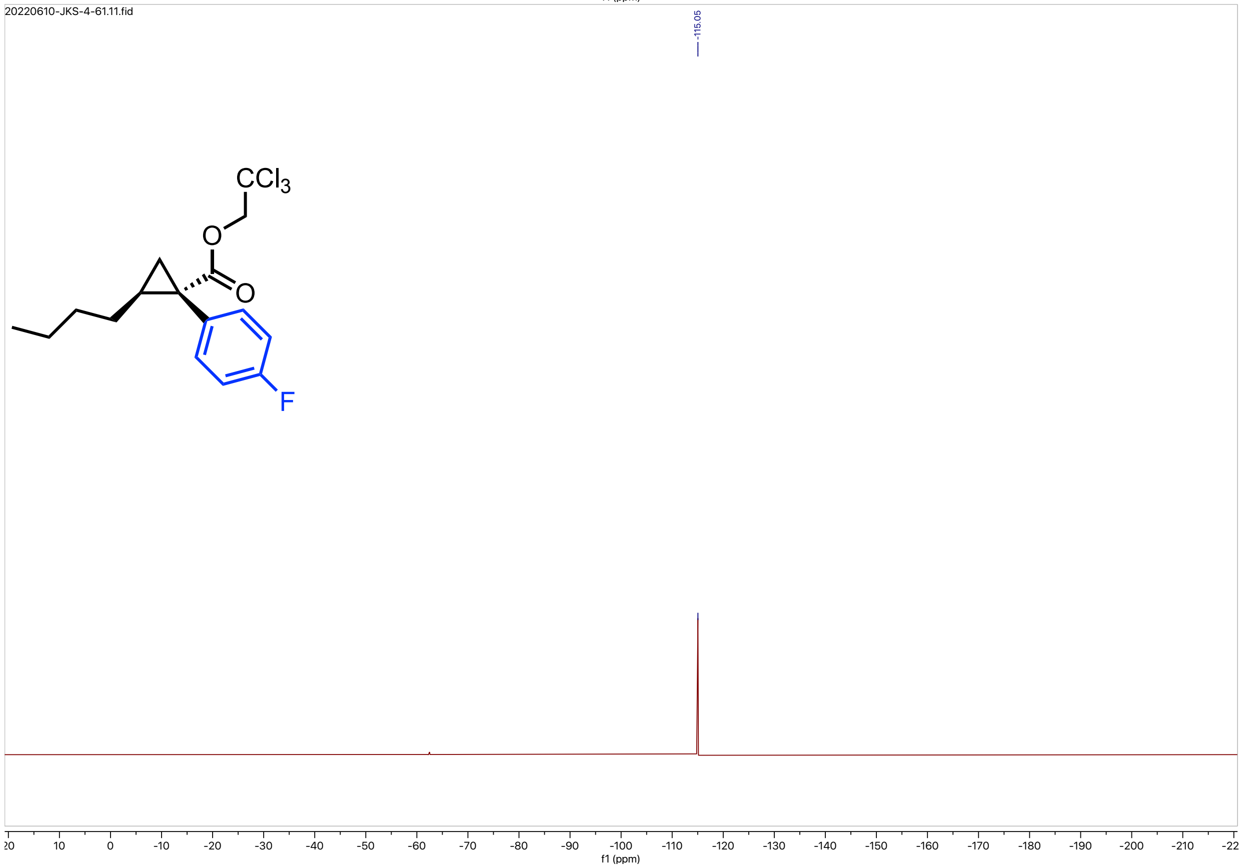
20220610-JKS-4-61.11.fid
-115.05
CCl3
O
O
F
f1 (ppm)

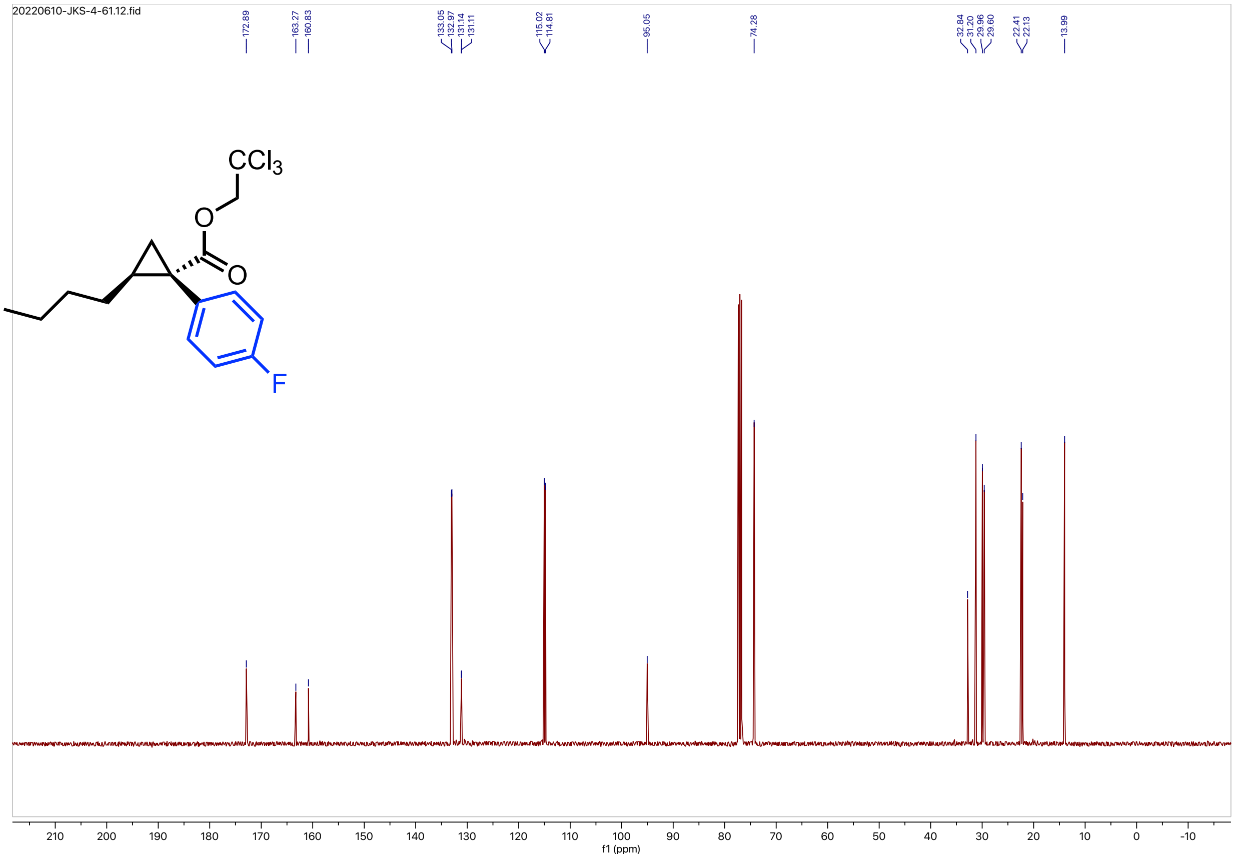

20220610-JKS-4-61.12.fid
172.89
163.27
160.83
133.05
132.97
131.14
131.11
115.02
114.81
95.05
74.28
32.84
31.20
29.96
29.60
22.41
22.13
13.99
CCl3
O
O
F
210
200
190
180
170
160
150
140
130
120
110
100
90
80
70
60
50
40
30
20
10
0
-10
f1 (ppm)

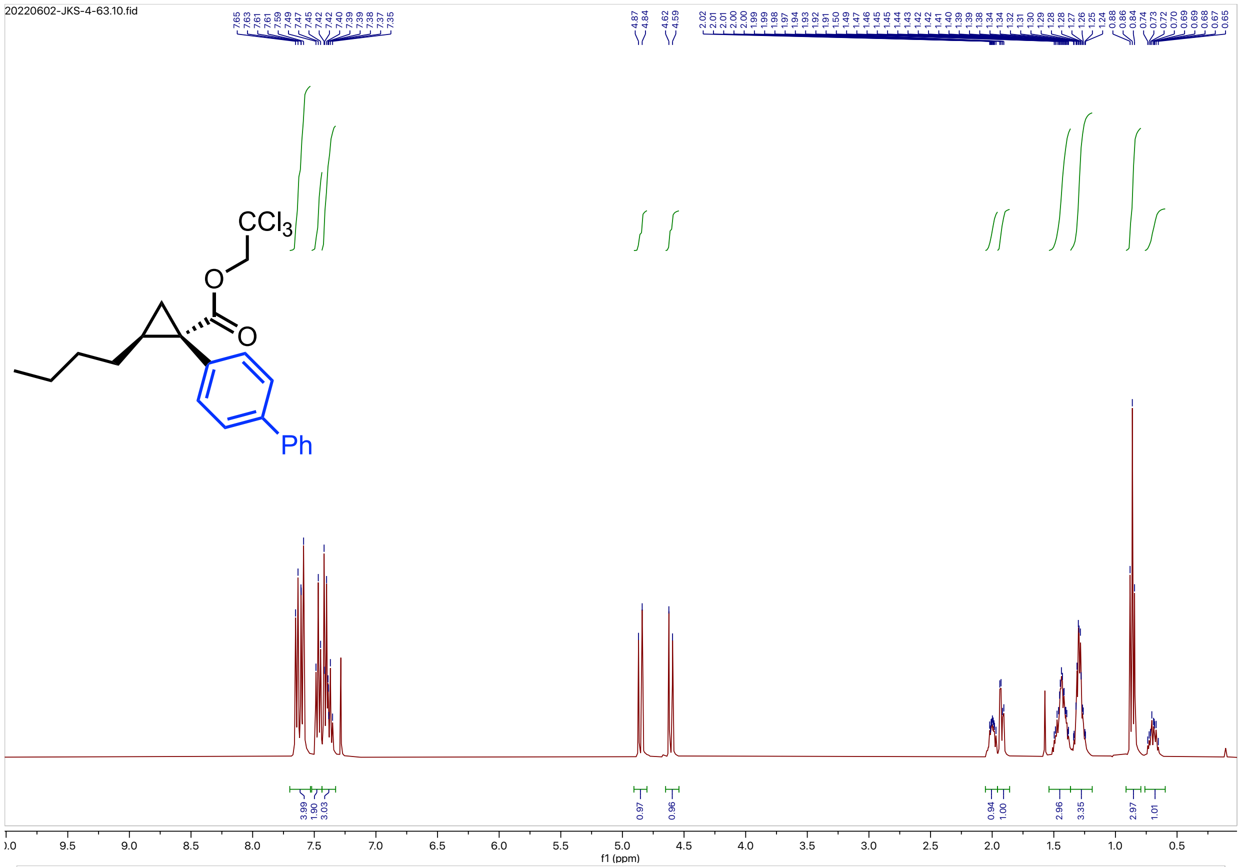
20220602-JKS-4-63.10.fid
CCl3
O
O
Ph
f1 (ppm)

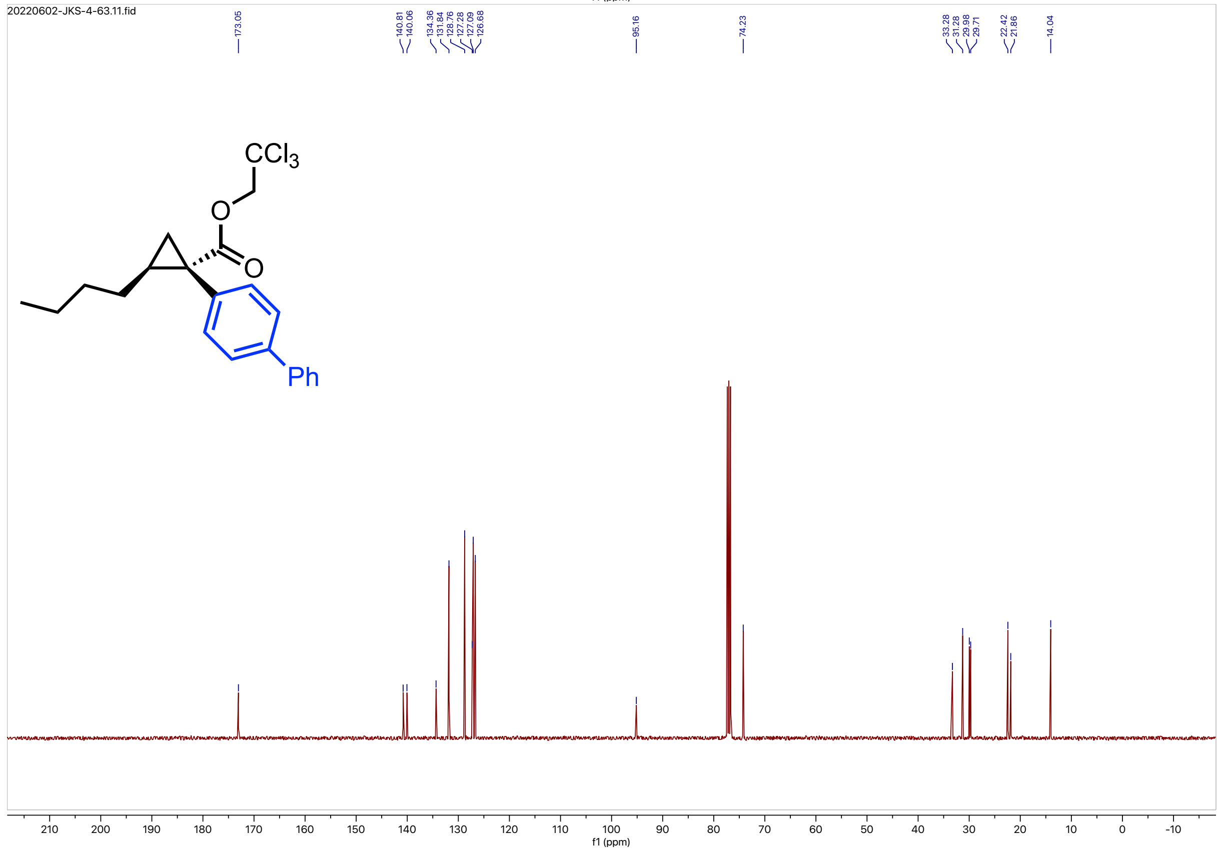
20220602-JKS-4-63.11.fid
173.05
140.81
140.06
134.36
131.84
128.76
127.28
127.09
126.68
95.16
74.23
33.28
31.28
29.98
29.71
22.42
21.86
14.04
CCl3
O
O
Ph
f1 (ppm)

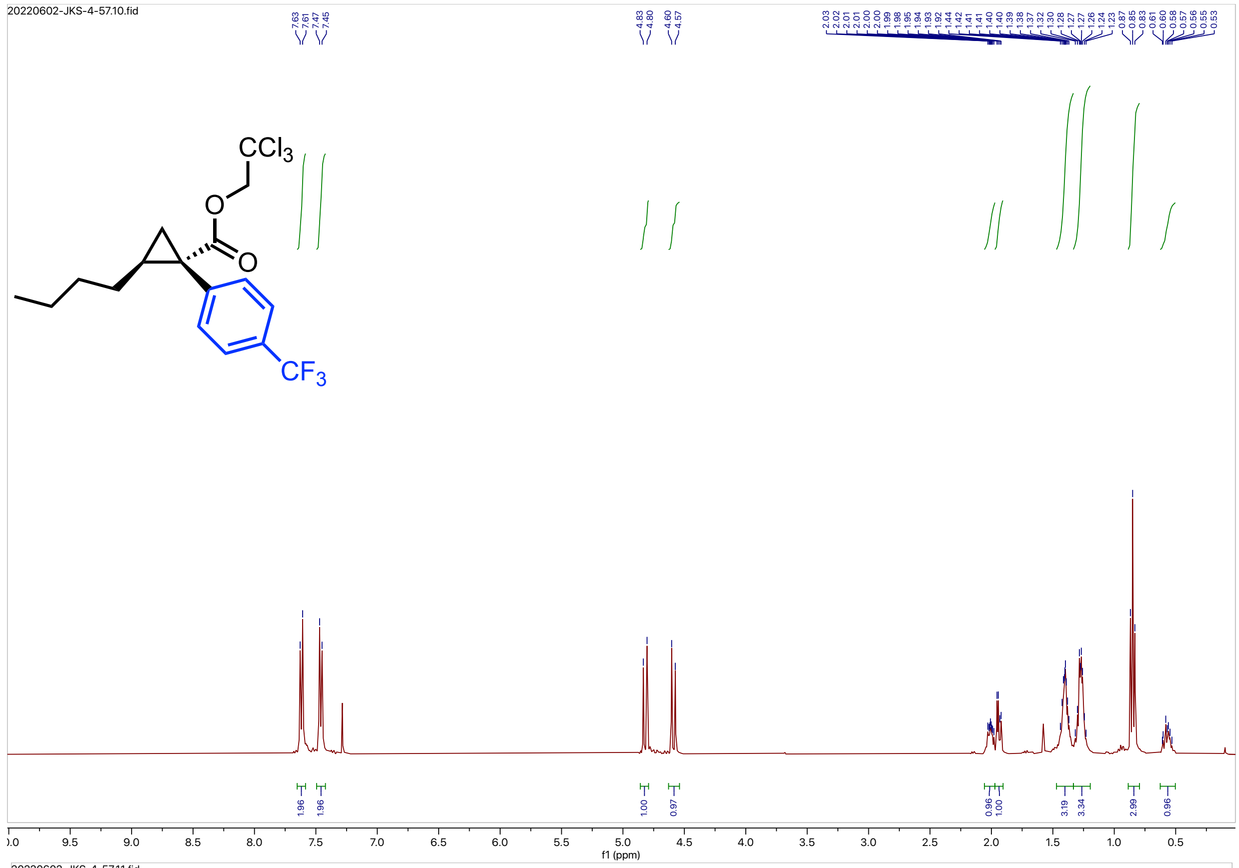

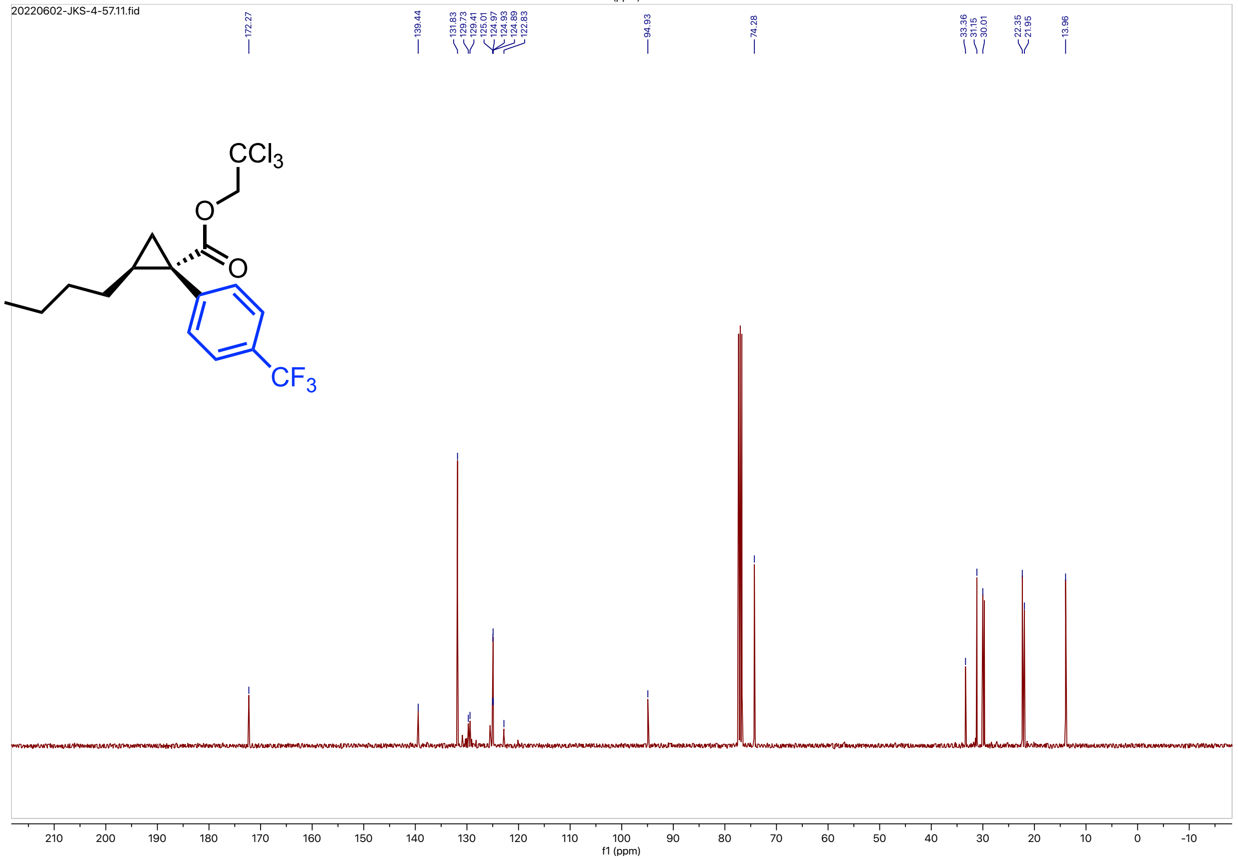

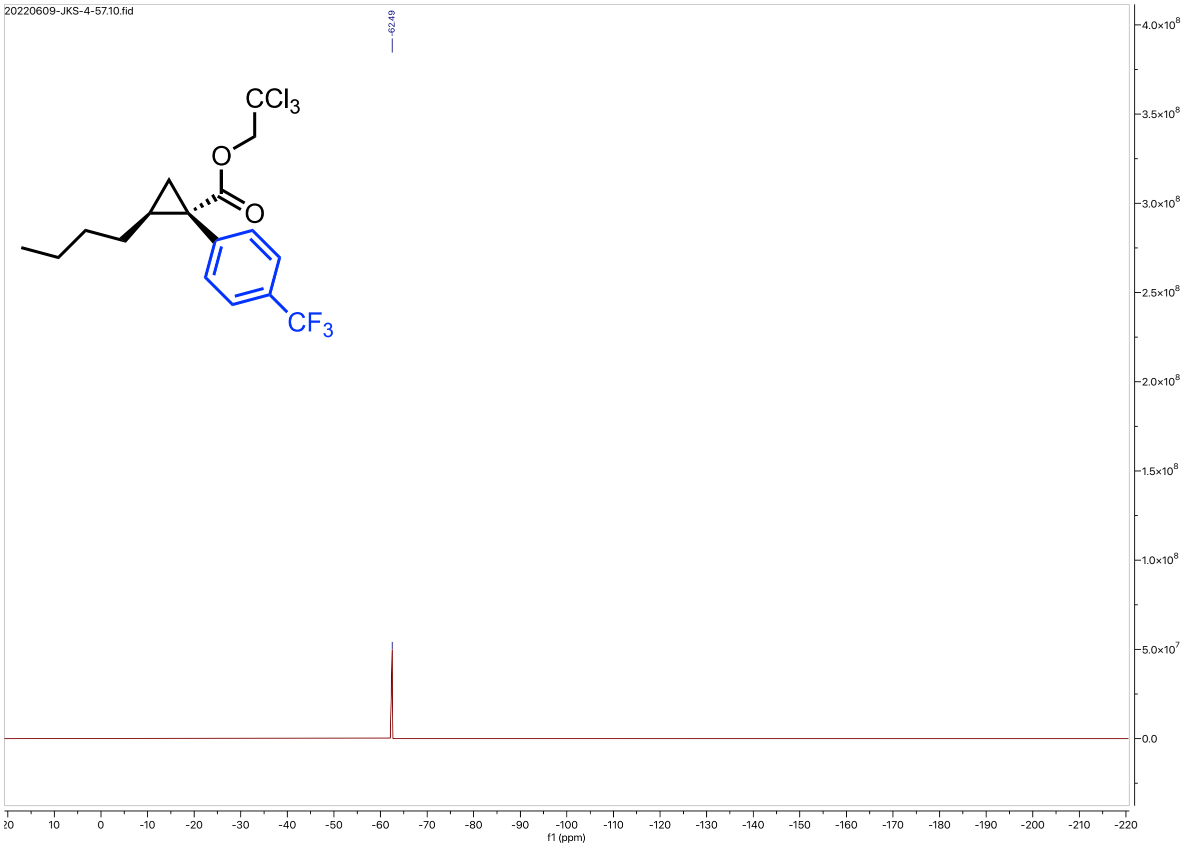
20220609-JKS-4-57.10.fid
-62.49
CCl3
O
O
CF3
f1 (ppm)

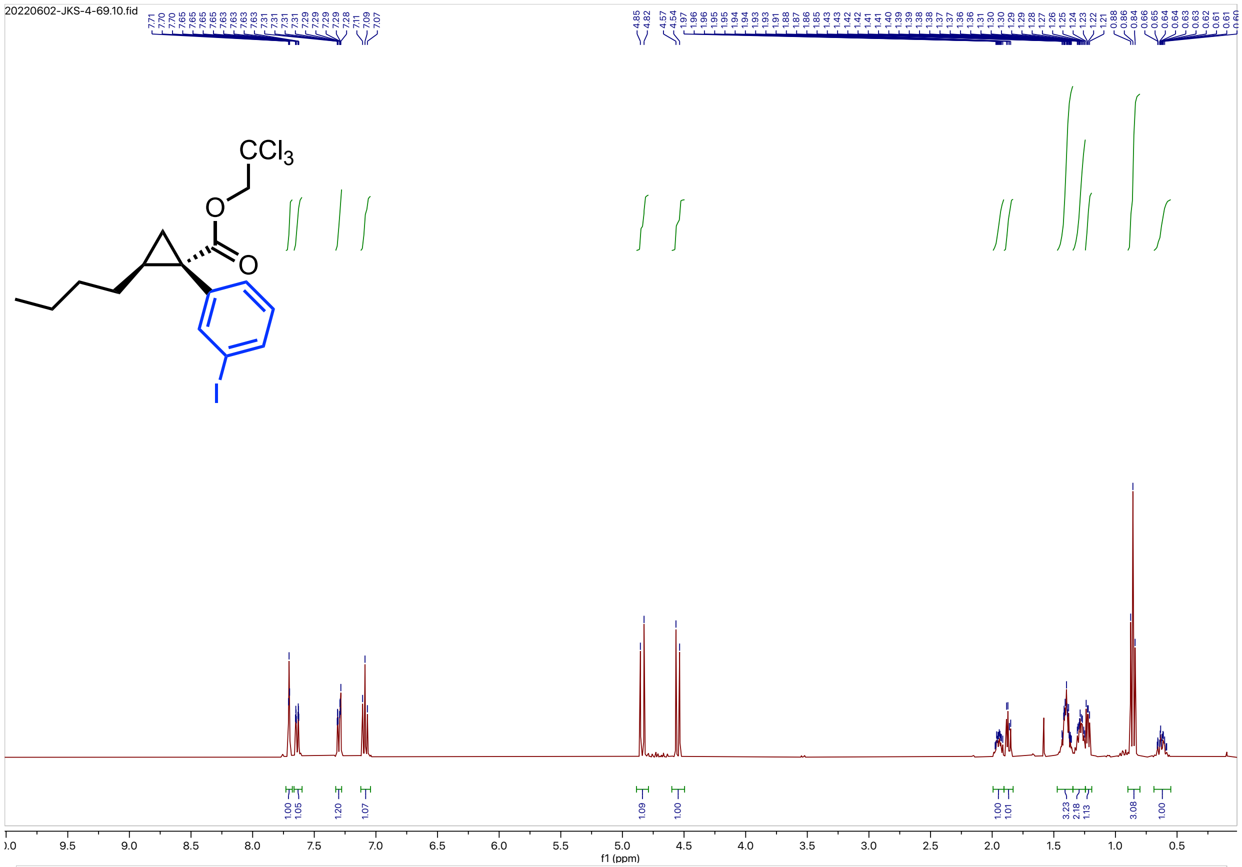
20220602-JKS-4-69.10.fid
CCl3
O
O
I
f1 (ppm)

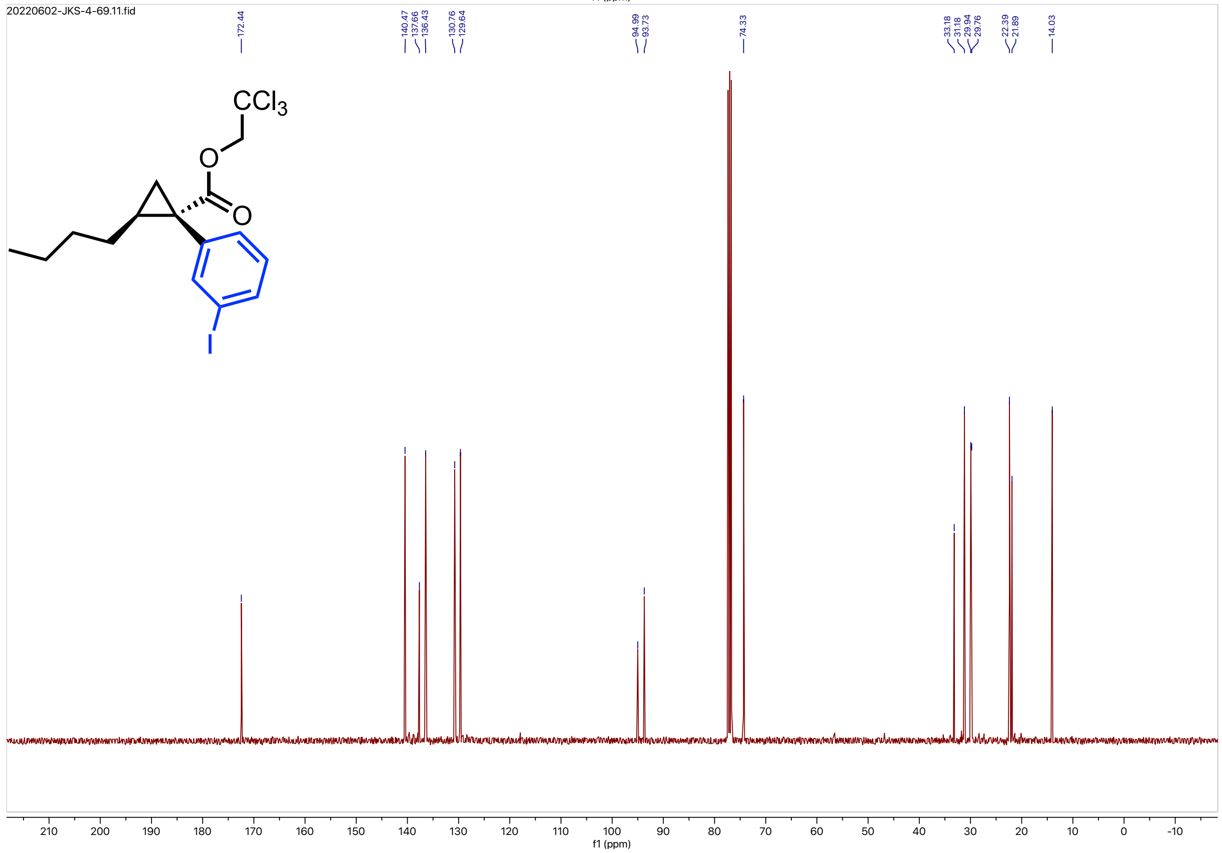
20220602-JKS-4-69.11.fid
172.44
140.47
137.66
136.43
130.76
129.64
94.99
93.73
74.33
33.18
31.18
29.94
29.76
22.39
21.89
14.03
CCl3
O
O
I
f1 (ppm)

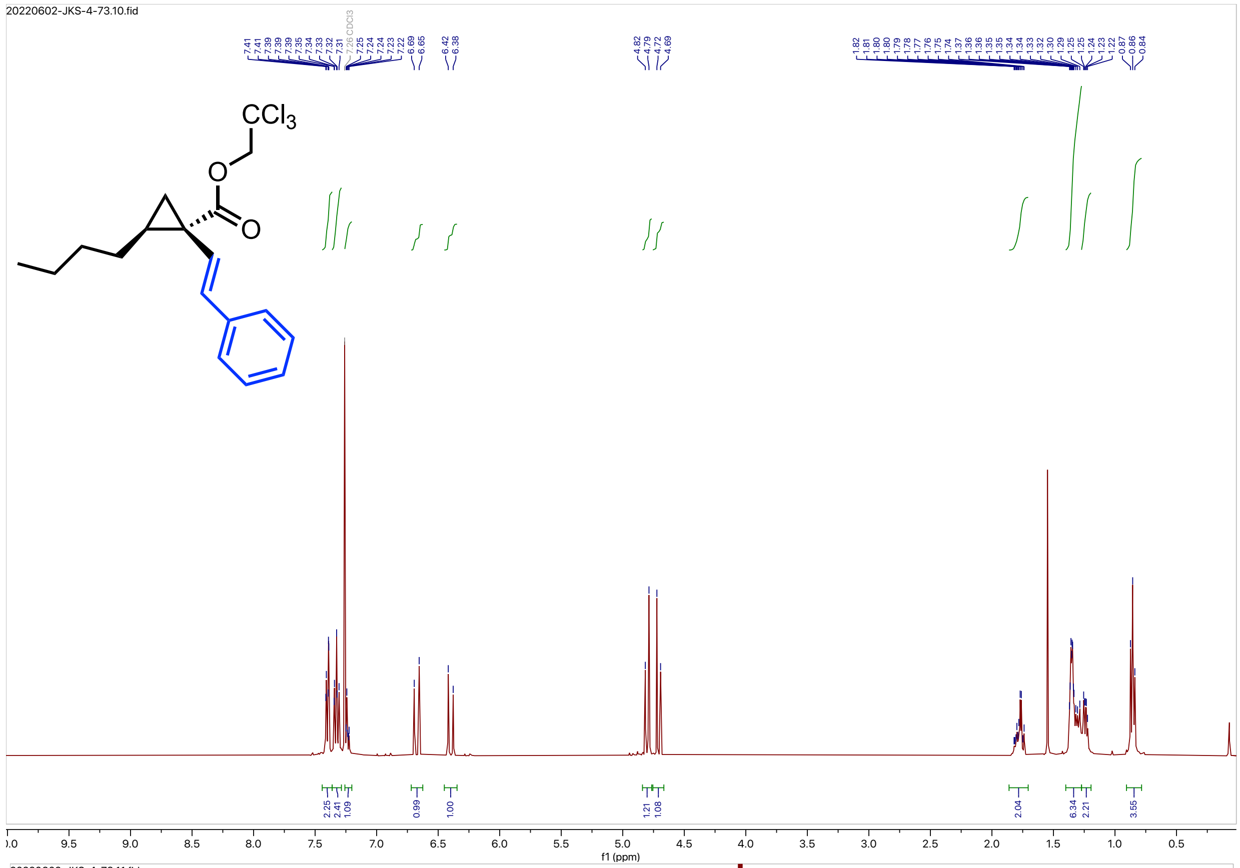
20220602-JKS-4-73.10.fid
CCl3
O
O
f1 (ppm)

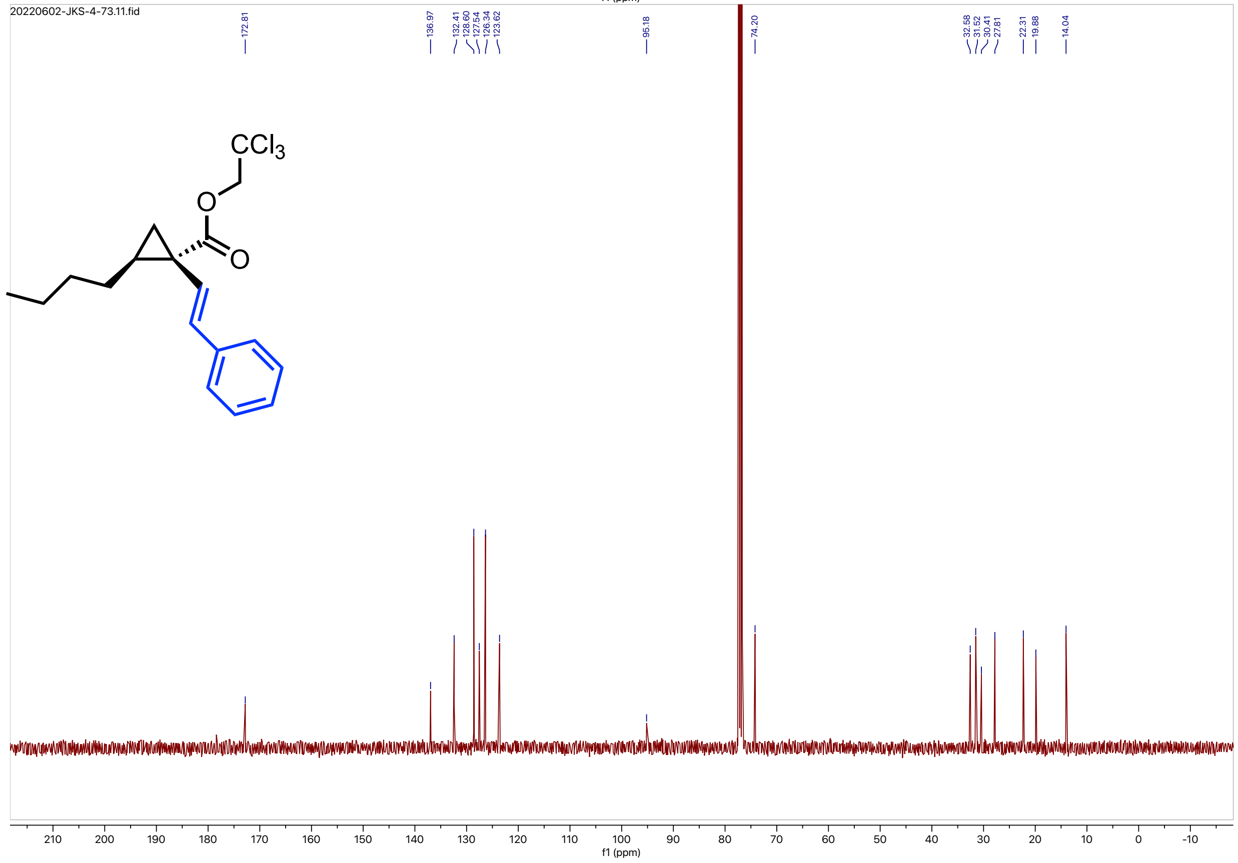
20220602-JKS-4-73.11.fid
172.81
136.97
132.41
128.60
127.54
126.34
123.62
95.18
74.20
32.58
31.52
30.41
27.81
22.31
19.88
14.04
CCl3
O
O
f1 (ppm)

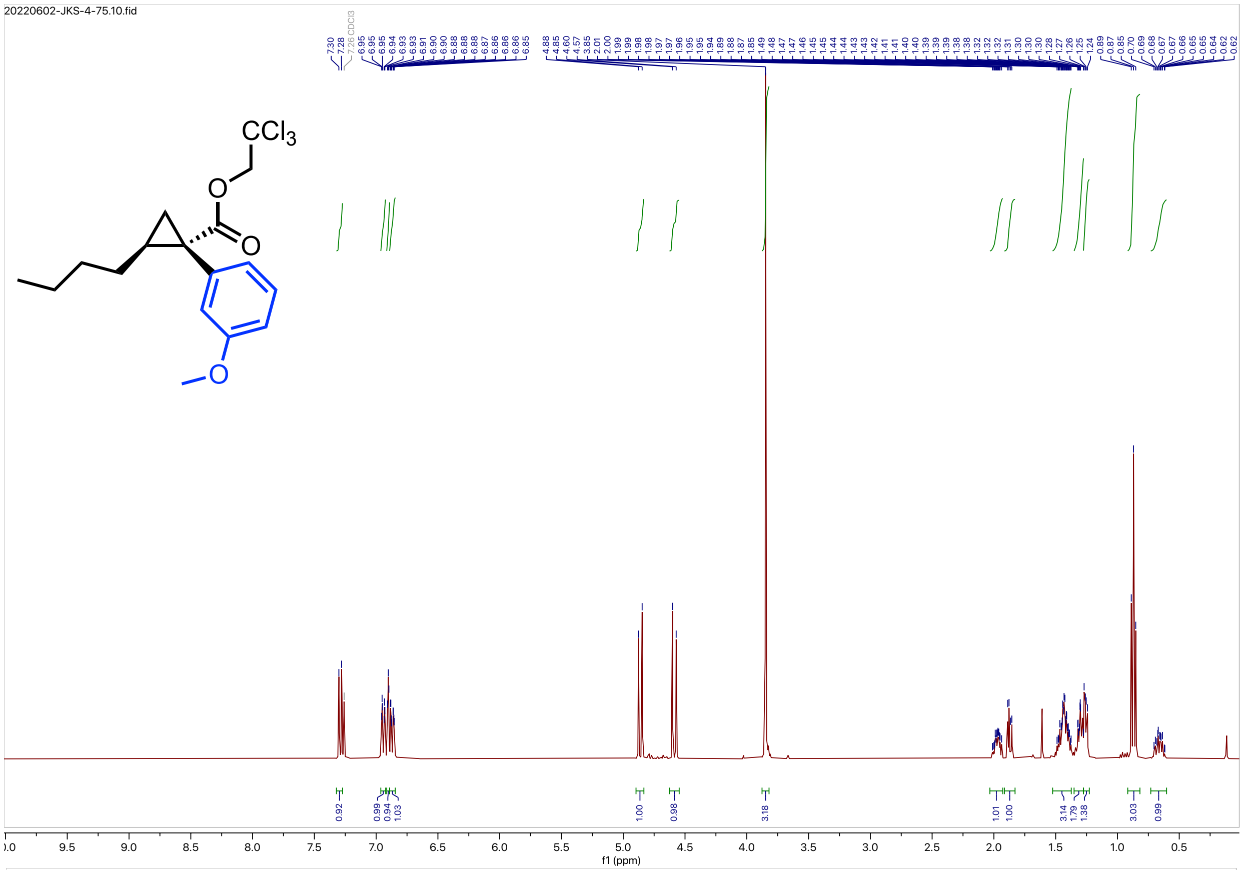
20220602-JKS-4-75.10.fid
f1 (ppm)

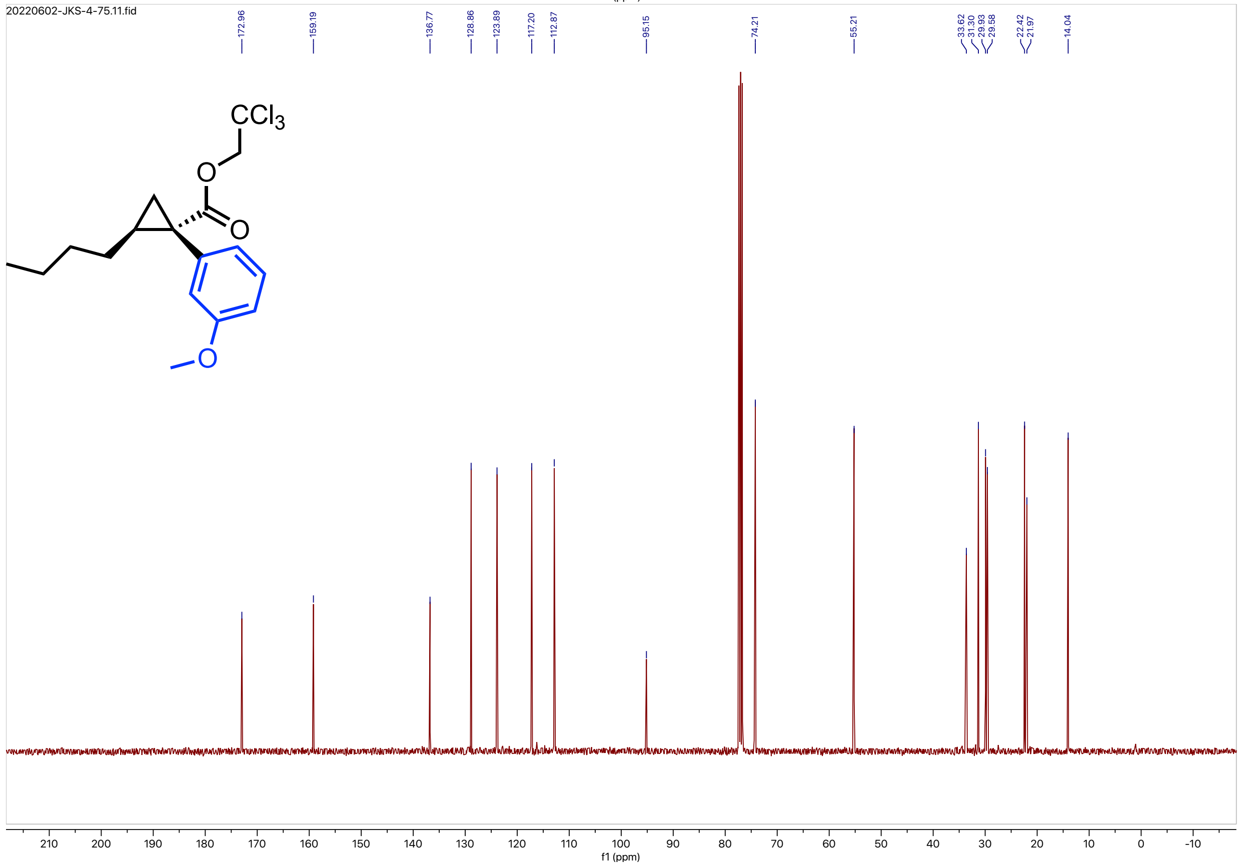
20220602-JKS-4-75.11.fid
172.96
159.19
136.77
128.86
123.89
117.20
112.87
95.15
74.21
55.21
33.62
31.30
29.93
29.58
22.42
21.97
14.04
f1 (ppm)

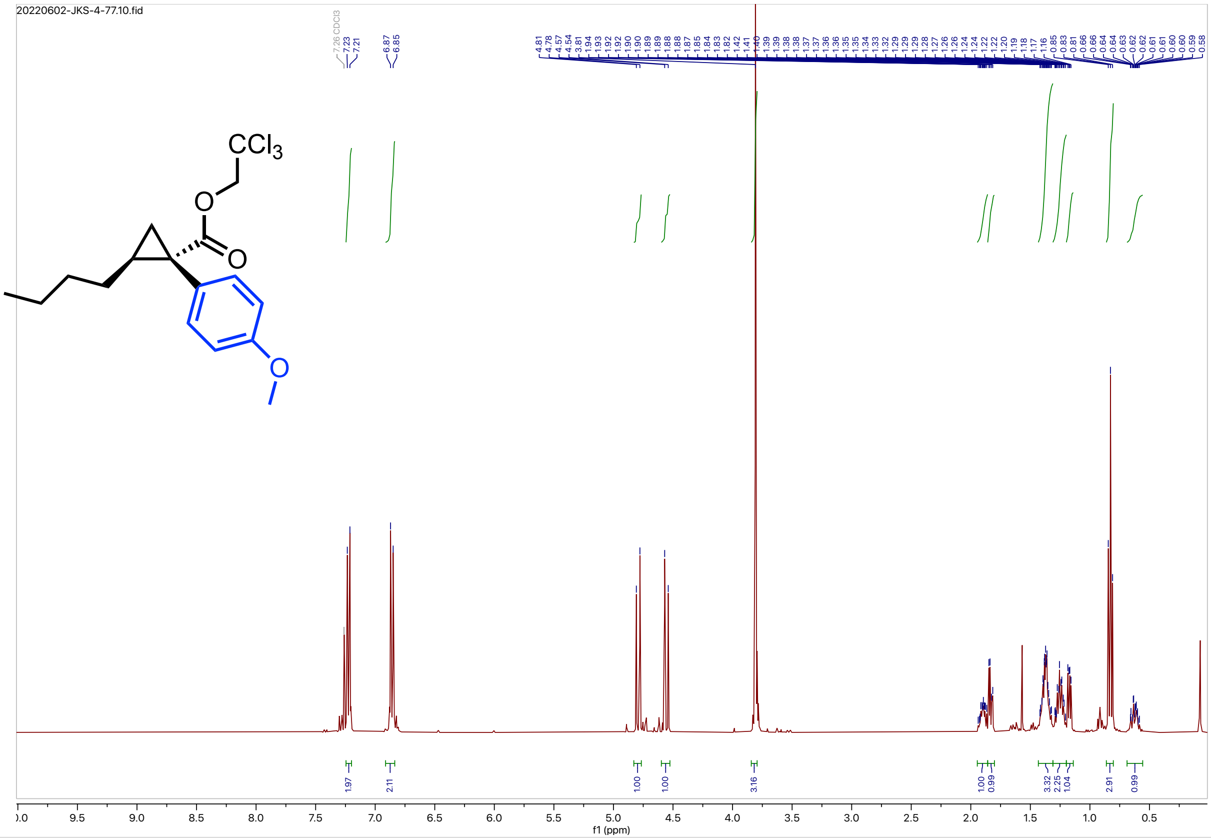
20220602-JKS-4-77.10.fid
CCl3
O
O
f1 (ppm)

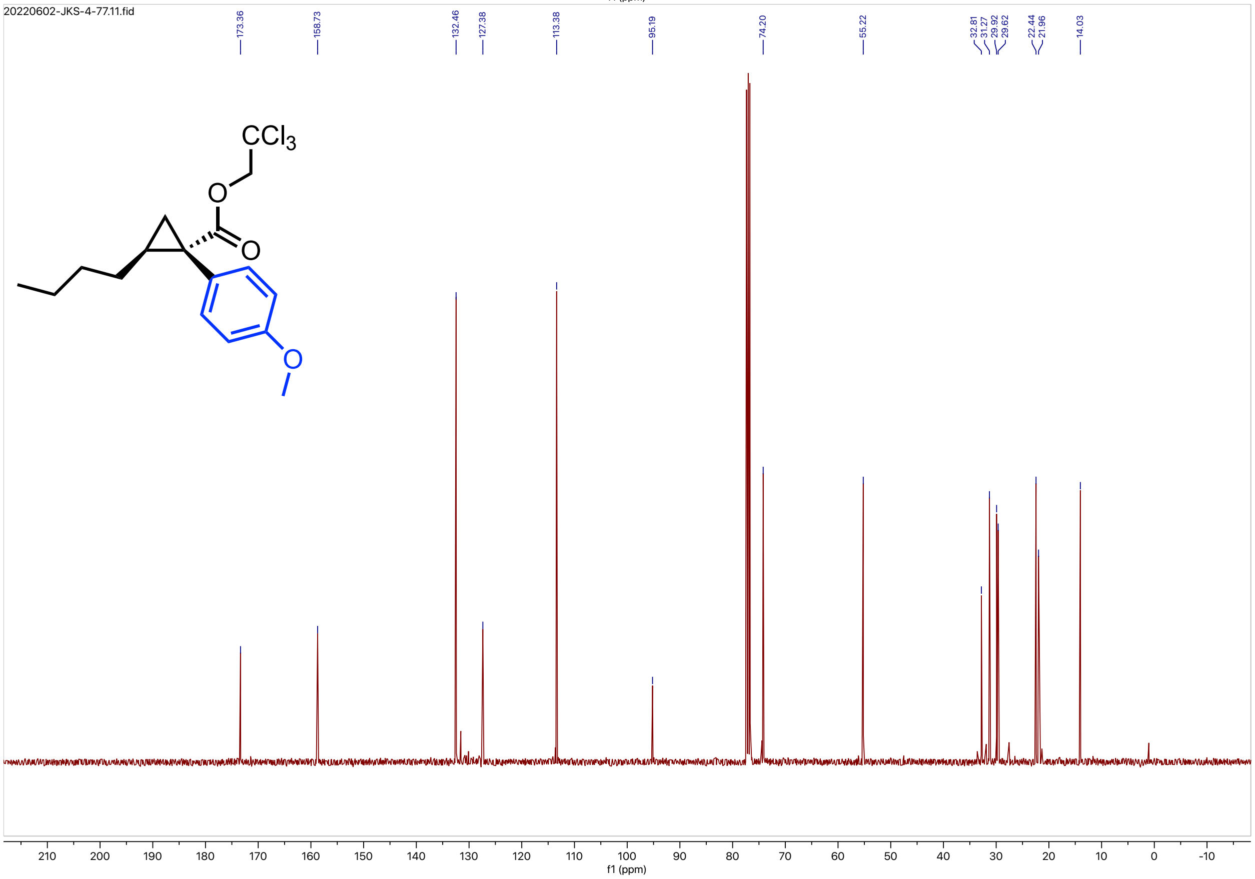
20220602-JKS-4-77.11.fid
173.36
158.73
132.46
127.38
113.38
95.19
74.20
55.22
32.81
31.27
29.92
29.62
22.44
21.96
14.03
CCl3
O
O
f1 (ppm)

References

1. Green, S. P.; Wheelhouse, K. M.; Payne, A. D.; Hallett, J. P.; Miller, P. W.; Bull, J. A., Thermal Stability and Explosive Hazard Assessment of Diazo Compounds and Diazo Transfer Reagents. *Organic Process Research & Development* **2020,** *24* (1), 67-84.

2. Guptill, D. M.; Davies, H. M. L., 2,2,2-Trichloroethyl Aryldiazoacetates as Robust Reagents for the Enantioselective C–H Functionalization of Methyl Ethers. *Journal of the American Chemical Society* **2014,** *136* (51), 17718-17721.

3. Wei, B.; Sharland, J. C.; Lin, P.; Wilkerson-Hill, S. M.; Fullilove, F. A.; McKinnon, S.; Blackmond, D. G.; Davies, H. M. L., In Situ Kinetic Studies of Rh(II)-Catalyzed Asymmetric Cyclopropanation with Low Catalyst Loadings. *ACS Catalysis* **2020,** *10* (2), 1161-1170.

4. Davies, H. M. L.; Venkataramani, C., Dirhodium Tetraprolinate-Catalyzed Asymmetric Cyclopropanations with High Turnover Numbers. *Organic Letters* **2003,** *5* (9), 1403-1406.

5. Garlets, Z. J.; Hicks, E. F.; Fu, J.; Voight, E. A.; Davies, H. M. L., Regio- and Stereoselective Rhodium(II)-Catalyzed C–H Functionalization of Organosilanes by Donor/Acceptor Carbenes Derived from Aryldiazoacetates. *Organic Letters* **2019,** *21* (12), 4910-4914.

6. Fu, J.; Ren, Z.; Bacsa, J.; Musaev, D. G.; Davies, H. M. L., Desymmetrization of cyclohexanes by site- and stereoselective C–H functionalization. *Nature* **2018,** *564* (7736), 395-399.

7. Tsutsui, H.; Abe, T.; Nakamura, S.; Anada, M.; Hashimoto, S., Practical Synthesis of Dirhodium(II) Tetrakis[<i>N</i>-phthaloyl-(<i>S</i>)-<i>tert</i>-leucinate]. *Chemical and Pharmaceutical Bulletin* **2005,** *53* (10), 1366-1368.

8. Reddy, R. P.; Lee, G. H.; Davies, H. M. L., Dirhodium Tetracarboxylate Derived from Adamantylglycine as a Chiral Catalyst for Carbenoid Reactions. *Organic Letters* **2006,** *8* (16), 3437-3440.

9. Reddy, R. P.; Davies, H. M. L., Dirhodium Tetracarboxylates Derived from Adamantylglycine as Chiral Catalysts for Enantioselective C–H Aminations. *Organic Letters* **2006,** *8* (22), 5013-5016.

10. Hoshino, Y.; Yamamoto, H., Novel α-Amino Acid-Based Hydroxamic Acid Ligands for Vanadium-Catalyzed Asymmetric Epoxidation of Allylic Alcohols. *Journal of the American Chemical Society* **2000,** *122* (42), 10452-10453.

11. Miyazawa, T.; Suzuki, T.; Kumagai, Y.; Takizawa, K.; Kikuchi, T.; Kato, S.; Onoda, A.; Hayashi, T.; Kamei, Y.; Kamiyama, F.; Anada, M.; Kojima, M.; Yoshino, T.; Matsunaga, S., Chiral paddle-wheel diruthenium complexes for asymmetric catalysis. *Nature Catalysis* **2020,** *3* (10), 851-858.

12. Wei, B.; Hatridge, T. A.; Jones, C. W.; Davies, H. M. L., Copper(II) Acetate-Induced Oxidation of Hydrazones to Diazo Compounds under Flow Conditions Followed by Dirhodium-Catalyzed Enantioselective Cyclopropanation Reactions. *Organic Letters* **2021,** *23* (14), 5363-5367.

13. Sharland, J. C.; Dunstan, D.; Majumdar, D.; Gao, J.; Tan, K.; Malik, H. A.; Davies, H. M. L., Hexafluoroisopropanol for the Selective Deactivation of Poisonous Nucleophiles Enabling Catalytic Asymmetric Cyclopropanation of Complex Molecules. *ACS Catalysis* **2022,** *12* (20), 12530-12542.

14. Singha, S.; Buchsteiner, M.; Bistoni, G.; Goddard, R.; Fürstner, A., A New Ligand Design Based on London Dispersion Empowers Chiral Bismuth–Rhodium Paddlewheel Catalysts. *Journal of the American Chemical Society* **2021,** *143* (15), 5666-5673.

Computational Details

All calculations were carried out by utilizing the Gaussian-16 quantum chemistry software package.[1] Geometries, frequencies, and thermodynamic parameters of these species were calculated at the B3LYP density functional,[2-4] in conjunction with Grimme's empirical dispersion-correction (D3)[5], and Becke and Becke-Johnson (BJ) damping-corrections.[6-8] In these calculations we utilized the 6-31G(d,p) basis sets for all atoms, except of transition metals (Cu, Co, Rh and Ru) and bromine. For later atoms we use LANL2DZ basis sets and associated effective core potentials (ECP).[9] Bulk solvent effects were incorporated into all calculations (including geometry optimizations and frequency calculations) using the self-consistent reaction field polarizable continuum model (IEF-PCM).[10,11] We chose dichloromethane as solvent. Below, we labeled this approximation as a {[B3LYP-D3(BJ)]+PCM}/[6-31G(d,p) + Lanl2dz] approximation. The reported thermodynamic data were computed at a temperature of 298.15K and at 1atm of pressure. Unless otherwise stated, energies are given as ΔH/ΔG in kcal/mol.

To validate the {[B3LYP-D3(BJ)]+PCM}/[6-31G(d,p) + Lanl2dz] calculated energetics of the reported structures we have also re-calculated their energetics at the [wB97xd[12] + PCM]/{[6-311+G(d,p)] + SDD[13] (for Cu, Co, Rh, Ru, and Br)} level of theory by utilizing their {[B3LYP-D3(BJ)]+PCM}/[6-31G(d,p) + Lanl2dz] optimized geometries. The calculated energetics of these structures at the {[B3LYP-D3(BJ)]+PCM}/[6-31G(d,p) + Lanl2dz] and [wB97xd + PCM]/{[6-311+G(d,p)] + SDD} levels of theory are given in Table 1S. As seen from this Table, both the {[B3LYP-D3(BJ)]+PCM}/[6-31G(d,p) + Lanl2dz] and {[B3LYP-D3(BJ)]+PCM}/[6-31G(d,p) + Lanl2dz] and [wB97xd + PCM]/{[6-311+G(d,p)] + SDD} calculated energies lead to the same conclusions, while the calculated values of each structure at these two levels of theory differ by a few kcal/mol. Since, we have complete sets of the {[B3LYP-D3(BJ)]+PCM}/[6-31G(d,p) + Lanl2dz] calculated energies and geometries, for sake of simplicity, in this paper we discuss only the {[B3LYP-D3(BJ)]+PCM}/[6-31G(d,p) + Lanl2dz] calculated data.

Table 1S. The Gibbs free energies (in kcal/mol) of various reported reactions calculated at the {[B3LYP-D3(BJ)]+PCM}/[6-31G(d,p) + Lanl2dz] and [wB97xd + PCM]/{[6-311+G(d,p)] + SDD} levels of theory. The [wB97xd + PCM]/{[6-311+G(d,p)] + SDD} reported energies include the Gibbs free corrections from the {[B3LYP-D3(BJ)]+PCM}/[6-31G(d,p) + Lanl2dz} level calculations.

Reaction	B3LYP-D3(BJ)	wB97XD
**MeCN + $Cu_2(OAc)_4$ → (MeCN)–$Cu_2(OAc)_4$**		
	-4.9	-2.5
**Diazo + $Cu_2(OAc)_4$ → (Carbene)–$Cu_2(OAc)_4$ + N_2**		
(Diazo)–$Cu_2(AcO)_4$	-9.3	-1.9
TS(N_2-ext)	15.1	20.1
(Carbene)–$Cu_2(AcO)_4$ + N_2	-1.8	-1.7
**Diazo + $Co_2(OAc)_4$ → (Carbene)–$Co_2(OAc)_4$ + N_2**		
(Diazo)–$Co_2(OAc)_4$	-4.8	-0.7
TS(N_2-ext.)	19.1	20.9
(Carbene)–$Co_2(OAc)_4$ + N_2	4.5	7.3
**Diazo + $[Ru_2(OAc)_4]^+$ → (Carbene)–$[Ru_2(OAc)_4]^+$ + N_2**		
(Diazo)–$[Ru_2(OAc)_4]^+$	3.0	3.0
TS(N_2-ext)	12.0	10.1
(Carbene)–$[Ru_2(OAc)_4]^+$ + N_2	-15.3	-21.5
**Diazo + $Cl[Ru_2(OAc)_4]$ → (Carbene)–$Cl[Ru_2(OAc)_4]$ + N_2**		
(Diazo)–$Ru_2(OAc)_4Cl$	-5.1	-13.8
TS(N_2-ext.)	19.4	7.9
(Carbene)–$[Ru_2(OAc)_4Cl]$ + N_2	-5.9	-9.6
**$Cl[Ru_2(OAc)_4]$ → Cl^- + $[Ru_2(OAc)_4]^+$**		
	-19.3	-20.3
**Diazo + $[Rh_2(OAc)_4]$ → (Carbene)–$[Rh_2(OAc)_4]$ + N_2**		
(Diazo)–$Rh_2(OAc)_4$	-4.3	0.8
TS(N_2-ext)	7.2	11.4

(Carbene)–$Rh_2(OAc)_4$ + N_2	-15.3	-13.1

References:

1) Frisch, M. J.; Trucks, G. W.; Schlegel, H. B.; Scuseria, G. E.; Robb, M. A.; Cheeseman, J. R.; Scalmani, G.; Barone, V.; Petersson, G. A.; Nakatsuji, H.; Li, X.; Caricato, M.; Marenich, A. V.; Bloino, J.; Janesko, B. G.; Gomperts, R.; Mennucci, B.; Hratchian, H. P.; Ortiz, J. V.; Izmaylov, A. F.; Sonnenberg, J. L.; Williams-Young, D.; Ding, F.; Lipparini, F.; Egidi, F.; Goings, J.; Peng, B.; Petrone, A.; Henderson, T.; Ranasinghe, D.; Zakrzewski, V. G.; Gao, J.; Rega, N.; Zheng, G.; Liang, W.; Hada, M.; Ehara, M.; Toyota, K.; Fukuda, R.; Hasegawa, J.; Ishida, M.; Nakajima, T.; Honda, Y.; Kitao, O.; Nakai, H.; Vreven, T.; Throssell, K.; Montgomery, J. A., Jr.; Peralta, J. E.; Ogliaro, F.; Bearpark, M. J.; Heyd, J. J.; Brothers, E. N.; Kudin, K. N.; Staroverov, V. N.; Keith, T. A.; Kobayashi, R.; Normand, J.; Raghavachari, K.; Rendell, A. P.; Burant, J. C.; Iyengar, S. S.; Tomasi, J.; Cossi, M.; Millam, J. M.; Klene, M.; Adamo, C.; Cammi, R.; Ochterski, J. W.; Martin, R. L.; Morokuma, K.; Farkas, O.; Foresman, J. B.; Fox, D. J., *Gaussian 16, Revision C.01*, **Gaussian**, Inc., Wallingford CT, 2019.
2) Becke, A. D. Density-Functional Exchange-Energy Approximation with Correct Asymptotic Behavior. *Phys. Rev. A* **1988,** *38,* 3098-3100.
3) Lee, C.; Yang, W.; Parr, R. G. Development of The Colle-Salvetti Correlation-Energy Formula into a Functional of the Electron Density. *Phys. Rev. B* **1988,** *37,* 785-789.
4) Becke, A. D. A New Mixing of Hartree–Fock and Local Density-Functional Theories. *J. Chem. Phys.* **1993,** *98,* 1372-1377.
5) Grimme, S.; Antony, J.; Ehrlich, S.; Krieg, H. A Consistent and Accurate Ab Initio Parametrization of Density Functional Dispersion Correction (DFT-D) for the 94 Elements H-Pu. *J. Chem. Phys.* **2010,** *132,* 154104-154122.
6) Becke, A. D.; Johnson, E. R. A Density-Functional Model of the Dispersion Interaction. *J. Chem. Phys.* **2005,** *123,* 154101-154106.
7) Becke, A. D.; Johnson, E. R. Exchange-Hole Dipole Moment and the Dispersion Interaction. *J. Chem. Phys.* **2005,** *122,* 154104-154109.
8) Johnson, E. R.; Becke, A. D. A Post-Hartree-Fock Model of Intermolecular Interactions: Inclusion of Higher-Order Corrections. *J. Chem. Phys.* **2006,** *124,* 174104-174112.
9) (a) Hay, P. J.; Wadt, W. R. Ab Initio Effective Core Potentials for Molecular Calcdulations. Potentials for the Transition Metal Atoms Sc to Hg. *J. Chem. Phys.* **1985**, *82,* 270-283. (b) Hay, P. J.; Wadt, W. R. Ab Initio Effective Core Potentials for Molecular Calcdulations. Potentials for K to Au Including the Outermost Core Orbitals. *J. Chem. Phys.* **1985**, *82,* 299-310. (c) Wadt, W. R.; Hay, P. J. Ab Initio Effective Core Potentials for Molecular Calcdulations. Potentials for Main Group Elements Na to Bi. *J. Chem. Phys*. **1985**, *82,* 284-298.
10) Barone, V.; Cossi, M. Quantum Calcdulation of Molecular Energies and Energy Gradients in Solution by a Conductor Solvent Model. *J. Phys. Chem. A* **1998**, *102,* 1995-2001.
11) Cossi, M.; Rega, N.; Scalmani, G.; Barone, V. Energies, Structures, and Electronic Properties of Molecules in Solution with the C-PCM Solvation Model. *J. Comput. Chem.* **2003**, *24,* 669-681.
12) Chai, J.-D.; Head-Gordon, M. Long-range Corrected Hybrid Density Functionals with Damped Atom-Atom Dispersion Corrections. *Phys. Chem. Chem. Phys.* **2008**, *10,* 6615-6620;
13) Küchle, W.; Dolg, M.; Stoll, H.; Preuss, H. Energy-adjusted pseudopotentials for the actinides. Parameter sets and test calculations for thorium and thorium monoxide. *J. Chem. Phys.* **1994**, *100,* 7535-7542.
