# Supplementary material for: Diruthenium Tetracarboxylate-Catalyzed Enantioselective Cyclopropanation with Aryldiazoacetates
Source: Organometallics. 2023 Jun 30;42(15):2122–33. doi: 10.1021/acs.organomet.3c00268 (PMC10428512; doi:10.1021/acs.organomet.3c00268)
Supplement: Supplementary file 2 — om3c00268_si_002.pdf [file om3c00268_si_002.pdf]

MeCN

6

N	-0.01763000	-0.00800200	3.25127800
C	-0.01733400	-0.00746900	4.41201500
C	-0.01703300	-0.00684200	5.87074500
H	1.00122200	0.12664400	6.24471600
H	-0.64157600	0.80838200	6.24481200
H	-0.41053600	-0.95519000	6.24544200

Styrene

16

C	2.51010300	-4.02888100	4.01609600
C	3.65373200	-4.84305700	4.08045200
C	1.36747100	-4.43233100	4.73185000
C	3.66121700	-6.01879600	4.83071600
H	4.54478100	-4.54604000	3.53373600
C	1.37356800	-5.60523000	5.48094200
H	0.46835700	-3.82522700	4.70229600
C	2.52013300	-6.40514500	5.53481800
H	4.55715900	-6.63137900	4.86486300
H	0.48138100	-5.89887800	6.02605000
H	2.52096300	-7.31938300	6.12032200
C	2.55863800	-2.79969700	3.20861600
C	1.57576400	-1.90801100	3.02996300
H	3.51227200	-2.62187000	2.71287800
H	1.72642000	-1.03199800	2.40780300
H	0.59686300	-2.01226500	3.48862000

N2 molecule

2

N 0.00000000 0.00000000 0.01493300

N 0.00000000 0.00000000 1.12506700

Diazocarbene, 2

24

C	-3.34728700	9.38633900	-10.51370300
C	-3.57176900	10.21943100	-9.33723600
C	-3.53924800	9.70928800	-11.94222200
N	-2.88659600	8.18759900	-10.20681300
O	-3.90341300	11.49389900	-9.69895100
O	-3.45607400	9.84472500	-8.18476300
C	-4.59424700	10.53745700	-12.35815200
C	-2.68319400	9.16205700	-12.91103000
N	-2.48714000	7.15809600	-9.94766300
C	-4.26454300	12.38339300	-8.65352300
C	-4.77769900	10.82909800	-13.70786200
H	-5.27399200	10.95981100	-11.63085300
C	-2.86778000	9.43218900	-14.26556600
H	-1.85684100	8.52483100	-12.61257600
H	-4.07452000	11.93007100	-7.68021100
H	-3.68450600	13.29993200	-8.77035800
C	-3.91232400	10.26904400	-14.64179400
H	-5.59164900	11.47266600	-14.01928300
H	-2.20023900	9.00608000	-15.00462700
Br	-4.17534400	10.67197400	-16.55305200
C	-5.75421400	12.73193700	-8.76312700
Cl	-6.75481200	11.24748900	-8.60539200
Cl	-6.10477300	13.51068400	-10.34663200
Cl	-6.14258200	13.87413900	-7.43252800

Cyclopropanation Product

38

C	1.59605900	-3.52137500	1.78165200
C	0.84247000	-4.80442000	1.94373300
C	-0.51484800	-4.80938400	2.27223700
C	1.50651100	-6.02652000	1.77860500
C	-1.20851600	-6.00971700	2.44575700
H	-1.04362600	-3.87015100	2.39828800
C	0.83544200	-7.23559300	1.94546400
H	2.56273200	-6.03193800	1.53249100
C	-0.51534700	-7.20194000	2.28026200
H	-2.26073100	-6.00732600	2.70337000
H	1.35536500	-8.17829700	1.82484000
Br	-1.47617100	-8.90838600	2.53449900
C	2.02123400	-3.22377700	0.38889600
O	2.58277800	-1.97664600	0.29342300
O	1.92190000	-3.97657600	-0.55610300
C	3.27486500	-1.66527200	-0.90311500
H	3.06002100	-2.40363600	-1.67653200
H	2.96954800	-0.66945900	-1.22687800
C	2.97182000	-4.10283100	3.95430900
C	4.26467100	-4.64616600	3.98868400
C	2.03835900	-4.53118200	4.90786100
C	4.61644700	-5.59600800	4.94683800
H	4.99508200	-4.32175200	3.25237400
C	2.38761800	-5.48324500	5.86503200
H	1.03082200	-4.13032200	4.90312400
C	3.67645600	-6.02043600	5.88833600
H	5.62236000	-6.00468800	4.95703100
H	1.65060700	-5.80511100	6.59453000
H	3.94590400	-6.76158600	6.63443700
C	2.64943500	-3.13253800	2.87507700
C	1.36904800	-2.37998000	2.75365300
H	3.51828000	-2.63544100	2.45789700
H	1.41501600	-1.37227500	2.35960000
H	0.59936000	-2.54177100	3.49988600
C	4.78513400	-1.64628000	-0.63093400
Cl	5.34314100	-3.26774700	-0.08509900
Cl	5.61182200	-1.20310300	-2.16231600
Cl	5.17742200	-0.43676800	0.64117900

$Cu_2(OAc)_4$, 3A

30

Cu	0.01581500	0.00630100	-1.24446900
O	-1.36704100	1.40817500	-1.13074500
O	1.41661200	1.38693400	-1.09218400
O	1.39561300	-1.39696000	-1.11034800
O	-1.38805400	-1.37583000	-1.14851000
Cu	-0.01498100	-0.00657100	1.26246700
C	-1.82348900	-1.78507100	-0.02622700
O	-1.41621800	-1.38673400	1.11037200
C	1.78509500	-1.82457100	0.02183900
O	1.36734200	-1.40904200	1.14852500
C	-1.78435400	1.82417700	-0.00406500
O	-1.39428300	1.39718100	1.12814800
O	1.38946000	1.37492000	1.16670700
C	1.82451800	1.78458100	0.04442800
C	2.94042700	2.80148800	0.06288500
H	3.89491300	2.26436600	0.07105700
H	2.90376100	3.42398900	-0.83170900
H	2.88303300	3.41466000	0.96279700
C	2.80195300	-2.94065400	0.02915000
H	3.42002800	-2.88685100	0.92603300

H	2.26478800	-3.89511000	0.03762900
H	3.41959800	-2.90050500	-0.86855100
C	-2.80142900	2.94006200	-0.01139600
H	-3.41971800	2.89913000	0.88583500
H	-2.26444900	3.89463200	-0.01867800
H	-3.41883700	2.88679600	-0.90876100
C	-2.93903600	-2.80237900	-0.04464500
H	-2.90197600	-3.42497200	0.84987400
H	-3.89372200	-2.26561100	-0.05253100
H	-2.88157800	-3.41542600	-0.94463500

(MeCN)–$Cu_2(OAc)_4$, 3A

34

Cu	0.01124900	-0.00904600	-1.35583900
O	-1.40929100	1.38314000	-1.13527800
O	1.40153800	1.41059900	-1.11898200
O	1.42885700	-1.39981800	-1.11142200
O	-1.38139600	-1.42758900	-1.12808200
Cu	-0.00347000	-0.00175600	1.20630400
C	-1.82747900	-1.79201600	0.00165600
O	-1.43349500	-1.35274100	1.12856200
C	1.79023900	-1.83600000	0.02314500
O	1.34778700	-1.43231800	1.14538300
C	-1.78320100	1.82604700	-0.00740800
O	-1.35374600	1.42853800	1.12204200
O	1.42738300	1.34892800	1.13850100
C	1.83455800	1.78154200	0.01370400
C	2.95294000	2.79858500	0.03250700
H	3.90739100	2.26150000	0.04856500
H	2.92106000	3.41853500	-0.86400800
H	2.89307300	3.41576800	0.92988300
C	2.80759600	-2.95398200	0.04739100
H	3.41676700	-2.89671700	0.95039000
H	2.27085700	-3.90872600	0.05558400
H	3.43543800	-2.91891300	-0.84349700
C	-2.79994600	2.94484300	-0.00099000
H	-3.41837000	2.89400200	0.89608300
H	-2.26257600	3.89924200	0.00633300
H	-3.41867800	2.90434600	-0.89800100
C	-2.94584900	-2.80917100	0.01338200
H	-2.89826700	-3.41905200	0.91645300
H	-3.90050900	-2.27221800	0.01176600
H	-2.90152500	-3.43633200	-0.87758300
N	0.02276500	-0.00760200	-3.59224100
C	0.02515000	0.01641500	-4.74890500
C	0.02812400	0.04824700	-6.20357500
H	-0.87308700	0.55121300	-6.56254900
H	0.90837500	0.58997100	-6.55812300
H	0.05122400	-0.97177500	-6.59462300

(MeCN)–$Cu_2(OAc)_4$, 1A (open shell singlet)

34

Cu	0.01125300	-0.00905300	-1.35702600
O	-1.40887900	1.38303300	-1.13551900
O	1.40143500	1.41019200	-1.11923500
O	1.42845600	-1.39970700	-1.11167600
O	-1.38127800	-1.42718800	-1.12830000
Cu	-0.00344900	-0.00173100	1.20775200
C	-1.82625300	-1.79062600	0.00197100
O	-1.43351200	-1.35230500	1.12940700
C	1.78885300	-1.83479400	0.02340400
O	1.34738000	-1.43235100	1.14619400
C	-1.78183000	1.82482200	-0.00711600
O	-1.35332000	1.42858400	1.12286400
O	1.42745000	1.34852700	1.13931300
C	1.83335100	1.78018000	0.01396200
C	2.95179100	2.79774900	0.03280000
H	3.90601200	2.26035700	0.04886700
H	2.91990600	3.41755700	-0.86368800
H	2.89195200	3.41479400	0.93016100
C	2.80668900	-2.95287600	0.04770500
H	3.41582300	-2.89555600	0.95061400
H	2.26959800	-3.90736500	0.05609100
H	3.43429200	-2.91794000	-0.84323000
C	-2.79916600	2.94361600	-0.00064200
H	-3.41740000	2.89280200	0.89645000
H	-2.26153900	3.89781600	0.00662300
H	-3.41780300	2.90304800	-0.89759200
C	-2.94468700	-2.80829900	0.01377500
H	-2.89704800	-3.41810600	0.91678000
H	-3.89911200	-2.27102600	0.01231100
H	-2.90045700	-3.43525400	-0.87721500
N	0.02277000	-0.00757600	-3.59373900
C	0.02518100	0.01644400	-4.75041800
C	0.02821100	0.04830700	-6.20511400
H	-0.87291600	0.55138500	-6.56414400
H	0.90853000	0.58992700	-6.55965200
H	0.05120200	-0.97170000	-6.59621200

Diazo_$Cu_2(OAc)_4$, O-iso, 1A_trans (open shell singlet)

54

Cu	1.60680200	10.23489000	10.29407600
O	-0.75703500	10.82189700	12.10647400
O	-0.69521300	12.34030300	9.78287200
O	-0.83907200	8.47112600	10.55874000
O	1.47933000	10.53107800	12.22923000
O	-0.61174700	10.03299600	8.21601800
O	1.55225500	12.18983200	9.98116500
O	1.63739900	9.91951100	8.35719700
C	0.33901000	10.77062700	12.74087400
C	0.23587600	7.81072600	10.68994900
C	0.47511800	12.83316800	9.77719800
C	0.54242500	9.86721500	7.71407200
Cu	-0.93583800	10.41932500	10.16636000
C	-3.78385000	8.31156200	11.23510700
C	-3.57421400	8.98385300	9.97181700
C	-3.54772800	8.91463800	12.55826700
N	-4.03772700	7.01914400	11.16183600
O	-3.87236400	8.19787400	8.91759800
O	-3.16653700	10.13989100	9.85696500
C	-3.86329100	10.26171700	12.78413200
C	-2.99818300	8.15430200	13.60063600
N	-4.24728600	5.90581900	11.10779200
C	-3.48385200	8.65640500	7.62225700
C	-3.62818100	10.84401900	14.02709500
H	-4.27892000	10.86195300	11.98494300
C	-2.76969100	8.72261800	14.85196300
H	-2.73356200	7.11412800	13.43943600
H	-2.65159100	9.35843600	7.68974100
H	-4.34076900	9.11429900	7.12377800
C	-3.08800600	10.06310100	15.04214600
H	-3.86746000	11.88704700	14.19524700
H	-2.34300400	8.13139000	15.65308400
Br	-2.74564400	10.88435800	16.80101300

O	1.40913000	8.28831500	10.59866300
C	-3.03658100	7.43566300	6.81859600
Cl	-1.59699500	6.67912500	7.57936600
Cl	-4.35766600	6.21932100	6.71504500
Cl	-2.62167600	8.01094700	5.16747900
C	0.27875400	10.97723500	14.23621600
H	-0.48166500	11.71811400	14.48404800
H	-0.02000100	10.03193500	14.69974400
H	1.25232600	11.27320200	14.62656800
C	0.61024100	14.30931000	9.47965500
H	1.00907600	14.42536900	8.46685100
H	-0.35558000	14.80941100	9.54446800
H	1.32246600	14.76257900	10.17216000
C	0.62239400	9.55079900	6.23935500
H	0.52455200	8.46745500	6.11706600
H	-0.19952700	10.02566400	5.70329700
H	1.58398000	9.86338200	5.83168500
C	0.09637600	6.34218900	11.01822200
H	-0.04081400	6.24563900	12.10059700
H	-0.78618800	5.92964100	10.52744100
H	0.99201500	5.79221100	10.72915700

Diazo_$Cu_2(OAc)_4$, O-iso, 3A_trans, triplet

54

Cu	1.60815600	10.25416900	10.28001000
O	-0.74980600	10.78655100	12.11794600
O	-0.71486600	12.32653800	9.80576100
O	-0.82137400	8.44960800	10.55690100
O	1.48349000	10.47588300	12.22613100
O	-0.60959000	10.02247600	8.22149900
O	1.53042400	12.21876600	10.04055000
O	1.64279900	10.01085000	8.33188600
C	0.34692500	10.70961300	12.74951500
C	0.26451000	7.79721200	10.61745800
C	0.44463600	12.84541900	9.83001500
C	0.54342200	9.91109800	7.70141400
Cu	-0.93162800	10.40107600	10.17529600
C	-3.78332300	8.31665400	11.26361300
C	-3.55789500	8.95552300	9.98548000
C	-3.57562300	8.95836400	12.57333100
N	-4.02759700	7.02101800	11.22157800
O	-3.82999100	8.13963000	8.94767300
O	-3.15848900	10.11205800	9.84746700
C	-3.87925900	10.31582900	12.74913200
C	-3.06391200	8.22466400	13.65348700
N	-4.22760500	5.90490900	11.19432500
C	-3.44453900	8.58185100	7.64516200
C	-3.66494000	10.93475400	13.97806600
H	-4.26773300	10.89562200	11.92192800
C	-2.85680400	8.83012600	14.89096400
H	-2.81099100	7.17612100	13.53317300
H	-2.61925000	9.29294200	7.70289400
H	-4.30578600	9.02410400	7.13997700
C	-3.15809000	10.18074100	15.02984900
H	-3.89348400	11.98582100	14.10658000
H	-2.45807700	8.25914600	15.72065800
Br	-2.83729000	11.05582100	16.76657600
O	1.42777600	8.29626700	10.50210500
C	-2.98518100	7.35484300	6.85860000
Cl	-1.53403500	6.62617600	7.62377500
Cl	-4.29183400	6.12146200	6.77619100
Cl	-2.58231100	7.91244200	5.19799100
C	0.29018500	10.87735600	14.24901600
H	-0.43464600	11.64811200	14.51357600
H	-0.05890600	9.93671000	14.68610300
H	1.27452900	11.11600000	14.65138600
C	0.55221400	14.32832000	9.56093900
H	0.91572900	14.47075600	8.53801200
H	-0.41821700	14.81321300	9.66463300
H	1.27949700	14.77797000	10.23973000
C	0.61489000	9.60316000	6.22537400
H	0.52005200	8.51999600	6.09802000
H	-0.21424300	10.07625100	5.69822600
H	1.57105100	9.92320700	5.81092700
C	0.17227400	6.30988200	10.86548000
H	0.54093500	6.09694100	11.87373100
H	-0.85733600	5.96562400	10.77542900
H	0.81322200	5.77711100	10.15921600

TS(N2-ext.), $Cu_2(OAc)_4$, 3A, trans

54

Cu	2.00259800	10.52913000	-10.05023200
O	-0.09794800	10.86502900	-12.13825700
O	-0.62072300	12.21557400	-9.71083300
O	-0.27653300	8.43292000	-10.69211300
O	2.14104900	11.00091400	-11.94178000
O	-0.17909800	9.82214200	-8.21393900
O	1.62571400	12.42178800	-9.58192300
O	2.05644000	10.06820000	-8.15730400
C	1.06013200	11.06534200	-12.60933000
C	0.90102200	7.95565500	-10.74084800
C	0.44389900	12.87691100	-9.50256800
C	0.93322500	9.80897300	-7.61457700
Cu	-0.59766100	10.31149500	-10.19786200
O	1.97332400	8.60213900	-10.53305000
C	-2.83403700	9.94178100	-10.15297100
C	-3.09871800	9.56274700	-8.76133000
C	-3.52899300	9.29757000	-11.25298300
N	-3.41013200	11.71928000	-10.19713400
O	-3.19667000	10.59824400	-7.90014000
O	-3.06375900	8.39348100	-8.41694800
C	-4.73893800	8.59320700	-11.07252100
C	-2.95243000	9.35580300	-12.53883500
N	-3.37397300	12.78388400	-10.49976300
C	-3.00617400	10.26007900	-6.52954100
C	-5.35068400	7.94995000	-12.13994700
H	-5.18901000	8.53739700	-10.08787100
C	-3.56313500	8.72822400	-13.61882500
H	-2.01889700	9.89101700	-12.67758800
H	-2.06785500	9.71664400	-6.41275100
H	-3.83890400	9.66387600	-6.15095300
C	-2.93024000	11.56711200	-5.74521600
C	-4.75079400	8.03378600	-13.39747300
H	-6.27453800	7.40193700	-12.00220200
H	-3.12109800	8.77073300	-14.60663900
Cl	-1.55259900	12.57183600	-6.30510100
Cl	-4.45648200	12.50054600	-5.92623800
Cl	-2.69242100	11.13091400	-4.01591800
Br	-5.61316100	7.13301900	-14.91053900
C	0.93492000	9.49147900	-6.13597700
H	1.93760100	9.23616700	-5.79337900
H	0.24349500	8.67272200	-5.92625400
H	0.58728100	10.37578100	-5.59262200
C	1.01981200	6.47956600	-11.04376800

H	0.19376600	6.14301000	-11.67207700
H	0.96001300	5.93940100	-10.09232800
H	1.97886800	6.25747200	-11.51125500
C	1.16391900	11.37633600	-14.08573400
H	0.38324000	12.08532900	-14.37004900
H	0.98768200	10.45155300	-14.64487400
H	2.14790400	11.76897500	-14.33898300
C	0.29333900	14.34205100	-9.16451000
H	1.07212500	14.65506900	-8.46683100
H	-0.69564200	14.53298400	-8.74934300
H	0.40967800	14.92720800	-10.08349400

TS(N2-ext), $Cu_2(OAc)_4$, trans_1A (open_shell singlet)

54

Cu	2.01550800	10.52692300	-10.05763400
O	-0.13475100	10.77379100	-12.13245400
O	-0.57016000	12.21989500	-9.76374800
O	-0.21416200	8.39336100	-10.63015700
O	2.09329000	11.05372300	-11.94111500
O	-0.22151000	9.87930800	-8.20074000
O	1.67067700	12.40641000	-9.53383300
O	2.02831700	10.01319200	-8.17371700
C	1.00858100	11.05268300	-12.60306200
C	0.96913400	7.95117000	-10.76192000
C	0.48831700	12.86762100	-9.49909500
C	0.90001700	9.81541700	-7.61924600
Cu	-0.57295300	10.28907200	-10.19220700
O	2.03616900	8.61758500	-10.59221500
C	-2.83144300	9.91741500	-10.14922300
C	-3.10121100	9.58375200	-8.74858700
C	-3.53948500	9.24559300	-11.22507100
N	-3.38683100	11.70113200	-10.25300000
O	-3.19899400	10.64046500	-7.91299000
O	-3.07193600	8.42345800	-8.37241100
C	-4.75726500	8.56198100	-11.01804600
C	-2.96794000	9.25483800	-12.51427900
N	-3.33161700	12.75220700	-10.59816900
C	-3.01652700	10.32856800	-6.53516300
C	-5.38080100	7.89159000	-12.06166800
H	-5.20443600	8.54370100	-10.03065500
C	-3.59021700	8.59994100	-13.57141600
H	-2.03004100	9.77612400	-12.67311000
H	-2.08883600	9.77038100	-6.40440500
H	-3.86022500	9.75377900	-6.14779200
C	-2.92192200	11.64734300	-5.77262400
C	-4.78504200	7.92745300	-13.32345400
H	-6.31062600	7.35952300	-11.90302200
H	-3.15185800	8.60563200	-14.56178400
Cl	-1.52010600	12.61537700	-6.33459500
Cl	-4.42841700	12.60676800	-5.98297000
Cl	-2.70941800	11.23622600	-4.03351500
Br	-5.66493900	6.98942100	-14.80373900
C	0.89952600	9.50250800	-6.13924900
H	1.90373000	9.26966800	-5.78641800
H	0.22491200	8.66734300	-5.93797200
H	0.52200900	10.37746600	-5.60099400
C	1.11689800	6.50959300	-11.19445600
H	1.18410900	6.48394400	-12.28741400
H	0.24802100	5.92677700	-10.88760300
H	2.03289900	6.08148800	-10.78540700
C	1.07916100	11.43755800	-14.06395600
H	0.63771300	12.43180200	-14.18551500
H	0.48741700	10.73731500	-14.65758500
H	2.11060300	11.45645800	-14.41420000
C	0.33211200	14.31863200	-9.10235800
H	0.70534500	14.45357800	-8.08313500
H	-0.71206400	14.62460000	-9.15514200
H	0.94106400	14.94283600	-9.76128900

(Carbene)–$Cu_2(OAc)_4$, 3A

52

Cu	0.17936900	-0.31647600	-1.18342400
O	-1.15485600	0.94813700	-1.83179100
O	1.48351400	1.16110100	-0.87291100
O	1.58392000	-1.63663800	-0.98949100
O	-1.22627400	-1.70683100	-0.88703500
Cu	-0.39172200	0.15090700	1.39867700
C	-1.81488900	-1.96484000	0.20624900
O	-1.63414000	-1.36841500	1.31533200
C	1.87284500	-1.87482500	0.23585100
O	1.30702000	-1.33029600	1.21529400
C	-1.88143600	1.49820800	-0.94162400
O	-1.77697000	1.28615800	0.29846600
O	0.89269600	1.63417700	1.26072800
C	1.57890500	1.81954300	0.20640200
C	2.57681900	2.95617400	0.23337800
H	3.45439100	2.70220700	-0.36357200
H	2.10832300	3.84040600	-0.21180300
H	2.86580200	3.19052600	1.25785200
C	2.99622500	-2.85961800	0.48581700
H	3.91417700	-2.29641200	0.68279600
H	2.77431200	-3.46555500	1.36663700
H	3.15901600	-3.50080600	-0.38089800
C	-2.91292500	2.49829900	-1.41442000
H	-3.75755900	2.52803500	-0.72487800
H	-2.44919800	3.49050700	-1.43292700
H	-3.24939800	2.25857900	-2.42381800
C	-2.85425100	-3.06389200	0.18330200
H	-3.01774300	-3.46231500	1.18494500
H	-3.79599600	-2.64160600	-0.18350600
H	-2.55012900	-3.85755700	-0.50105700
C	-0.33366900	0.29273900	3.46587500
C	0.67481200	-0.60431700	4.01307300
O	0.39257800	-1.72995400	4.38439900
O	1.91897900	-0.10116000	3.93904800
C	-1.11205300	1.14417900	4.25392600
C	-2.09630000	1.95768600	3.61233900
C	-0.94528700	1.25315400	5.66963300
C	-2.86762400	2.84084100	4.34359300
H	-2.22154700	1.87896500	2.53633400
C	-1.71925500	2.12613600	6.40512300
H	-0.20623000	0.63612900	6.16907200
C	-2.66796800	2.90820400	5.72794800
H	-3.61068700	3.46454900	3.86311000
H	-1.60100800	2.21241900	7.47776900
Br	-3.75348400	4.13960000	6.76222600
C	2.96685600	-1.06722100	4.03624400
H	2.81578700	-1.83768300	3.27920100
H	2.99774500	-1.51295200	5.03190100
C	4.28731600	-0.34933000	3.77381300
Cl	5.58891400	-1.58546400	3.90432200
Cl	4.31687000	0.37810200	2.13529400
Cl	4.55520800	0.93850900	4.99992700

(Carbene)–$Cu_2(OAc)_4$, 1A (open shell)

52

Cu	0.17716700	-0.31622500	-1.19713900
O	-1.15763900	0.95489800	-1.82022800
O	1.48959700	1.15953900	-0.88837100
O	1.57766800	-1.63629200	-0.99362700
O	-1.23245000	-1.70924400	-0.90676200
Cu	-0.37782600	0.13339400	1.39420400
C	-1.81009200	-1.97336400	0.19035100
O	-1.62038800	-1.38553700	1.30249100
C	1.87033100	-1.87325500	0.23013100
O	1.30647800	-1.33186800	1.21316400
C	-1.88022200	1.49597600	-0.92158300
O	-1.77090700	1.27474600	0.31638400
O	0.90457700	1.61868100	1.25098100
C	1.58539000	1.81109500	0.19460200
C	2.58027000	2.95135300	0.22486400
H	3.45955300	2.70039800	-0.37078100
H	2.10961400	3.83390000	-0.22130000
H	2.86649600	3.18613000	1.24987600
C	2.99765600	-2.85409200	0.47820000
H	3.91205300	-2.28698500	0.68031300
H	2.77633400	-3.46470100	1.35588100
H	3.16532100	-3.49044800	-0.39102000
C	-2.91530800	2.49872100	-1.38168300
H	-3.75512000	2.52426100	-0.68625300
H	-2.45137000	3.49083800	-1.39756800
H	-3.25835700	2.26471000	-2.39014400
C	-2.85074500	-3.07232500	0.17010100
H	-3.00659300	-3.47607900	1.17071300
H	-3.79480100	-2.64703500	-0.18705700
H	-2.55251200	-3.86216200	-0.52113900
C	-0.33680800	0.29162800	3.47105600
C	0.66993100	-0.60324200	4.02293800
O	0.38894000	-1.72929800	4.39491000
O	1.91496000	-0.10024400	3.94868400
C	-1.11433200	1.14747200	4.25600400
C	-2.09612700	1.95990100	3.60980300
C	-0.94945800	1.26198700	5.67134100
C	-2.86723000	2.84744300	4.33638100
H	-2.21915700	1.87649100	2.53391500
C	-1.72309900	2.13920700	6.40233400
H	-0.21203400	0.64583200	6.17432300
C	-2.66951000	2.91997000	5.72057500
H	-3.60853500	3.47050500	3.85232500
H	-1.60627500	2.22970500	7.47479700
Br	-3.75486000	4.15748200	6.74863500
C	2.96242300	-1.06682800	4.04279800
H	2.81094500	-1.83566300	3.28410600
H	2.99422900	-1.51506100	5.03731000
C	4.28303000	-0.34892900	3.78101300
Cl	5.58433900	-1.58590900	3.90699200
Cl	4.31153200	0.38301900	2.14449500
Cl	4.55272000	0.93551800	5.01040600

(Carbene)–$Cu_2(OAc)_4$, ^{1}A (closed shell)

52

Cu	0.09848800	-0.30899400	-1.06711100
O	-1.07212200	1.13712300	-1.44439300
O	1.55398600	0.89048600	-0.98144000
O	1.30696900	-1.75093100	-0.82166400
O	-1.32379300	-1.52946700	-1.30739600
Cu	-0.48366500	-0.12617000	1.67727500
C	-1.76867000	-2.12882400	-0.25336600
O	-1.44073900	-1.87767600	0.92006400
C	1.77523200	-1.90479400	0.37247300
O	1.39355900	-1.29900400	1.38854000
C	-1.95226900	1.44835700	-0.55320900
O	-2.05533900	0.94956900	0.58312400
O	0.75150300	1.63490200	0.98899000
C	1.54386600	1.69162300	0.03637700
C	2.59807500	2.78233200	-0.03330000
H	3.52381400	2.40143400	-0.46715200
H	2.22408900	3.58174400	-0.68081500
H	2.77741100	3.18184600	0.96445300
C	2.90697400	-2.91190300	0.48259200
H	3.82419500	-2.36050700	0.71063000
H	2.69984500	-3.58599700	1.31670100
H	3.04053400	-3.47788400	-0.43805500
C	-2.89683000	2.55669400	-0.98481600
H	-3.78454300	2.55083000	-0.35272500
H	-2.37821000	3.51329700	-0.86563200
H	-3.16902100	2.44590300	-2.03522600
C	-2.76533200	-3.23368500	-0.55243400
H	-3.20416500	-3.59827500	0.37550600
H	-3.54400500	-2.86121500	-1.22151600
H	-2.24693100	-4.04990800	-1.06359300
C	-0.42047300	0.23604300	3.60947400
C	0.64208700	-0.56653800	4.20913000
O	0.44656300	-1.66591100	4.69999200
O	1.86469200	-0.01454500	4.04110600
C	-1.13478000	1.17944300	4.38130500
C	-2.16172700	1.93441800	3.74601400
C	-0.85455200	1.42891300	5.75593600
C	-2.86943000	2.89852600	4.44382000
H	-2.36999700	1.74535400	2.69727700
C	-1.56391200	2.38144600	6.46329200
H	-0.07823600	0.85980900	6.25640700
C	-2.55958400	3.10252200	5.79173700
H	-3.64652200	3.47895600	3.96245800
H	-1.35618600	2.57188300	7.50873100
Br	-3.55666000	4.45061900	6.78446100
C	2.95663400	-0.92373500	4.17007200
H	2.84167400	-1.73220500	3.44626100
H	3.01669500	-1.32587600	5.18280500
C	4.24101100	-0.15949400	3.86564900
Cl	5.60080200	-1.32663100	4.04190000
Cl	4.23092800	0.49073400	2.19382500
Cl	4.45787100	1.19537100	5.02820600

$Co_2(AcO)_4$, ^{3}A

30

Co	0.03498500	0.04910200	-1.19162700
O	-1.29046100	1.46027600	-1.05477600
O	1.44007600	1.36181400	-1.15318700
O	1.35370100	-1.37063300	-1.07896300
O	-1.36879500	-1.26298900	-1.28179900
Co	-0.01296700	-0.01867800	1.25894900
C	-1.81212900	-1.69395500	-0.17681100
O	-1.39124600	-1.30456700	0.96385800
C	1.71694800	-1.82167000	0.05141200
O	1.29155900	-1.41401700	1.18454700
C	-1.70753900	1.83982900	0.08319600
O	-1.31641300	1.37905400	1.20830600

O 1.37778600 1.27920600 1.08939200
C 1.84113400 1.73079900 -0.01058400
C -2.77886700 2.90052900 0.11955900
H -2.75487800 3.49966700 -0.79087600
H -3.75436900 2.40702400 0.18697100
H -2.65783600 3.53311100 1.00008400
C -2.89512600 -2.73994100 -0.18474700
H -3.25125800 -2.91931300 -1.19845400
H -2.49587300 -3.66866300 0.23379800
H -3.72073100 -2.41774300 0.45446500
C 2.91924900 2.77809700 0.07856600
H 2.48569100 3.69916400 0.48022200
H 3.69731600 2.44881300 0.77102400
H 3.34601200 2.97656500 -0.90392300
C 2.70892100 -2.95742500 0.06615900
H 2.15545300 -3.90199800 0.10031500
H 3.31655300 -2.94486000 -0.83899800
H 3.33983200 -2.90079900 0.95419900

$Co_2(AcO)_4$, ^1A (open shell)
30
Co 0.03293900 0.04432100 -1.16373100
O -1.26601500 1.45657500 -1.10351500
O 1.44478500 1.34034400 -1.05369300
O 1.32895500 -1.36990600 -1.12126800
O -1.38095300 -1.25295400 -1.17026900
Co -0.01075200 -0.01231600 1.19264300
C -1.82915000 -1.68945500 -0.06161100
O -1.42301800 -1.30862500 1.08299500
C 1.70790400 -1.83346200 0.00201200
O 1.28789800 -1.42484000 1.13203100
C -1.69567700 1.85613100 0.02600500
O -1.30708500 1.40165700 1.14982100
O 1.40268100 1.28490600 1.19965400
C 1.85062300 1.72140900 0.09089400
C -2.76440400 2.91742000 0.03354500
H -2.68979900 3.54120700 -0.85764000
H -3.74243600 2.42420800 0.02951000
H -2.69146600 3.52468300 0.93646300
C -2.89067000 -2.75693400 -0.10868300
H -3.50534800 -2.64330800 -1.00233900
H -2.39773100 -3.73408900 -0.15302900
H -3.50704900 -2.72267500 0.79008400
C 2.91144600 2.78962200 0.13741900
H 2.41781000 3.76677100 0.17379700
H 3.52229500 2.68110800 1.03428400
H 3.53179400 2.75085500 -0.75847700
C 2.69847700 -2.96813200 -0.00744400
H 2.14531500 -3.91322100 -0.02769100
H 3.32645500 -2.91802400 -0.89751100
H 3.30890400 -2.94747500 0.89602200

(Diazo)–$Co_2(AcO)_4$, ^3A_trans
54
Co 1.57871000 10.23844500 -10.70500500
O 0.05083100 12.20542600 -12.21629500
O -0.02872800 12.57590500 -9.53237900
O -1.17532600 9.84615600 -11.95093000
O 2.11648900 11.32745000 -12.19768700
O -0.94012700 10.06329900 -9.26834300
O 2.04926700 11.67712000 -9.48859000
O 1.09300400 9.11641900 -9.21925500
C 1.23573600 12.10347300 -12.67434900
C -0.33268100 8.95136000 -12.29478600
C 1.18821200 12.54401000 -9.14489600
C -0.09396800 9.23789100 -8.79233800
Co -0.66520000 11.23533500 -10.74115700
C -4.00356400 11.12270900 -11.39666800
C -4.14519300 12.52802400 -11.03517800
C -4.04753700 9.96762900 -10.48168800
N -3.77731000 10.89318000 -12.67808700
O -4.01946400 13.33066700 -12.14032700
O -4.35649400 12.95562700 -9.91631800
C -4.24742100 10.14300100 -9.10298400
C -3.85956100 8.66504400 -10.97503100
N -3.58483500 10.67345800 -13.77252000
C -4.11173700 14.73285300 -11.93837900
C -4.24120300 9.04905400 -8.23921000
H -4.39522800 11.13613400 -8.70410900
C -3.84997200 7.56721000 -10.12091500
H -3.70311200 8.49394000 -12.03301000
H -4.77971900 15.13519400 -12.70083100
H -4.49061900 14.95220400 -10.93985800
C -4.03644400 7.77806400 -8.75895900
H -4.38885000 9.19825200 -7.17627100
H -3.69815000 6.57009700 -10.51639200
Br -3.99864800 6.23064100 -7.53577700
O 0.87997300 8.89841300 -11.92353300
C -2.73391500 15.38969500 -12.10065700
Cl -2.07863800 15.06133500 -13.74680800
Cl -1.59138000 14.76904000 -10.87754100
Cl -2.96580800 17.16505500 -11.89518300
C 1.57941400 12.94588200 -13.87401100
H 1.11547000 13.92966600 -13.78479400
H 1.17159900 12.46240400 -14.76798000
H 2.65989100 13.03682100 -13.98384400
C 1.61477400 13.61856100 -8.17677200
H 1.24644200 13.35744600 -7.17912800
H 1.16953500 14.57461200 -8.45845700
H 2.70121800 13.69705400 -8.14317600
C -0.56226200 8.34071300 -7.67885200
H -1.14392600 7.52211200 -8.11361700
H -1.22303100 8.88948100 -7.00626600
H 0.28669100 7.92638600 -7.13542000
C -0.84826100 7.85543000 -13.19253900
H -1.54000900 8.26761800 -13.92934800
H -1.39921300 7.13560300 -12.57795800
H -0.02481300 7.33994200 -13.68629800

(Diazo)–$Co_2(AcO)_4$, ^1A_trans (open shell)
54
Co 1.55152100 10.21944300 -10.79000800
O 0.19084600 12.31950700 -12.44540500
O 0.27709400 12.71989900 -9.75264200
O -1.14243200 10.00360600 -12.06820900
O 2.17302400 11.25823900 -12.28059500
O -1.07156600 10.40973200 -9.37363800
O 2.20394400 11.56325500 -9.58803100
O 0.84503700 9.22642700 -9.31027900
C 1.38082100 12.08938500 -12.83143600
C -0.37204500 9.04482900 -12.39491800
C 1.45675100 12.55298200 -9.30367700
C -0.32143800 9.50891500 -8.88139000
Co -0.48637700 11.38892000 -10.91234700

C	-4.04511100	11.03902000	-11.22366700
C	-4.14950000	12.42906300	-10.80008700
C	-4.07925600	9.85210100	-10.35278900
N	-3.88666600	10.85644900	-12.52220300
O	-4.12525100	13.27116200	-11.88289600
O	-4.26060400	12.81464200	-9.65175700
C	-4.46762000	9.95542900	-9.00737500
C	-3.72278900	8.59064700	-10.85713500
N	-3.75119800	10.67965300	-13.63302300
C	-4.22859700	14.66328100	-11.62619900
C	-4.48007200	8.83123900	-8.18363500
H	-4.74501500	10.91730500	-8.60039100
C	-3.73289600	7.46188700	-10.04331900
H	-3.41677900	8.47989400	-11.88946500
H	-4.99157800	15.06989300	-12.29124100
H	-4.49253300	14.84173900	-10.58348000
C	-4.10791700	7.60146300	-8.71176200
H	-4.77476700	8.92391500	-7.14514600
H	-3.44693700	6.49718300	-10.44502500
Br	-4.08385000	6.01699700	-7.53762200
O	0.82944000	8.91544100	-11.99824600
C	-2.89604600	15.36064500	-11.92684400
Cl	-2.42953800	15.09807700	-13.64725400
Cl	-1.60444500	14.73349400	-10.86431100
Cl	-3.13929200	17.12194000	-11.63762900
C	1.87796400	12.82950600	-14.04542700
H	1.32336200	13.75838400	-14.17989900
H	1.72155700	12.19830900	-14.92674100
H	2.94691100	13.02831200	-13.95600600
C	1.98681300	13.58565400	-8.34435500
H	1.62453500	13.34500600	-7.33916400
H	1.61193100	14.57484300	-8.61145000
H	3.07676900	13.57709700	-8.33373100
C	-0.85413500	8.69791200	-7.73066900
H	-1.22960400	7.74666300	-8.12000500
H	-1.67933000	9.21717300	-7.24545600
H	-0.05499800	8.48415300	-7.01902500
C	-0.93267000	7.97080200	-13.28982200
H	-1.73194900	8.37181000	-13.91386700
H	-1.34498100	7.17380000	-12.66179100
H	-0.14362700	7.54142000	-13.90822200

(Diazo)–$Co_2(AcO)_4$, ^{3}A_cis

54

Co	1.72582200	11.00475200	-10.55409700
O	-0.78189800	11.37335700	-12.23399100
O	-0.64229300	12.47902200	-9.78213000
O	-0.28412700	8.94616100	-11.12310700
O	1.46712300	11.58003400	-12.38688100
O	-0.49852600	10.03405900	-8.67070200
O	1.56763200	12.82551900	-9.95005900
O	1.73731300	10.38875300	-8.71060000
C	0.29085000	11.65731900	-12.86256000
C	0.89328100	8.51224700	-11.35029400
C	0.39741600	13.20867600	-9.65875500
C	0.66453400	10.01857500	-8.14200700
Co	-0.70090700	10.68869700	-10.45974300
C	-3.41686500	8.81014600	-10.82506700
C	-3.48276000	9.46297100	-9.52382500
C	-3.35667500	9.42982200	-12.16441200
N	-3.34714100	7.49614900	-10.74046400
O	-3.16321800	10.79518600	-9.64513900
O	-3.73448300	8.92199700	-8.46601200
C	-4.05833700	10.61513500	-12.42888000
C	-2.60598300	8.83674100	-13.18986100
N	-3.26289400	6.36653700	-10.68064000
C	-3.29269500	11.60367500	-8.47829800
C	-3.98683800	11.21623400	-13.68294700
H	-4.65629600	11.07857500	-11.65530300
C	-2.54334800	9.41666400	-14.45386800
H	-2.04133400	7.93226100	-12.99624200
H	-3.56669300	10.98934600	-7.62147900
H	-2.33826000	12.10278600	-8.31834800
C	-3.22951100	10.60531100	-14.67562600
H	-4.52348100	12.13695700	-13.87761200
H	-1.95746700	8.95567200	-15.23990700
Br	-3.12265400	11.45168400	-16.45320600
O	1.94627500	9.18980400	-11.16482200
C	-4.37374100	12.65868800	-8.71911800
Cl	-5.96247500	11.86532500	-9.01588000
Cl	-3.95947700	13.68742700	-10.13215400
Cl	-4.46737200	13.67458600	-7.24027500
C	0.13278500	12.08809300	-14.29766000
H	-0.74409600	12.72823900	-14.40339700
H	-0.03870500	11.19690400	-14.90947300
H	1.02924200	12.59753600	-14.65044400
C	0.19588800	14.59261400	-9.10236900
H	0.06902500	14.51632300	-8.01729300
H	-0.71420200	15.03161400	-9.51513900
H	1.05891700	15.22323900	-9.31447000
C	0.75852000	9.48421400	-6.73535000
H	0.77520200	8.38997400	-6.77909700
H	-0.11788000	9.78437000	-6.15853500
H	1.67306500	9.82959400	-6.25335600
C	1.01169800	7.11781400	-11.90521300
H	0.84290000	7.16069400	-12.98665600
H	0.24714400	6.47267200	-11.46932500
H	2.00721400	6.71355500	-11.72226200

(Diazo)–$Co_2(AcO)_4$, ^{1}A_cis (open shell)

54

Co	1.66588600	11.02343500	-10.56671400
O	-0.87838900	11.42920300	-12.08940800
O	-0.67854400	12.65656100	-9.68627800
O	-0.50071400	8.99919300	-10.86860700
O	1.35245600	11.59290900	-12.36852000
O	-0.33609700	10.25723500	-8.47170200
O	1.54742600	12.84303400	-9.96863300
O	1.89267000	10.44186000	-8.74959100
C	0.15199700	11.69258300	-12.78435000
C	0.63724300	8.54258200	-11.20537900
C	0.41395900	13.30843500	-9.63221100
C	0.86339000	10.16746000	-8.05487500
Co	-0.67305100	10.82535100	-10.28116600
C	-3.39482100	8.71835200	-10.97150200
C	-3.32390200	9.31002500	-9.65009600
C	-3.33609000	9.38403800	-12.28774600
N	-3.40987900	7.40022700	-10.94253000
O	-2.93910200	10.64587500	-9.72014500
O	-3.53248700	8.74190300	-8.59822700
C	-4.06508800	10.55765200	-12.51757500
C	-2.56345700	8.84204200	-13.32353600
N	-3.40384500	6.26597400	-10.92796700
C	-3.19528300	11.43202700	-8.54957800

C	-4.00762100	11.19712200	-13.75340700
H	-4.67883400	10.97801200	-11.73198200
C	-2.51561300	9.45713400	-14.57197000
H	-1.98185300	7.94308100	-13.15397600
H	-3.42171900	10.78111300	-7.70664600
H	-2.30783500	12.02973200	-8.35638200
C	-3.23589800	10.63152000	-14.76191800
H	-4.56716300	12.10895800	-13.92378200
H	-1.91855500	9.03358400	-15.37054100
Br	-3.15795800	11.52371200	-16.51853100
O	1.72360100	9.20247000	-11.16148700
C	-4.37680800	12.37016800	-8.80717400
Cl	-5.87205800	11.42289800	-9.14375100
Cl	-4.04574400	13.45577500	-10.19690900
Cl	-4.60524200	13.34801200	-7.31667200
C	-0.05905200	12.12373300	-14.21114800
H	-1.00842000	12.64860300	-14.31318800
H	-0.10279400	11.22975300	-14.84100600
H	0.76926400	12.74752900	-14.54835500
C	0.35330800	14.71651900	-9.10207500
H	0.41626700	14.67880900	-8.00917000
H	-0.59638500	15.18028300	-9.37207900
H	1.19074900	15.30480400	-9.47772700
C	1.07253500	9.66876500	-6.64895700
H	1.04505200	8.57390500	-6.66084400
H	0.26828700	10.01994700	-6.00062500
H	2.04199300	9.98918700	-6.26717300
C	0.69134500	7.13034200	-11.72691900
H	0.60374600	7.16069500	-12.81851800
H	-0.13757200	6.54396200	-11.32923200
H	1.64591000	6.66621600	-11.47587100

(Carbene)–$Co_2(AcO)_4$, ^{3}A

52

Co	0.11782500	0.04845200	-1.09971800
O	-1.32153300	1.29355100	-0.98355300
O	1.34921500	1.45132500	-0.79739800
O	1.55215500	-1.19324100	-1.09808900
O	-1.12126200	-1.38116500	-1.00321200
Co	0.06206900	-0.12043400	1.36966200
C	-1.53216400	-1.93349500	0.06910300
O	-1.17474200	-1.57564700	1.23013600
C	1.92761700	-1.70699600	0.00984700
O	1.45952900	-1.41877000	1.15356000
C	-1.78240200	1.61756400	0.15816600
O	-1.36951100	1.16792400	1.27615900
O	1.29654100	1.35568500	1.44174900
C	1.70392100	1.86190300	0.35677700
C	-2.89327100	2.63301200	0.20410700
H	-3.32832300	2.77464800	-0.78468300
H	-3.65602800	2.31352600	0.91713100
H	-2.48296300	3.58443800	0.55728000
C	-2.47462500	-3.09798400	-0.05430600
H	-3.09082200	-2.99448800	-0.94807700
H	-1.88275900	-4.01477400	-0.14682100
H	-3.09597300	-3.17520700	0.83828700
C	2.64495100	3.03293300	0.41703900
H	2.11026100	3.93036400	0.09069100
H	3.01382200	3.17725800	1.43132400
H	3.47839400	2.87110300	-0.26957800
C	2.99292400	-2.76883000	-0.05823000
H	2.52696000	-3.70737000	-0.37631100
H	3.74433600	-2.49576200	-0.80100500
H	3.45460300	-2.90919600	0.91767700
C	0.04468700	-0.18048700	3.29414800
C	-1.10345200	-0.53982300	4.07010200
C	-2.42854300	-0.51633000	3.55839200
C	-0.93765900	-0.96331600	5.42103700
C	-3.51478100	-0.88727000	4.33901800
H	-2.59561300	-0.18492700	2.54583200
C	-2.01783900	-1.33851200	6.20618900
H	0.05514700	-0.99539000	5.85239800
C	-3.29666100	-1.29764300	5.65256500
H	-4.51762400	-0.85178500	3.93000500
H	-1.86524500	-1.66212900	7.22888800
Br	-4.83500700	-1.83220500	6.75753900
C	1.27550800	0.25628900	3.95465700
O	1.29757500	1.00019900	4.92844400
O	2.41804500	-0.20652500	3.39102500
C	3.60704300	0.39183400	3.87698300
H	3.75073600	0.18752900	4.94051900
H	3.58945500	1.47311200	3.71890600
C	4.78074300	-0.20188500	3.10098000
Cl	6.28236100	0.55825800	3.74147800
Cl	4.86394500	-1.98093100	3.34644200
Cl	4.64470900	0.15127700	1.34510000

(Carbene)–$Co_2(AcO)_4$, ^{1}A (open shell)

52

Co	0.11665500	0.04906200	-1.09820200
O	-1.32695800	1.28950900	-0.98195700
O	1.34224000	1.45487300	-0.79549800
O	1.55502500	-1.18812800	-1.09933800
O	-1.11785600	-1.38419400	-1.00572200
Co	0.06390600	-0.12344100	1.36900000
C	-1.52996500	-1.93614500	0.06642200
O	-1.17394500	-1.57771200	1.22765400
C	1.93046200	-1.70558200	0.00699600
O	1.46121500	-1.42213500	1.15132900
C	-1.78350300	1.61472000	0.16113900
O	-1.36617500	1.16631300	1.27799800
O	1.29995300	1.35130500	1.44334600
C	1.70196300	1.86181900	0.35863600
C	-2.89424400	2.63013000	0.21038500
H	-3.33379600	2.77002900	-0.77666600
H	-3.65369900	2.31190600	0.92747500
H	-2.48233900	3.58222500	0.55990200
C	-2.47187100	-3.10098900	-0.05777400
H	-3.08639900	-2.99853700	-0.95280500
H	-1.87961500	-4.01773600	-0.14815800
H	-3.09488900	-3.17746900	0.83372300
C	2.64230400	3.03334800	0.41823000
H	2.10668000	3.93081600	0.09359300
H	3.01294200	3.17676200	1.43200300
H	3.47466700	2.87245800	-0.26995300
C	2.99709000	-2.76587600	-0.06456000
H	2.53218200	-3.70397800	-0.38547500
H	3.74807600	-2.48962000	-0.80657700
H	3.45905200	-2.90874300	0.91085200
C	0.04542800	-0.18509400	3.29471800
C	-1.10426900	-0.53904900	4.07067600
C	-2.42871300	-0.51749000	3.55718900
C	-0.94050000	-0.95660400	5.42380600
C	-3.51611400	-0.88453900	4.33807900

H	-2.59448600	-0.19068700	2.54297000
C	-2.02183700	-1.32808100	6.20910000
H	0.05167100	-0.98709200	5.85669800
C	-3.29995500	-1.28917600	5.65369700
H	-4.51838200	-0.85050900	3.92752500
H	-1.87065800	-1.64718500	7.23343000
Br	-4.83998500	-1.81823600	6.75908600
C	1.27600800	0.25395600	3.95463900
O	1.29824400	1.00029500	4.92647500
O	2.41842800	-0.21111000	3.39264600
C	3.60758000	0.38758400	3.87793800
H	3.75210000	0.18271600	4.94126100
H	3.58927700	1.46893300	3.72053100
C	4.78112200	-0.20497500	3.10080200
Cl	6.28276700	0.55461900	3.74190800
Cl	4.86477400	-1.98428900	3.34376100
Cl	4.64461400	0.15068900	1.34542600

(Carbene)–$Co_2(AcO)_4$, ^1A (closed shell)

52

Co	0.00890700	0.07729500	-1.08627400
O	-1.43535100	1.33173000	-1.03030700
O	1.27264100	1.51045900	-1.05380900
O	1.44412700	-1.17420500	-1.19236100
O	-1.25288500	-1.35751000	-1.17908500
Co	0.03484300	-0.04258600	1.18954300
C	-1.56097700	-1.93148500	-0.09282500
O	-1.12357100	-1.58706800	1.05337700
C	1.95415800	-1.57448200	-0.10124300
O	1.55327300	-1.22324000	1.05374500
C	-1.92686100	1.60697000	0.10377000
O	-1.50236800	1.13215000	1.20808300
O	1.20710300	1.48557300	1.19249000
C	1.62652000	1.94511500	0.08426100
C	-3.07743900	2.57850900	0.17606400
H	-3.52278000	2.72350100	-0.80806800
H	-3.82647200	2.21697100	0.88380400
H	-2.70567600	3.53954300	0.54607400
C	-2.48962600	-3.11816900	-0.14015200
H	-2.99588500	-3.17384800	-1.10372900
H	-1.90416800	-4.03122100	0.00918700
H	-3.21817600	-3.05374300	0.67091100
C	2.60812000	3.08906500	0.12916200
H	2.15794500	3.96991000	-0.33792600
H	2.88724000	3.31968300	1.15659500
H	3.49753700	2.82718500	-0.45031900
C	3.13240200	-2.51156000	-0.16974400
H	3.05820700	-3.26863500	0.61229900
H	3.19253600	-2.97870400	-1.15285300
H	4.04615600	-1.93771000	0.01181600
C	0.06702700	-0.12226000	3.19999300
C	-0.98998800	-0.46710300	4.07137200
C	-2.30435600	-0.66653400	3.55619100
C	-0.78257600	-0.62943100	5.47591900
C	-3.35090500	-1.01112100	4.39409100
H	-2.47082700	-0.52652400	2.49919100
C	-1.81801900	-0.99038800	6.31477800
H	0.20636000	-0.48032400	5.89474200
C	-3.09019800	-1.17392500	5.75696200
H	-4.35103900	-1.15261400	4.00403100
H	-1.65662300	-1.12355600	7.37701600
Br	-4.55656500	-1.67098500	6.93618700
C	1.32004400	0.33125700	3.81223400
O	1.45781900	1.49609700	4.13443200
O	2.25659800	-0.63478800	3.95421300
C	3.50923200	-0.23542300	4.51008200
H	3.68483900	-0.82905900	5.40822000
H	3.49640700	0.82722000	4.75233600
C	4.64232500	-0.50778200	3.51688000
Cl	6.17410300	-0.00388200	4.32503800
Cl	4.73040200	-2.25852400	3.12534800
Cl	4.41273100	0.43982500	2.01662100

TS(N_2-ext.)_$Co_2(AcO)_4$, trans, ^3A

54

Co	1.98614300	10.75211300	-10.11947500
O	-0.40172500	11.06722800	-11.91398400
O	-0.77399500	12.03538600	-9.34549700
O	-0.12915000	8.51025700	-10.79588100
O	1.81179400	11.51628600	-11.86401600
O	-0.05257900	9.57805100	-8.25998000
O	1.44328400	12.40657600	-9.33417500
O	2.16672200	9.99669800	-8.37637000
C	0.66785400	11.51335500	-12.42696700
C	1.07095800	8.22649300	-11.07651200
C	0.23809600	12.75334300	-9.10274300
C	1.11690900	9.57805100	-7.77989600
Co	-0.51077000	10.26283800	-10.07085300
O	2.05999300	9.01024300	-10.89799600
C	-2.65155200	9.86329000	-10.12079000
C	-3.17403300	9.83305000	-8.74607700
C	-3.19567900	8.92049600	-11.10163400
N	-3.19740400	11.50728700	-10.74537900
O	-3.65151600	11.01495000	-8.27642700
O	-3.09994700	8.81279100	-8.08520500
C	-4.27941900	8.06824100	-10.80688300
C	-2.59937400	8.84619900	-12.37728900
N	-3.03543100	12.42364000	-11.34860500
C	-4.09029500	11.04166800	-6.92332400
C	-4.73668900	7.14622500	-11.74200700
H	-4.75029800	8.10482200	-9.83194800
C	-3.06741000	7.95140400	-13.33204500
H	-1.77066800	9.50394600	-12.60815200
H	-4.00223500	10.05197300	-6.47420100
H	-5.12890400	11.37554900	-6.91286800
C	-3.25298300	12.03654700	-6.11300700
C	-4.12373800	7.10858300	-12.99263900
H	-5.55894700	6.48205700	-11.50593800
H	-2.61070000	7.90160500	-14.31296400
Cl	-1.54321500	11.51973500	-6.05141600
Cl	-3.36369900	13.68127300	-6.83164400
Cl	-3.94298100	12.06754100	-4.44740900
Br	-4.77231500	5.81936400	-14.32453500
C	1.30100400	9.07253100	-6.36848000
H	0.44434800	8.47329500	-6.06071100
H	1.39012000	9.93358100	-5.69732600
H	2.22277400	8.49292300	-6.28997600
C	1.36284400	6.85790100	-11.63622900
H	1.60109600	6.18470300	-10.80576500
H	2.22445100	6.89405700	-12.30419500
H	0.48758000	6.46820700	-12.15714400
C	0.58844800	12.06164500	-13.83164700
H	-0.32926900	12.64017800	-13.95363100
H	0.54995900	11.22067000	-14.53221500

H	1.45984000	12.67399300	-14.06332700
C	0.01404400	14.09990500	-8.46541500
H	-0.93623200	14.51962200	-8.79683700
H	0.83752700	14.77708000	-8.69350900
H	-0.04041400	13.96246700	-7.38076400

TS(N_2-ext.)_Co_2(AcO)$_4$, cis, ^{3}A

54

Co	2.08983000	10.72656900	-10.51100500
O	-0.51705800	11.08629300	-11.97260100
O	-0.52212700	12.07302500	-9.37811600
O	-0.12315400	8.53255000	-10.88787300
O	1.70116500	11.41479900	-12.25389900
O	0.27348600	9.63151200	-8.36762800
O	1.66747300	12.42164500	-9.74686200
O	2.46229600	10.03068800	-8.76908100
C	0.48947700	11.45640000	-12.64858000
C	1.03400700	8.20229100	-11.27776100
C	0.52011500	12.78738200	-9.32845700
C	1.49723600	9.62249400	-8.04152300
Co	-0.38610200	10.29581700	-10.13180200
O	2.06104900	8.95457200	-11.21367600
C	-2.49876000	9.88675400	-9.88100200
C	-2.71217200	9.83485800	-8.42223600
C	-3.23274700	8.99960200	-10.77523500
N	-3.18264200	11.56927300	-10.23164500
O	-2.39329600	8.56531800	-8.02668300
O	-2.99037500	10.73863500	-7.66324500
C	-4.31049600	8.20615500	-10.32666500
C	-2.83062000	8.90737300	-12.12400100
N	-3.16088700	12.51999400	-10.80200600
C	-2.16547100	8.35517700	-6.64280300
C	-4.94870200	7.32089000	-11.18663800
H	-4.63937500	8.26615200	-9.29562000
C	-3.47590200	8.04312300	-13.00023300
H	-2.00830100	9.52299700	-12.46733500
H	-2.26836700	9.28941700	-6.09025100
H	-1.16186900	7.94574400	-6.52339700
C	-3.18482300	7.34422000	-6.11064800
C	-4.51922100	7.25850600	-12.51169300
H	-5.76589900	6.70191200	-10.83759500
H	-3.16771700	7.97279800	-14.03616300
Cl	-4.85538900	7.98712000	-6.28688600
Cl	-3.05649700	5.79146600	-7.00828300
Cl	-2.82081700	7.06935200	-4.37301700
Br	-5.42015600	6.01746800	-13.73575200
C	1.84850200	9.05558400	-6.68622300
H	1.76479400	7.96463200	-6.73037600
H	1.13856900	9.41540900	-5.93857100
H	2.86632100	9.32144600	-6.40154300
C	1.22449900	6.81273700	-11.82917300
H	1.51342400	6.14847100	-11.00762000
H	2.02130700	6.80191100	-12.57392600
H	0.29133100	6.44571700	-12.25782300
C	0.23630200	11.96456400	-14.04778800
H	-0.63661400	12.62052200	-14.05197300
H	0.01377700	11.10920100	-14.69438000
H	1.10808500	12.48827900	-14.43943300
C	0.41867700	14.15759000	-8.70929700
H	0.80821800	14.10936200	-7.68699900
H	-0.61980800	14.48672200	-8.67620400
H	1.02907300	14.86964900	-9.26769100

TS(N_2-ext.)_Co_2(AcO)$_4$, trans, ^{1}A (open_shell)

54

Co	1.99847500	10.75439800	-10.12064500
O	-0.40366500	11.06138300	-11.90304800
O	-0.75678300	12.04371200	-9.34536500
O	-0.11022600	8.51106700	-10.79340800
O	1.80725700	11.52647100	-11.85787000
O	-0.07113700	9.58061200	-8.26492000
O	1.46026100	12.41817200	-9.32050300
O	2.15162800	9.98200200	-8.38523700
C	0.66206500	11.51607700	-12.41805100
C	1.08698100	8.22598000	-11.08874100
C	0.25394500	12.76261500	-9.09615400
C	1.09949100	9.57146100	-7.78693100
Co	-0.49770300	10.26630000	-10.06924000
O	2.07891300	9.00682100	-10.92199700
C	-2.65292300	9.86274200	-10.12025300
C	-3.17510200	9.83520200	-8.74615700
C	-3.19500800	8.91750100	-11.09898300
N	-3.19616600	11.50912900	-10.74822300
O	-3.65052500	11.01865500	-8.27747100
O	-3.10115800	8.81610200	-8.08321000
C	-4.28187900	8.06857800	-10.80505700
C	-2.59277900	8.83512500	-12.37147800
N	-3.02407800	12.42506400	-11.34947700
C	-4.08979500	11.04710400	-6.92474400
C	-4.73628500	7.14234500	-11.73726200
H	-4.75728700	8.11135300	-9.83254400
C	-3.05766500	7.93584900	-13.32356900
H	-1.76166400	9.48982400	-12.60173400
H	-3.99954000	10.05859100	-6.47342300
H	-5.12923400	11.37856700	-6.91499900
C	-3.25529800	12.04576300	-6.11619600
C	-4.11716700	7.09680700	-12.98467700
H	-5.56084600	6.48085800	-11.50169100
H	-2.59617300	7.87965800	-14.30189900
Cl	-1.54466800	11.53224400	-6.04965300
Cl	-3.36728900	13.68805700	-6.84019800
Cl	-3.94803700	12.08098800	-4.45176300
Br	-4.76133900	5.80130400	-14.31249000
C	1.28146600	9.06364000	-6.37656700
H	0.42046500	8.47127400	-6.06773300
H	1.37904700	9.92350800	-5.70508300
H	2.19861300	8.47643600	-6.30054600
C	1.36626900	6.85620300	-11.65361200
H	1.60566400	6.17969800	-10.82615100
H	2.22350600	6.88805800	-12.32736200
H	0.48511200	6.47274800	-12.16928900
C	0.57383700	12.06552900	-13.82126100
H	-0.34718400	12.63975900	-13.93853900
H	0.53657900	11.22535400	-14.52284000
H	1.44138100	12.68243400	-14.05521100
C	0.02176300	14.10844600	-8.45891000
H	-0.92797800	14.52568700	-8.79503400
H	0.84431200	14.78849300	-8.68184900
H	-0.03822800	13.97016400	-7.37465800

TS(N_2-ext.)_Co_2(AcO)$_4$, cis, ^{1}A (open shell)

54

Co	2.10277700	10.73152300	-10.51742200
O	-0.52174300	11.08267900	-11.95493600

O -0.50301800 12.07601000 -9.36971600
O -0.09948700 8.53270700 -10.88929400
O 1.69341100 11.42036600 -12.25306600
O 0.26458400 9.62838300 -8.37107900
O 1.68308600 12.43975300 -9.74698600
O 2.45301300 10.02839300 -8.77624700
C 0.47872000 11.45743000 -12.63853100
C 1.05448900 8.20083700 -11.29011700
C 0.53584500 12.79676800 -9.32358900
C 1.48939000 9.61870000 -8.04748100
Co -0.37054800 10.29630800 -10.12792400
O 2.08317900 8.94967200 -11.23507300
C -2.49721800 9.88485400 -9.88169200
C -2.71480100 9.83724800 -8.42404200
C -3.22984700 8.99609900 -10.77483000
N -3.17341800 11.57074600 -10.24068100
O -2.39876400 8.56772400 -8.02461600
O -2.99302800 10.74295900 -7.66723900
C -4.31323500 8.20910800 -10.32780900
C -2.82037900 8.89393500 -12.12077000
N -3.13690200 12.51857100 -10.81534700
C -2.17559300 8.36034400 -6.63969800
C -4.94987500 7.32111400 -11.18598300
H -4.64775400 8.27669900 -9.29902700
C -3.46361900 8.02650000 -12.99536400
H -1.99384500 9.50427200 -12.46292100
H -2.27793400 9.29611300 -6.08959500
H -1.17345800 7.94850900 -6.51601900
C -3.19889800 7.35303800 -6.10806500
C -4.51283100 7.24897200 -12.50813400
H -5.77143800 6.70733000 -10.83801800
H -3.14959200 7.94843500 -14.02897900
Cl -4.86742400 7.99947500 -6.29030500
Cl -3.07179800 5.79794900 -7.00184900
Cl -2.84035800 7.08113600 -4.36880000
Br -5.41138000 6.00392300 -13.72974500
C 1.84260300 9.04907400 -6.69434700
H 1.75899700 7.95820500 -6.74059300
H 1.13413100 9.40742200 -5.94462200
H 2.86097300 9.31472700 -6.41151400
C 1.23332400 6.81029400 -11.84465100
H 1.52115100 6.14288400 -11.02524800
H 2.02677100 6.79499800 -12.59287500
H 0.29597300 6.44965200 -12.26968000
C 0.21136500 11.96551900 -14.03459900
H -0.66280900 12.61979000 -14.03017900
H -0.01543000 11.11014100 -14.67966800
H 1.07866600 12.49120600 -14.43345000
C 0.42555200 14.16541500 -8.70109900
H 0.81537900 14.11716500 -7.67889000
H -0.61485400 14.48842200 -8.66685300
H 1.03154900 14.88263300 -9.25766000

[Ru$_2$(AcO)$_4$]Cl, ^{4}A

31
Ru 0.01871300 0.00047200 -1.17114500
O -1.40522700 1.49834600 -1.13176900
O 1.51510000 1.42447900 -1.09258600
O 1.44102200 -1.49764400 -1.09431500
O -1.47875700 -1.42395800 -1.13354200
Ru -0.01422800 -0.00019600 1.15347600
C -1.92802800 -1.83338100 -0.02067000
O -1.49448800 -1.40377900 1.10958000
C 1.83352400 -1.93234400 0.03056800
O 1.38857100 -1.48274600 1.14817700
C -1.83161700 1.93012400 -0.01833700
O -1.41803700 1.48000000 1.11140000
O 1.46726400 1.40340500 1.14989900
C 1.93208400 1.83404700 0.03288300
C 3.01296300 2.87141400 0.08212800
H 2.62654500 3.76562600 0.57937100
H 3.84768900 2.49449500 0.67882900
H 3.35063600 3.12125900 -0.92253400
C 2.86168100 -3.02204200 0.07826700
H 3.72169400 -2.67977600 0.65999200
H 2.43858500 -3.89054900 0.59068700
H 3.17467000 -3.29826300 -0.92749700
C -3.01659100 -2.86367100 -0.00347800
H -3.89385800 -2.44871100 0.50057700
H -3.27634100 -3.16334600 -1.01768900
H -2.68222100 -3.73104800 0.57181600
C -2.86836800 3.01253100 0.00032900
H -2.49515800 3.85899300 0.58274500
H -3.10885800 3.33014000 -1.01314900
H -3.76725000 2.63764800 0.49779100
Cl 0.05384700 0.00153300 -3.63841200

[Ru$_2$(AcO)$_4$]Cl, ^{2}A

31
Ru -0.00258700 -0.01633300 -1.17624100
O -1.46144200 1.43746700 -1.09674700
O 1.49620200 1.42861600 -1.09457800
O 1.38416300 -1.53823800 -1.06517100
O -1.50971400 -1.46025300 -1.16169100
Ru -0.03063500 -0.01763000 1.23027900
C -1.94374700 -1.86693700 -0.04048000
O -1.52193900 -1.43363100 1.08785300
C 1.82010300 -1.94651400 0.05633200
O 1.35704400 -1.52991800 1.17031200
C -1.84508300 1.91923400 0.01517400
O -1.46964300 1.44494000 1.13907900
O 1.45440700 1.40289800 1.15205900
C 1.90750600 1.83311300 0.03549300
C 2.96447800 2.89884700 0.07983900
H 2.48191800 3.85991300 0.28580100
H 3.66740900 2.69425300 0.88919500
H 3.48398800 2.96022700 -0.87590700
C 2.87761100 -3.00932700 0.07549400
H 3.50457600 -2.89931500 0.96099600
H 2.38647000 -3.98714500 0.11932200
H 3.47763700 -2.96191100 -0.83330000
C -3.00725500 -2.92710500 -0.01798200
H -3.82321600 -2.61359800 0.63730100
H -3.37913800 -3.11690300 -1.02396900
H -2.58171800 -3.84626700 0.39595100
C -2.77850100 3.09208500 0.00806500
H -2.17986600 4.00887000 0.03664800
H -3.37416600 3.09547700 -0.90489600
H -3.41895900 3.07061200 0.89029400
Cl 0.06965300 0.01753000 -3.60113100

(Diazo)–[Ru$_2$(AcO)$_4$]Cl, cis ^{4}A

55
Ru 1.79834200 11.08974000 -10.65820400

O -0.55810500 11.46679700 -12.59566200
O -0.74963900 12.68344900 -9.99117500
O -0.22935100 8.86821400 -11.33390700
O 1.66495900 11.76471600 -12.60741600
O -0.42016700 10.12280400 -8.74981800
O 1.46790900 13.01452600 -9.96392500
O 1.80551400 10.39744900 -8.70563700
C 0.53075400 11.82532700 -13.17366400
C 0.96309900 8.44211900 -11.54625600
C 0.28227800 13.41629700 -9.76723100
C 0.71569000 10.06153200 -8.15233900
Ru -0.50219300 10.78214100 -10.68153800
C -3.67833100 8.69873400 -10.63387800
C -3.58997100 9.37328300 -9.34372100
C -3.55850800 9.27150500 -11.99066400
N -3.81154700 7.39088100 -10.51909800
O -3.23896300 10.68911100 -9.50377400
O -3.76762300 8.84708900 -8.26179700
C -4.09220500 10.53664600 -12.28110800
C -2.87963400 8.56939200 -12.99853700
N -3.91668500 6.26529200 -10.42724200
C -3.21826800 11.50081500 -8.33552600
C -3.93091900 11.10102300 -13.54437800
H -4.62549200 11.08997800 -11.51980400
C -2.72799300 9.11545400 -14.27045300
H -2.43870200 7.60179000 -12.78609900
H -3.37250800 10.89127900 -7.44579200
H -2.25526000 12.00732700 -8.30044800
C -3.25040200 10.37973300 -14.51822600
H -4.33782600 12.08180800 -13.75886300
H -2.19617600 8.57050200 -15.04093700
Br -3.00966600 11.17693300 -16.30449600
O 1.99670300 9.15084500 -11.35131200
C -4.32632500 12.55279600 -8.43000700
Cl -5.93820900 11.75750700 -8.50012000
Cl -4.11389800 13.57497800 -9.89247200
Cl -4.21404600 13.57823400 -6.95851900
C 0.43465200 12.36141900 -14.56888900
H 0.06598000 13.39142800 -14.51912400
H -0.28751800 11.77789100 -15.14215700
H 1.41243300 12.35534200 -15.04921500
C 0.04484200 14.81006100 -9.27135300
H -0.73058200 14.79826100 -8.50270100
H -0.31967100 15.41936800 -10.10464100
H 0.96661300 15.24257700 -8.88452000
C 0.73077700 9.55367300 -6.74259400
H 0.48173300 8.48801700 -6.74990900
H -0.03643800 10.07039000 -6.16143600
H 1.71239800 9.69671400 -6.29315700
C 1.11677000 7.03974300 -12.05197000
H 0.58254400 6.94376000 -13.00136700
H 0.65581600 6.34922500 -11.34057400
H 2.16844900 6.79201400 -12.18755400
Cl 4.24392100 11.41490200 -10.63234300

(Diazo)–$[Ru_2(AcO)_4]Cl$, cis ^2A

55
Ru 1.81423400 11.09950700 -10.65935100
O -0.60177400 11.52915600 -12.55034700
O -0.73418300 12.71128500 -9.92113200
O -0.27889800 8.90698300 -11.32069500
O 1.62569900 11.80031800 -12.60077300
O -0.42176500 10.13530300 -8.72405400
O 1.48832300 13.01893200 -9.93257700
O 1.81078500 10.37047500 -8.71363000
C 0.48302600 11.87884100 -13.14369500
C 0.90319400 8.47495300 -11.57671500
C 0.31185700 13.42935200 -9.71022900
C 0.72475200 10.04446100 -8.14965600
Ru -0.51199200 10.81863500 -10.64730400
C -3.64563900 8.69982800 -10.61354300
C -3.59119200 9.40401300 -9.33640900
C -3.51070200 9.23235800 -11.98474800
N -3.76856700 7.39394900 -10.46537100
O -3.25188500 10.72000700 -9.51396200
O -3.78530300 8.89661400 -8.24825200
C -3.99959900 10.50708000 -12.31037300
C -2.86552700 8.47685100 -12.97636600
N -3.86387200 6.27013500 -10.34548300
C -3.24891200 11.55001700 -8.35776000
C -3.82679100 11.02721600 -13.59114800
H -4.50641400 11.10335100 -11.56400200
C -2.70213500 8.97912800 -14.26431600
H -2.46076700 7.49910000 -12.74009000
H -3.41618600 10.95398800 -7.46135400
H -2.28712800 12.05864000 -8.31743000
C -3.17981100 10.25405100 -14.54710000
H -4.19843400 12.01595800 -13.83146500
H -2.19677500 8.39162700 -15.02125500
Br -2.92279700 10.98840500 -16.35824700
O 1.94989600 9.16435000 -11.39163900
C -4.35627900 12.59863200 -8.48570800
Cl -5.96640600 11.80128700 -8.56664800
Cl -4.12200200 13.59652400 -9.96195300
Cl -4.26628300 13.64847400 -7.03011600
C 0.36711900 12.42183200 -14.53474100
H -0.08590200 13.41712900 -14.48248700
H -0.30012900 11.78776200 -15.12192900
H 1.34773700 12.49390700 -15.00316200
C 0.09856600 14.81604900 -9.18424100
H -0.55540400 14.77331300 -8.30982200
H -0.41177600 15.40799900 -9.94997900
H 1.04836400 15.28338700 -8.92881400
C 0.75580900 9.51237400 -6.74888900
H 0.46804600 8.45669300 -6.76777700
H 0.02041800 10.04574600 -6.14196800
H 1.75152700 9.61368600 -6.31968900
C 1.02521400 7.08847600 -12.13252700
H 0.53664900 7.05935100 -13.11113600
H 0.49874200 6.38908700 -11.47836200
H 2.07100500 6.80171600 -12.23189800
Cl 4.25962000 11.39453500 -10.66523700

(Diazo)–$[Ru_2(AcO)_4]Cl$, trans ^4A

55
Ru 1.83475400 10.92454700 10.54181300
O -0.16812000 12.47003300 12.30494700
O -0.58951300 12.49576500 9.44915900
O -0.29287400 9.59455400 12.30246200
O 2.05372200 12.36927600 12.00503600
O -0.72064400 9.59791900 9.46561100
O 1.62787200 12.41058200 9.11758600
O 1.48881400 9.47560600 9.10713800
C 1.02941500 12.84473300 12.58231500

C 0.85154500 9.07601000 12.56037500
C 0.48276500 12.89300900 8.86351400
C 0.30136100 9.09449200 8.87506700
Ru -0.45773500 11.03748000 10.88535500
C -3.69471800 9.24770300 9.90800200
C -3.74054800 10.61432000 9.39994000
C -3.47819800 8.86503100 11.31351100
N -3.60075600 8.31347400 8.98452200
O -3.64045400 10.64606300 8.04439300
O -3.82132900 11.62155100 10.08188900
C -3.70761400 9.78284500 12.35252700
C -2.98221300 7.58988200 11.63442800
N -3.52624100 7.50195100 8.19428100
C -3.43921000 11.93678900 7.47799200
C -3.42461600 9.44290300 13.67447500
H -4.08869100 10.76799100 12.12485600
C -2.70514600 7.23987100 12.95209300
H -2.78813100 6.86025300 10.85549000
H -2.74426700 12.51571700 8.08864800
H -4.38730100 12.47099100 7.38131800
C -2.92216200 8.17939500 13.95384000
H -3.59565900 10.16217500 14.46657400
H -2.31592100 6.25598900 13.18450900
Br -2.48294000 7.70978600 15.81775000
O 1.92119800 9.44474500 11.98423900
C -2.83991700 11.73703300 6.08759400
Cl -1.25267800 10.89843900 6.19922000
Cl -3.94422100 10.77280600 5.05102700
Cl -2.60602300 13.36837400 5.36999200
C 1.20480100 13.89015500 13.64186400
H 0.62485900 14.77685500 13.37269700
H 0.80978500 13.50931500 14.58795700
H 2.25665300 14.14838100 13.75480700
C 0.36467400 13.95731200 7.81494800
H 0.19463500 13.47290600 6.84834900
H -0.48576100 14.60546400 8.02787800
H 1.28844100 14.53323800 7.75777800
C 0.07534700 7.98233600 7.89651500
H -0.93336400 8.03214600 7.48878700
H 0.82002700 8.02370000 7.10091500
H 0.19139700 7.03071700 8.42636100
C 0.89941500 7.97122800 13.57062100
H 0.28989600 8.23585300 14.43666700
H 0.45976800 7.07472400 13.12228400
H 1.92708500 7.76410500 13.86596900
Cl 4.27337800 10.81390400 10.16566100

(Diazo)–[$Ru_2(AcO)_4$]Cl, trans ^{2}A

55
Ru 1.86180400 10.92220800 10.53150100
O -0.17984700 12.47008300 12.28893600
O -0.59395600 12.48944900 9.43420500
O -0.30358900 9.59141300 12.28898100
O 2.04536500 12.36988100 12.00493500
O -0.72145200 9.59717300 9.45051900
O 1.62656800 12.41635400 9.11148400
O 1.48993000 9.47217700 9.09555000
C 1.01762600 12.84285700 12.57423300
C 0.84187400 9.07475700 12.55230200
C 0.48132400 12.89332000 8.85616200
C 0.30384800 9.09178300 8.86484100
Ru -0.45247100 11.03377800 10.87079300
C -3.67822300 9.24926200 9.91567000
C -3.72396900 10.61597700 9.40829300
C -3.46638100 8.86542500 11.32145300
N -3.57990000 8.31593300 8.99175100
O -3.62157300 10.64877700 8.05308000
O -3.80799400 11.62279900 10.09059200
C -3.69432800 9.78347900 12.36051600
C -2.97865500 7.58723500 11.64287400
N -3.50178000 7.50518000 8.20112500
C -3.42527300 11.94113200 7.48860500
C -3.41727200 9.44086400 13.68300300
H -4.06939300 10.77081500 12.13241500
C -2.70776500 7.23447800 12.96104000
H -2.78643500 6.85703500 10.86400500
H -2.72358200 12.51735800 8.09417300
H -4.37394900 12.47618500 7.40275700
C -2.92254500 8.17443400 13.96282600
H -3.58677000 10.16037200 14.47522400
H -2.32519800 6.24807500 13.19389000
Br -2.49112300 7.70076100 15.82776800
O 1.91357700 9.43964500 11.98130700
C -2.84034500 11.74488400 6.09176000
Cl -1.25129700 10.90724500 6.18446700
Cl -3.95460200 10.78259700 5.06406200
Cl -2.61464900 13.37812200 5.37546400
C 1.18532500 13.88716600 13.63619800
H 0.61003500 14.77506700 13.36083200
H 0.77795800 13.50668200 14.57720400
H 2.23630700 14.14317300 13.76138900
C 0.36223800 13.96274400 7.81272400
H 0.16062400 13.48423900 6.84928200
H -0.47202600 14.62511200 8.04587300
H 1.29452000 14.52220800 7.73669700
C 0.07658500 7.97519700 7.89152800
H -0.90748000 8.06668600 7.43316300
H 0.86127500 7.96581500 7.13482200
H 0.11404300 7.02902800 8.44227300
C 0.88506600 7.97380900 13.56703100
H 0.28685000 8.25157600 14.43701900
H 0.42714800 7.08224800 13.12741800
H 1.91212500 7.75346100 13.85460200
Cl 4.29645000 10.81491000 10.16292500

TS(N_2-ext.), [$Ru_2(AcO)_4$]Cl, cis ^{4}A

55
Ru 2.14921500 10.74947000 -10.54112000
O -0.57324600 11.09521500 -11.98186100
O -0.40427200 12.13548200 -9.30316400
O 0.05210800 8.41301000 -10.94798900
O 1.60146600 11.50378500 -12.37838600
O 0.24644200 9.51975600 -8.27839700
O 1.76600800 12.58002600 -9.67144000
O 2.43007700 9.93536200 -8.63344100
C 0.37945600 11.52840100 -12.72071900
C 1.20854100 8.08328900 -11.39162700
C 0.61804900 12.90379600 -9.23748000
C 1.48015800 9.51071600 -7.91626600
Ru -0.19783500 10.26669700 -10.12157100
O 2.21982300 8.84665200 -11.34853000
C -2.36383200 9.89028900 -9.93129700
C -2.64497500 9.87434800 -8.46627400
C -3.09709700 8.97943900 -10.81551700

N -3.00888300 11.51354700 -10.33172400
O -2.42113100 8.60835300 -8.02883200
O -2.91350200 10.81844300 -7.75797300
C -4.23620200 8.28095000 -10.36113700
C -2.66089300 8.77994700 -12.14068500
N -3.03943900 12.43194000 -10.95191300
C -2.33525500 8.40481300 -6.62377400
C -4.89746100 7.38079200 -11.18658700
H -4.60339200 8.42839500 -9.35286400
C -3.32364900 7.89415600 -12.98175400
H -1.80515000 9.32832800 -12.50798500
H -2.40304600 9.35445100 -6.09307400
H -1.38417300 7.91341200 -6.41838200
C -3.47822600 7.49090800 -6.17179600
C -4.42688200 7.20202100 -12.48659000
H -5.76293000 6.83763000 -10.82810400
H -2.98410700 7.74215600 -13.99880300
Cl -5.06677500 8.27486600 -6.48435200
Cl -3.41284100 5.92496100 -7.05069200
Cl -3.27802200 7.20814900 -4.41052300
Br -5.35467600 5.93661600 -13.66036600
C 1.78680100 8.94057900 -6.56114000
H 1.59465900 7.86323600 -6.57974100
H 1.12149300 9.38605200 -5.81778200
H 2.82770900 9.11957000 -6.29538900
C 1.35857200 6.70838200 -11.97380400
H 0.50014900 6.48427100 -12.61025300
H 1.36814800 5.98141800 -11.15518400
H 2.28777900 6.62789800 -12.53659900
C 0.01242500 12.08252500 -14.06597400
H -0.75568500 12.85028700 -13.94414100
H -0.41420800 11.28030000 -14.67549700
H 0.88748500 12.49809800 -14.56353900
C 0.44219500 14.24515800 -8.58871000
H 0.41047300 14.10529900 -7.50318100
H -0.50769200 14.68454300 -8.89860200
H 1.27259600 14.90424900 -8.83958600
Cl 4.70031500 10.96442300 -10.59776900

TS(N_2-ext.), [Ru_2(AcO)$_4$]Cl, cis ^{2}A

55
Ru 2.21197900 10.76006200 -10.55052900
O -0.58690200 11.11173200 -11.99769800
O -0.41361500 12.15159000 -9.32982500
O 0.04128100 8.42167900 -10.96182800
O 1.59434700 11.51511300 -12.37635800
O 0.22527800 9.53923000 -8.29351400
O 1.76868200 12.57883700 -9.66849200
O 2.41930200 9.92777000 -8.63483100
C 0.37769800 11.54335900 -12.72570700
C 1.20152200 8.09167800 -11.40048400
C 0.61814200 12.90881500 -9.25389100
C 1.45859500 9.51821500 -7.92737700
Ru -0.21415600 10.27898700 -10.14436200
O 2.21481900 8.84788000 -11.36092200
C -2.33902200 9.88934400 -9.91823200
C -2.61175800 9.85812800 -8.44913900
C -3.07789200 8.97376600 -10.80377700
N -3.00036100 11.48042000 -10.30048300
O -2.40228800 8.58775800 -8.02210800
O -2.86901600 10.80023900 -7.73406500
C -4.20982900 8.27102500 -10.34179100
C -2.65642200 8.78307100 -12.13382600
N -3.07149100 12.40425800 -10.90933500
C -2.32007200 8.37284000 -6.61855700
C -4.87752100 7.37222700 -11.16463400
H -4.56880600 8.41277000 -9.32996300
C -3.32522300 7.89859200 -12.97241900
H -1.80767700 9.33612800 -12.50981300
H -2.36338400 9.32024400 -6.08137300
H -1.38145800 7.85617600 -6.41779900
C -3.48397200 7.48399900 -6.16946200
C -4.42023000 7.19995200 -12.46958200
H -5.73779400 6.82562500 -10.79887500
H -2.99558900 7.75352500 -13.99376700
Cl -5.05419300 8.30846200 -6.47018400
Cl -3.46026900 5.92381900 -7.06032800
Cl -3.28468000 7.18358100 -4.41091400
Br -5.35598000 5.93524600 -13.63996900
C 1.74778900 8.94837500 -6.56760500
H 1.52509500 7.87688600 -6.57961900
H 1.09285500 9.41713500 -5.82921800
H 2.79242100 9.10057100 -6.29996700
C 1.34534600 6.71399300 -11.97905300
H 0.51097800 6.51349100 -12.65468100
H 1.29623400 5.98578600 -11.16313100
H 2.29631300 6.61084500 -12.49998800
C 0.02116400 12.10342300 -14.07158900
H -0.73324800 12.88475000 -13.94927300
H -0.42101000 11.30870500 -14.67978000
H 0.90303800 12.50350300 -14.56973500
C 0.43678900 14.25762700 -8.62091800
H 0.23028500 14.12066000 -7.55496300
H -0.42866900 14.75458400 -9.06525400
H 1.33179000 14.86573900 -8.74483200
Cl 4.77745400 10.90463700 -10.54406100

(Carbene)- [Ru_2(AcO)$_4$]Cl, ^{4}A

53
Ru 5.76302500 12.63056800 6.99259200
Ru 8.02680200 12.66517300 5.75814100
O 7.05516500 13.85250100 4.36279200
O 8.50512700 14.35953500 6.81698400
O 8.84176000 11.49095100 7.20285900
O 7.40166600 11.00685200 4.71546000
O 5.08250800 13.81090600 5.45642200
O 6.53187100 14.30792000 7.89834700
O 6.87750200 11.42860700 8.31457800
O 5.44694600 10.96756000 5.82957000
C 5.82749800 14.17282100 4.48797100
C 7.66004400 14.83618400 7.64932100
C 8.09271900 11.11401000 8.17865500
C 6.25442100 10.50154800 4.96599900
C 9.72485000 12.77282200 4.75695100
C 10.56007300 11.71067300 4.30398800
C 10.29352100 10.35958800 4.65798200
C 11.70560100 11.98570000 3.50403800
C 11.12799000 9.33794900 4.23554400
H 9.44239800 10.13163000 5.28120100
C 12.53208300 10.96806600 3.06268100
H 11.93236400 13.00625700 3.21657600
C 12.23029800 9.65589300 3.43922300
H 10.92896000 8.31154500 4.51735200
H 13.39554700 11.18271700 2.44576500

Br 13.39927400 8.21728000 2.83517300
C 10.18660400 14.16622900 4.51753700
O 11.02988600 14.54634100 5.49757800
O 9.82106600 14.87274100 3.60272800
C 11.35227000 15.93580500 5.54313300
H 10.48446300 16.50501600 5.88308900
H 11.67242400 16.29467800 4.56387000
C 8.73966300 10.24865500 9.22365500
H 9.25269500 9.41436600 8.73922600
H 9.49272200 10.83845600 9.75503900
H 7.99725300 9.88024200 9.93013500
C 5.21544700 15.01321600 3.40290800
H 5.92470500 15.77769600 3.08117900
H 4.99971600 14.36989600 2.54351800
H 4.28765900 15.46838400 3.74815900
C 8.02258300 16.11370900 8.34966900
H 9.08419500 16.10963800 8.60130100
H 7.84074800 16.94809700 7.66391400
H 7.41192300 16.24894000 9.24180900
C 5.83091800 9.30272700 4.16787800
H 5.44875700 9.64622300 3.20081400
H 6.69028800 8.65645000 3.98268900
H 5.04143500 8.75874600 4.68577700
C 12.49329100 16.10720900 6.54163200
Cl 13.93725200 15.18722300 5.99699900
Cl 12.88146300 17.86005800 6.61193900
Cl 12.00401900 15.53424900 8.17268200
Cl 3.95714700 12.23113400 8.77396900

(Carbene)- [$Ru_2(AcO)_4$]Cl, ^{2}A

53
Ru 5.72633700 12.62737100 7.01606900
Ru 8.00521700 12.67439100 5.74883400
O 7.05053200 13.85212800 4.34725100
O 8.48556800 14.37016000 6.79713100
O 8.83585500 11.50760400 7.18536000
O 7.38263200 11.02097100 4.70621300
O 5.08508400 13.81639100 5.45278100
O 6.52752800 14.31331600 7.91219300
O 6.88602100 11.42450000 8.32262600
O 5.43489700 10.95192400 5.83768000
C 5.81994500 14.17663500 4.48441800
C 7.64521600 14.84166700 7.64567600
C 8.09666000 11.12134200 8.17205100
C 6.23943600 10.50005800 4.97289200
C 9.72022100 12.76958500 4.75273400
C 10.55121500 11.70571800 4.30264700
C 10.27270000 10.35469400 4.64999700
C 11.70508800 11.97548500 3.51164900
C 11.10308300 9.32897000 4.23030900
H 9.41422200 10.13078900 5.26443800
C 12.52808500 10.95401800 3.07399500
H 11.94067000 12.99546900 3.22932500
C 12.21397300 9.64241000 3.44381500
H 10.89473400 8.30282500 4.50618900
H 13.39787100 11.16437500 2.46454100
Br 13.37687100 8.19846500 2.84339800
C 10.18339400 14.16078600 4.51001400
O 11.02246300 14.54414000 5.49249600
O 9.81978800 14.86456500 3.59217600
C 11.34031000 15.93469200 5.53854100
H 10.47118100 16.50019400 5.88124100
H 11.65682700 16.29556200 4.55886800
C 8.76993600 10.25461700 9.19882200
H 9.23495400 9.39955300 8.70144300
H 9.56572400 10.82912100 9.68203200
H 8.05287500 9.91536900 9.94486400
C 5.22330700 15.04952100 3.41684700
H 5.77013300 15.99606700 3.38371200
H 5.34475100 14.56358600 2.44518600
H 4.16869900 15.23535300 3.61529300
C 8.02642900 16.11722900 8.34003200
H 9.07525500 16.07458400 8.63913100
H 7.91423700 16.94437900 7.63119100
H 7.38363900 16.29338000 9.20164600
C 5.85129600 9.27881100 4.19045200
H 5.76136000 9.54782700 3.13391100
H 6.64280300 8.52980300 4.27517800
H 4.90671500 8.87411900 4.55094500
C 12.48296800 16.10909300 6.53458500
Cl 13.92906200 15.19515600 5.98537800
Cl 12.86470400 17.86325100 6.60631900
Cl 11.99956000 15.53206300 8.16598900
Cl 3.93857300 12.19729700 8.81848600

$\{[Ru_2](AcO)_4\}^+$ ^{4}A

30
Ru 0.01555200 -0.00129100 -1.11208500
O -1.38871200 1.48252000 -1.10461100
O 1.49630100 1.40442200 -1.06515300
O 1.42027000 -1.48302900 -1.06472700
O -1.46723100 -1.40687200 -1.10423900
Ru -0.01984800 -0.00024500 1.17675800
C -1.92880300 -1.83801300 0.00614800
O -1.50043900 -1.40599000 1.12987900
C 1.82559100 -1.93645000 0.05911200
O 1.38443300 -1.48402000 1.16938000
C -1.82993900 1.93486000 0.00562600
O -1.42461700 1.48139700 1.12949000
O 1.46288200 1.40530400 1.16897000
C 1.92460800 1.83637300 0.05857200
C 2.97350500 2.90118300 0.07463200
H 2.47430600 3.87563600 0.04148800
H 3.55720900 2.84101400 0.99321400
H 3.61459500 2.81139800 -0.80253700
C 2.82178800 -3.05068800 0.07523500
H 3.40468500 -3.02119000 0.99583100
H 2.27684300 -4.00005800 0.03696100
H 3.46960500 -2.98861800 -0.79944400
C -2.97759500 -2.90292000 -0.00950300
H -3.62387500 -2.80734100 0.86329700
H -3.55592100 -2.84867800 -0.93180600
H -2.47874100 -3.87715000 0.03346600
C -2.82618600 3.04904900 -0.01043400
H -2.28126600 3.99845900 0.02722800
H -3.40947900 3.01920000 -0.93077100
H -3.47361800 2.98726400 0.86454700

$\{[Ru_2](AcO)_4\}^+$ ^{2}A

30
Ru 0.04466900 -0.00487400 -1.09296800
O -1.35453600 1.50106700 -1.09907500
O 1.52888600 1.41883000 -1.05826900
O 1.45305900 -1.50371300 -1.05788100

O -1.43543200 -1.43190000 -1.09837200
Ru 0.00873900 -0.00395700 1.15836800
C -1.91775700 -1.84933600 0.00547300
O -1.46942800 -1.43061800 1.12363500
C 1.84191400 -1.97814600 0.05997400
O 1.41573400 -1.50614400 1.16477000
C -1.81500400 1.94255000 0.00493600
O -1.39181900 1.49879700 1.12331600
O 1.49517600 1.41976000 1.16403700
C 1.94042400 1.87404900 0.05941000
C 2.94861200 2.97716900 0.07538700
H 2.41216000 3.93188600 0.05614900
H 3.54254100 2.93078400 0.98834300
H 3.58486800 2.91892300 -0.80792400
C 2.80193000 -3.12357700 0.07566900
H 3.39324700 -3.10585800 0.99131400
H 2.22660200 -4.05511300 0.04936700
H 3.44429900 -3.08787300 -0.80446600
C -2.99444600 -2.88573500 -0.01103700
H -3.63710600 -2.77344100 0.86247900
H -3.57230600 -2.81286100 -0.93241800
H -2.52296100 -3.87338700 0.02920100
C -2.83314300 3.03648700 -0.01164200
H -2.30762300 3.99666400 0.02444600
H -3.41660300 2.99313400 -0.93140000
H -3.47879400 2.96195700 0.86370400

(Diazo)–[$Ru_2(AcO)_4$]$^+$, cis, ^{4}A

54
Ru 1.96937500 10.82488700 -10.52400400
O -0.53677500 11.27018600 -12.27674200
O -0.65472200 12.08631800 -9.50932600
O 0.08613300 8.55209700 -11.41020600
O 1.64506200 11.76431500 -12.32133700
O -0.07844200 9.38457900 -8.70528400
O 1.53166600 12.56390300 -9.53977500
O 2.11204000 9.84669700 -8.72466000
C 0.48352400 11.80124000 -12.84142600
C 1.29202200 8.25957400 -11.73733500
C 0.33227800 12.85264600 -9.22575600
C 1.08701400 9.33109400 -8.17295400
Ru -0.30195700 10.33139300 -10.49153200
C -2.75435900 9.64076100 -10.54904600
C -3.17115500 10.54598200 -9.44211500
C -3.24308300 9.69817200 -11.96551300
N -2.55999700 8.38267600 -10.07234000
O -3.43682400 11.77634900 -9.92975900
O -3.20011200 10.22959700 -8.27239200
C -4.37674800 10.44933100 -12.30609000
C -2.57553100 8.97520700 -12.96526900
N -2.30810700 7.35096400 -9.71152100
C -3.74289900 12.80165000 -8.98981000
C -4.81014600 10.51427700 -13.62910400
H -4.92707700 10.99348500 -11.55256900
C -3.01031600 9.01994500 -14.28703600
H -1.69891000 8.38914000 -12.72054500
H -3.66955400 12.42173900 -7.97123300
H -3.03762000 13.61668700 -9.15012600
C -4.11756900 9.80110200 -14.60103000
H -5.68112100 11.10500500 -13.88528400
H -2.48458000 8.46344100 -15.05327800
Br -4.72263800 9.89345300 -16.46933100
O 2.26565300 9.04059800 -11.49953000
C -5.16421200 13.31158400 -9.24334400
Cl -6.34889800 11.98036400 -9.01088900
Cl -5.31104000 13.94734300 -10.91863300
Cl -5.47928100 14.62663200 -8.06543300
C 0.28161000 12.50195200 -14.14701700
H -0.16757900 13.48096100 -13.95016700
H -0.41371500 11.93039000 -14.76445800
H 1.23315400 12.64069500 -14.65876100
C 0.05018500 14.13964700 -8.51721700
H -0.64156800 13.96025400 -7.69159300
H -0.43146700 14.82762600 -9.21955500
H 0.97411400 14.58592500 -8.15223900
C 1.22721100 8.65482500 -6.84651400
H 0.66066400 7.72162300 -6.84912500
H 0.79799200 9.30580200 -6.07793800
H 2.27617100 8.47002800 -6.61932100
C 1.53659100 6.95861600 -12.43240300
H 1.06653800 6.99422800 -13.42005900
H 1.06363400 6.15198300 -11.86740500
H 2.60458400 6.77533100 -12.53902700

(Diazo)–[$Ru_2(AcO)_4$]$^+$, cis, ^{2}A

54
Ru 2.05191700 10.76498600 -10.42766000
O -0.48835400 11.27014500 -12.24039300
O -0.65409100 12.04126800 -9.45315900
O 0.06637000 8.51183300 -11.38831300
O 1.70652300 11.72645600 -12.21880800
O -0.16025900 9.31471900 -8.68521500
O 1.54563600 12.48392100 -9.42580100
O 2.04258100 9.73877800 -8.64021800
C 0.56144100 11.79345200 -12.76120600
C 1.27914000 8.21597900 -11.69607400
C 0.34506700 12.78123900 -9.13824800
C 0.99433200 9.23786900 -8.12856800
Ru -0.32068500 10.29313600 -10.46610200
C -2.66242300 9.74097200 -10.59780800
C -3.14492800 10.65926700 -9.51928500
C -3.19021200 9.76601500 -12.00916600
N -2.57263600 8.46592200 -10.09236400
O -3.38605100 11.87900100 -10.04003600
O -3.23873900 10.35661900 -8.35057700
C -4.39488100 10.41635600 -12.30945600
C -2.49389700 9.10755800 -13.03196300
N -2.39411900 7.42526900 -9.72376800
C -3.72811800 12.92281300 -9.13343300
C -4.87243300 10.45266800 -13.61830600
H -4.96879800 10.90587600 -11.53570000
C -2.97041200 9.12238800 -14.34040700
H -1.56985700 8.58927400 -12.81324900
H -3.67548500 12.56916300 -8.10421500
H -3.02916800 13.74259200 -9.29704700
C -4.14902900 9.80777600 -14.61526600
H -5.79775600 10.96813400 -13.84441100
H -2.42317200 8.61635700 -15.12634000
Br -4.81445000 9.85875400 -16.46449200
O 2.25764300 8.97589900 -11.43326600
C -5.14867000 13.41024500 -9.43354200
Cl -6.32747300 12.07576700 -9.18381600
Cl -5.26558600 13.99046700 -11.13056700
Cl -5.50168500 14.75759800 -8.30403700

C 0.40363400 12.52210400 -14.05863700
H -0.13095300 13.45844300 -13.86928100
H -0.20624800 11.92226200 -14.73763300
H 1.37481800 12.74010900 -14.50051100
C 0.06617600 14.06335600 -8.41705400
H -0.67539400 13.89069700 -7.63460300
H -0.35440600 14.77971700 -9.13043000
H 0.98223200 14.47411700 -7.99478300
C 1.08664900 8.53036000 -6.81301700
H 0.63361800 7.54012700 -6.90434700
H 0.50954700 9.09156900 -6.07191400
H 2.12317200 8.44744200 -6.49015800
C 1.52000300 6.92009700 -12.40382100
H 1.03603100 6.95922000 -13.38445000
H 1.05614500 6.10895100 -11.83722000
H 2.58690300 6.73919400 -12.52421500

(Diazo)–$[Ru_2(AcO)_4]^+$, trans, ^{4}A

54
Ru 1.88115500 10.78151400 10.40245200
O -0.61119100 11.55250500 12.04855300
O -0.68918800 12.02701100 9.21643400
O -0.12517700 8.74052600 11.55796700
O 1.59372800 11.93408800 12.07366200
O -0.19918000 9.21247000 8.74861700
O 1.51348700 12.41612600 9.21655100
O 2.00514500 9.59604300 8.72950800
C 0.42850600 12.09473400 12.56317400
C 1.06206900 8.43208600 11.92849500
C 0.32992700 12.71741700 8.85967100
C 0.96595400 9.04852800 8.23948900
Ru -0.40736600 10.38507700 10.39826400
C -2.88211200 9.84238400 10.46511600
C -3.33540200 11.01842000 9.66841800
C -3.14445200 9.65029400 11.91997400
N -2.82946900 8.70921000 9.72567100
O -3.27528600 10.74740900 8.34793800
O -3.66116500 12.08789400 10.13195300
C -3.47595200 10.74416000 12.73348400
C -2.99832800 8.38256700 12.50470000
N -2.72122800 7.77340600 9.11453100
C -3.50071500 11.85969000 7.48173900
C -3.65927600 10.57235500 14.10414900
H -3.58062200 11.72765600 12.30075600
C -3.18070600 8.20294800 13.87254800
H -2.72357300 7.52072300 11.90821000
H -2.88256300 12.70275500 7.79039200
H -4.55420700 12.14533400 7.49320800
C -3.50666500 9.30566100 14.65518500
H -3.91527300 11.42215400 14.72523900
H -3.06666300 7.21970900 14.31209800
Br -3.75343000 9.06489600 16.59171500
O 2.07915000 9.11923500 11.59163100
C -3.11412700 11.42974400 6.06856100
Cl -1.37366000 10.98518800 5.99069700
Cl -4.10497500 10.02906400 5.54397700
Cl -3.42134800 12.83386900 4.99276500
C 0.25017800 12.96380100 13.76717400
H -0.04044000 13.96495600 13.43125900
H -0.55176600 12.56771300 14.39171300
H 1.18232300 13.03624300 14.32678500
C 0.10860100 13.90584300 7.97903900
H -0.18449500 13.55285500 6.98549400
H -0.71020000 14.50900200 8.37750700
H 1.01788900 14.50000500 7.90173600
C 1.09471300 8.16157400 7.04246100
H 0.25732200 8.33427500 6.36431600
H 2.04496300 8.33435100 6.53886100
H 1.04885500 7.11988400 7.37712400
C 1.24263600 7.20671600 12.76647600
H 0.42771900 7.13343600 13.48868600
H 1.19625100 6.32953200 12.11258000
H 2.20855300 7.22598600 13.26969400

(Diazo)–$[Ru_2(AcO)_4]^+$, trans, ^{2}A

54
Ru 1.95304700 10.79148000 10.40571700
O -0.63533400 11.52742500 12.04178600
O -0.69149400 12.01691300 9.20606600
O -0.12814600 8.70810700 11.52565100
O 1.57430200 11.89773400 12.10225100
O -0.19402300 9.20602600 8.72721700
O 1.51150900 12.43342900 9.24518100
O 2.00972100 9.61978200 8.71014900
C 0.40565300 12.05637200 12.57210800
C 1.06469800 8.40474000 11.89146100
C 0.33121000 12.71763700 8.87451700
C 0.97795600 9.06557800 8.22148300
Ru -0.41720500 10.36779900 10.38589100
C -2.78346300 9.88293700 10.48746200
C -3.28614600 11.03682600 9.67606000
C -3.09756100 9.69303400 11.93703100
N -2.77109800 8.73041800 9.75727000
O -3.24716300 10.74180900 8.36169400
O -3.62598200 12.10558900 10.12779400
C -3.45019600 10.79021400 12.73591600
C -2.97102900 8.42783500 12.52972200
N -2.68268500 7.78751500 9.15847300
C -3.51107200 11.83252900 7.47843200
C -3.67366500 10.62442600 14.10141900
H -3.53844500 11.77250600 12.29748500
C -3.19347200 8.25340700 13.89278000
H -2.67863600 7.56367100 11.94551000
H -2.90783900 12.69498800 7.76217400
H -4.57072000 12.09339400 7.50173300
C -3.53949600 9.35959300 14.66128100
H -3.94475000 11.47737600 14.71170500
H -3.09373900 7.27172700 14.33919500
Br -3.84157600 9.12722100 16.59136800
O 2.08019600 9.09250900 11.56670900
C -3.13570200 11.38513500 6.06771300
Cl -1.38352900 10.99621200 5.96759100
Cl -4.09014900 9.94250300 5.59045400
Cl -3.50787800 12.75523200 4.96858400
C 0.21121600 12.90888500 13.78654200
H -0.19122000 13.87651300 13.46894500
H -0.52196000 12.44111900 14.44625900
H 1.15717000 13.06541000 14.30336900
C 0.10751100 13.90970400 7.99784400
H -0.19002600 13.55860500 7.00491700
H -0.71187600 14.50947900 8.40063600
H 1.01493300 14.50641000 7.91889400
C 1.11465800 8.19093000 7.01514800
H 0.31938900 8.42332700 6.30405900

H 2.09395300 8.31708200 6.55586300
H 0.98916200 7.14893300 7.32695100
C 1.24235300 7.17501200 12.72528200
H 0.51397200 7.18335700 13.53929700
H 1.03664100 6.29832200 12.10305600
H 2.25695600 7.11630500 13.11630000

TS(N_2-ext.), $[Ru_2(AcO)_4]^+$, ^{4}A

54
Ru 2.16403800 10.73240300 -10.51172500
O -0.57959600 11.10155900 -12.02197000
O -0.41549000 12.15817600 -9.36462700
O 0.06041700 8.42957100 -10.97712500
O 1.60826500 11.48181700 -12.34032600
O 0.23219800 9.55501000 -8.31329700
O 1.77025000 12.56006300 -9.66918700
O 2.41867200 9.94543500 -8.63484300
C 0.40082500 11.52857200 -12.73093200
C 1.22525800 8.08216700 -11.37851300
C 0.61528500 12.91177700 -9.26909500
C 1.45012300 9.53365400 -7.92057400
Ru -0.20561600 10.29355300 -10.17801500
O 2.24092100 8.84519800 -11.30614600
C -2.31945800 9.87827600 -9.90920700
C -2.57340400 9.82934000 -8.43092500
C -3.07047000 8.97610700 -10.79448000
N -2.98024700 11.46969000 -10.25364500
O -2.37896800 8.55450700 -8.02677000
O -2.81061000 10.77012400 -7.70965100
C -4.21950500 8.30296500 -10.32795000
C -2.65477300 8.77057200 -12.12462400
N -3.13981300 12.39788000 -10.83560700
C -2.32223300 8.30729300 -6.62416900
C -4.90873800 7.41782400 -11.14622200
H -4.57614800 8.45898000 -9.31752200
C -3.34414300 7.89715600 -12.95700400
H -1.79561500 9.30120300 -12.50826300
H -2.33295300 9.24455500 -6.06819100
H -1.40690200 7.74924200 -6.42828700
C -3.52383300 7.45389700 -6.20519600
C -4.45593700 7.22685400 -12.45081500
H -5.78197400 6.89456700 -10.77726300
H -3.01881000 7.73957400 -13.97778100
Cl -5.05748800 8.34882200 -6.49172500
Cl -3.55577300 5.91927000 -7.13752800
Cl -3.34499200 7.10228500 -4.45513000
Br -5.41975600 5.98108100 -13.61249900
C 1.73745200 8.97726000 -6.55944500
H 1.56463500 7.89659700 -6.57859400
H 1.04824400 9.41434000 -5.83360800
H 2.76944500 9.17449800 -6.27294800
C 1.38961200 6.70277800 -11.93844000
H 0.54173700 6.46611500 -12.58393900
H 1.38975300 5.98881900 -11.10824600
H 2.32821100 6.61827100 -12.48468100
C 0.08849500 12.09274400 -14.08226500
H -0.68770800 12.85565000 -13.98537800
H -0.30902100 11.29279600 -14.71422900
H 0.98198400 12.51471500 -14.53971600
C 0.44624100 14.25664300 -8.63213300
H 0.38931400 14.12124100 -7.54700600
H -0.49044000 14.70757700 -8.96424900
H 1.29184400 14.90157400 -8.86809200

TS(N_2-ext.), $[Ru_2(AcO)_4]^+$, ^{2}A

54
Ru 2.22611900 10.74737000 -10.52158200
O -0.59197600 11.10871600 -12.01708000
O -0.42383900 12.16118500 -9.36323000
O 0.05454500 8.42826400 -10.96885800
O 1.60114500 11.47512100 -12.34510300
O 0.23170300 9.56362800 -8.30812200
O 1.76677000 12.56173000 -9.66581900
O 2.42376100 9.94527300 -8.63786000
C 0.39464000 11.52497000 -12.72793900
C 1.22362800 8.08657300 -11.36987700
C 0.61269500 12.91073400 -9.27071500
C 1.45471400 9.53847100 -7.92585800
Ru -0.21999200 10.29404600 -10.17547600
O 2.23840000 8.84610000 -11.30389400
C -2.30679000 9.88658100 -9.90387500
C -2.55993000 9.82550200 -8.42377900
C -3.05988700 8.97926300 -10.79187300
N -2.97124700 11.45132900 -10.24056900
O -2.36782900 8.54915300 -8.02654100
O -2.79837800 10.76301600 -7.69862700
C -4.20873600 8.30925900 -10.32366900
C -2.64793900 8.77405600 -12.12209300
N -3.14602400 12.38325000 -10.81234300
C -2.31625800 8.29358000 -6.62529700
C -4.90275500 7.42639800 -11.14135600
H -4.56389800 8.46535300 -9.31278000
C -3.34129400 7.90240900 -12.95391700
H -1.78883700 9.30273300 -12.50809400
H -2.32315700 9.22761000 -6.06388200
H -1.40437400 7.72956000 -6.43048900
C -3.52335000 7.44414400 -6.21376600
C -4.45353800 7.23470800 -12.44655000
H -5.77674100 6.90581000 -10.77035800
H -3.01796400 7.74521800 -13.97542300
Cl -5.05173300 8.34984900 -6.49490700
Cl -3.56337900 5.91659400 -7.15734400
Cl -3.34848300 7.07871000 -4.46609600
Br -5.42365900 5.99173400 -13.60802500
C 1.74239600 8.97777900 -6.56585200
H 1.54411400 7.90147500 -6.58179500
H 1.06796100 9.43101600 -5.83583800
H 2.78048900 9.15270900 -6.28722000
C 1.38472400 6.70593800 -11.92917600
H 0.56980900 6.49896700 -12.62606300
H 1.31092400 5.98706000 -11.10680800
H 2.35045700 6.59762900 -12.42080100
C 0.07908400 12.08627600 -14.08022000
H -0.66048600 12.88408800 -13.97472700
H -0.36893800 11.29861000 -14.69319000
H 0.97946000 12.46492300 -14.56133900
C 0.43608600 14.26199900 -8.64797500
H 0.19276700 14.13063500 -7.58915200
H -0.40681000 14.77126500 -9.12089300
H 1.34463500 14.85427200 -8.74568100

(Carbene)-{$[Ru_2](AcO)_4\}^+$ ^{4}A

52
Ru 5.78160100 12.58431600 7.05100500

Ru 8.00090400 12.67858700 5.72519300
O 7.05362100 13.87480500 4.35799000
O 8.49787900 14.34675400 6.81176000
O 8.82756700 11.47521600 7.16367300
O 7.36948000 11.03700700 4.67296100
O 5.13921900 13.79879200 5.53670800
O 6.57462900 14.22810100 7.97067800
O 6.88508000 11.38265400 8.29436700
O 5.44826700 10.96035400 5.84141200
C 5.82910200 14.19009500 4.54100300
C 7.68622000 14.78396100 7.69694300
C 8.10813300 11.07176600 8.14276800
C 6.23256000 10.51576100 4.94492600
C 9.72763200 12.75845800 4.76358200
C 10.57372700 11.69709200 4.35507100
C 10.27504000 10.34158100 4.67547500
C 11.77075400 11.97244500 3.62846600
C 11.12492400 9.31998500 4.29227800
H 9.38169400 10.11117800 5.23490100
C 12.61636000 10.95460300 3.23230900
H 12.02474000 12.99384300 3.36921300
C 12.28052000 9.63901000 3.57237500
H 10.89994100 8.29128300 4.54409600
H 13.51960200 11.16869900 2.67520200
Br 13.46937000 8.20229200 3.02608700
C 10.17226500 14.15320900 4.48133000
O 10.98634900 14.58704200 5.45871900
O 9.80963700 14.80511000 3.52702200
C 11.31847800 15.97722500 5.43762500
H 10.47191100 16.56162600 5.80419300
H 11.58497700 16.29760100 4.42971700
C 8.76203700 10.19232600 9.16551100
H 9.26671500 9.36506900 8.66081700
H 9.52397500 10.77386100 9.69327600
H 8.02877800 9.81507200 9.87666100
C 5.17339700 15.06220800 3.51274200
H 5.79806600 15.94000300 3.33164900
H 5.10311200 14.50553900 2.57334100
H 4.17845000 15.36191400 3.83834100
C 8.06063900 16.03700500 8.42919000
H 9.13849300 16.06105200 8.59498400
H 7.79415700 16.89422900 7.80153800
H 7.51795100 16.10786200 9.37134800
C 5.79980300 9.32817600 4.13905900
H 5.44491400 9.68053100 3.16498200
H 6.65172100 8.66770900 3.96863100
H 4.99236000 8.79718100 4.64193900
C 12.51241900 16.17379600 6.36709700
Cl 13.91644900 15.22017000 5.77989100
Cl 12.91528900 17.92277500 6.35455100
Cl 12.10173500 15.66203900 8.04083100

(Carbene)-{[Ru_2]$(AcO)_4$}$^+$ ^{2}A

52
Ru 5.75734300 12.58754300 7.07482000
Ru 7.99299100 12.68421700 5.71352700
O 7.02624800 13.85466500 4.34074600
O 8.48740400 14.37048200 6.77018800
O 8.84214300 11.50624600 7.15572600
O 7.36575400 11.03207000 4.68070300
O 5.11240400 13.77445700 5.52587400
O 6.56872100 14.26595300 7.94397000
O 6.91791500 11.42303700 8.32320800
O 5.45848800 10.93543700 5.87520900
C 5.79615200 14.15986500 4.52870000
C 7.67082800 14.82050000 7.65066300
C 8.13540300 11.11692300 8.15484000
C 6.23627200 10.49765300 4.97599000
C 9.71334100 12.76976600 4.74456000
C 10.55067300 11.71024400 4.31451600
C 10.25819200 10.35328500 4.63455600
C 11.73394500 11.98885700 3.56692900
C 11.10082700 9.33346100 4.23117300
H 9.37714500 10.12046200 5.21208300
C 12.57062400 10.97255100 3.14852600
H 11.98294700 13.01134100 3.30719400
C 12.24166900 9.65544100 3.48949900
H 10.88147600 8.30390500 4.48448700
H 13.46248900 11.18908000 2.57430500
Br 13.41993500 8.22127500 2.91431200
C 10.17104600 14.16824900 4.50039500
O 11.03317000 14.54150400 5.46201600
O 9.78273400 14.87217900 3.59480500
C 11.37552200 15.92896500 5.49773800
H 10.51346800 16.51288600 5.82651300
H 11.70726900 16.27261000 4.51693500
C 8.81595200 10.25690000 9.17691200
H 9.29402300 9.41184000 8.67538500
H 9.60268200 10.84240200 9.66196000
H 8.10401600 9.90489800 9.92181400
C 5.14033800 15.03049100 3.49898000
H 5.68151600 15.97884000 3.43929400
H 5.21414300 14.54562700 2.52191700
H 4.09669200 15.20982700 3.75261300
C 8.04915900 16.09095700 8.35035500
H 9.10768900 16.06282800 8.61456800
H 7.90032400 16.92421400 7.65553000
H 7.43060700 16.24410400 9.23366400
C 5.82871700 9.28601600 4.19274900
H 5.68883200 9.57316700 3.14636100
H 6.63311700 8.54704600 4.22853400
H 4.90592800 8.86340300 4.58711300
C 12.51287300 16.08956400 6.50231800
Cl 13.94401200 15.14224200 5.97388700
Cl 12.92605400 17.83538600 6.56315800
Cl 12.00142700 15.53411100 8.13361400

[Rh_2]$(AcO)_4$ ^{1}A

30
Rh 5.89983800 12.57941900 6.93838700
Rh 7.95706300 12.72298600 5.72374600
O 6.99563000 13.95943700 4.37867200
O 8.44957600 14.36279800 6.87632600
O 8.80860200 11.47970200 7.13376000
O 7.35626700 11.07424100 4.63738700
O 5.04807800 13.82164100 5.52821100
O 6.50039300 14.22838100 8.02339700
O 6.86186000 11.34408500 8.28484200
O 5.40821500 10.93869500 5.78610000
C 5.76564700 14.23647300 4.56074500
C 7.63229300 14.75576900 7.77086700
C 8.08803600 11.05587500 8.09524800
C 6.23069900 10.53837200 4.89936400
C 8.73041700 10.10655100 9.07392900

H 8.56546500 9.08150400 8.72524100
H 9.80498500 10.28396100 9.12786200
H 8.27333700 10.21105400 10.05870600
C 5.09679600 15.10300700 3.52464300
H 5.81193400 15.81379800 3.10828800
H 4.73835900 14.46238100 2.71191600
H 4.24296700 15.62537900 3.95696400
C 8.05176700 15.92380600 8.62609300
H 8.52024100 15.53854000 9.53796700
H 8.77707900 16.54313400 8.09784000
H 7.18017600 16.51329300 8.91406800
C 5.85249200 9.30700900 4.11677800
H 6.35884300 9.29798500 3.15121300
H 6.16574100 8.42332100 4.68280900
H 4.77070600 9.26096200 3.98407200

(Diazo)–[$Rh_2(AcO)_4$], trans

54
Rh 1.88743300 10.78590900 10.38951600
O -0.62689800 11.56018800 12.05039100
O -0.69963100 12.03307500 9.18303700
O -0.12422000 8.71699800 11.57027100
O 1.60254200 11.96760300 12.06000000
O -0.19465200 9.17131100 8.72557400
O 1.53285800 12.41306400 9.16617900
O 2.03289900 9.57989600 8.71494800
C 0.42470000 12.09925100 12.52504800
C 1.07081200 8.45880300 11.93090200
C 0.33763300 12.68289800 8.82713100
C 0.98046200 9.03743500 8.25059600
Rh -0.48016900 10.35853800 10.38278500
C -2.99565400 9.82430900 10.47445200
C -3.38460700 11.02132600 9.69716700
C -3.21058400 9.62653600 11.92977000
N -2.88380200 8.71361400 9.72871500
O -3.31668600 10.77579000 8.36680700
O -3.69934700 12.09422200 10.16805400
C -3.53903800 10.71083500 12.75771300
C -3.03616900 8.35848200 12.50658100
N -2.74546500 7.78104300 9.11177700
C -3.43521900 11.92641500 7.53500200
C -3.69288800 10.53001900 14.13115700
H -3.66261100 11.69455700 12.33037800
C -3.18774300 8.16880300 13.87708000
H -2.75883900 7.50541600 11.89891000
H -2.72186100 12.68548500 7.85567400
H -4.45136500 12.32455800 7.56547800
C -3.51351100 9.26261900 14.67129600
H -3.94611000 11.37328200 14.76242500
H -3.04941100 7.18527900 14.30926400
Br -3.72152400 9.00701900 16.61527600
O 2.10257000 9.13070800 11.61233800
C -3.11141100 11.49987800 6.10575400
Cl -1.43662900 10.87216000 5.98469500
Cl -4.26082900 10.23349000 5.55029900
Cl -3.28209900 12.96400900 5.07305100
C 0.24577500 12.99364200 13.72541300
H 0.04832600 14.01282400 13.37655100
H -0.60813300 12.66186200 14.31745600
H 1.15301200 13.00579100 14.33045900
C 0.11988200 13.85500100 7.90470300
H -0.09096600 13.47457400 6.90029300
H -0.74572800 14.43367500 8.23404000
H 1.00640800 14.48764500 7.86897000
C 1.12881400 8.13393400 7.05337000
H 0.25101900 8.21449300 6.41038300
H 2.03510600 8.37897000 6.49927500
H 1.20014500 7.09844000 7.40260100
C 1.26870300 7.23220400 12.78425600
H 0.40547600 7.08581200 13.43528800
H 1.35471500 6.35922100 12.12862400
H 2.18343100 7.31811200 13.37122500

(Diazo)–[$Rh_2(AcO)_4$], cis

54
Rh 1.96870900 10.82874200 -10.49967600
O -0.56087400 11.25746300 -12.26402900
O -0.68834000 12.06495900 -9.46092000
O 0.09885000 8.51231400 -11.41792700
O 1.64575300 11.77019200 -12.31328900
O -0.06186200 9.32453000 -8.66212000
O 1.51804700 12.57623900 -9.50163500
O 2.14422600 9.84395500 -8.68829000
C 0.46948700 11.78143900 -12.79915500
C 1.31280100 8.28179800 -11.74021600
C 0.30682400 12.81736100 -9.19971400
C 1.11241900 9.31519700 -8.16483500
Rh -0.37464800 10.27908200 -10.45970500
C -2.87010000 9.59364200 -10.53325200
C -3.22385400 10.53534500 -9.45111300
C -3.30944300 9.66186000 -11.95827600
N -2.62097800 8.36177400 -10.05529500
O -3.47849200 11.76095700 -9.97074200
O -3.24452200 10.26231700 -8.26799700
C -4.44937900 10.39289600 -12.32193000
C -2.59218400 8.97351200 -12.94751400
N -2.34196600 7.33686400 -9.68193300
C -3.70199900 12.82121600 -9.05406700
C -4.84689600 10.46742200 -13.65594900
H -5.02928500 10.91602200 -11.57415300
C -2.99197700 9.02480700 -14.28075000
H -1.69857800 8.42195200 -12.68101700
H -3.61056400 12.46759800 -8.02725600
H -2.96693600 13.59926800 -9.25831800
C -4.11004900 9.78134700 -14.61453500
H -5.72267300 11.04243600 -13.93111900
H -2.42888800 8.49665400 -15.04069300
Br -4.66682200 9.88335000 -16.50234300
O 2.29692100 9.04567900 -11.49619200
C -5.10705500 13.38831500 -9.27153700
Cl -6.34474400 12.12376500 -8.95343200
Cl -5.29110200 13.97950600 -10.96026100
Cl -5.32089600 14.75500500 -8.12656300
C 0.25951800 12.49664900 -14.10946800
H -0.10578000 13.50809000 -13.90288400
H -0.49735900 11.97734600 -14.69976200
H 1.19633500 12.56856900 -14.66230800
C 0.00789500 14.11587700 -8.49420400
H -0.73447400 13.95261000 -7.71049900
H -0.41318400 14.82168900 -9.21776200
H 0.91768300 14.54268600 -8.07261000
C 1.27733200 8.64494300 -6.82493400
H 0.58091800 7.81045700 -6.73200600
H 1.04577600 9.37249800 -6.03980100

H 2.30487600 8.30553800 -6.69172700
C 1.57792000 7.00291100 -12.49115100
H 1.19703700 7.10948100 -13.51196100
H 1.03984000 6.17807300 -12.01923300
H 2.64594900 6.79010000 -12.52629400

TS(N_2-ext.), [Rh_2(AcO)$_4$], cis ^{1}A

54
Rh 2.09871800 10.70389200 -10.48407200
O -0.58687600 11.13229000 -12.03716400
O -0.41458100 12.21105300 -9.36403600
O 0.01692000 8.43325700 -10.98076900
O 1.63177800 11.47104500 -12.35357700
O 0.19428400 9.57134100 -8.28337400
O 1.80515100 12.57987300 -9.65133500
O 2.41317100 9.92032900 -8.59551700
C 0.42173200 11.52179600 -12.72253100
C 1.18400700 8.09271900 -11.36921800
C 0.64299700 12.92194400 -9.27501600
C 1.41568900 9.53447100 -7.91422600
Rh -0.29033000 10.31122700 -10.16234700
O 2.23167900 8.80594000 -11.30236300
C -2.38437800 9.89587300 -9.90198700
C -2.64338600 9.85433200 -8.43634900
C -3.11229300 8.98608400 -10.79697900
N -3.04955800 11.50643200 -10.27501800
O -2.41400500 8.58089200 -8.02149400
O -2.90210900 10.78567700 -7.70649500
C -4.22631300 8.24892300 -10.34525100
C -2.68729600 8.82996000 -12.13070400
N -3.07492800 12.44242800 -10.86992800
C -2.28911300 8.36130100 -6.62356900
C -4.87620200 7.35202900 -11.18468200
H -4.58253200 8.36173900 -9.32858100
C -3.34024900 7.94919900 -12.98588600
H -1.84685200 9.40728200 -12.49076300
H -2.37301300 9.30106600 -6.07774900
H -1.32007300 7.89584300 -6.44324600
C -3.39510800 7.40904800 -6.16065900
C -4.41888200 7.21808500 -12.49416100
H -5.72277200 6.77806600 -10.82891200
H -3.01044100 7.83034900 -14.01063200
Cl -5.01182200 8.14971900 -6.43167600
Cl -3.30217900 5.85567000 -7.06000400
Cl -3.15176400 7.10924500 -4.40695500
Br -5.33198700 5.95716600 -13.68909100
C 1.67803300 8.95367200 -6.54613900
H 1.51225500 7.87213900 -6.58252800
H 0.97696200 9.37581300 -5.82270200
H 2.70424800 9.14800200 -6.23540000
C 1.31502700 6.69971200 -11.93440600
H 0.44092600 6.46014900 -12.54277800
H 1.35385700 5.98579000 -11.10506200
H 2.22833500 6.60807200 -12.52229600
C 0.11583500 12.07110400 -14.09316300
H -0.68157000 12.81459400 -14.02279700
H -0.24482800 11.25708800 -14.72963800
H 1.00642200 12.51308100 -14.53879500
C 0.48249200 14.27817500 -8.63398200
H 0.48411500 14.15377500 -7.54597700
H -0.47313900 14.72002600 -8.92114600
H 1.30760200 14.93335500 -8.91379200

TS(N_2-ext.), [Rh_2(AcO)$_4$], trans ^{1}A

54
Rh 2.06445100 10.75089800 -10.54666700
O -0.61038600 11.08194100 -12.13999700
O -0.52312000 12.14057800 -9.45776900
O 0.08594000 8.39569000 -11.09723400
O 1.59658900 11.51968800 -12.41532700
O 0.16703900 9.51663200 -8.39154200
O 1.68434000 12.60324700 -9.69881300
O 2.37501200 9.96197700 -8.65863800
C 0.39112800 11.52326400 -12.80329100
C 1.27229000 8.11031600 -11.47145000
C 0.50183100 12.89308900 -9.34220000
C 1.38151800 9.52874700 -7.99917400
Rh -0.30981600 10.25100400 -10.26749600
O 2.28623700 8.86821000 -11.38267600
C -2.39021600 9.73233500 -10.03148500
C -2.64322800 9.57120800 -8.57789800
C -3.07681000 8.81961400 -10.96003600
N -3.10611800 11.31912000 -10.39568600
O -2.78666400 10.74875800 -7.92354900
O -2.59380400 8.48375000 -8.04111600
C -4.17087100 8.03207200 -10.54789700
C -2.63607500 8.72852600 -12.29409400
N -3.17093200 12.27044400 -10.96293200
C -2.86093100 10.69816700 -6.50597200
C -4.78953300 7.15443700 -11.43078500
H -4.53159300 8.09151600 -9.52872100
C -3.25677200 7.86597300 -13.19139300
H -1.81005800 9.34387300 -12.62333900
H -2.13224900 11.40552100 -6.10819100
H -2.65613000 9.68937100 -6.14680800
C -4.26139700 11.12204200 -6.05073200
C -4.31953500 7.08761400 -12.74082800
H -5.62348700 6.54377300 -11.10764500
H -2.91455900 7.79883800 -14.21678600
Cl -4.63151800 12.78287300 -6.63026200
Cl -5.49676700 9.98480600 -6.69319400
Cl -4.27947200 11.08986300 -4.25536700
Br -5.19102200 5.85473500 -13.99612800
C 1.64447900 8.94295500 -6.63363300
H 1.65419000 7.85112600 -6.71587100
H 0.84336400 9.22033100 -5.94611300
H 2.60936300 9.27717100 -6.25229800
C 1.47305900 6.73010600 -12.04755700
H 0.62545100 6.46331000 -12.68165100
H 1.51786900 6.00857600 -11.22517400
H 2.40424700 6.68141800 -12.61201600
C 0.08454200 12.07749800 -14.17196900
H -0.74828400 12.78152100 -14.10638700
H -0.22564100 11.25673700 -14.82619200
H 0.96111400 12.56717400 -14.59500300
C 0.26959500 14.23671000 -8.69581800
H 0.15666500 14.09330000 -7.61627400
H -0.65554300 14.67740300 -9.07255500
H 1.11223700 14.90285900 -8.88016600

(Carbene)-[Rh_2](AcO)$_4$ ^{1}A,

52
Rh 5.92510700 12.60543700 6.97896200
Rh 8.00133000 12.69219000 5.66923400

O 6.98410300 13.89433200 4.33084300
O 8.48922200 14.38765900 6.75863700
O 8.83691100 11.47011100 7.11864700
O 7.31491000 11.01994200 4.66016600
O 5.06339300 13.81767100 5.53843200
O 6.58230900 14.29515000 7.98458500
O 6.92367500 11.39566200 8.33540500
O 5.40822700 10.92150600 5.88521800
C 5.76322100 14.20032400 4.55325200
C 7.68475200 14.82286200 7.65202400
C 8.13272200 11.09054600 8.11738200
C 6.19321000 10.49677300 4.98832100
C 9.75694200 12.75992000 4.67736800
C 10.59938000 11.68823600 4.28817400
C 10.24312500 10.34310200 4.58837600
C 11.83318200 11.92849100 3.61511800
C 11.07655200 9.29429700 4.23717200
H 9.31287100 10.14944000 5.10151700
C 12.66350400 10.88497900 3.25066700
H 12.12874800 12.94437200 3.37709400
C 12.27035100 9.58078400 3.57098300
H 10.80832400 8.27175300 4.47183200
H 13.59738800 11.07035500 2.73511800
Br 13.44082900 8.10468900 3.06913500
C 10.20991900 14.14349500 4.43725800
O 11.01114800 14.55509200 5.44428400
O 9.85796900 14.83467100 3.50300700
C 11.26404800 15.95591800 5.51436000
H 10.39106200 16.46352800 5.93050800
H 11.49875500 16.36099900 4.52919800
C 8.83825900 10.21277800 9.12146000
H 9.37226000 9.41364600 8.60231800
H 9.57879800 10.81241500 9.65973500
H 8.12625900 9.79395800 9.83207000
C 5.12169500 15.12598700 3.54884700
H 5.48656600 16.14314300 3.72471900
H 5.40960600 14.83801900 2.53597900
H 4.03713000 15.11328600 3.65591500
C 8.09957400 16.09775600 8.34429400
H 9.16536800 16.06648000 8.57823400
H 7.93012000 16.93821900 7.66313500
H 7.51276700 16.25050500 9.24990900
C 5.79748100 9.26026900 4.21993800
H 5.76057000 9.49154400 3.15201000
H 6.55765300 8.48736700 4.36439700
H 4.82774400 8.89353700 4.55477700
C 12.45762100 16.16166900 6.44193100
Cl 13.90929900 15.33638400 5.77730000
Cl 12.75851100 17.93060200 6.54352400
Cl 12.10817200 15.51712100 8.08265900
